# Facile Conversion of α‐Amino Acids into α‐Amino Phosphonates by Decarboxylative Phosphorylation using Visible‐Light Photocatalysis

**DOI:** 10.1002/anie.202207063

**Published:** 2022-08-04

**Authors:** Dominik Reich, Adam Noble, Varinder K. Aggarwal

**Affiliations:** ^1^ School of Chemistry University of Bristol Cantock's Close Bristol BS8 1TS UK

**Keywords:** Amino Acids, Phosphorylation, Photoredox Catalysis, Radical–Polar Crossover, Reaction Mechanisms

## Abstract

Amino phosphonates exhibit potent inhibitory activity for a wide range of biological processes due to their specific structural and electronic properties, making them important in a plethora of applications, including as enzyme inhibitors, herbicides, antiviral, antibacterial, and antifungal agents. While the traditional synthesis of α‐amino phosphonates has relied on the multicomponent Kabachnik‐Fields reaction, we herein describe a novel and facile conversion of activated derivatives of α‐amino acids directly to their respective α‐amino phosphonate counterparts via a decarboxylative radical–polar crossover process enabled by the use of visible‐light organophotocatalysis. The novel method shows broad applicability across a range of natural and synthetic amino acids, operates under mild conditions, and has been demonstrated to successfully achieve the late‐stage functionalization of drug molecules.

## Graphical abstract

The facile conversion of α‐amino acids to the respective α‐amino phosphonates via their redox active esters using organophotoredox catalysis is reported. A novel photocatalytic phosphorylation combines radical–polar crossover with Arbuzov‐type reactivity, providing an operationally simple, high yielding and general synthetic approach for the generation of α‐amino phosphonates.

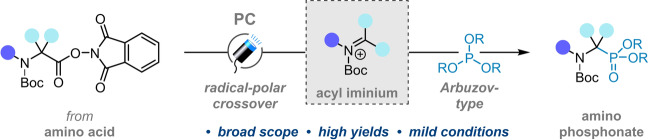

Amino phosphonic acids are analogues of amino acids in which a carboxylic acid group is replaced by phosphonic acid or phosphonate. The diverse biological activity of α‐amino phosphonic acids and their derivatives was discovered over half a century ago,[^1^, ^2^] and has led to wide‐spread interest amongst chemists, biologists, pharmacologists and physicians.[^3^, ^4^, ^5^, ^6^, ^7^] In drug design, α‐amino phosphonic acids often serve as surrogates for α‐amino acid moieties, forming a unique compound class for the construction of inhibitors, particularly for proteases and ligases.^[5]^ This mode of action relies on the fact that the structure of the phosphonic acid moiety mimics the tetrahedral intermediate of amide bond hydrolysis (a so‐called transition state analogue), resulting in the specific complexation of α‐amino phosphonic acids in enzyme active sites.^[8]^ Important examples include alafosfalin, an antibacterial and antifungal agent;^[9]^ phospholeucine, a potent inhibitor of leucine aminopeptidase;^[10]^ and the naturally occurring tripeptide angiotensin converting enzyme inhibitor K‐26 (Scheme 1A).^[11]^

**Scheme 1:**
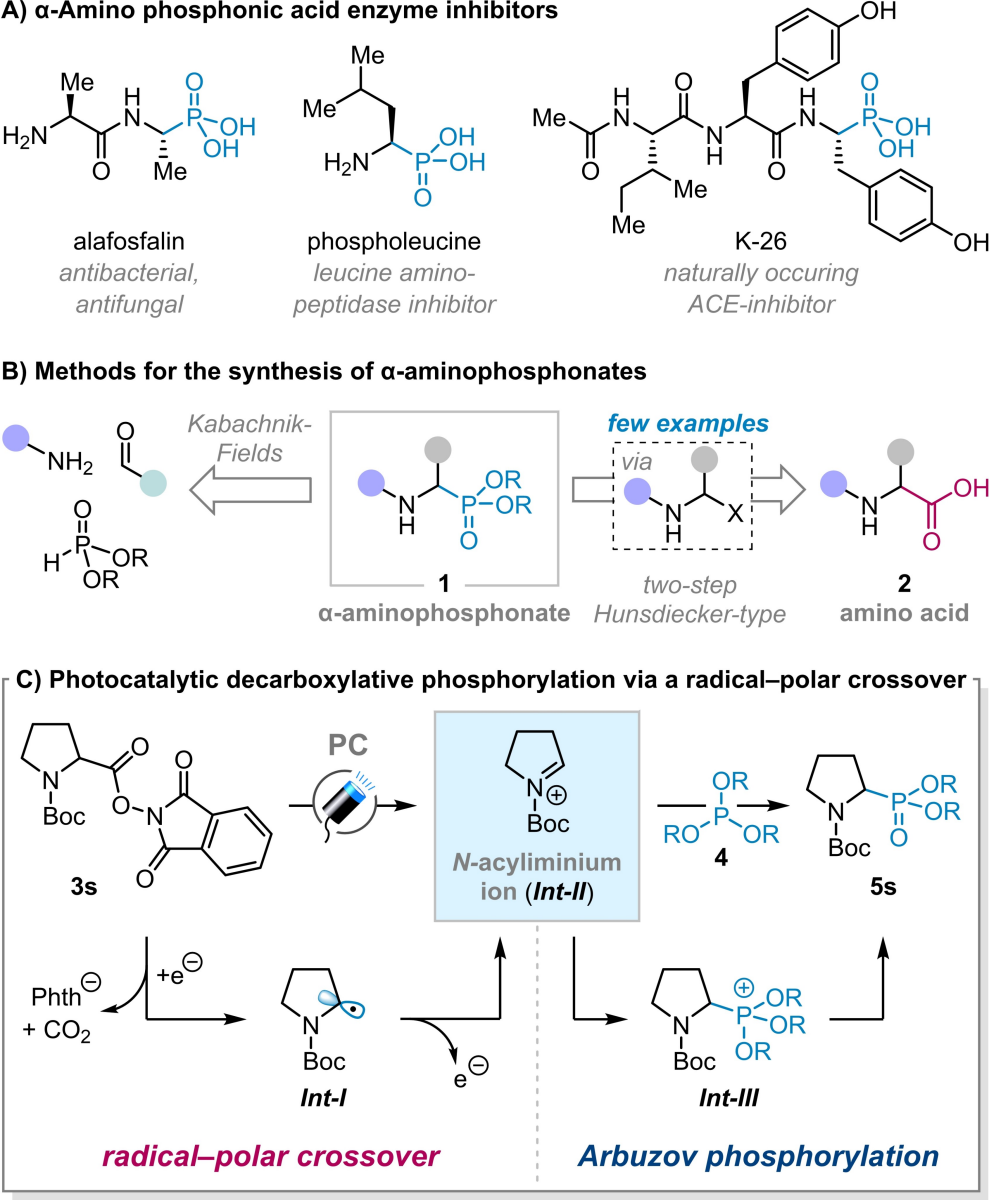
Bioactive examples and synthetic approaches to amino phosphonic acids.

The great potential for application of α‐amino phosphonic acids in drug design has stimulated significant research into their synthesis.^[7]^ The majority of reported methods are simple variations of the Kabachnik‐Fields reaction (Scheme 1B), which was first reported 70 years ago.[^7^, ^12^, ^13^] This powerful three component phospha‐Mannich reaction between a carbonyl, an amine, and dialkyl or trialkyl phosphites is considered the most versatile method for the generation of α‐amino phosphonates **1**, and, as a result, has been developed extensively to enable rapid assembly of structurally diverse analogues.[^14^, ^15^] However, given that α‐amino phosphonic acids are most commonly used as transition state analogues of α‐amino acids **2**, arguably, the most attractive route to their synthesis would be by direct substitution of the carboxylic acid group in the original α‐amino acid (or peptide) for a phosphonate. Such decarboxylative phosphorylations would benefit from the ready availability of α‐amino acids and, in doing so, would avoid the requirement for de novo synthesis of phosphonate analogues. Despite these advantages, decarboxylative phosphorylation of amino acids remains underdeveloped (and unused) as virtually all reports of such decarboxylations principally serve the formation of electrophilic “Hunsdiecker‐type intermediates” (see Scheme 1B), such as α‐haloamines^[16]^ and aminals.[^17^, ^18^, ^19^, ^20^, ^21^, ^22^] The initial oxidation step requires strong stoichiometric oxidants[^16^, ^17^, ^18^, ^19^, ^20^] or electro‐chemical methods.[^21^, ^22^, ^23^] Subsequently, the Hunsdiecker‐type intermediates generated are, in a second separate step, reacted with strong Lewis acids, reforming an iminium ion and enabling phosphorylation.[^24^, ^25^, ^26^, ^27^] The use of strong oxidants and strong Lewis acids inevitably limits substrate scope.

In stark contrast to this Hunsdiecker‐type two‐step approach,[^19^, ^23^] visible‐light photoredox catalysis provides a simple and mild pathway for decarboxylative functionalizations, and has indeed been used extensively to convert carboxylic acids and their derivatives into alkyl radical intermediates.[^28^, ^29^, ^30^] However applying such a process to the synthesis of α‐amino phosphonates is challenging because (i) alkyl radicals rarely undergo productive radical addition to most phosphorus compounds and (ii) many phosphorus sources, such as triphenylphosphine, are redox active.[^31^, ^32^] To overcome these challenges, we propose a fundamentally different strategy: the generation of an *N*‐acyliminium species in situ from readily available α‐amino acid derivatives via a radical–polar crossover process,[^33^, ^34^, ^35^, ^36^] followed by trapping with a nucleophilic P^III^ reagent in an Arbuzov‐type phosphorylation (Scheme 1C).[^37^, ^38^] This approach would circumvent the problematic addition of alkyl radicals to phosphorus and avoids the use of strong oxidants and strong Lewis acids that are invariably required when using Hunsdiecker‐type intermediates.

We were mindful of a series of challenges which could impede the development of this desired protocol: (i) oxidation of the α‐amino radical (***Int‐I***) to the respective *N*‐acyliminium ion (***Int‐II***) would likely be difficult in the presence of a P^III^ reagent, a reductant; (ii) selective reaction of the *N*‐acyliminium ion with P^III^ reagent would have to occur over all other nucleophilic species present in the reaction mixture; and (iii) the Arbuzov‐type reactivity of the resulting phosphonium ion (***Int‐III***) must be maintained and promoted by a suitable nucleophile. Notwithstanding these challenges, we herein describe the successful realization of this plan and report a comprehensive strategy for the photocatalyzed decarboxylative phosphorylation of amino acid derivatives by the action of visible‐light.

At the outset of our studies, we chose *N*‐hydroxyphthalimide esters (“redox active esters”)^[39]^ of *N*‐Boc‐protected α‐amino acids as potential starting materials for the reaction due to their reactivity, ease of synthesis, stability, and high solubility in organic solvents. Accordingly, our initial investigation of the proposed α‐amino phosphonate synthesis focused on the reaction of *N*‐Boc α‐amino acid *N*‐hydroxyphthalimide esters **3** with trimethyl phosphite under irradiation with visible‐light. Following optimization studies with proline derivative **3 s** (see Supporting Information), standard conditions were established employing organophotocatalyst 4CzIPN (2 mol %),^[40]^ trifluoroacetic acid (TFA, 1.5 equiv) and trimethyl phosphite (3 equiv) in acetonitrile (0.1 M) under irradiation (40 W blue LED) for 2 h, which pleasingly delivered the desired α‐amino phosphonate **5 s** in excellent yield (91 % by ^1^H NMR). Control reactions highlighted the importance of both light and photocatalyst in promoting the reaction, as **5 s** was not observed in their absence (see Supporting Information). Use of triethyl phosphite gave diethyl phosphonate **5 s′** in 70 % yield. When the reaction was performed without TFA as an additive, the rate of reaction was significantly diminished, with **5 s** formed in only 23 % after 14 h irradiation whereas in the presence of TFA, the reaction was essentially complete within 20 min as determined by ReactIR.

We then turned our attention to the scope of *N*‐hydroxyphthalimide esters **3** in the decarboxylative phosphorylation (Scheme 2). Pleasingly, derivatives of proteinogenic Boc‐protected α‐amino acids with aliphatic and aromatic side chains **5 a**–**5 h**, including the protected form of amino‐peptidase inhibitor phospholeucine **5 d** (see Supporting Information for hydrolysis to phospholeucine), delivered the α‐amino phosphonate products in good to excellent yields. Amino acids with nitrogen‐containing functional groups, arginine and lysine, also delivered the products in excellent to near quantitative yield (**5 i**, **5 k**) when the side chain was protected with Boc‐ and trifluoroacetyl‐groups, respectively. The successful formation of lysine‐derived product **5 k** is in contrast to previously reported phosphorylations proceeding via initial Hunsdiecker‐type decarboxylation, which resulted in undesired cyclization of the amine‐containing side chain onto the *N*‐acyliminium intermediate, leading to piperidine products.^[19]^ Aspartate and glutamate, with acid‐containing side chains, successfully underwent the reaction as the respective *tert*‐butyl and benzyl esters (**5 l**, **5 m**). Cyclic acetals of threonine, serine and cysteine were amenable to the reaction, delivering the desired products (**5 na**, **5 o**, **5 p**), albeit in low diastereoselectivities. Although the d.r. could not be improved when more sterically demanding tribenzylphosphite was used, the corresponding α‐amino dibenzyl phosphonate **5 nb** could be obtained in moderate yield with facile separation of the *cis* and *trans* isomers (see Supporting Information). Glycine derived **3 r** gave a lower yield of the phosphorylated product **5 r**, likely due to challenging formation and subsequent oxidation of the intermediate primary radical. A number of non‐canonical amino acid derivatives were tested and well tolerated (**5 t**–**5 z**), although fluorinated proline derivative **5 x** was formed in diminished yield presumably due to electronic factors. Gratifyingly, dipeptide **3 aa**, and derivatives of drug molecules acetyl captopril and fosinopril gave good to excellent yields of the respective α‐amino phosphonates (**5 aa**, **5 ab**, **5 ac**) in a late‐stage functionalization approach. Unfortunately, histidine and acyclic serine derivatives were unsuccessful in this transformation. Lastly, we were pleased to find that in addition to the Boc‐group, several substituents on nitrogen proved compatible in the reaction (methyl, acetyl, Fmoc, Cbz, Bz, Ts), providing the phosphorylation products in moderate to good yields (**5 ad**–**5 ai**).

**Scheme 2:**
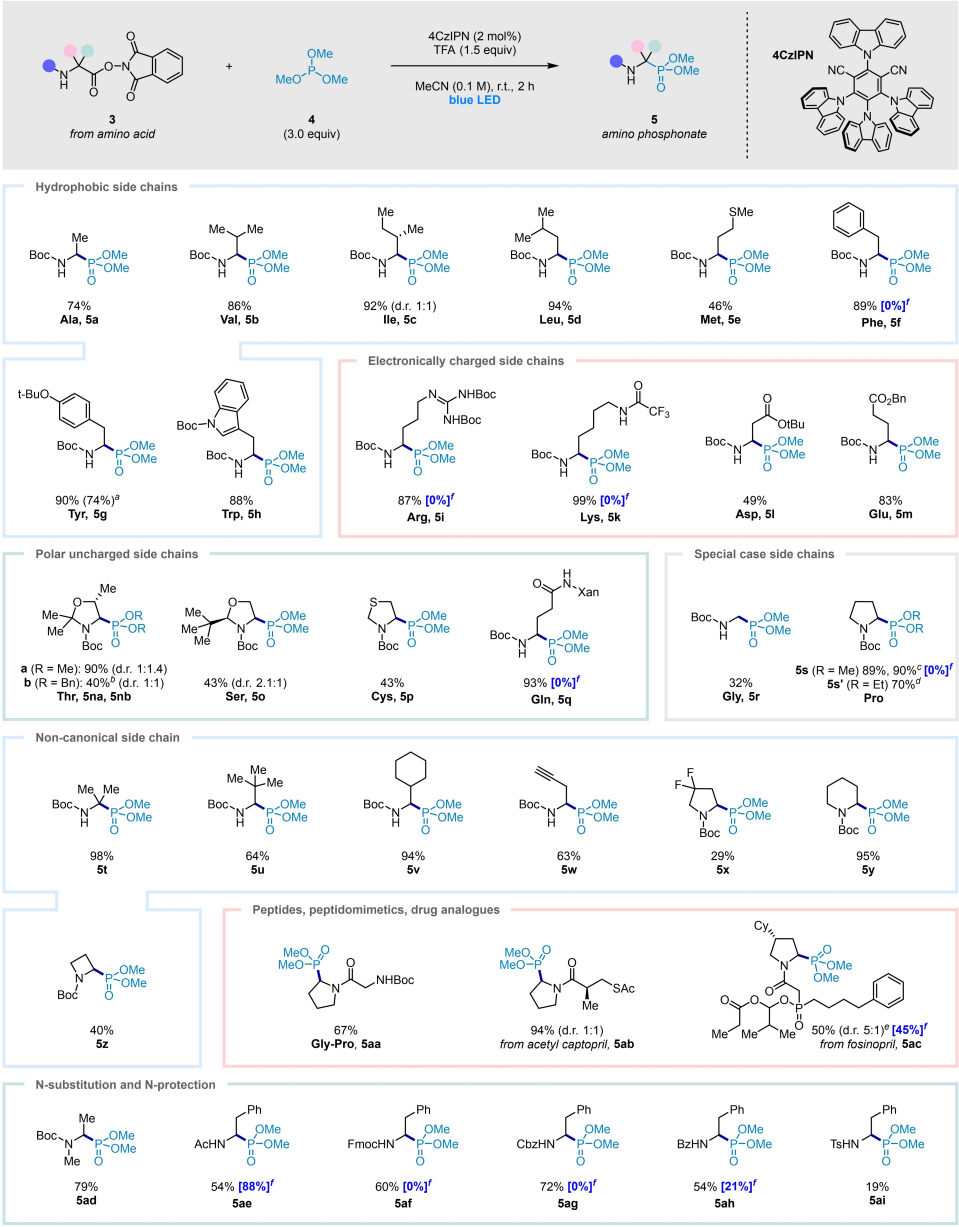
Optimized conditions and scope of the photocatalytic decarboxylative phosphorylation. Standard conditions: **3** (0.2 mmol), **4** (0.6 mmol), 4CzIPN (2 mol %), trifluoroacetic acid (TFA, 0.3 mmol), in acetonitrile (2.0 mL), 40 W blue LED, 2 h. Three letter code for parent N‐Boc amino acids shown. Yields are of isolated products. ^a^ Yield of large scale reaction (2.4 mmol); ^b^ tribenzyl phosphite used; ^c^ Ir[(dtbppy)(ppy)_2_]PF_6_ (1 mol %) used; ^d^ triethyl phosphite used; ^e^ starting **3 ac** d.r.=5 : 1; Cy, cyclohexyl; Xan, xanthenyl; ^f^ reaction yield (^1^H NMR) using conditions from current optimum methodology by Hernández et al. (see Supporting Information for protocol).^[19]^

In order to compare our method with the current optimum methodology employing Hernández's^[19]^ Hunsdiecker‐type decarboxylative phosphorylations, we attempted the synthesis of a series of compounds using this protocol (**5 f**, **5 i**, **5 k**, **5 q**, **5 s**, **5 ac** and **5 ae**–**5 ah**). Whilst products were obtained in the cases of **5 ac**, **5 ae** and **5 ah** (albeit **5 ah** in considerably lower yield), no product formation was observed for **5 f**, **5 i**, **5 k**, **5 q**, **5 s**, **5 af** and **5 ag** (Scheme 2). This highlights one of the limitations of Hernández's methodology, being restricted to amides, which are of course more difficult to manipulate.

To our surprise, when our decarboxylative phosphorylation was applied to acyclic cysteine‐derived esters **3 ak** and **3 al**, containing methyl‐ and trityl‐protected thiols, respectively, both produced the identical product **5 a**, which is also the product formed upon phosphorylation of the alanine‐derived ester **3 a**. We believe that in this case, after decarboxylation, the α‐amino radical undergoes elimination of a thiyl radical to give enamine intermediate ***Int‐IV*** (Scheme 3A). Subsequent protonation of the enamine gives the desulfurized *N*‐acyliminium ***Int‐II***_***a***_, which is finally captured by the phosphite to give **5 a**. A similar process is also likely responsible for the low yield of tosyl‐protected α‐amino phosphonate **5 ai**, which, upon formation of an α‐amino radical, could eliminate a sulfinyl radical to form an imine (Scheme 3B). The involvement of this competing process was confirmed upon observation of primary amine **7** by LC–MS.

**Scheme 3:**
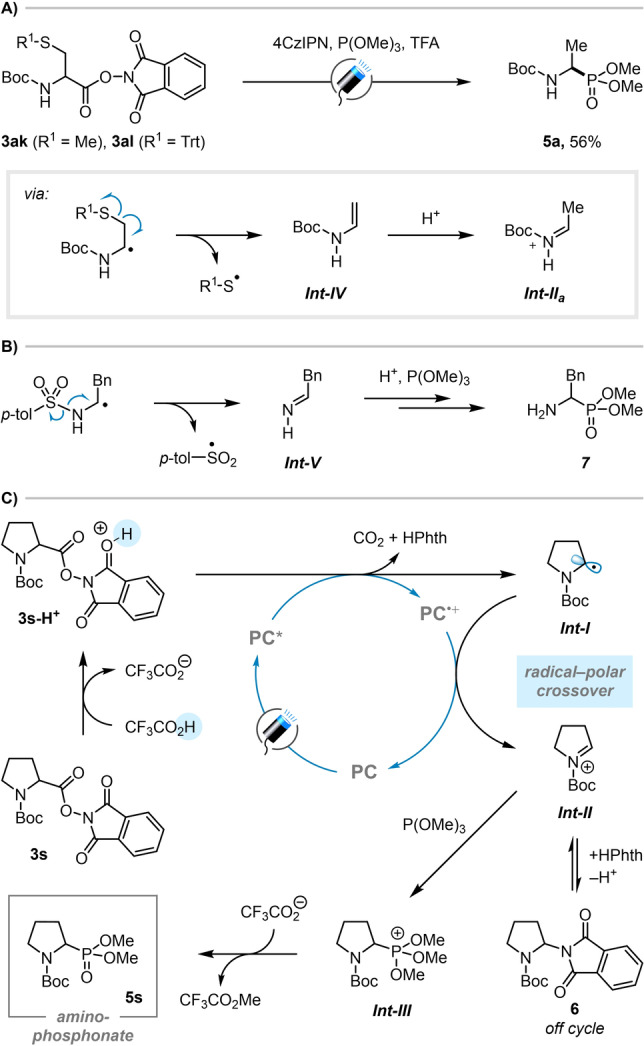
A), B) Mechanism of desulfurization reactions providing additional evidence for radical intermediates and C) proposed mechanism.

Based on these studies, we propose the following mechanism for the decarboxylative phosphorylation (Scheme 3C). After initial excitation of photocatalyst 4CzIPN (**PC**), protonation of redox active ester **3 s** (*E*_1/2_^red^=−1.20 V vs SCE)^[41]^ by TFA significantly lowers its reduction potential, enabling the excited state photocatalyst (PC*) (4CzIPN^+.^/4CzIPN*, *E*_1/2_^red^=−1.04 V vs SCE)[^40^, ^42^] to engage in single electron transfer with **3 s‐H^+^**. Oxidation of trimethyl phosphite by the photocatalyst was ruled out due to its high reduction potential (*E*_1/2_^red^=+1.83 V vs SCE).^[43]^ After decarboxylation and liberation of phthalimide, the resulting α‐amino radical ***Int‐I*** is oxidized by **PC^+^** ⋅ (4CzIPN^+.^/4CzIPN, *E*_1/2_^red^=+1.52 V vs SCE). This radical‐polar crossover process gives rise to *N*‐acyliminium ion ***Int‐II*** directly and regenerates the photocatalyst in its ground state. The *N*‐acyliminium ion ***Int‐II*** is then captured by trimethyl phosphite, leading to phosphonium ion ***Int‐III***, which undergoes an Arbuzov‐type demethylation promoted by trifluoroacetate (methyl trifluoroacetate was observed in the ^19^F NMR) to give the desired α‐amino phosphonate product **5 s**. The *N*‐acyliminium ion ***Int‐II*** can also be captured by phthalimide in an off‐cycle process,[^44^, ^45^] forming undesired aminal **6**, which was experimentally observed by LC–MS. The use of TFA renders this process reversible, enabling the *N*‐acyliminium ion ***Int‐II*** to be regenerated and subsequently captured by trimethyl phosphite. For the first time, this method merges photocatalytic radical‐polar crossover with an Arbuzov type phosphorylation into a highly synthetically useful and mechanistically unprecedented process. The successful realization of this method relied on employing a suitable Bronsted acid, an oxidatively impervious phosphorous source, and a metal‐free, and efficient photocatalyst, which all worked in concert, enabling the rapid, direct formation of bioactive amino phosphonates **5**.

In summary, we have reported a facile conversion of α‐amino acids to their respective α‐amino phosphonates via their redox active esters using organophotoredox catalysis. The reaction is operationally simple, high yielding, proceeds under metal‐free, mild conditions and offers a broad scope across the pool of proteinogenic and synthetic α‐amino acids. Most common N‐protecting groups can be employed in this protocol, and the transformation is amenable to late‐stage functionalization of peptides and drug molecules, giving direct synthetic access to an abundance of previously synthetically intractable α‐amino phosphonates. We expect that due to its simplicity, efficacy, mild conditions and broad scope, this photocatalytic decarboxylative phosphorylation reaction will find widespread use in both academia and industry.

## Conflict of interest

The authors declare no conflict of interest.

## Supporting information

As a service to our authors and readers, this journal provides supporting information supplied by the authors. Such materials are peer reviewed and may be re‐organized for online delivery, but are not copy‐edited or typeset. Technical support issues arising from supporting information (other than missing files) should be addressed to the authors.

Supporting Information

## Data Availability

The data that support the findings of this study are available in the Supporting Information of this article.
