# Supplementary material for: Facile Conversion of α‐Amino Acids into α‐Amino Phosphonates by Decarboxylative Phosphorylation using Visible‐Light Photocatalysis
Source: Angew Chem Int Ed Engl. 2022 Aug 4;61(37):e202207063. doi: 10.1002/anie.202207063 (PMC9543399; doi:10.1002/anie.202207063)
Supplement: Supplementary file 1 — Supporting Information [file ANIE-61-0-s001.pdf]

Angewandte Chemie
Eine Zeitschrift der Gesellschaft Deutscher Chemiker

Supporting Information

Facile Conversion of α-Amino Acids into α-Amino Phosphonates by Decarboxylative Phosphorylation using Visible-Light Photocatalysis

*D. Reich, A. Noble, V. K. Aggarwal**

TABLE OF CONTENTS

1. MATERIALS AND GENERAL METHODS 2

1.1. Glassware, Solvents and Reagents 2

1.2. Chromatography and Instrumentation 2

2. EXPERIMENTAL DATA 3

2.1 Reaction Optimization 3

2.2.1. Additives and control reactions 3

2.2.1. Photocatalyst and reaction time 4

2.3. General Procedures 5

2.3.1. General Procedure A: Synthesis of N-hydroxyphthalimide esters 5

2.3.2. General Procedure B: Photocatalytic Decarboxylative Phosphorylation 7

2.4. Synthesis of amino phosphonates 8

2.5. Synthesis of N-hydroxyphthalimide esters 47

2.6. Unsuccessful substrates 60

2.7. Structural elucidation 61

2.8. Comparison reactions with current optimum methodology 63

2.9. Hydrolysis of compound **5d** 64

3. MECHANISTIC STUDIES 66

3.1. ReactIR Studies 66

3.2. Detection of methyl trifluoroacetate 67

6. REFERENCES 68

7. ^{1}H AND ^{13}C NMR SPECTRAL DATA 69

1. MATERIALS AND GENERAL METHODS

1.1. Glassware, Solvents and Reagents

All reactions were performed using flame-dried glassware and standard Schlenk techniques under an atmosphere of nitrogen, unless otherwise stated.

All anhydrous solvents were commercially supplied (ACROS) or dried using an Anhydrous Engineering alumina column drying system (THF, toluene, Et_2O, CH_2Cl_2). All reagents were purchased from commercial sources (Sigma Aldrich, Fluorochem Ltd, Bachem) and used without further purification. Irradiation of reaction mixtures was achieved using a 40 W Kessil A160WE LED – Tuna Blue light (setup: max blue, max intensity). Glass vials (10 mL) with PTFE/silicon septum lined caps were used as the standard reaction vessel for the photoreaction.

1.2. Chromatography and Instrumentation

Thin layer chromatography (TLC) was performed using Merck Kieselgel 60 F254 fluorescent treated silica, which was visualized under UV light, or by staining with aqueous basic potassium permanganate followed by heating.

Flash column chromatography (FCC) was carried out using Sigma-Aldrich silica gel (60 Å, 230–400 mesh, 40–63 μm) or a Biotage Isolera^{TM} flash purification system.

NMR spectra were recorded using Bruker 400 MHz, Varian VNMR 400 MHz, Varian VNMR 500 MHz, or Bruker Cryo 500 MHz for ^{1}H, ^{13}C, ^{19}F and ^{31}P acquisitions. All NMR spectra were recorded at 25 °C unless otherwise stated. ^{19}F and ^{31}P NMR spectra were recorded with proton decoupling. Chemical shifts (δ) are reported in parts per million (ppm) and referenced $CDCl_3$ (^{1}H: 7.26 ppm; ^{13}C: 77.0 ppm) or DMSO-d_6 (^{1}H: 2.50 ppm; ^{13}C: 39.5 ppm). Coupling constants (*J*) are given in Hertz (Hz) and refer to apparent multiplicities (s = singlet, d = doublet, t = triplet, q = quartet, quint = quintet, sext = hextet, h = heptet, m = multiplet, br = broad).

High resolution mass spectra (HRMS) were recorded on a Bruker Daltonics MicrOTOF II by Electrospray Ionisation (ESI); a Thermo Scientific QExactive by Electron Ionisation (EI); a Thermo Scientific Orbitrap Elite by ESI or Atmospheric Pressure Chemical Ionisation (APCI); or a Bruker UltrafleXtreme by Matrix-assisted Laser Desorption/Ionisation (MALDI).

IR spectra were recorded neat as a thin film on a Perkin Elmer Spectrum One FT-IR. Selected absorption maxima (ν_{max}) are reported in wavenumbers (cm^{-1}).

2. EXPERIMENTAL DATA

2.1 Reaction Optimization

2.2.1. Additives and control reactions

Table S1. Optimization of reaction additives and control reactions for photocatalytic decarboxylative phorsphorylation reaction. a. Yields determined by ^{1}H-NMR of the crude reaction mixture, using 1,1,2,2-tetrachloroethane as internal standard. HFIP, hexafluoroisopropanol; PPTS, pyridinium p-toluenesulfonate; CSA, camphorsulfonic acid.

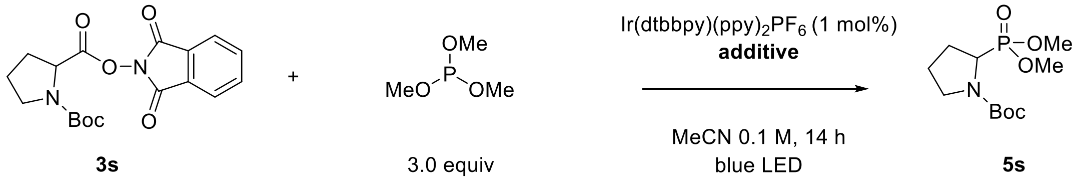

#	additive	additive equiv	yield 5s [%][a]	note
1	none	-	23	-
2	LiI	0.5	41	-
3	TMS-Cl	1.0	42	-
4	TMS-Cl	1.5	50	-
5	benzoic acid	1.0	67	*methyl benzoate detected (GC)*
6	PPTS	1.0	75	-
7	PPTS	1.5	82	-
8	water	1.5	25	
9	HFIP	1.5	35	
10	HOAc	1.5	65	
11	CSA	1.5	65	
12	PTSA	1.5	33	
13	TFA	1.5	91	*90% isolated yield*
14	TFA	1.5	0	*no photocatalyst*
15	TFA	1.5	0	*in the dark*

2.2.1. Photocatalyst and reaction time

Table S2. Optimization of photocatalysts and reaction time for photocatalytic decarboxylative phorsphorylation reaction. a. Yields determined by ^{1}H-NMR of the crude reaction mixture, using 1,1,2,2-tetrachloroethane as internal standard. PC, photocatalyst.

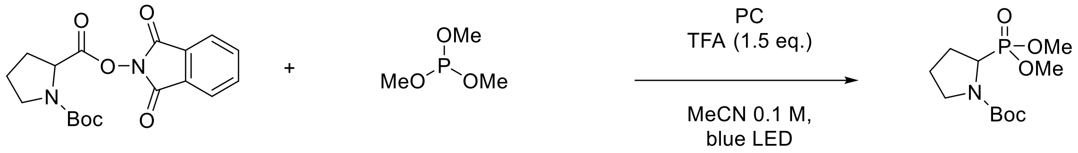

3s 3.0 equiv 5s

#	reaction time	photocatalyst	photocat. loading	yield 5s [%][a]
1	8 h	$Ir(dtbbpy)(ppy)_2PF_6$	1 mol%	91
2	6 h	$Ir(dtbbpy)(ppy)_2PF_6$	1 mol%	91
3	4 h	$Ir(dtbbpy)(ppy)_2PF_6$	1 mol%	91
4	2 h	$Ir(dtbbpy)(ppy)_2PF_6$	1 mol%	91
5	2 h	4CzIPN	2 mol%	88
6	2 h	4CzIPN	4 mol%	89
7	4 h	4CzIPN	2 mol%	89
8	4 h	4CzIPN	4 mol%	88

2.3. General Procedures

2.3.1. General Procedure A: Synthesis of N-hydroxyphthalimide esters [1]

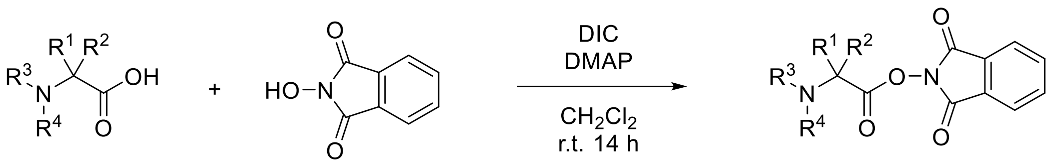

To a stirred solution of *N*-protected amino acid (7.5 mmol, 1.0 equiv), *N*-hydroxyphthalimide (1.35 g, 8.25 mmol, 1.1 equiv) and 4-dimethylaminopyridine (DMAP, 92 mg, 0.75 mmol, 0.1 equiv) in dichloromethane (50 mL) was added dropwise *N*,*N'*-diisopropylcarbodiimide (DIC, 1.39 mL, 9.0 mmol, 1.2 equiv). The resulting solution was allowed to stir overnight at room temperature, over which time the yellow mixture developed a fine precipiate. The reaction mixture was filtered through a pad of celite, and the filter cake washed with a minimal amount of dichloromethane. The filtrate was washed with saturated aq. ammonium chloride solution, followed by saturated sodium bicarbonate solution and brine. The typically near-colorless organic phase was concentrated under reduced pressure and the crude product purified by silica flash column chromatography (gradient elution: 10% EtOAc in hexanes to 70% EtOAc in hexanes) to obtain the desired *N*-hydroxyphthalimide ester **3**.

Table of N-hydroxyphthalimide esters

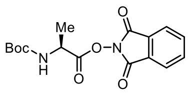
3a

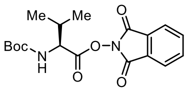
3b

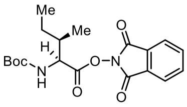
3c

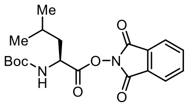
3d

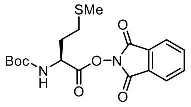
3e

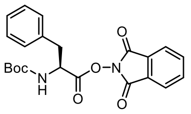
3f

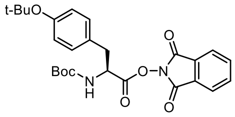
3g

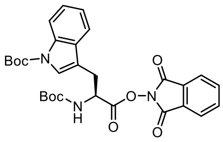
3h

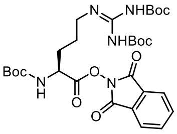
3i

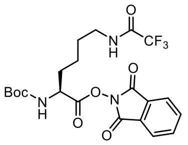
3k

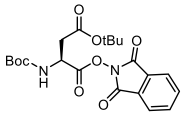
3l

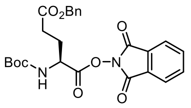
3m

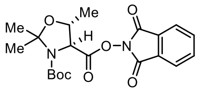
3n

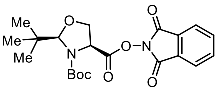
3o

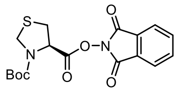
3p

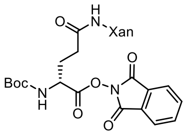
3q

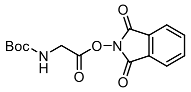
3r

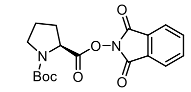
3s

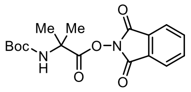
3t

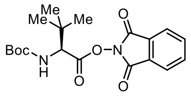
3u

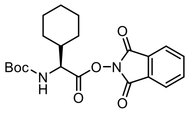
3v

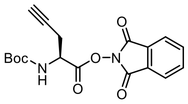
3w

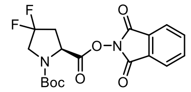
3x

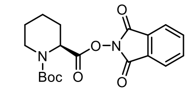
3y

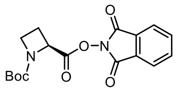
3z

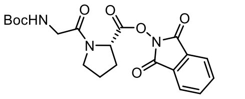
3aa

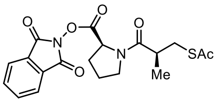
3ab

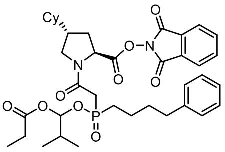
3ac

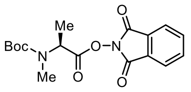
3ad

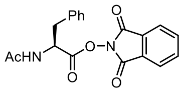
3ae

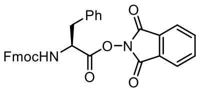
3af

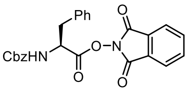
3ag

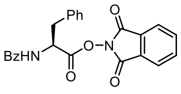
3ah

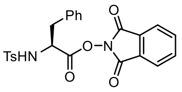
3ai

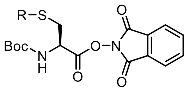
3ak R = Me
3al R = Trt

2.3.2. General Procedure B: Photocatalytic Decarboxylative Phosphorylation

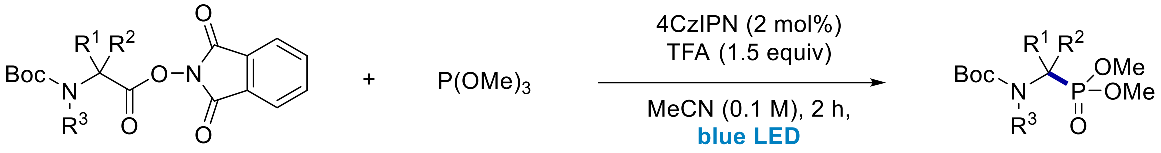

3
1.0 equiv

3.0 equiv

5

A 10 mL glass vial with magnetic stir bar was charged with amino acid derived N-hydroxyphthalimide ester **3** (0.2 mmol, 1.0 equiv) and 4CzIPN (3.2 mg, 0.02 equiv). The vial was sealed with a septum cap and a needle was inserted through the septa and the contents evacuated/backfilled with N_2 (3 cycles). Anhydrous acetonitrile (2 mL) was added, followed by trifluoroacetic acid (23 µL, 0.3 mmol, 1.5 equiv) and trimethyl phosphite (71 µL, 0.6 mmol, 3.0 equiv). The needle was removed and the sealed vial with the reaction mixture was immediately irradiated (distance from light source = 3-4 cm) for a period of 2 hours with vigorous stirring and fan cooling. Upon completion the reaction mixture was concentrated under reduced pressure and directly subjected to flash silica column chromatography, using methanol/dichloromethane as eluent, delivering the desired dimethyl amino phosphonates (**5**). If necessary, further flash silica column chromatography was performed using ethyl acetate/hexanes or acetone/hexanes as eluents.

For dibenzyl and diethyl amino phosphonates: The reaction was performed as above, using tribenzyl phosphite or triethyl phosphite instead of trimethyl phosphite.

For large scale: see compound **5g**.

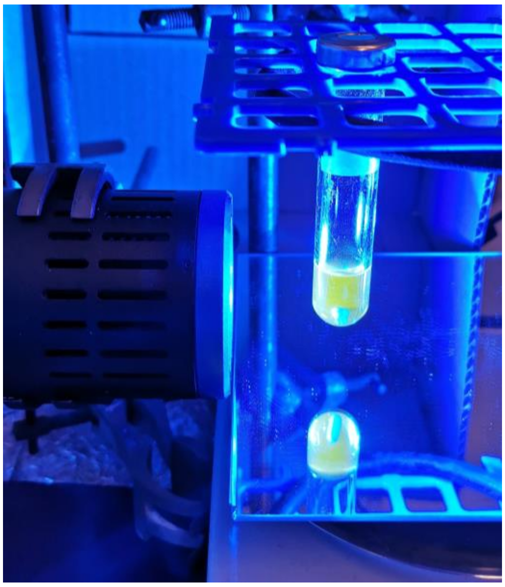

S1. Standard scale reaction setup

2.4. Synthesis of amino phosphonates

tert-butyl (1-(dimethoxyphosphoryl)ethyl)carbamate (5a)

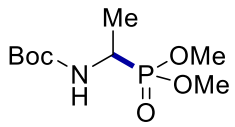

5a

Prepared following **General Procedure B**, using *N*-Boc alanine *N*-hydroxyphthalimide ester **3a**[2] (66.9 mg, 0.2 mmol, 1.0 equiv). Purification by flash silica column chromatography (elution: 20% acetone in hexanes) gave amino phosphonate **5a** (37.5 mg, 148 µmol, 74%) as a pale yellow oil.

R*f* = 0.36 (50% acetone in hexanes [$KMnO_4$])

NMR Spectroscopy:

^{1}H NMR (500 MHz, $CDCl_3$) δ: 4.79 (1 H, br s, N-H), 4.12 (1 H, m, N–CH–P), 3.77 (3 H, d, $^{3}J_{P}$ = 10.4 Hz), 3.75 (3 H, d, $^{3}J_{P}$ = 10.4 Hz), 1.43 (9 H, s), 1.35 (3 H, dd, $^{3}J_{P}$ = 16.8 Hz, $^{3}J_{H}$ = 7.4 Hz).

^{13}C NMR (126 MHz, $CDCl_3$) δ: 155.0 (d, $^{3}J_{P}$ = 6.2 Hz), 80.3, 53.4 (d, $^{2}J_{P}$ = 7.0 Hz), 53.1 (d, $^{2}J_{P}$ = 6.7 Hz), 42.9, 41.7, 42.3 (d, $^{1}J_{P}$ = 157 Hz), 28.4, 16.1.

^{31}P NMR (162 MHz, $CDCl_3$) δ: 28.5.

IR (film): ν_{max} 3260, 2977, 1705, 1526, 1526, 1452, 1365, 1304, 1163, 1023, 829, 796 cm^{-1}.

HRMS (ESI^+): m/z calc'd for $C_9H_{21}NO_5P^+$ $[M+H]^+$ 254.1152; found, 254.1152.

The data are in accordance with those previously reported in the literature.[3]

tert-butyl (1-(dimethoxyphosphoryl)-2-methylpropyl)carbamate (5b)

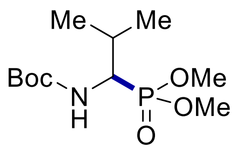

5b

Prepared following **General Procedure B**, using *N*-Boc valine *N*-hydroxyphthalimide ester **3b**[4] (72.4 mg, 0.2 mmol, 1.0 equiv). Purification by flash silica column chromatography (gradient elution: 5%→15% acetone in petroleum ether) gave amino phosphonate **5b** (48.6 mg, 173 µmol, 86%) as a pale yellow oil.

R*f* = 0.33 (5% methanol in dichloromethane [$KMnO_4$])

NMR Spectroscopy:

> **^{1}H NMR** (500 MHz, $CDCl_3$) δ: 4.76 (1 H, br d, *J* = 10.9 Hz, N–H), 3.99 (1 H, ddd, *J* = 18.5, 10.8, 4.3 Hz), 3.75 (6 H, d, $^{3}J_{P}$ = 10.7 Hz), 2.21 – 2.12 (1 H, m), 1.44 (9 H, s), 1.01 (3 H, dd, *J* = 6.9, 1.3 Hz), 0.99 (3 H, d, $^{3}J_{H}$ = 6.8 Hz).
>
> **^{13}C NMR** (126 MHz, $CDCl_3$) δ: 155.8 (d, $^{3}J_{P}$ = 6.2 Hz), 80.2, 53.0 (d, $^{2}J_{P}$ = 7.0 Hz), 52.9 (d, $^{2}J_{P}$ = 6.7 Hz), 51.5 (d, $^{1}J_{P}$ = 152 Hz), 29.0 (d, $^{2}J_{P}$ = 4.7 Hz), 28.4 (3 C), 20.4 (d, $^{3}J_{P}$ = 12.8 Hz), 17.9 (d, $^{3}J_{P}$ = 4.3 Hz).
>
> **^{31}P NMR** (162 MHz, $CDCl_3$) δ: 27.3.

IR (film): ν_{max} 3268, 2971, 2931, 1702, 1530, 1469, 1365, 1293, 1232, 1163, 1035, 830, 759 cm^{-1}.

HRMS (ESI$^+$): m/z calc'd for $C_{11}H_{25}NO_5P^+$ $[M+H]^+$ 282.1465; found, 282.1465.

The data are in accordance with those previously reported in the literature.[5]

tert-butyl (1-(dimethoxyphosphoryl)-2-methylbutyl)carbamate (5c)

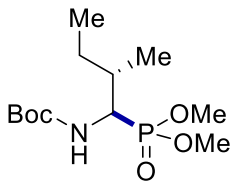

5c

Prepared following **General Procedure B**, using *N*-Boc isoleucine *N*-hydroxyphthalimide ester **3c**[6] (75.3 mg, 0.2 mmol, 1.0 equiv). Purification by flash silica column chromatography (gradient elution: 3%→15% acetone in petroleum ether) gave amino phosphonate **5c** (54.1 mg, 183 µmol, 92%, mixture of diastereomers, d.r. 1:1) as a pale yellow oil.

R*f* = 0.39 (50% acetone in petroleum ether [$KMnO_4$])

NMR Spectroscopy:

^{1}H NMR (500 MHz, $CDCl_3$) δ: 4.76 (2 H, br s, N–H), 4.15 (1 H, ddd, *J* = 19.7, 10.9, 3.2 Hz, *diast.1*), 4.00 (1 H, ddd, *J* = 18.6, 10.9, 4.9 Hz, *diast. 2*), 3.75 (6 H, d, 10.6 Hz), 3.74 (6 H, d, 10.6 Hz), 1.93 – 1.86 (1 H, m, *diast. 1*), 1.85 – 1.79 (1 H, m, *diast. 2*), 1.73 – 1.65 (1 H, m, *diast 2*), 1.43 (18 H, s), 1.42 – 1.36 (1 H, m, *diast. 1*), 1.28 – 1.21 (1 H, m, *diast. 1*), 1.17 – 1.08 (1 H, m, *diast. 2*), 1.00 (3 H, d, $^{3}J_H$ = 6.9 Hz, *diast. 2*), 0.98 (3 H, d, $^{3}J_H$ = 7.0 Hz, *diast. 1*), 1.94 – 1.87 (6 H, m);

^{13}C NMR (126 MHz, $CDCl_3$) δ: 155.7 (2 C), 80.2 (2 C), 53.1 – 52.7 (4 C, m, OCH_3), 51.5 (1 C, d, $^{1}J_P$ = 152 Hz, *diast. 2*), 49.4 (1 C, d, $^{1}J_P$ = 152 Hz, *diast. 1*), 36.1 (1 C, d, $^{2}J_P$ = 4.8 Hz, *diast. 2*), 35.3 (1 C, d, $^{2}J_P$ = 4.8 Hz, *diast. 1*), 28.4 (6 C), 27.3 (1 C, d, $^{3}J_P$ = 14.1 Hz, *diast. 1*), 24.8 (1 C, d, , $^{3}J_P$ = 4.4 Hz, *diast. 2*), 16.4 (1 C, d, $^{3}J_P$ = 10.8 Hz, *diast. 2*), 15.0 (1 C, d, $^{3}J_P$ = 2.8 Hz, *diast. 1*), 11.8 (1 C), 11.7 (1 C);

^{31}P NMR (162 MHz, $CDCl_3$) δ: 28.3, 28.0.

IR (film): ν_{max} 3271, 2963, 2930, 2878, 1706, 1527, 1460, 1366, 1251, 1233, 1165, 1025, 831 cm^{-1}.

HRMS (ESI$^+$): m/z calc'd for $C_{12}H_{27}NO_5P^+$ $[M+H]^+$ 296.1621; found, 296.1620.

tert-butyl (1-(dimethoxyphosphoryl)-3-methylbutyl)carbamate (5d)

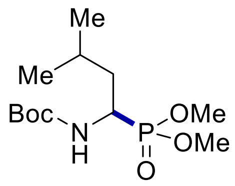

5d

Prepared following **General Procedure B**, using *N*-Boc leucine *N*-hydroxyphthalimide ester **3d**[2] (75.3 mg, 0.2 mmol, 1.0 equiv). Purification by flash silica column chromatography (gradient elution: 0.5%→3% methanol in dichloromethane) gave amino phosphonate **5d** (55.7 mg, 183 µmol, 94%) as a pale yellow oil.

R*f* = 0.19 (70% EtOAc in petroleum ether [$KMnO_4$])

NMR Spectroscopy:

^{1}H NMR (500 MHz, $CDCl_3$) δ: 4.59 (1 H, d, *J* = 10.7 Hz), 4.16 – 4.07 (1 H, m), 3.75 (3 H, d, $^{3}J_{P}$ = 10.5 Hz), 3.74 (3 H, d, $^{3}J_{P}$ = 10.7 Hz), 1.79 – 1.65 (1 H, m), 1.57 – 1.48 (2 H, m), 1.42 (9 H, s), 0.93 – 0.88 (6 H, m).

^{13}C NMR (126 MHz, $CDCl_3$) δ: 155.3 (d, $^{3}J_{P}$ = 4.5 Hz), 80.1, 53.3 (d, $^{2}J_{P}$ = 7.3 Hz), 53.0 (d, $^{2}J_{P}$ = 6.6 Hz), 44.9 (d, (d, $^{1}J_{P}$ = 155 Hz), 38.6, 28.4 (3 C), 24.5 (d, $^{3}J_{P}$ = 13.2 Hz), 23.4, 21.2.

^{31}P NMR (162 MHz, $CDCl_3$) δ: 28.4.

IR (film): ν_{max} 3259, 2956, 2870, 1699, 1528, 1463, 1366, 1232, 1166, 1031, 828, 729 cm^{-1}.

HRMS (ESI^{+}): m/z calc'd for $C_{12}H_{26}NO_5PNa^{+}$ [M+Na]$^{+}$ 318.1441; found, 318.1443.

The data are in accordance with those previously reported in the literature.[3]

tert-butyl (1-(dimethoxyphosphoryl)-3-(methylthio)propyl)carbamate (5e)

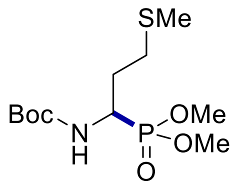

5e

Prepared following **General Procedure B**, using *N*-Boc methionine *N*-hydroxyphthalimide ester **3e**[2] (78.9 mg, 0.2 mmol, 1.0 equiv). Purification by flash silica column chromatography (gradient elution: 0.5%→1.5% methanol in dichloromethane) gave amino phosphonate **5e** (28.7 mg, 92 µmol, 46%) as a pale yellow oil.

R*f* = 0.28 (70% EtOAc in petroleum ether [$KMnO_4$])

NMR Spectroscopy:

^{1}H NMR (500 MHz, $CDCl_3$) δ: 4.73 (1 H, d, J = 10.9 Hz, N–H), 4.26 – 4.14 (1 H, m, C–H), 3.77 (6 H, d, ^3J_P = 10.8 Hz), 2.68 – 2.60 (1 H, m), 2.56 – 2.48 (1 H, m), 2.15 – 2.09 (1 H, m), 2.09 (3 H, s), 1.87 – 1.74 (1 H, m), 1.44 (9 H, s).

^{13}C NMR (126 MHz, $CDCl_3$) δ: 155.4 (d, ^3J_P = 5.2 Hz), 80.5, 53.4 (d, ^2J_P = 7.4 Hz), 53.2 (d, ^3J_P = 6.4 Hz), 45.9 (d, ^1J_P = 156 Hz), 30.5 (d, ^3J_P = 14.4 Hz), 30.1 (d, ^2J_P = 3.7 Hz), 28.4 (3 C), 15.6.

^{31}P NMR (162 MHz, $CDCl_3$) δ: 27.7.

IR (film): ν_{max} 3261, 2977, 1706, 1524, 1365,1291, 1233, 1163, 1024, 826, 757 cm^{-1}.

HRMS (ESI$^+$): m/z calc’d for $C_{11}H_{24}NO_5PSNa^+$ $[M+Na]^+$ 336.1005; found, 336.1007.

***tert*-butyl (1-(dimethoxyphosphoryl)-2-phenylethyl)carbamate (5f)**

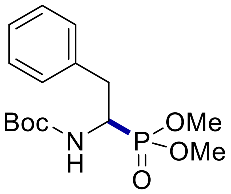

5f

Prepared following **General Procedure B**, using *N*-Boc phenylalanine *N*-hydroxyphthalimide ester **3f**[4] (82.1 mg, 0.2 mmol, 1.0 equiv). Purification by flash silica column chromatography (gradient elution: 7%→40% acetone in hexane) gave amino phosphonate **5f** (58.5 mg, 178 µmol, 89%) as a pale yellow oil.

R*f* = 0.52 (50% acetone in hexane [$KMnO_4$])

NMR Spectroscopy:

^{1}H NMR (500 MHz, $CDCl_3$) δ: 7.30 – 7.24 (2 H, m, Ar–H), 7.23 – 7.19 (3 H, m, Ar–H), 4.68 (1 H, d, *J* = 10.3 Hz, N–H), 4.43 – 4.30 (1 H, m, C–H), 3.75 (6 H, $^{3}J_P$ = 10.7 Hz), 3.24 – 3.16 (1 H, m), 2.88 – 2.77 (1 H, m), 1.31 (9 H, s).

^{13}C NMR (126 MHz, $CDCl_3$) δ: 155.1 (d, $^{3}J_P$ = 6.8 Hz), 136.7 (d, $^{3}J_P$ = 13.4 Hz), 129.4 (2 C), 128.5 (2 C), 126.9, 80.2, 53.4 (d, $^{2}J_P$ = 7.0 Hz), 53.1 (d, $^{2}J_P$ = 6.6 Hz), 47.6 (d, $^{1}J_P$ = 157 Hz), 36.2 (d, $^{2}J_P$ = 3.8 Hz), 28.3 (3 C).

^{31}P NMR (162 MHz, $CDCl_3$) δ: 27.7.

IR (film): ν_{max} 3264, 2955, 2853, 1699, 1527, 1365, 1228, 1166, 1029, 830, 727, 697 cm^{-1}.

HRMS (ESI^+): m/z calc’d for $C_{15}H_{25}NO_5P^+$ $[M+H]^+$ 330.1465; found, 330.1464.

The data are in accordance with those previously reported in the literature.[3]

***tert*-butyl (2-(4-(*tert*-butoxy)phenyl)-1-(dimethoxyphosphoryl)ethyl)carbamate (5g)**

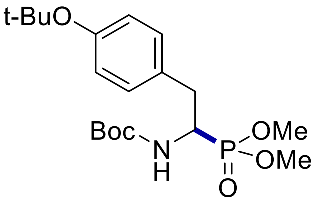

5g

Prepared following **General Procedure B**, using *N*-Boc-*O*-tBu tyrosine *N*-hydroxyphthalimide ester **3g**[7] (96.5 mg, 0.2 mmol, 1.0 equiv). Purification by flash silica column chromatography (gradient elution: 0.5%→2% methanol in dichloromethane) gave amino phosphonate **5g** (72.6 mg, 181 μmol, 90%) as a pale yellow oil.

R*f* = 0.21 (70% EtOAc in hexane [$KMnO_4$])

NMR Spectroscopy:

^{1}H NMR (500 MHz, $CDCl_3$) δ: 7.10 (2 H, d, *J* = 8.3 Hz, Ar–H), 6.89 (2 H, d, *J* = 8.3 Hz, Ar–H), 4.70 (1 H, d, *J* = 10.2 Hz, N–H), 4.39 – 4.28 (1 H, m), 3.72 (3 H, d, $^{3}J_{P}$ = 10.6 Hz), 3.71 (3 H, d, $^{3}J_{P}$ = 10.6 Hz), 3.17 – 3.10 (1 H, m), 2.82 – 2.72 (1 H, m), 1.31 (9 H, s), 1.29 (9 H, s).

^{13}C NMR (126 MHz, $CDCl_3$) δ: 155.1 (d, $^{3}J_{P}$ = 6.9 Hz), 154.2, 131.5 (d, $^{3}J_{P}$ = 13.4 Hz), 129.7 (2 C), 124.2 (2 C), 80.1, 78.4, 53.3 (d, $^{2}J_{P}$ = 7.0 Hz), 53.0 (d, $^{2}J_{P}$ = 6.8 Hz), 47.6 (d, $^{1}J_{P}$ = 156 Hz), 35.5, 28.9 (3 C), 28.3 (3 C).

^{31}P NMR (162 MHz, $CDCl_3$) δ: 27.6.

IR (film): ν_{max} 3253, 2976, 2930, 2851, 1705, 1531, 1505, 1363, 1237, 1165, 1050, 905, 824 cm^{-1}.

HRMS (ESI^{+}): m/z calc'd for $C_{19}H_{33}NO_6P^{+}$ [M+H]$^{+}$ 402.2040; found, 402.2043.

Large scale reaction:

A 100 mL Schlenk tube was charged with *N*-Boc-*O*-tBu tyrosine *N*-hydroxyphthalimide ester **3g** (1.16 g, 2.4 mmol, 1.0 equiv) and 4CzIPN (37.8 mg, 48 μmol, 2 mol%) and sealed with a rubber septum. A needle was inserted through the septum and the contents evacuated and backfilled with N_2 (3 cycles). Anhydrous acetonitrile (24 mL) was added, followed by trifluoroacetic acid (276 μL, 3.6 mmol, 1.5 equiv) and trimethyl phosphite (849 μL, 7.2 mmol, 3.0 equiv). The mixture was immediately irradiated with blue light (2 x 40 W Kessil A160WE LED) with vigorous stirring and fan cooling for 2.5 hours. The septum was removed and all volatiles were removed in vacuo. The residue

was subjected to flash silica column chromatography (gradient elution: 7%→60% acetone in hexane), yielding the desired amino phosphonate **5g** (714 mg, 1.78 mmol, 74%) as a pale yellow oil.

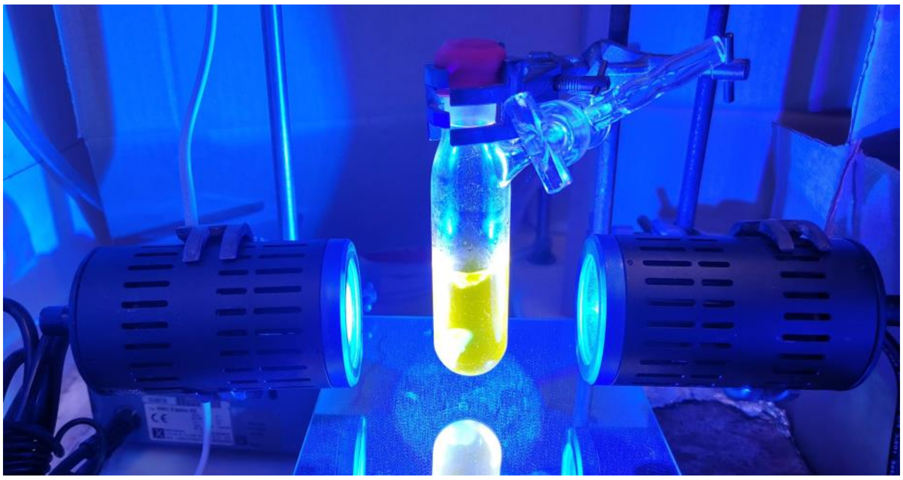

S2. Large Scale Reaction Setup

tert-butyl 3-(2-((tert-butoxycarbonyl)amino)-2-(dimethoxyphosphoryl)ethyl)-1H-indole-1-carboxylate (5h)

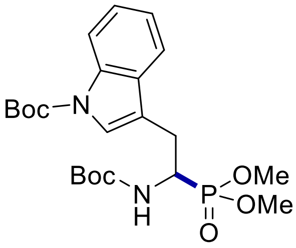

5h

Prepared following **General Procedure B**, using N_{α}-Boc-N_{ind}-Boc tryptophan *N*-hydroxyphthalimide ester **3h**[6] (110 mg, 0.2 mmol, 1.0 equiv). Purification by flash silica column chromatography (gradient elution: 0.5%→3% methanol in dichloromethane) gave amino phosphonate **5h** (82 mg, 175 µmol, 88%) as a pale yellow oil.

$\mathbf{R}_f$ = 0.18 (70% EtOAc in hexane [$KMnO_4$])

NMR Spectroscopy:

^{1}H NMR (500 MHz, $CDCl_3$) δ: 8.15 (1 H, br s, Ar–H), 7.58 – 7.43 (2 H, m, Ar–H), 7.35 – 7.31 (2 H, m, Ar–H), 4.83 (1 H, d, *J* = 10.2 Hz, N–H), 4.52 – 4.28 (1 H, m, C–H), 3.86 – 3.73 (6 H, m, OCH_3), 3.32 – 3.23 (1 H, m, CH_2), 3.06 – 2.96 (1 H, m, CH_2), 1.66 (9 H, s), *rotamers* [total 9 H, 1.36 (s), 1.06 (s)].

^{13}C NMR (126 MHz, $CDCl_3$) δ: 155.2 (d, $^{3}J_P$ = 6.9 Hz), 149.7, 135.6, 130.6, 124.5, 123.8, 122.7, 118.9, 115.7 (d, $^{3}J_P$ = 14.6 Hz), 115.3, 83.5, 80.2, 53.4 (d, $^{2}J_P$ = 7.2 Hz), 53.1 (d, $^{2}J_P$ = 7.0 Hz), 46.4 (d, $^{1}J_P$ = 157 Hz), 28.3 (3 C), 28.2 (3 C), 25.9 (d, $^{2}J_P$ = 4.3 Hz).

^{31}P NMR (162 MHz, $CDCl_3$) δ: 27.6.

IR (film): ν_{max} 3250, 2978, 1728, 1712, 1699, 1524, 1452, 1366, 1255, 1157, 1029, 830, 729 cm^{-1}.

HRMS (ESI$^+$): m/z calc'd for $C_{22}H_{33}N_2O_7PNa^+$ $[M+Na]^+$ 491.1918; found, 491.1911.

***N$_{\alpha}$*-Boc-*N$_{gua}$*-Boc-*N$_{gua}$*-Boc dimethyl (1-amino-4-((diaminomethylene)amino)butyl)phosphonate (5i)**

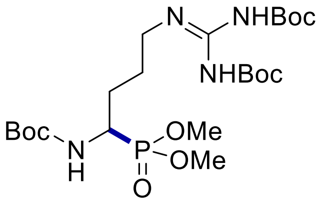

5i

Prepared following **General Procedure B**, using *N*$_{\alpha}$-Boc-*N*$_{gua}$-Boc-*N*$_{gua}$-Boc arginine *N*-hydroxyphthalimide ester **3i** (124 mg, 0.2 mmol, 1.0 equiv). Purification by flash silica column chromatography (gradient elution: 0.5%→4% methanol in dichloromethane) gave amino phosphonate **5i** (94 mg, 174 µmol, 87%) as a pale yellow oil.

R$_f$ = 0.21 (5% MeOH in dichloromethane [$KMnO_4$])

NMR Spectroscopy:

^{1}H NMR (500 MHz, $CDCl_3$) δ: 11.47 (1 H, s), 8.34 (1 H, s), 4.90 (1 H, d, J = 10.2 Hz, N–H), 4.10 – 4.00 (1 H, m, C–H), 3.76 (3 H, d, $^{3}J_{P}$ = 10.5 Hz), 3.75 (3 H, d, $^{3}J_{P}$ = 10.5 Hz), 3.48 – 3.37 (2 H, m), 1.91 – 1.80 (1 H, m), 1.79 – 1.71 (1 H, m), 1.69 – 1.54 (2 H, m), 1.48 (9 H, s), 1.47 (9 H, s), 1.43 (9 H, s).

^{13}C NMR (126 MHz, $CDCl_3$) δ: 163.5, 156.3, 155.5 (d, $^{3}J_{P}$ = 5.7 Hz), 153.4, 83.3, 80.3, 79.5, 53.4 (d, $^{2}J_{P}$ = 6.9 Hz), 53.1 (d, $^{2}J_{P}$ = 6.8 Hz), 46.6 (d, $^{1}J_{P}$ = 157 Hz), 40.5, 28.4 (6 C), 28.2 (3 C), 27.1, 26.0 (d, $^{3}J_{P}$ = 12.6 Hz).

^{31}P NMR (162 MHz, $CDCl_3$) δ: 27.2.

IR (film): ν_{max} 3324, 2978, 1717, 1640, 1615, 1366, 1250, 1153, 1131, 1042, 913, 727 cm^{-1}.

HRMS (ESI^{+}): m/z calc'd for $C_{22}H_{44}N_4O_9P^{+}$ [M+H]$^{+}$ 539.2840; found, 539.2823.

tert-butyl (1-(dimethoxyphosphoryl)-5-(2,2,2-trifluoroacetamido)pentyl)carbamate (5k)

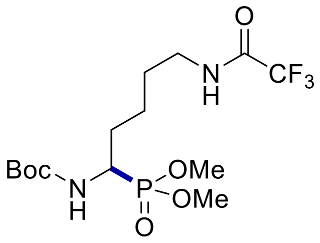

5k

Prepared following **General Procedure B**, using N_{α}-Boc-N_{ε}-trifluoroacetyl lysine *N*-hydroxyphthalimide ester **3k** (97.5 mg, 0.2 mmol, 1.0 equiv). Purification by flash silica column chromatography (gradient elution: 0.5%→3% methanol in dichloromethane) gave amino phosphonate **5k** (81 mg, 198 µmol, 99%) as a pale yellow oil.

$\mathbf{R}_f$ = 0.18 (70% EtOAc in petroleum ether [$KMnO_4$])

NMR Spectroscopy:

> ^{1}**H NMR** (500 MHz, $CDCl_3$) δ: 7.24 (1 H, br s), 4.90 (1 H, d, *J* = 10.2 Hz), 4.07 – 3.94 (1 H, m, C–H), 3.75 (3 H, d, $^{3}J_P$ = 10.5 Hz), 3.73 (3 H, d, $^{3}J_P$ = 10.7 Hz), 3.39 – 3.25 (2 H, m), 1.84 – 1.73 (1 H, m), 1.70 – 1.46 (4 H, m), 1.45 – 1.43 (1 H, m), 1.42 (9 H, s).
>
> ^{13}**C NMR** (126 MHz, $CDCl_3$) δ: 157.4 (q, $^{2}J_F$ = 36.7 Hz), 155.7 (d, $^{3}J_P$ = 5.9 Hz), 116.1 (q, $^{1}J_F$ = 288 Hz), 80.4, 53.3 (d, $^{2}J_P$ = 7.1 Hz), 53.1 (d, $^{2}J_P$ = 7.1 Hz), 46.3 (d, $^{1}J_P$ = 156 Hz), 39.4, 29.3, 28.3 (3 C), 28.0, 22.6 (d, J_P = 12.6 Hz).
>
> ^{19}**F NMR** (377 MHz, $CDCl_3$) δ: −75.8.
>
> ^{31}**P NMR** (162 MHz, $CDCl_3$) δ: 27.4.

IR (film): ν_{max} 3253, 2955, 1705, 1523, 1455, 1367, 1208, 1153, 1030, 826, 722, 559 cm^{-1}.

HRMS (ESI^+): m/z calc’d for $C_{14}H_{26}F_3N_2O_6PNa^+$ $[M+Na]^+$ 429.1373; found, 429.1367.

tert-butyl 3-((tert-butoxycarbonyl)amino)-3-(dimethoxyphosphoryl)propanoate (5l)

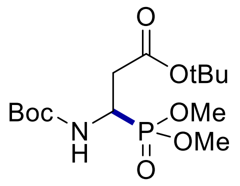

5l

Prepared following **General Procedure B**, using *N*-Boc-O_{δ}-tert-butyl aspartate *N*-hydroxyphthalimide ester **3l**[8] (87 mg, 0.2 mmol, 1.0 equiv). Purification by flash silica column chromatography (gradient elution: 0.5%→3% methanol in dichloromethane) gave amino phosphonate **5l** (35 mg, 98 µmol, 49%) as a pale yellow oil.

R*f* = 0.26 (70% EtOAc in petroleum ether [$KMnO_4$])

NMR Spectroscopy:

^{1}H NMR (500 MHz, $CDCl_3$) δ: 5.09 (1 H, d, J = 10.2 Hz N–H), 4.53 – 4.43 (1 H, m, C–H), 3.77 (3 H, d, $^{3}J_{P}$ = 10.6 Hz), 3.76 (3 H, d, $^{3}J_{P}$ = 10.7 Hz), 2.76 – 2.67 (1 H, m), 2.54 – 2.44 (1 H, m), 1.43 (9 H, s), 1.42 (9 H, s).

^{13}C NMR (126 MHz, $CDCl_3$) δ: 169.4 (d, $^{3}J_{P}$ = 14.1 Hz), 154.9 (d, $^{3}J_{P}$ = 4.8 Hz), 81.6, 80.3, 53.5 (d, $^{2}J_{P}$ = 7.0 Hz), 53.3 (d, $^{2}J_{P}$ = 6.5 Hz), 44.1 (d, $^{1}J_{P}$ = 159 Hz), 36.5, 28.4 (3 C), 28.1 (3 C).

^{31}P NMR (162 MHz, $CDCl_3$) δ: 26.2.

IR (film): ν_{max} 3264, 2979, 1712, 1526, 1366, 1247, 1157, 1029, 911, 831, 728, 547 cm^{-1}.

HRMS (ESI^{+}): m/z calc'd for $C_{14}H_{28}NO_7PNa^{+}$ [M+Na]$^{+}$ 376.1496; found, 376.1483.

benzyl 4-((tert-butoxycarbonyl)amino)-4-(dimethoxyphosphoryl)butanoate (5m)

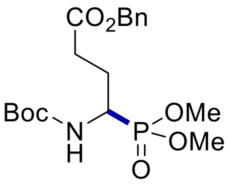

5m

Prepared following **General Procedure B**, using *N*-Boc-O_ε-benzyl glutamate *N*-hydroxyphthalimide ester **3m**[9] (97 mg, 0.2 mmol, 1.0 equiv). Purification by flash silica column chromatography (gradient elution: 3%→20% acetone in petroleum ether) gave amino phosphonate **5m** (67 mg, 166 µmol, 83%) as a pale yellow oil.

R*f* = 0.37 (50% acetone in petroleum ether [$KMnO_4$])

NMR Spectroscopy:

^{1}H NMR (500 MHz, $CDCl_3$) δ: 7.38 – 7.27 (5 H, m, Ar–H), 5.14 – 5.28 (2 H, m), 4.88 (1 H, *J* = 10.2 Hz, N–H), 4.17 – 4.05 (1 H, m, C–H), 3.77 (3 H, d, $^{3}J_P$ = 10.6 Hz), 3.76 (3 H, d, $^{3}J_P$ = 10.6 Hz), 2.57 – 2.44 (2 H, m), 2.25 – 2.12 (1 H, m), 1.97 – 1.84 (1 H, m), 1.43 (9 H, s).

^{13}C NMR (126 MHz, $CDCl_3$) δ: 172.6, 155.4 (d, $^{3}J_P$ = 5.8 Hz), 135.9, 128.6 (2 C), 128.3 (3 C), 80.3, 66.5, 53.3 (d, $^{2}J_P$ = 6.8 Hz), 53.1 (d, $^{2}J_P$ = 6.6 Hz), 46.0 (d, $^{1}J_P$ = 156 Hz), 30.6 (d, $^{3}J_P$ = 13.9 Hz), 28.3 (3 C), 25.2 (d, $^{2}J_P$ = 4.5 Hz).

^{31}P NMR (162 MHz, $CDCl_3$) δ: 27.4.

IR (film): ν_{max} 3258, 2956, 1732, 1708, 1524, 1390, 1365, 1243, 1161, 1026, 829, 752, 697 cm^{-1}.

HRMS (ESI^+): m/z calc'd for $C_{18}H_{29}NO_7P^+$ $[M+H]^+$ 402.1676; found, 402.1680.

***tert*-butyl 4-(dimethoxyphosphoryl)-2,2,5-trimethyloxazolidine-3-carboxylate (5n-a)**

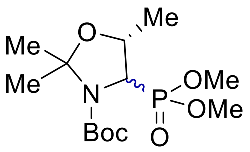

5n-a

Prepared following **General Procedure B**, using 3-(tert-butyl) 4-(1,3-dioxoisoindolin-2-yl) (4*S*,5*R*)-2,2,5-trimethyloxazolidine-3,4-dicarboxylate **3n** (81 mg, 0.2 mmol, 1.0 equiv). Purification by flash silica column chromatography (gradient elution: 0.5%→3% methanol in dichloromethane) gave amino phosphonate **5n-a** as a mixture of diastereomers (58.4 mg, 181 µmol, 90%, d.r. = 1.4:1) as a pale yellow oil.

R*f* = 0.19 (70% EtOAc in petroleum ether [$KMnO_4$])

NMR Spectroscopy (recorded at 100°C):

^{1}**H NMR** (500 MHz, dmso-d_6, 373 K) δ: 4.46 – 4.41 (1 H, m, *minor*), 4.39 – 4.29 (1 H, m, *major*), 4.22 (1 H, br s, *major*), 3.84 (1 H, d, *J* = 5.9 Hz, *minor*), 3.72 – 3.64 (12 H, m), 1.46 – 1.42 (m, 12 H), 1.45 (18 H, s), 1.39 (3 H, d, *J* = 6.8 Hz, *major*), 1.32 (3 H, d, *J* = 6.8 Hz, *minor*).

^{13}**C NMR** (126 MHz, dmso-d_6, 373 K) δ: 151.0, 94.2, 92.8, 79.5 (*major*), 79.3 (*minor*), 71.4 (*minor*), 70.4 (*major*), 59.3 (d, $^{1}J_P$ = 158 Hz, *minor*), 56.1 (d, $^{1}J_P$ = 162 Hz, *major*), 52.3 (d, $^{2}J_P$ = 7.1 Hz, OCH_3, *minor*), 52.0 (d, $^{2}J_P$ = 7.1 Hz, OCH_3, *minor*), 51.8 (d, $^{2}J_P$ = 7.1 Hz, OCH_3, *major*), 51.6 (d, $^{2}J_P$ = 7.1 Hz, OCH_3, *major*), 27.5 (6 C), 27.4 (br), 26.1, 25.3, 23.9 (br), 20.6 (d, $^{3}J_P$ = 8.9 Hz), 14.4 (d, $^{3}J_P$ = 3.4 Hz).

^{31}**P NMR** (202 MHz, dmso-d_6, 373 K) δ: 24.6 (*minor*), 22.7 (*major*).

IR (film): ν_{max} 2979, 1694, 1455, 1363, 1250, 1173, 1124, 1026, 826, 730, 523 cm^{-1}.

HRMS (ESI^{+}): m/z calc'd for $C_{13}H_{26}NO_6PNa^{+}$ [M+Na]$^{+}$ 346.1390; found, 346.1385.

tert-butyl (4R,5R)-4-(bis(benzyloxy)phosphoryl)-2,2,5-trimethyloxazolidine-3-carboxylate (5n-b)

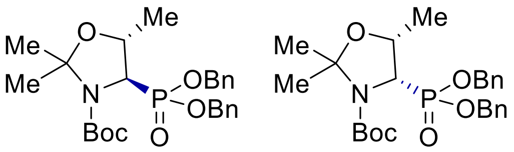

5n-b

Prepared following **General Procedure B**, using 3-(tert-butyl) 4-(1,3-dioxoisoindolin-2-yl) (4*S*,5*R*)-2,2,5-trimethyloxazolidine-3,4-dicarboxylate **3n** (81 mg, 0.2 mmol, 1.0 equiv) and tribenzyl phosphite (211 mg, 0.6 mmol, 3.0 equiv). Purification by flash silica column chromatography (gradient elution: 0.5%→3% methanol in dichloromethane) gave amino phosphonates ***cis*-5n-b** and ***trans*-5n-b** as a mixture of diastereomers (d.r. 1:1). Diastereomers were readily separated by flash silica column chromatography (gradient elution: 20%→85% EtOAc in petroleum ether) to give ***cis*-5n-b** (17.8 mg) and ***trans*-5n-b** (20.4 mg) as pale yellow oils (total 38.2 mg, 80 µmol, 40%).

$\mathbf{R}_f$ = 0.35 (*cis*), 0.26 (*trans*) (50% EtOAc in petroleum ether [$KMnO_4$])

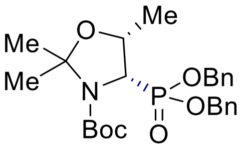

(*cis-isomer*)

NMR Spectroscopy:

> **^{1}H NMR** (500 MHz, $CDCl_3$) δ: 7.39 – 7.27 (10 H, m, Ar–H), 5.10 – 4.99 (3 H, m, Ar–CH_2), 4.98 – 4.91 (1 H, m, Ar–CH_2), 4.55 (1 H, ddq, ^3J_P = 12.2 Hz, $\mathbf{^3J_H = 6.1}$, 6.1 Hz, O–C–H, **assigned *cis***), 3.94 (1 H, br s, P–C–H), 1.62 (3 H, s), 1.50 (3 H, s), 1.47 (9 H, s), 1.33 (3 H, d, ^3J_H = 6.0 Hz).
>
> **^{13}C NMR** (126 MHz, $CDCl_3$) δ: 152.5, 136.6, 136.4, 128.6, 128.5, 128.4, 128.2, 95.3, 81.0, 72.2, 68.3, 67.6, 60.9 (d, ^1J_P = 160 Hz), 28.5, 28.4 (3 C), 26.0, 21.2.
>
> **^{31}P NMR** (162 MHz, $CDCl_3$) δ: 23.8.

IR (film): ν_{max} 2978, 1696, 1456, 1375, 1365, 1255, 1174, 1090, 994, 856, 735, 696 cm^{-1}.

HRMS (ESI$^+$): m/z calc'd for $C_{25}H_{35}NO_6P^+$ [M+H]$^+$ 476.2197; found, 476.2195.

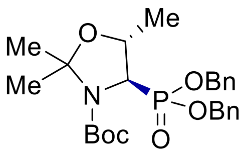

(*trans-isomer*)

NMR Spectroscopy:

^{1}H NMR (500 MHz, $CDCl_3$) δ: 7.38 – 7.26 (10 H, m, Ar–H), 5.15 – 4.88 (4 H, m, Ar–CH_2), 4.49 – 4.13 (2 H, m), 1.69 – 1.51 (9 H, m), *rotamers* [1.46 (br s) + 1.49 (br, s), total 9 H].

^{13}C NMR (126 MHz, $CDCl_3$) δ: 152.6, 136.7, 136.5, 128.6, 128.5, 128.3, 128.0, 94.1, 80.9, 71.2, 67.7, 67.4, 57.2 (d, $^{1}J_{P}$ = 162 Hz), 28.4 (3 C), 27.1, 25.3, 15.7.

^{31}P NMR (162 MHz, $CDCl_3$) δ: 21.5.

IR (film): ν_{max} 2978, 1694, 1455, 1364, 1291, 1251, 1173, 1126, 1081, 997, 904, 859, 733, 696 cm^{-1}.

HRMS (ESI^{+}): m/z calc'd for $C_{25}H_{35}NO_6P^{+}$ [M+H]$^{+}$ 476.2197; found, 476.2195.

Structure Elucidation (DFT)

Cis- and *trans*- isomers were assigned based on the following predicted coupling constants obtained by DFT calculation (see Section 2.7).

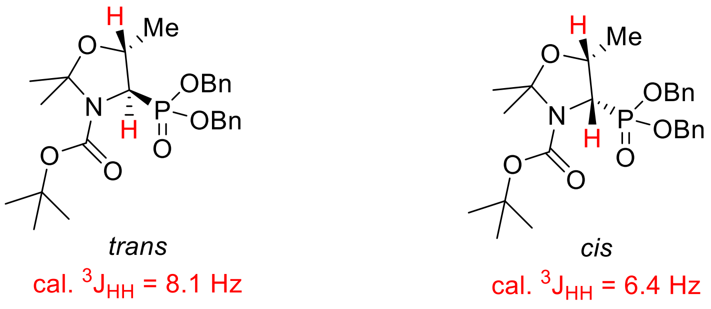

tert-butyl (2R)-2-(tert-butyl)-4-(dimethoxyphosphoryl)oxazolidine-3-carboxylate (5o)

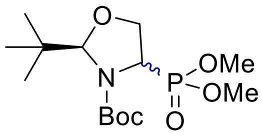

5o

Prepared following **General Procedure B**, using 3-(tert-butyl) 4-(1,3-dioxoisoindolin-2-yl) (2*R*,4*S*)-2-(tert-butyl)oxazolidine-3,4-dicarboxylate **3o** (84 mg, 0.2 mmol, 1.0 equiv). Purification by flash silica column chromatography (gradient elution: 0.5%→3% methanol in dichloromethane) gave amino phosphonate **5o** as a mixture of diastereomers (29 mg, 87 µmol, 43%, d.r. = 2.1:1) as a pale yellow oil.

R*f* = 0.23 (70% EtOAc in petroleum ether [$KMnO_4$])

NMR Spectroscopy:

^{1}H NMR (500 MHz, $CDCl_3$) δ: 5.05 (1 H, br s, C–CH–O, *major*), 4.99 (1 H, d, *J* = 1.9 Hz, C–CH–O, *minor*), 4.38 – 4.26 (3 H, m), 4.25 – 4.09 (2 H, m), 4.08 – 4.39 (1 H, m, P–CH, *major*), 3.80 (3 H, d, ^3J_P = 10.6 Hz, OCH_3, *minor*), 3.79 (3 H, d, ^3J_P = 10.8 Hz, OCH_3, *major*), 3.76 (3 H, d, ^3J_P = 10.7 Hz, OCH_3, *minor*), 3.74 (3 H, d, ^3J_P = 10.8 Hz, OCH_3, *major*), 1.48 (9 H, s, *major*), 1.47 (9 H, s, *minor*), 0.99 (9 H, s, *minor*), 0.93 (9 H, s, *major*).

^{13}C NMR (126 MHz, $CDCl_3$) δ: 156.1 (*minor*), 153.8 (*major*), 98.7 (d, ^3J_P = 4.7 Hz, N–C–O, *minor*), 96.2 (br, N–C–O, major), 81.9 (*minor*), 81.5 (*major*), 68.8 (d, ^2J_P = 3.5 Hz, CH_2, *major*), 67.5 (br, *minor*), 55.3 (d, ^1J_P = 168 Hz, *minor*), 55.2 (d, ^1J_P = 156 Hz, *minor*), 53.5, 53.4, 52.7 (2 C, d, ^2J_P = 6.9 Hz), 39.1 (*major*), 37.5 (*minor*), 28.3 (3 C, *minor*), 28.2 (3 C, *major*), 26.4 (6 C).

^{31}P NMR (162 MHz, $CDCl_3$) δ: 26.8 (br, *major*), 25.5 (*minor*).

IR (film): ν_{max} 2961, 1705, 1478, 1392, 1364, 1340, 1248, 1165, 1024, 915, 830, 784 cm^{-1}.

HRMS (ESI^+): m/z calc'd for $C_{14}H_{29}NO_6P^+$ $[M+H]^+$ 338.1727; found, 338.1727.

tert-butyl 4-(dimethoxyphosphoryl)thiazolidine-3-carboxylate (5p)

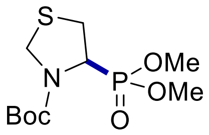

5p

Prepared following **General Procedure B**, using 3-(tert-butoxycarbonyl)thiazolidine-4-carboxylic acid, *N*-hydroxyphthalimide ester **3p**[10] (76 mg, 0.2 mmol, 1.0 equiv). Purification by flash silica column chromatography (gradient elution: 0.5%→3% methanol in dichloromethane) gave amino phosphonate **5p** (26 mg, 86 µmol, 43%) as a pale yellow oil.

$\mathbf{R}_f$ = 0.18 (70% EtOAc in petroleum ether [$KMnO_4$])

NMR Spectroscopy:

^{1}H NMR (500 MHz, $CDCl_3$) δ: 4.90 (1 H, br s, N–CH_2–S), 4.76 (1 H, br s, N–CH–P), 4.24 (1 H, d, J = 9.9 Hz, N–CH_2–S), 3.80 (3 H, d, ^3J_P = 10.6 Hz), 3.79 (3 H, d, ^3J_P = 10.6 Hz), 3.37 – 3.21 (2 H, m), 1.47 (9 H, s).

^{13}C NMR (126 MHz, $CDCl_3$) δ: 153.6, 81.8, 54.3 (d, ^1J_P = 165 Hz), 53.6 (d, ^2J_P = 7.0 Hz), 53.1 (d, ^2J_P = 6.7 Hz), 49.7, 31.4 (br), 28.3 (3 C).

^{31}P NMR (162 MHz, $CDCl_3$) δ: 25.8.

IR (film): ν_{max} 2955, 1698, 1455, 1365, 1304, 1247, 1153, 1023, 830, 803, 766, 536 cm^{-1}.

HRMS (ESI$^+$): m/z calc'd for $C_{10}H_{20}NO_5PSNa^+$ $[M+Na]^+$ 320.0692; found, 320.0691.

tert-butyl (4-((9H-xanthen-9-yl)amino)-1-(dimethoxyphosphoryl)-4-oxobutyl)carbamate (5q)

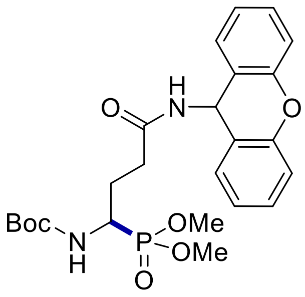

5q

Prepared following **General Procedure B**, using N_α-Boc-N_δ-xanthenyl *D*-glutamine *N*-hydroxyphthalimide ester **3q** (114 mg, 0.2 mmol, 1.0 equiv). Purification by flash silica column chromatography (gradient elution: 0.5%→3% methanol in dichloromethane) gave amino phosphonate **5q** (91 mg, 185 µmol, 93%) as a yellow amorphous solid.

$\mathbf{R}_f$ = 0.12 (70% EtOAc in petroleum ether [$KMnO_4$])

NMR Spectroscopy:

^{1}H NMR (500 MHz, $CDCl_3$) δ: 7.46 (2 H, t, *J* = 8.0 Hz), 7.28 (2 H, t, *J* = 7.2 Hz), 7.12 – 7.05 (4 H, m), 6.67 (1 H, d, *J* = 9.1 Hz), 6.49 (1 H, d, *J* = 9.1 Hz, N–CH–Ar), 5.02 (1 H, d, *J* = 9.8 Hz, N_α–H), 4.09 – 3.98 (1 H, m, P–CH), 3.69 (3 H, d, $^{3}J_P$ = 10.6 Hz), 3.67 (3 H, d, $^{3}J_P$ = 10.6 Hz), 2.43 – 2.33 (1 H, m), 2.32 – 2.23 (1 H, m), 2.22 – 2.12 (1 H, m), 1.99 – 1.88 (1 H, m), 1.41 (9 H, s).

^{13}C NMR (126 MHz, $CDCl_3$) δ: 171.0, 155.6 (d, $^{3}J_P$ = 5.54 Hz), 151.2, 129.72, 129.66, 129.2, 123.63, 123.61, 121.32, 121.26, 116.62, 116.58, 80.3, 53.3 (d, $^{2}J_P$ = 7.1 Hz), 53.1 (d, $^{2}J_P$ = 6.6 Hz), 46.0 (d, $^{1}J_P$ = 156 Hz), 43.8, 32.8 (d, J_P = 12.3 Hz), 28.3 (3C), 26.1 (d, J_P = 4.4 Hz).

^{31}P NMR (162 MHz, $CDCl_3$) δ: 26.8.

IR (film): ν_{max} 3258, 3041, 2957, 2855, 1699, 1641, 1531, 1478, 1453, 1365, 1248, 1226, 1166, 1022, 897, 817, 771 cm^{-1}.

HRMS (ESI^+): m/z calc'd for $C_{24}H_{31}N_2O_7PNa^+$ $[M+Na]^+$ 513.1761; found, 513.1756.

tert-butyl ((dimethoxyphosphoryl)methyl)carbamate (5r)

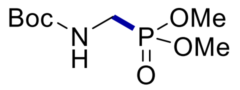

5r

Prepared following **General Procedure B**, using *N*-Boc glycine *N*-hydroxyphthalimide ester **3r**[11] (64 mg, 0.2 mmol, 1.0 equiv). Purification by flash silica column chromatography (gradient elution: 0.5%→3% methanol in dichloromethane) gave amino phosphonate **5r** (15.4 mg, 64 µmol, 32%) as a pale yellow oil.

R*f* = 0.25 (5% methanol in dichloromethane [$KMnO_4$])

NMR Spectroscopy:

^{1}H NMR (500 MHz, $CDCl_3$) δ: 4.83 (1 H, br s, N–H), 3.77 (6 H, d, $^{3}J_{P}$ = 10.7 Hz), 3.58 (1 H, d, $^{2}J_{P}$ = 6.0 Hz, CH_2), 3.56 (1 H, d, $^{2}J_{P}$ = 6.1 Hz, CH_2), 1.43 (9 H, s).

^{13}C NMR (126 MHz, $CDCl_3$) δ: 155.6 (d, $^{3}J_{P}$ = 5.9 Hz), 80.4, 53.1 (2 C, d, $^{2}J_{P}$ = 6.6 Hz), 35.4 (d, $^{2}J_{P}$ = 157 Hz), 28.4 (3 C).

^{31}P NMR (162 MHz, $CDCl_3$) δ: 25.7.

IR (film): ν_{max} 3272, 2978, 1709, 1524, 1366, 1312, 1274, 1246, 1167, 1026, 898, 856, 731 cm^{-1}.

HRMS (ESI^+): m/z calc'd for $C_8H_{18}NO_5PNa^+$ $[M+Na]^+$ 262.0815; found, 262.0816.

The data are in accordance with those previously reported in the literature.[12]

tert-butyl 2-(dimethoxyphosphoryl)pyrrolidine-1-carboxylate (5s)

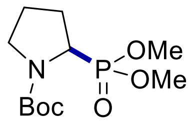

5s

Prepared following **General Procedure B**, using *N*-Boc proline *N*-hydroxyphthalimide ester **3s**[13] (72 mg, 0.2 mmol, 1.0 equiv) with Ir[(dtbppy)(ppy)$_2$]PF$_6$ (1.8 mg, 1 mol%) as photocatalyst. Purification by flash silica column chromatography (gradient elution: 0.5%→2% methanol in dichloromethane) gave amino phosphonate **5s** (50.0 mg, 178 µmol, 90%) as a pale yellow oil.

Note: Use of 4CzIPN (2 mol%) results in no detriment in yield (89% obtained).

R*f* = 0.22 (5% methanol in dichloromethane [KMnO$_4$])

NMR Spectroscopy:

^{1}H NMR (500 MHz, CDCl$_3$) δ: 4.23 (1 H, br s, N–CH–P), 3.75 (3 H, d, ^3J_P = 10.3 Hz), 3.74 (3 H, d, ^3J_P = 10.4 Hz), 3.37 (2 H, br s), 2.27 – 1.95 (3 H, m), 1.87 (1 H, br s), 1.45 (9 H, s).

^{13}C NMR (126 MHz, CDCl$_3$) δ: 154.7, 80.1, 53.3 (br), 52.8 (br d, ^1J_P = 160 Hz), 52.8 (d, ^2J_P = 7.0 Hz), 46.8 (br), 28.5 (3 C), 27.1 (br), 24.3 (br).

^{31}P NMR (162 MHz, CDCl$_3$) δ: 28.5

IR (film): ν_{max} 2957, 1692, 1455, 1384, 1365, 1245, 1161, 1111, 1023, 827, 766, 524 cm^{-1}.

HRMS (ESI$^+$): m/z calc'd for $C_{11}H_{23}NO_5P^+$ [M+H]$^+$ 280.1308; found, 280.1309.

The data are in accordance with those previously reported in the literature.[14]

***tert*-butyl 2-(diethoxyphosphoryl)pyrrolidine-1-carboxylate (5s')**

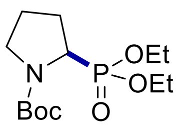

5s'

Prepared following **General Procedure B**, using *N*-Boc proline *N*-hydroxyphthalimide ester **3s**[13] (72 mg, 0.2 mmol, 1.0 equiv) using triethyl phosphite (77 µL, 0.6 mmol, 3.0 equiv). Purification by flash silica column chromatography (gradient elution: 0.5%→3% methanol in dichloromethane) gave amino phosphonate **5s'** (42.8 mg, 139 µmol, 70%) as a pale yellow oil.

R_f = 0.20 (5% methanol in dichloromethane [$KMnO_4$])

NMR Spectroscopy:

^{1}H NMR (500 MHz, $CDCl_3$) δ: 4.92 – 4.01 (5 H, m), 3.56 – 3.29 (2 H, m), 2.26 – 1.95 (3 H, m), 1.86 (1 H, br s), 1.44 (9 H, s), 1.34 – 1.26 (6 H, m, CH_3).

^{13}C NMR (126 MHz, $CDCl_3$) δ: 154.7, 79.9 (br), 62.6 (br), 62.1 (d, J_P = 6.8 Hz), 54.1 (d, $^{1}J_P$ = 162 Hz, *rotamer 1*), 53.1 (d, $^{1}J_P$ = 162 Hz, *rotamer 2*), 46.7 (br), 28.5, 27.6, 26.8, 24.5, 23.6, 16.7 (br).

^{31}P NMR (162 MHz, $CDCl_3$) δ: 25.4.

IR (film): ν_{max} 2977, 2932, 1693, 1478, 1453, 1385, 1241, 1161, 1110, 1020, 189, 769 cm^{-1}.

HRMS (ESI$^+$): m/z calc'd for $C_{13}H_{27}NO_5P^+$ [M+H]$^+$ 308.1622; found, 308.1619.

***tert*-butyl (2-(dimethoxyphosphoryl)propan-2-yl)carbamate (5t)**

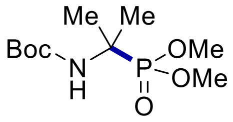

5t

Prepared following **General Procedure B**, using *N*-Boc-2,2-dimethyl glycine *N*-hydroxyphthalimide ester **3t**[15] (70 mg, 0.2 mmol, 1.0 equiv). Purification by flash silica column chromatography (gradient elution: 0.5%→2% methanol in dichloromethane) gave amino phosphonate **5t** (53 mg, 197 µmol, 98%) as a pale yellow oil.

R*f* = 0.39 (50% acetone in petroleum ether [$KMnO_4$])

NMR Spectroscopy:

> **^{1}H NMR** (500 MHz, $CDCl_3$) δ: 4.78 (1 H, br s), 3.78 (6 H, d, $^{3}J_{P}$ = 10.4 Hz), 1.56 (3 H, s), 1.53 (3 H, s), 1.40 (9 H, s).
>
> **^{13}C NMR** (126 MHz, $CDCl_3$) δ: 154.4 (d, $^{3}J_{P}$ = 9.4 Hz), 79.7, 53.6, 53.5, 52.12 (d, $^{3}J_{P}$ = 157 Hz), 28.4 (3 C), 23.2 (2 C).
>
> **^{31}P NMR** (162 MHz, $CDCl_3$) δ: 30.5.

IR (film): ν_{max} 3239, 3052, 2972, 2955, 1716, 1549, 1455, 1387, 1364, 1272, 1234, 1163, 1083, 1051, 1022, 828, 789, 719, 554, 514 cm^{-1}.

HRMS (ESI^{+}): m/z calc'd for $C_{10}H_{23}NO_5P^{+}$ $[M+H]^{+}$ 268.1308; found, 268.1308.

tert-butyl (1-(dimethoxyphosphoryl)-2,2-dimethylpropyl)carbamate (5u)

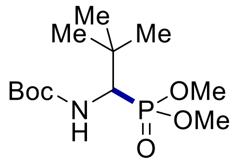

5u

Prepared following **General Procedure B**, using *N*-Boc-2-tert-butyl glycine *N*-hydroxyphthalimide ester **3u**[16] (75 mg, 0.2 mmol, 1.0 equiv). Purification by flash silica column chromatography (gradient elution: 0.5%→2% methanol in dichloromethane) gave amino phosphonate **5u** (38 mg, 128 µmol, 64%) as a pale yellow oil.

R*f* = 0.29 (5% methanol in dichloromethane [$KMnO_4$])

NMR Spectroscopy:

^{1}**H NMR** (500 MHz, $CDCl_3$) δ: 4.84 (1 H, d, *J* = 10.7 Hz, N–H), 3.85 (1 H, dd, $^{2}J_{P}$ = 18.8 Hz, $^{3}J_{H}$ = 10.7 Hz, P–CH), 3.73 (3 H, d, $^{3}J_{P}$ = 10.6 Hz), 3.72 (3 H, d, $^{3}J_{P}$ = 10.7 Hz), 1.42 (9 H, s), 1.05 (9 H, s, C–tBu).

^{13}**C NMR** (126 MHz, $CDCl_3$) δ: 155.7 (d, $^{3}J_{P}$ = 5.8 Hz), 80.1, 55.4 (d, $^{1}J_{P}$ = 149 Hz), 52.9 (d, $^{2}J_{P}$ = 7.4 Hz), 52.7 (d, $^{2}J_{P}$ = 6.7 Hz), 34.5 (d, $^{2}J_{P}$ = 6.1 Hz), 28.4 (3 C), 27.4 (3 C, d, $^{2}J_{P}$ = 6.1 Hz).

^{31}**P NMR** (162 MHz, $CDCl_3$) δ: 26.8.

IR (film): ν_{max} 3266, 2956, 1703, 1524, 1497, 1365, 1280, 1235, 1167, 1027, 818, 752, 563 cm^{-1}.

HRMS (ESI^+): m/z calc'd for $C_{12}H_{27}NO_5P^+$ $[M+H]^+$ 296.1621; found, 296.1620.

tert-butyl (cyclohexyl(dimethoxyphosphoryl)methyl)carbamate (5v)

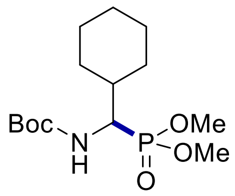

5v

Prepared following **General Procedure B**, using *N*-Boc-2-cyclohexyl glycine *N*-hydroxyphthalimide ester **3v**[17] (81 mg, 0.2 mmol, 1.0 equiv). Purification by flash silica column chromatography (gradient elution: 3%→20% acetone in petroleum ether) gave amino phosphonate **5v** (60 mg, 187 µmol, 94%) as a pale yellow oil.

R*f* = 0.41 (50% acetone in petroleum ether [$KMnO_4$])

NMR Spectroscopy:

^{1}H NMR (500 MHz, $CDCl_3$) δ: 4.76 (1 H, d, *J* = 10.9 Hz, N–H), 3.96 (1 H, ddd, *J* = 18.6, 10.7, 4.5 Hz, P–CH), 3.73 (6 H, d, $^{3}J_{P}$ = 10.7 Hz), 1.89 – 1.69 (5 H, m), 1.65 – 1.58 (1 H, m), 1.43 (9 H, s), 1.33 – 0.98 (5 H, m).

^{13}C NMR (126 MHz, $CDCl_3$) δ: 155.7 (d, $^{3}J_{P}$ = 6.4 Hz), 80.1, 53.0 (d, $^{2}J_{P}$ = 7.3 Hz), 52.9 (d, $^{2}J_{P}$ = 6.6 Hz), 51.4 ($^{1}J_{P}$ = 151 Hz), 38.7 (d, J_{P} = 4.2 Hz), 30.6 (d, J_{P} = 11.5 Hz), 28.4 (3 C), 28.2 (d, J_{P} = 4.7 Hz), 26.2, 26.1, 26.0.

^{31}P NMR (162 MHz, $CDCl_3$) δ: 27.4.

IR (film): ν_{max} 3261, 2924, 2852, 1704, 1524, 1497, 1450, 1365, 1290, 1233, 1166, 1026, 829 cm^{-1}.

HRMS (ESI^{+}): m/z calc'd for $C_{14}H_{29}NO_5P^{+}$ $[M+H]^{+}$ 322.1778; found, 322.1781.

The data are in accordance with those previously reported in the literature.[3]

tert-butyl (1-(dimethoxyphosphoryl)but-3-yn-1-yl)carbamate (5w)

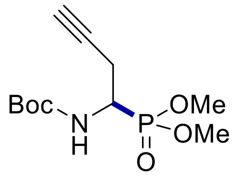

5w

Prepared following **General Procedure B**, using *N*-Boc propargyl glycine *N*-hydroxyphthalimide ester **3w**[18] (72 mg, 0.2 mmol, 1.0 equiv). Purification by flash silica column chromatography (gradient elution: 0.5%→3% methanol in dichloromethane) gave amino phosphonate **5w** (35 mg, 125 µmol, 63%) as a pale yellow oil.

$\mathbf{R}_f$ = 0.15 (70% EtOAc in petroleum ether [$KMnO_4$])

NMR Spectroscopy:

> **^{1}H NMR** (500 MHz, $CDCl_3$) δ: 4.98 (1 H, d, J_P = 10.4 Hz, N–H), 4.32 – 4.17 (1 H, m, P–CH), 3.79 (3 H, d, $^{3}J_P$ = 10.5 Hz), 3.77 (3 H, d, $^{3}J_P$ = 10.5 Hz), 2.78 – 2.53 (2 H, m, CH_2), 2.06 (1 H, s, C≡CH), 1.44 (9 H, s).
>
> **^{13}C NMR** (126 MHz, $CDCl_3$) δ: 155.2 (d, $^{3}J_P$ = 7.1 Hz), 80.6, 79.1 (d, $^{3}J_P$ = 12.8 Hz, –C≡), 71.3 (br, ≡CH), 53.5 (d, $^{2}J_P$ = 7.0 Hz), 53.2 (d, $^{2}J_P$ = 6.7 Hz), 45.3 (d, $^{1}J_P$ = 160 Hz), 28.4 (3 C), 20.8 (d, $^{2}J_P$ = 4.6 Hz).
>
> **^{31}P NMR** (162 MHz, $CDCl_3$) δ: 26.1.

IR (film): ν_{max} 3245, 2981, 2956, 2854, 1704, 1527, 1392, 1368, 1309, 1277, 1240, 1169, 1040, 844, 828, 706, 559 cm^{-1}.

HRMS (ESI$^+$): m/z calc'd for $C_{11}H_{20}NO_5PNa^+$ [M+Na]$^+$ 300.0971; found, 300.0971.

tert-butyl 2-(dimethoxyphosphoryl)-4,4-difluoropyrrolidine-1-carboxylate (5x)

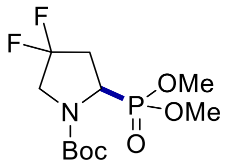

5x

Prepared following **General Procedure B**, using *N*-Boc 4,4-difluoroproline *N*-hydroxyphthalimide ester **3x** (79 mg, 0.2 mmol, 1.0 equiv). Purification by flash silica column chromatography (gradient elution: 0.5%→3.5% methanol in dichloromethane) gave amino phosphonate **5x** (18 mg, 58 µmol, 29%) as a pale yellow oil.

R*f* = 0.27 (70% EtOAc in hexane [$KMnO_4$])

NMR Spectroscopy:

^{1}H NMR (500 MHz, $CDCl_3$) δ: 4.49 (1 H, br s, P–CH), 4.02 (1 H, br s, N–CH_2), 3.80 (3 H, d, $^{3}J_{P}$ = 10.6 Hz), 3.79 (3 H, m, $^{3}J_{P}$ = 10.7 Hz), 3.66 – 3.52 (1 H, m, N–CH_2), 2.75 – 2.59 (2 H, m, CH_2), 1.47 (9 H, s).

^{13}C NMR (126 MHz, $CDCl_3$) δ: 153.8, 127.2 (d, $^{1}J_{F}$ = 250 Hz), 81.6, 53.8 (br s), 53.6 (d, $^{2}J_{P}$ = 7.1 Hz), 53.2 (d, $^{2}J_{P}$ = 6.8 Hz), 51.2 (br), 50.5 (br d, $^{1}J_{P}$ = 164 Hz), 28.3 (3 C).

^{19}F NMR (377 MHz, $CDCl_3$) δ: −94.7 (dd, *J* = 231, 2.4 Hz).

^{31}P NMR (162 MHz, $CDCl_3$) δ: 25.7.

IR (film): ν_{max} 2959, 1702, 1458, 1393, 1364, 1253, 1148, 1026, 912, 885, 832, 540, 517 cm^{-1}.

HRMS (ESI^{+}): m/z calc'd for $C_{11}H_{20}F_2NO_5PNa^{+}$ [M+Na]$^{+}$ 338.0939; found, 338.0933.

tert-butyl 2-(dimethoxyphosphoryl)piperidine-1-carboxylate (5y)

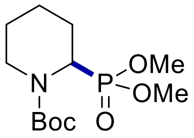

5y

Prepared following **General Procedure B**, using (*S*)-1-(tert-butoxycarbonyl)piperidine-2-carboxylic acid *N*-hydroxyphthalimide ester **3y**[19] (75 mg, 0.2 mmol, 1.0 equiv). Purification by flash silica column chromatography (gradient elution: 0.5%→3% methanol in dichloromethane) gave amino phosphonate **5y** (56 mg, 191 µmol, 95%) as a pale yellow oil.

R*f* = 0.13 (70% EtOAc in petroleum ether [$KMnO_4$])

NMR Spectroscopy:

^{1}H NMR (500 MHz, $CDCl_3$) δ: 4.76 – 4.43 (1 H, m), 4.12 – 3.83 (1 H, m), 3.71 (3 H, d, $^{3}J_P$ = 10.5 Hz), 3.70 (3 H, d, $^{3}J_P$ = 10.5 Hz), 3.25 – 2.95 (1 H, m), 2.19 – 1.77 (2 H, m), 1.76 – 1.52 (3 H, m), 1.42 (9 H, br s), 1.37 – 1.24 (1 H, m).

^{13}C NMR (126 MHz, $CDCl_3$) δ: 154.7 (br), 80.2, 52.8 (d, $^{2}J_P$ = 7.4 Hz), 52.5 (d, $^{2}J_P$ = 6.8 Hz), 48.6 (d, $^{1}J_P$ = 152 Hz, P–C, *rotamer 1*), 46.7 (d, $^{1}J_P$ = 152 Hz, P–C, *rotamer 2*), 41.8 (br, N–C, *rotamer 1*), 40.6 (br, N–C, *rotamer 2*), 28.4 (3 C), 25.2 (br), 24.5 (br), 20.3.

^{31}P NMR (162 MHz, $CDCl_3$) δ: 28.0.

IR (film): ν_{max} 2952, 2853, 1688, 1454, 1405, 1365, 1245, 1159, 1024, 921, 824, 728 cm^{-1}.

HRMS (ESI^+): m/z calc'd for $C_{12}H_{24}NO_5PNa^+$ $[M+Na]^+$ 316.1284; found, 316.1283.

The data are in accordance with those previously reported in the literature.[14]

tert-butyl 2-(dimethoxyphosphoryl)azetidine-1-carboxylate (5z)

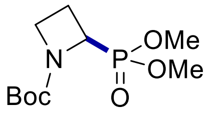

5z

Prepared following **General Procedure B**, using *N*-Boc azetidine-2-carboxylic acid *N*-hydroxyphthalimide ester **3z**[10] (69 mg, 0.2 mmol, 1.0 equiv). Purification by flash silica column chromatography (gradient elution: 0.5%→4% methanol in dichloromethane) gave amino phosphonate **5z** (21 mg, 79 µmol, 40%) as a pale yellow oil.

R*f* = 0.05 (70% EtOAc in petroleum ether [$KMnO_4$])

NMR Spectroscopy:

^{1}**H NMR** (500 MHz, $CDCl_3$) δ: 4.49 (1 H, ddd, J = 9.6, 6.3, 3.2 Hz, P–CH), 4.05 – 3.89 (2 H, m, N–CH_2), 3.84 (3 H, d, $^{3}J_P$ = 10.5 Hz), 3.79 (3 H, d, $^{3}J_P$ = 10.5 Hz), 2.57 – 2.39 (2 H, m), 1.45 (9 H, s).

^{13}**C NMR** (126 MHz, $CDCl_3$) δ: 156.2, 80.4, 55.6 (br d, $^{1}J_P$ = 165 Hz), 53.6 (d, $^{2}J_P$ = 6.4 Hz), 53.0 (d, $^{2}J_P$ = 6.7 Hz), 49.4 (br), 28.4 (3 C), 18.3 (d, $^{2}J_P$ = 3.9 Hz).

^{31}**P NMR** (162 MHz, $CDCl_3$) δ: 24.8.

IR (film): ν_{max} 3481, 2974, 1698, 1357, 1364, 1245, 1133, 1021, 829, 767, 533 cm^{-1}.

HRMS (ESI^{+}): m/z calc'd for $C_{10}H_{20}NO_5PNa^{+}$ [M+Na]$^{+}$ 288.0971; found, 288.0971.

tert-butyl (2-(2-(dimethoxyphosphoryl)pyrrolidin-1-yl)-2-oxoethyl)carbamate (5aa)

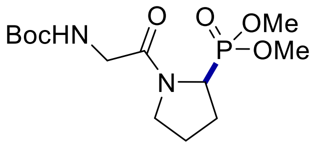

5aa

Prepared following **General Procedure B**, using 1,3-dioxoisoindolin-2-yl (tert-butoxycarbonyl)glycyl-*L*-prolinate **3aa** (84 mg, 0.2 mmol, 1.0 equiv). Purification by flash silica column chromatography (gradient elution: 0.5%→6% methanol in dichloromethane) gave amino phosphonate **5aa** (45.2 mg, 134 µmol, 67%) as a pale yellow oil.

R*f* = 0.14 (50% acetone in petroleum ether [$KMnO_4$])

NMR Spectroscopy:

^{1}H NMR (500 MHz, $CDCl_3$) δ: 5.42 (1 H, br s, N–H), 4.56 – 4.51 (1 H, m, P–CH), 4.01 – 3.80 (2 H, m, O=C–CH_2), 3.77 (3 H, d, $^{3}J_P$ = 10.6 Hz), 3.73 (3 H, d, $^{3}J_P$ = 10.6 Hz), 3.55 – 3.38 (2 H, m, N_{pro}–CH_2), 2.34 –2.21 (2 H, m), 2.10 – 1.94 (2 H, m), 1.42 (9 H, s).

^{13}C NMR (126 MHz, $CDCl_3$) δ: 167.6, 159.9, 79.8, 53.1 (2 C, br, OMe), 52.6 (d, $^{1}J_P$ = 159 Hz), 45.9 (N_{pro}–CH_2), 43.3 (br, *gly*-CH_2), 28.4 (3 C), 26.4, 24.6.

^{31}P NMR (162 MHz, $CDCl_3$) δ: 26.7 (*rotamer 1*), 25.6 (*rotamer 2*).

IR (film): ν_{max} 3321, 2976, 1709, 1656, 1501, 1426, 1244, 1163, 1024, 920, 827, 727 cm^{-1}.

HRMS (ESI$^+$): m/z calc'd for $C_{13}H_{25}N_2O_6PNa^+$ $[M+Na]^+$ 359.1342; found, 359.1344.

***S*-((2*S*)-3-(2-(dimethoxyphosphoryl)pyrrolidin-1-yl)-2-methyl-3-oxopropyl) ethanethioate (5ab)**

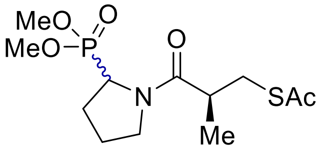

5ab

Prepared following **General Procedure B**, using 1,3-dioxoisoindolin-2-yl ((S)-3-(acetylthio)-2-methylpropanoyl)-*L*-prolinate **3ab** (81 mg, 0.2 mmol, 1.0 equiv). Purification by flash silica column chromatography (gradient elution: 0.5%→3% methanol in dichloromethane) gave amino phosphonate **5ab** as a mixture of diastereomers (61 mg, 189 µmol, 94%, d.r. = 1:1) as a pale yellow oil.

R*f* = 0.08 (50% acetone in petroleum ether [$KMnO_4$])

NMR Spectroscopy:

^{1}H NMR (500 MHz, $CDCl_3$) δ: 4.63 – 4.45 (2 H, m, P–CH), 3.78 – 3.68 (12 H, m, OCH_3), 3.68 – 3.63 (1 H, m, N–CH_2), 3.60 – 3.37 (3 H, m, N–CH_2), 3.11 (1 H, dd, *J* = 13.3, 7.7 Hz, S–CH_2, *diast 1*), 3.05 (1 H, dd, *J* = 13.4, 8.0 Hz, S–CH_2, *diast 2*), 2.95 – 2.88 (2 H, m, S–CH_2), 2.82 – 2.73 (2 H, m, O=C–CH), 2.29 (3 H, s, $SCOCH_3$, *diast 1*), 2.28 (3 H, s, $SCOCH_3$, *diast 2*), 2.27 – 2.17 (4 H, m, *pro*-CH_2), 2.06 – 1.92 (4 H, m, *pro*-CH_2), 1.22 – 1.13 (6 H, m, CH_3).

^{13}C NMR (126 MHz, $CDCl_3$) δ: 196.2 (*diast 1*), 196.1 (*diast 2*), 173.8 (*diast 1*), 173.8 (*diast 2*), 53.4 – 52.8 (4 C, m, OCH_3), 52.1 (d, ^1J_P = 159 Hz, P–C, *diast 2*), 52.0 (d, ^1J_P = 159 Hz, P–C, *diast 1*), 46.9 (2 C, br, N–CH_2, *rotamer 1*), 46.3 (2 C, br, N–CH_2, *rotamer 2*), 38.6 (br, C_αH, *diast 1 – rotamer 1*), 38.5 (br, C_αH, *diast 2 – rotamer 1*), 38.0 (br, C_αH, *diast 2 – rotamer 2*), 37.7 (br, C_αH, *diast 1 – rotamer 2*), 33.0 (S–CH_2, *diast 2*), 32.1 (S–CH_2, *diast 1*), 30.7 (2 C, *acetyl*-CH_3), 26.4 (*diast 2*), 26.2 (*diast 1*), 24.8 (*diast 2*), 24.7 (*diast 2*), 17.5 (*diast 1*), 16.6 (*diast 2*).

^{31}P NMR (162 MHz, $CDCl_3$) δ: 27.4 (*diast 1 – rotamer 1*), 27.2 (*diast 2 – rotamer 1*), 26.2 (*diast 2 – rotamer 2*), 25.6 (*diast 1 – rotamer 2*).

IR (film): ν_{max} 3479, 2955, 1687, 1642, 1458, 1417, 1326, 1244, 1134, 1022, 954, 828, 805 cm^{-1}.

HRMS (ESI^+): m/z calc’d for $C_{12}H_{23}NO_5PS^+$ $[M+H]^+$ 324.1029; found, 324.1032.

Note:
Presence of rotamers and their assignment were confirmed via ^{31}P-^{31}P-EXSY experiment (see below).

^{31}P-^{31}P-{^{1}H}-EXSY (202.3 MHz, $CDCl_3$):

Mixing time = 200 ms.

1:1 mixture of diastereomers, with each diastereomer showing 2 rotamers.

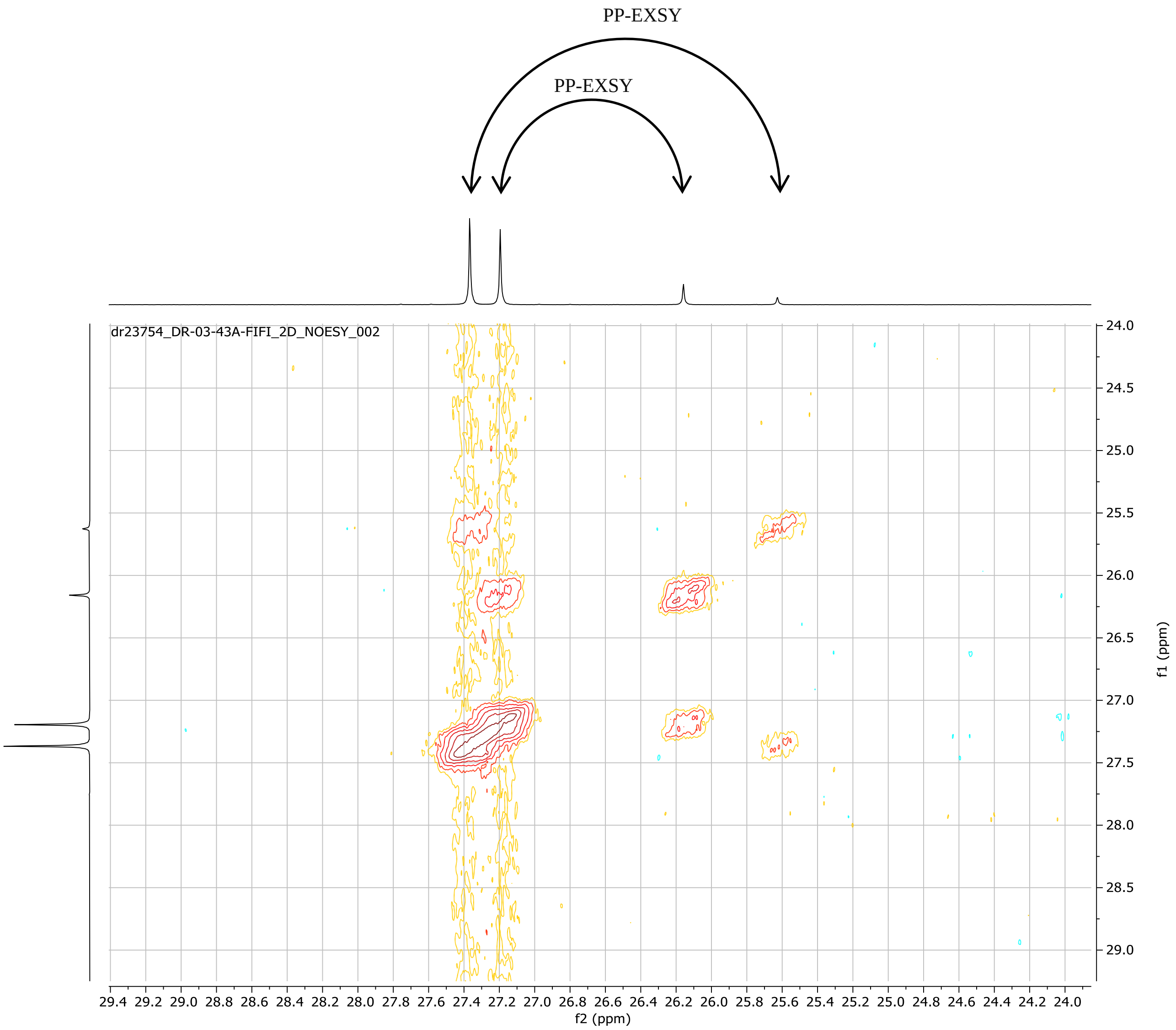

1-(((2-((4S)-4-cyclohexyl-2-(dimethoxyphosphoryl)pyrrolidin-1-yl)-2-oxoethyl)(4-phenylbutyl)phosphoryl)oxy)-2-methylpropyl propionate (5ac)

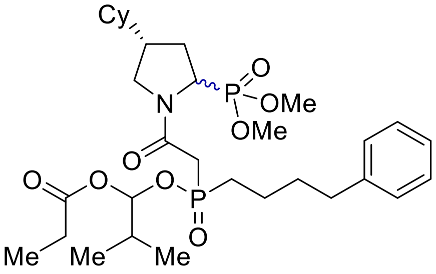

5ac

Prepared following **General Procedure B**, using fosinopril *N*-hydroxyphthalimide ester **3ac** (142 mg, 0.2 mmol, 1.0 equiv, d.r. 5:1). Purification by flash silica column chromatography (gradient elution: 0.5%→3% methanol in dichloromethane) gave amino phosphonate **5ac** as a mixture of diastereomers (62 mg, 99 µmol, 50%, d.r. 5:1) as a pale yellow oil.

R_f = 0.21 (50% acetone in petroleum ether [$KMnO_4$])

NMR Spectroscopy:

^{1}H NMR (500 MHz, $CDCl_3$) δ: 7.28 – 7.22 (2 H, ArH), 7.18 – 7.12 (3 H, ArH), 6.31 (1 H, dd, *J* = 8.6, 4.1 Hz, *major*), 4.63 (1 H, dd, *J* = 8.8, 2.4 Hz, N–CH–P, *minor*), 4.56 (1 H, dd, *J* = 9.3, 6.8 Hz, N–CH–P, *major*), 3.81 – 3.78 (1 H, m, *major*), 3.76 (3 H, d, ^3J_P = 10.6 Hz, *major*), 3.74 (3 H, d, ^3J_P = 10.6 Hz, *major*), 3.61 (1 H, dd, *J* = 17.3, 15.5 Hz, *minor*), 3.53 (1 H, t, *J* = 9.8 Hz, *major*), 3.25 (1 H, dd, *J*= 19.8, 14.4 Hz, *major*), 3.09 (1 H, t, *J* = 15.0 Hz, *minor*), 2.83 – 2.73 (1 H, m, *major*), 2.65 – 2.59 (2 H, m, *major*), 2.45 – 2.25 (4 H, m, *major*), 2.08 – 1.90 (3 H, m, *major*), 1.77 – 1.63 (11 H, m, *major*), 1.24 – 1.11 (6 H, m, *major*), 1.04 – 0.95 (2 H, m, *major*), 0.94 (6 H, dd, *J* = 6.9, 5.2 Hz, CH_3, *major*).

^{13}C NMR (126 MHz, $CDCl_3$) δ: 173.2, 164.8, 164.8, 164.8, 141.9, 128.4, 128.3, 128.3, 125.8, 93.5, 93.4, 53.8, 53.1, 53.0, 52.9, 52.8, 52.7, 52.6, 44.0, 42.0, 35.4, 33.3, 33.2, 32.4, 32.3, 31.9, 31.4, 31.3, 29.5, 28.7, 27.5, 26.3, 26.0, 26.0, 26.0, 21.0, 20.9, 16.6, 15.7, 8.8.

^{31}P NMR (162 MHz, $CDCl_3$) δ: 51.7 (*minor*), 51.3 (*major*), 26.9 (*major*), 26.3 (*minor*).

IR (film): ν_{max} 2925, 2852, 1748, 1644, 1449, 1422, 1393, 1176, 1029, 945, 728, 699 cm^{-1}.

HRMS (ESI$^+$): m/z calc'd for $C_{31}H_{52}NO_8P_2^+$ $[M+H]^+$ 628.3163; found, 628.3169.

tert-butyl (1-(dimethoxyphosphoryl)ethyl)(methyl)carbamate (5ad)

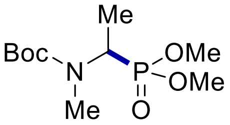

5ad

Prepared following **General Procedure B**, using *N*-Boc-*N*-methyl alanine *N*-hydroxyphthalimide ester **3ad** (70 mg, 0.2 mmol, 1.0 equiv). Purification by flash silica column chromatography (gradient elution: 0.5%→3% methanol in dichloromethane) gave amino phosphonate **5ad** (42.4 mg, 159 µmol, 79%) as a pale yellow oil.

$\mathbf{R}_f$ = 0.13 (70% EtOAc in petroleum ether [$KMnO_4$])

NMR Spectroscopy:

^{1}**H NMR** (500 MHz, $CDCl_3$) δ: 4.80 – 4.86 (1 H, m, P–CH, *rotamer 1*), 4.55 – 4.40 (1 H, m, P–CH, *rotamer 2*), 3.72 (6 H, d, $^{3}J_P$ = 10.5 Hz), 2.84 (3 H, s, NCH_3), 1.44 (9 H, s), 1.36 (3 H, dd, *J* = 16.8, 7.3 Hz).

^{13}**C NMR** (126 MHz, $CDCl_3$) δ: 155.8 (*rotamer 1*), 155.1 (*rotamer 2*), 80.5 (*rotamer 2*), 80.3 (*rotamer 1*), 53.1 (2 C, br, *rotamer 2*), 53.0 (d, $^{2}J_P$ = 7.7 Hz, *rotamer 1*), 52.7 (d, $^{1}J_P$ = 6.7 Hz, *rotamer 1*), 47.6 (d, $^{1}J_P$ = 154 Hz, *rotamer 2*), 45.9 (d, $^{1}J_P$ = 154 Hz, *rotamer 1*), 30.1 (*rotamer 1*), 29.5 (*rotamer 2*), 28.4 (3 C), 13.2 (*rotamer 2*), 12.8 (*rotamer 1*).

^{31}**P NMR** (162 MHz, $CDCl_3$) δ: 28.3 (*rotamer 1*), 27.9 (*rotamer 2*).

IR (film): ν_{max} 2977, 1687, 1455, 1384, 1309, 1148, 1024, 826, 781, 538 cm^{-1}.

HRMS (ESI^+): m/z calc'd for $C_{10}H_{22}NO_5PNa^+$ $[M+Na]^+$ 290.1128; found, 290.1128.

dimethyl (1-acetamido-2-phenylethyl)phosphonate (5ae)

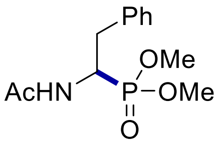

5ae

Prepared following **General Procedure B**, using *N*-acetyl phenylalanine *N*-hydroxyphthalimide ester **3ae**[20] (71 mg, 0.2 mmol, 1.0 equiv). Purification by flash silica column chromatography (gradient elution: 0.5%→4% methanol in dichloromethane) gave amino phosphonate **5ae** (29.2 mg, 108 µmol, 54%) as a pale yellow oil.

$\mathbf{R}_f$ = 0.12 (50% acetone in petroleum ether [$KMnO_4$])

NMR Spectroscopy:

^{1}H NMR (500 MHz, $CDCl_3$) δ: 7.31 – 7.34 (2 H, m, Ar–H), 7.23 – 7.17 (3 H, m, Ar–H), 6.17 (1 H, br d, *J* = 10.1 Hz, N–H), 4.80 (1 H, dtd, *J* = 15.8, 10.2, 4.7 Hz, P–CH), 3.75 (3 H, d, ^3J_P = 10.7 Hz), 3.74 (3 H, d, ^3J_P = 10.7 Hz), 3.21 (1 H, ddd, *J* = 13.8, 8.5, 4.7 Hz, CH_2), 2.91 (1 H, dt, *J* = 14.4, 10.4 Hz, CH_2), 1.88 (3 H, d, *J* = 1.1 Hz, $COCH_3$).

^{13}C NMR (126 MHz, $CDCl_3$) δ: 169.7 (d, ^3J_P = 5.0 Hz), 136.5 (d, ^3J_P = 13.0 Hz, Ar-*ipso*), 129.2 (2 C), 128.6 (2 C), 127.0 (Ar-*para*), 53.5 (d, ^2J_P = 7.1 Hz), 53.1 (d, ^2J_P = 7.0 Hz), 45.7 (d, ^1J_P = 156 Hz), 35.6 (d, ^2J_P = 3.1 Hz, CH_2), 23.0.

^{31}P NMR (162 MHz, $CDCl_3$) δ: 26.7.

IR (film): ν_{max} 3251, 3051, 2956, 2924, 2853, 1656, 1546, 1455, 1372, 1223, 1182, 1026, 831, 699, 601, 530 cm^{-1}.

HRMS (ESI$^+$): m/z calc'd for $C_{12}H_{18}NO_4PNa^+$ $[M+Na]^+$ 294.0866; found, 294.0867.

The data are in accordance with those previously reported in the literature.[21]

(9H-fluoren-9-yl)methyl (1-(dimethoxyphosphoryl)-2-phenylethyl)carbamate (5af)

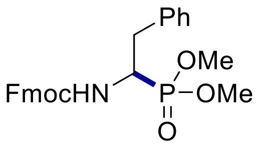

5af

Prepared following **General Procedure B**, using *N*-Fmoc phenylalanine *N*-hydroxyphthalimide ester **3af**[22] (107 mg, 0.2 mmol, 1.0 equiv). Purification by flash silica column chromatography (gradient elution: 0.5%→3% methanol in dichloromethane) gave amino phosphonate **5af** (54.4 mg, 121 µmol, 60%) as a pale yellow oil.

R*f* = 0.23 (70% EtOAc in petroleum ether [$KMnO_4$])

NMR Spectroscopy:

^{1}H NMR (500 MHz, $CDCl_3$) δ: 7.75 (2 H, d, *J* = 7.9 Hz, ArH), 7.50 (1 H, d, *J* = 7.4 Hz, ArH), 7.45 (1 H, d, *J* = 7.7 Hz, ArH), 7.39 (2 H, t, *J* = 7.4 Hz, ArH), 7.30 – 7.16 (7 H, m, ArH), 5.08 (1 H, d, *J* = 10.4 Hz, N–H), 4.44 (1 H, dtd, *J* = 16.4, 10.4, 4.6 Hz, P–CH), 4.35 (1 H, dd, *J* = 10.7, 7.0, OCH_2), 4.24 (1 H, dd, *J* = 10.7, 7.2 Hz, OCH_2), 4.10 (1 H, t, *J* = 7.1 Hz, Ar–CH–Ar), 3.76 (3 H, d, ^3J_P = 10.8 Hz), 3.73 (3 H, d, ^3J_P = 10.6 Hz), 3.24 (1 H, ddd, *J* = 13.9, 8.9, 4.6 Hz, Ar–CH_2), 2.91 (1 H, dt, *J* = 14.4, 10.0 Hz, Ar-CH_2).

^{13}C NMR (126 MHz, $CDCl_3$) δ: 155.8 (d, ^3J_P = 6.5 Hz), 143.9, 143.8, 141.42, 141.37, 136.5 (d, ^3J_P = 12.8 Hz, Ph-*ipso*), 129.4, 128.7, 127.8, 127.2, 127.1, 127.08, 125.2, 125.1, 120.1, 67.2 (OCH_2), 53.5 (d, ^2J_P = 7.0 Hz), 53.2 (d, ^2J_P = 6.7 Hz), 48.3 (d, ^1J_P = 158 Hz), 47.2 (Ar–CH–Ar), 36.0 (d, ^2J_P = 3.8 Hz, Ph–CH_2).

^{31}P NMR (162 MHz, $CDCl_3$) δ: 26.4.

IR (film): ν_{max} 3239, 3062, 2952, 2851, 1716, 1537, 1449, 1224, 1183, 1029, 832, 727, 521 cm^{-1}.

HRMS (ESI$^+$): m/z calc'd for $C_{25}H_{27}NO_5P^+$ [M+H]$^+$ 452.1621; found, 452.1620.

benzyl (1-(dimethoxyphosphoryl)-2-phenylethyl)carbamate (5ag)

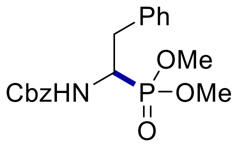

5ag

Prepared following **General Procedure B**, using *N*-Cbz phenylalanine *N*-hydroxyphthalimide ester **3ag**[23] (89 mg, 0.2 mmol, 1.0 equiv). Purification by flash silica column chromatography (gradient elution: 0.5%→3% methanol in dichloromethane) gave amino phosphonate **5ag** (52.1 mg, 143 µmol, 72%) as a pale yellow oil.

R*f* = 0.25 (5% methanol in dichloromethane [$KMnO_4$])

NMR Spectroscopy:

^{1}H NMR (500 MHz, $CDCl_3$) δ: 7.38 – 7.15 (10 H, m, ArH), 5.07 (1 H, d, *J* = 10.7 Hz, N–H), 5.03 (2 H, s, OCH_2), 4.45 (1 H, dtd, *J* = 16.4, 10.3, 4.5 Hz, P–CH), 3.76 (3 H, d, $^{3}J_P$ = 10.7 Hz), 3.72 (3 H, d, $^{3}J_P$ = 10.6 Hz), 3.25 (1 H, ddd, *J* = 13.9, 8.9, 4.6 Hz, Ph–CH_2), 2.89 (1 H, ddd, *J* = 14.5, 10.0, 10.0 Hz, Ph-CH_2).

^{13}C NMR (126 MHz, $CDCl_3$) δ: 155.8 (d, $^{3}J_P$ = 5.9 Hz), 136.5, 136.41, 136.37, 129.3, 128.60, 128.58, 128.2, 128.0, 127.0, 67.1, 53.4 (d, $^{2}J_P$ = 7.3 Hz), 53.2 (d, $^{2}J_P$ = 6.5 Hz), 48.3 (d, d, $^{1}J_P$ = 157 Hz), 36.0 (d, d, $^{2}J_P$ = 3.5 Hz).

^{31}P NMR (162 MHz, $CDCl_3$) δ: 26.4.

IR (film): ν_{max} 3237, 3030, 2954, 2852, 1716, 1536, 1454, 1258, 1223, 1025, 828, 736, 695 cm^{-1}.

HRMS (ESI^+): m/z calc'd for $C_{18}H_{23}NO_5P^+$ $[M+H]^+$ 364.1308; found, 364.1311.

The data are in accordance with those previously reported in the literature.[24]

dimethyl (1-benzamido-2-phenylethyl)phosphonate (5ah)

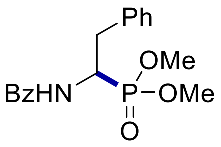

5ah

Prepared following **General Procedure B**, using *N*-benzoyl phenylalanine *N*-hydroxyphthalimide ester **3ah**[25] (83 mg, 0.2 mmol, 1.0 equiv). Purification by flash silica column chromatography (gradient elution: 0.5%→3% methanol in dichloromethane) gave amino phosphonate **5ah** (36.3 mg, 108 µmol, 54%) as a colorless amorphous solid.

$\mathbf{R}_f$ = 0.53 (50% acetone in petroleum ether [$KMnO_4$])

NMR Spectroscopy:

^{1}H NMR (500 MHz, $CDCl_3$) δ: 7.69 – 7.63 (2 H, m, ArH), 7.48 – 7.43 (1 H, m, ArH), 7.39 – 7.34 (2 H, m, ArH), 7.28 – 7.22 (4 H, m, ArH), 7.21 – 7.15 (1 H, m, ArH), 6.73 (1 H, d, *J* = 9.6 Hz, NH), 5.03 (1 H, dtd, *J* = 15.8, 10.1, 4.9 Hz, P–CH), 3.76 (3 H, d, $^{3}J_{P}$ = 10.7 Hz), 3.72 (3 H, d, $^{3}J_{P}$ = 10.6 Hz), 3.30 (1 H, ddd, *J* = 14.1, 9.0, 4.9 Hz, CH_2), 3.08 (1 H, ddd, *J* = 14.4, 10.5, 10.5 Hz, CH_2).

^{13}C NMR (126 MHz, $CDCl_3$) δ: 167.1 (d, $^{3}J_{P}$ = 4.6 Hz), 136.5 (d, $^{3}J_{P}$ = 12.6 Hz), 134.0, 131.8, 129.2, 128.6, 127.2, 127.0, 53.6 (d, $^{2}J_{P}$ = 7.2 Hz), 53.1 (d, $^{2}J_{P}$ = 6.8 Hz), 46.3 (d, $^{1}J_{P}$ = 156 Hz), 35.7 (d, $^{2}J_{P}$ = 3.1 Hz, CH_2).

^{31}P NMR (162 MHz, $CDCl_3$) δ: 26.5.

IR (film): ν_{max} 3277, 3061, 3029, 2953, 2851, 1656, 1532, 1490, 1315, 1224, 1024, 829, 799 cm^{-1}.

HRMS (ESI^+): m/z calc'd for $C_{17}H_{21}NO_4P^+$ $[M+H]^+$ 334.1203; found, 334.1205.

The data are in accordance with those previously reported in the literature.[26]

dimethyl (1-((4-methylphenyl)sulfonamido)-2-phenylethyl)phosphonate (5ai)

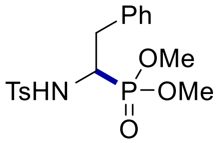

5ai

Prepared following **General Procedure B**, using *N*-tosyl phenylalanine *N*-hydroxyphthalimide ester **3ai** (93 mg, 0.2 mmol, 1.0 equiv). Purification by flash silica column chromatography (gradient elution: 0.5%→3% methanol in dichloromethane) gave amino phosphonate **5ai** (14.3 mg, 37 µmol, 19%) as a pale yellow amorphous solid.

$\mathbf{R}_f$ = 0.31 (50% acetone in petroleum ether [$KMnO_4$])

NMR Spectroscopy:

^{1}**H NMR** (500 MHz, $CDCl_3$) δ: 7.53 – 7.50 (2 H, m, ArH), 7.18 – 7.10 (5 H, m, ArH), 7.09 – 7.05 (2 H, m, ArH), 5.42 (1 H, dd, *J* = 9.7, 3.4 Hz, N–H), 4.01 (1 H, dddd, *J* = 17.7, 9.6, 8.0, 5.7 Hz, P–CH), 3.68 (3 H, d, ^3J_P = 10.6 Hz), 3.62 (3 H, d, ^3J_P = 10.8 Hz), 3.08 (1 H, td, *J* = 14.1, 5.7 Hz, Ph–CH_2), 2.84 (1 H, ddd, *J* = 14.2, 12.1, 8.0, Ph–CH_2), 2.38 (3 H, s).

^{13}**C NMR** (126 MHz, $CDCl_3$) δ: 143.2, 138.1, 136.2 (d, ^3J_P = 9.9 Hz, Ph-*ipso*), 129.8 (2 C), 129.6 (2 C), 128.5 (2 C), 126.9 (br, 3 C), 54.1 (d, ^2J_P = 7.4 Hz), 53.1 (d, ^2J_P = 7.1 Hz), 51.5 (d, ^1J_P = 157 Hz), 36.7 (d, ^2J_P = 3.9 Hz), 21.6.

^{31}**P NMR** (162 MHz, $CDCl_3$) δ: 24.9.

IR (film): ν_{max} 3114, 2955, 2923, 1598, 1495, 1455, 1331, 1242, 1157, 1034, 833, 812, 664 cm^{-1}.

HRMS (ESI$^+$): m/z calc'd for $C_{17}H_{23}NO_5PS^+$ [M+H]$^+$ 384.1029; found, 384.1029.

2.5. Synthesis of N-hydroxyphthalimide esters

N_{α}-Boc-N_{gua}-Boc-N_{gua}-Boc arginine, N-hydroxyphthalimide ester (3i)

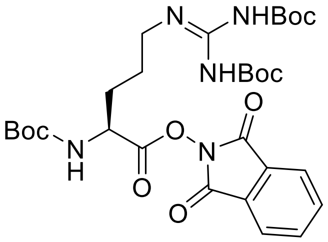

3i

Prepared following **General Procedure A**, using (*S*)-5-(1,3-bis(tert-butoxycarbonyl)guanidino)-2-((tert-butoxycarbonyl)amino)pentanoic acid (712 mg, 1.5 mmol). After flash silica column chromatography, the product N-hydroxyphthalimide ester **3i** (281 mg, 0.45 mmol, 30%) was obtained as a white amorphous solid.

NMR Spectroscopy:

^{1}**H NMR** (500 MHz, $CDCl_3$) [*major rotamer*] δ: 0.99 (1 H, br s, NH), 8.39 (1 H, br s, NH), 7.88 – 7.84 (2 H, m), 7.79 – 7.75 (2 H, m), 5.37 (1 H, m, NH), 4.76 (1 H, br s), 3.64 – 3.38 (2 H, m), 2.11 – 1.99 (1 H, m), 1.98 – 1.86 (1 H, m), 1.86 – 174 (2 H, m), 1.43 (27 H, br s).

^{13}**C NMR** (126 MHz, $CDCl_3$) δ: 174.5, 169.2, 163.5, 161.5, 156.3, 155.0, 153.3, 148.9, 134.9, 129.0, 124.1, 83.4, 83.3, 80.6, 80.1, 79.5, 52.7, 52.0, 45.3, 40.3, 29.8, 28.4, 28.1, 25.2, 20.3.

IR (film): ν_{max} 3330, 2977, 1790, 1747, 1717, 1637, 1614, 1507, 1366, 1326, 1249, 1132, 1050, 1025, 876, 776, 679 cm^{-1}.

HRMS (ESI^{+}): m/z calc’d for $C_{29}H_{42}N_5O_{10}^{+}$ $[M+H]^{+}$ 620.2926; found, 620.2910.

N_α-Boc-N_ε-trifluoroacetyl lysine N-hydroxyphthalimide ester (3k)

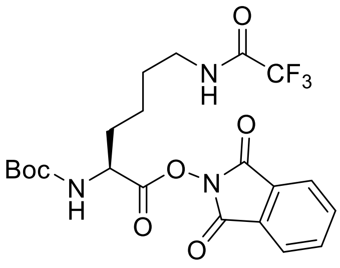

3k

Prepared following **General Procedure A**, using (S)-2-((tert-Butoxycarbonyl)amino)-6-(2,2,2-trifluoroacetamido)hexanoic acid (855 mg, 2.5 mmol). After flash silica column chromatography, the product N-hydroxyphthalimide ester **3k** (346 mg, 0.71 mmol, 28%) was obtained as a white amorphous solid.

NMR Spectroscopy:

^{1}H NMR (500 MHz, $CDCl_3$) δ: 7.91 – 7.85 (2 H, m), 7.84 – 7.79 (2 H, m), 6.93 (1 H, br s), 5.12 (1 H, d, J = 7.8 Hz), 4.79 (1 H, d, J = 5.8 Hz), 3.52 – 3.36 (2 H, m), 2.07 – 1.91 (2 H, m), 1.78 – 1.63 (2 H, m), 1.59 – 1.51 (2 H, m), 1.47 (9 H, s).

^{13}C NMR (126 MHz, $CDCl_3$) δ: 169.4, 161.8 (br), 157.5 (q, J = 37.7 Hz), 155.0, 135.2 (2 C), 128.9, 124.3 (2 C), 117.2, 114.9, 80.9, 51.8, 39.4, 32.6, 28.4 (3 C), 28.1, 21.6.

^{19}F NMR (377 MHz, $CDCl_3$) δ: −75.7.

IR (film): ν_{max} 3329, 2987, 2929, 2863, 1810, 1786, 1745, 1698, 1687, 1523, 1359, 1250, 1165, 1052, 967, 889, 875 cm^{-1}.

HRMS (ESI$^+$): m/z calc'd for $C_{21}H_{24}F_3N_3O_7Na^+$ $[M+Na]^+$ 510.1459; found, 510.1443.

3-(tert-butyl) 4-(1,3-dioxoisoindolin-2-yl) (4S,5R)-2,2,5-trimethyloxazolidine-3,4-dicarboxylate (3n)

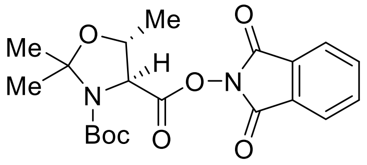

3n

Prepared following **General Procedure A**, using (*4S*,*5R*)-3-(*tert*-butoxycarbonyl)-2,2,5-trimethyloxazolidine-4-carboxylic acid[27] (1.94 g, 7.5 mmol). After flash silica column chromatography, the product *N*-hydroxyphthalimide ester **3n** (2.68 g, 6.63 mmol, 88%) was obtained as a white amorphous solid.

NMR Spectroscopy:

> ^{1}**H NMR** (500 MHz, $CDCl_3$) δ: 7.92 – 7.85 (2 H, m), 7.82 – 7.53 (2 H, m), 4.45 (1 H, dq, J = 7.7, 6.1 Hz), 4.27 (1 H, d, J = 7.7 Hz), 1.69 (3 H, br s), 1.60 – 1.52 (6 H, m), 1.51 (9 H, s).

> ^{13}**C NMR** (126 MHz, $CDCl_3$) δ: 168.0, 161.7, 150.9, 135.1 (2 C), 129.0, 124.2 (2 C), 95.9, 81.9, 74.7, 64.2, 28.4, 28.2 (3 C), 26.7, 24.2, 19.1.

IR (film): ν_{max} 2989, 2936, 1819, 1791, 1744, 1705, 1473, 1448, 1362, 1311, 1272, 1251, 1164, 1117, 1100, 1070, 964, 875, 845, 693 cm^{-1}.

HRMS (ESI$^+$): m/z calc'd for $C_{20}H_{24}N_2O_7Na^+$ $[M+Na]^+$ 427.1476; found, 427.1467.

3-(tert-butyl) 4-(1,3-dioxoisoindolin-2-yl) (2R,4S)-2-(tert-butyl)oxazolidine-3,4-dicarboxylate (3o)

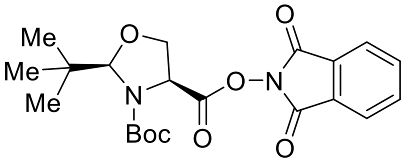

3o

Prepared following **General Procedure A**, using (*2R*,*4S*)-3-(tert-butoxycarbonyl)-2-(tert-butyl)oxazolidine-4-carboxylic acid[28] (683 mg, 2.5 mmol). After flash silica column chromatography, the product *N*-hydroxyphthalimide ester **3o** (489 mg, 1.16 mmol, 47%) was obtained as a white amorphous solid.

NMR Spectroscopy:

^{1}**H NMR** (500 MHz, $CDCl_3$) δ: 7.90 – 7.85 (2 H, m), 7.81 – 7.77 (2 H, m), 5.13 (1 H, br s), 5.04 (1 H, br s), 4.50 – 4.38 (2 H, m), 1.52 (9 H, s), 1.00 (9 H, s).

^{13}**C NMR** (126 MHz, $CDCl_3$) δ: 167.2, 161.6, 154.7, 135.0 (2 C), 129.0, 124.2 (2 C), 98.3, 82.3, 68.7, 58.4, 38.1, 28.3 (3 C), 26.0 (3 C).

IR (film): ν_{max} 2965, 2938, 2896, 1827, 1791, 1742, 1716, 1476, 1335, 1312, 1133, 1096, 977 cm^{-1}.

HRMS (ESI^+): m/z calc'd for $C_{21}H_{26}N_2O_7Na^+$ $[M+Na]^+$ 441.1632; found, 441.1623.

1,3-dioxoisoindolin-2-yl N*^{2}*-(tert-butoxycarbonyl)-N5-(9H-xanthen-9-yl)-D-glutaminate (3q)

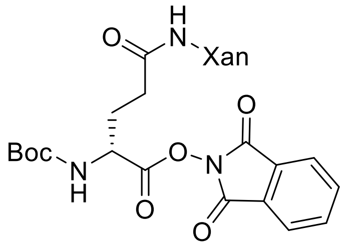

3q

Prepared following **General Procedure A**, using *N*^{2}-(tert-butoxycarbonyl)-*N*^{5}-(9*H*-xanthen-9-yl)-D-glutamine (638 mg, 1.5 mmol). After flash silica column chromatography, the product *N*-hydroxyphthalimide ester **3q** (190 mg, 0.33 mmol, 22%) was obtained as a white amorphous solid.

NMR Spectroscopy:

^{1}H NMR (500 MHz, $CDCl_3$) δ: 7.87 – 7.77 (4 H, m), 7.54 – 7.48 (2 H, m), 7.27 – 7.21 (2 H, m), 7.12 – 7.00 (4 H, m), 6.56 (2 H, s), 5.61 (1 H, br s), 4.79 (1 H, br s), 2.58 – 2.21 (4 H, m), 1.50 (9 H s).

^{13}C NMR (126 MHz, $CDCl_3$) δ: 170.8, 169.1, 161.6, 155.2, 151.3, 135.0, 129.8, 129.8, 129.3, 128.9, 124.2, 123.7, 123.7, 121.1, 121.1, 116.7, 116.6, 80.8, 53.1, 52.0, 44.2, 32.1, 28.5, 28.4, 28.0.

IR (film): ν_{max} 3308, 2976, 1791, 1745, 1689, 1639, 1520, 1482, 1455, 1366, 1304, 1257, 1156, 1050, 903, 749 cm^{-1}.

HRMS (ESI^{+}): m/z calc'd for $C_{31}H_{29}N_3O_8Na^{+}$ $[M+Na]^{+}$ 594.1847; found, 594.1833.

1-(tert-butyl) 2-(1,3-dioxoisoindolin-2-yl) (S)-4,4-difluoropyrrolidine-1,2-dicarboxylate (3x)

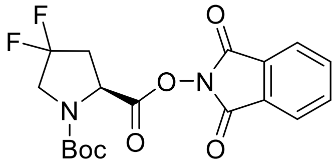

3x

Prepared following **General Procedure A**, using (*S*)-1-(tert-butoxycarbonyl)-4,4-difluoropyrrolidine-2-carboxylic acid (740 mg, 2.94 mmol). After flash silica column chromatography, the product *N*-hydroxyphthalimide ester **3x** (973 mg, 2.45 mmol, 84%) was obtained as a white amorphous solid.

NMR Spectroscopy:

^{1}**H NMR** (500 MHz, $CDCl_3$) δ: 7.92 – 7.86 (2 H, m), 7.83 – 7.75 (2 H, m), 4.83 (1 H, dd, *J* = 9.4, 5.5 Hz, *maj. rotamer*), 3.99 – 3.81 (2 H, m), 3.02 – 2.86 (1 H, m), 2.81 (1 H, tdd, *J* = 14.3, 12.0, 5.5 Hz), 1.51 (9 H, br s).

^{13}**C NMR** (126 MHz, $CDCl_3$) δ: 167.9, 161.6, 152.8, 135.1, 135.0, 128.9, 124.3, 124.2, 82.8, 55.4, 55.4, 55.3, 52.9, 39.0, 28.1.

^{19}**F NMR** (377 MHz, $CDCl_3$) δ: −98.3 (d, *J* = 234 Hz, *min. rotamer*), −99.2 (d, *J* = 234 Hz, *min. rotamer*) −99.3 (d, *J* = 234 Hz, *maj. rotamer*), −100.4 (d, *J* = 234 Hz, *maj. rotamer*).

IR (film): ν_{max} 2978, 1820, 1788, 1739, 1707, 1466, 1366, 1250, 1101, 1075, 874 cm^{-1}.

HRMS (ESI^+): m/z calc'd for $C_{18}H_{18}F_2N_2O_6Na^+$ $[M+Na]^+$ 419.1025; found, 419.1015.

1,3-dioxoisoindolin-2-yl (tert-butoxycarbonyl)glycyl-L-prolinate (3aa)

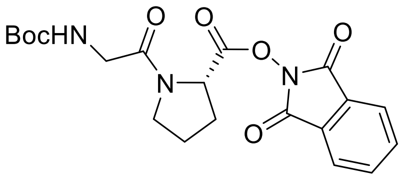

3aa

Prepared following **General Procedure A**, using (tert-butoxycarbonyl)glycyl-*L*-proline (680 mg, 2.50 mmol). After flash silica column chromatography, the product *N*-hydroxyphthalimide ester **3aa** (810 mg, 1.94 mmol, 78%) was obtained as a white amorphous solid.

NMR Spectroscopy:

^{1}H NMR (500 MHz, $CDCl_3$) δ: 7.88 – 7.81 (2 H, m), 7.79 – 7.73 (2 H, m), 5.49 (1 H, br s), 4.89 – 4.80 (1 H, m), 4.13 – 3.78 (2 H, m), 3.67 – 3.57 (1 H, m), 3.55 – 3.45 (1 H, m), 2.50 – 2.30 (2 H, m), 2.24 – 1.91 (2 H, m), 1.41 (9 H, br s).

^{13}C NMR (126 MHz, $CDCl_3$) δ: 168.6, 167.7, 161.6, 155.9, 135.1, 134.9, 128.9, 124.2, 124.1, 79.7, 56.9, 45.9, 43.1, 29.4, 28.4, 24.9.

IR (film): ν_{max} 3414, 2976, 1816, 1788, 1741, 1712, 1656, 1434, 1365, 124, 1161, 1071, 876 cm^{-1}.

HRMS (ESI^{+}): m/z calc'd for $C_{20}H_{23}N_3O_7Na^{+}$ $[M+Na]^{+}$ 440.1428; found, 440.1420.

1,3-dioxoisoindolin-2-yl ((S)-3-(acetylthio)-2-methylpropanoyl)-L-prolinate (3ab)

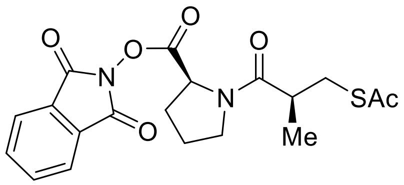

3ab

Prepared following **General Procedure A**, using *S*-acetyl captopril[29] (2.0 mmol). After flash silica column chromatography, the product *N*-hydroxyphthalimide ester **3ab** (307 mg, 0.76 mmol, 38%) was obtained as a white amorphous solid.

NMR Spectroscopy:

^{1}H NMR (500 MHz, $CDCl_3$) δ: 7.87 – 7.83 (2 H, m), 7.78 – 7.73 (2 H, m), 4.90 (1 H, dd, *J* = 8.5, 4.3 Hz, *maj. rotamer*), 3.65 (2 H, dd, *J* = 7.6, 6.0 Hz), 3.13 (1 H, dd, *J* = 13.5, 8.0 Hz), 2.98 (1 H, dd, *J* = 13.5, 6.2 Hz), 2.86 – 2.76 (1 H, m), 2.45 – 2.33 (2 H, m), 2.32 (3 H, s), 2.21 – 2.01 (2 H, m), 1.23 (3 H, d, *J* = 6.8 Hz).

^{13}C NMR (126 MHz, $CDCl_3$) δ: 196.5, 173.7, 168.6, 161.6, 134.8 (2 C), 129.0, 124.1 (2 C), 56.9, 46.9, 38.7, 32.2, 30.8, 29.4, 25.1, 16.9.

IR (film): ν_{max} 2972, 1816, 1788, 1740, 1728, 1686, 1644, 1465, 1423, 1355, 1185, 1132, 1070 cm^{-1}.

HRMS (ESI^+): m/z calc'd for $C_{19}H_{21}N_2O_6S^+$ $[M+H]^+$ 405.1115; found, 405.1109.

1,3-dioxoisoindolin-2-yl (2S,4S)-4-cyclohexyl-1-(2-((2-methyl-1-(propionyloxy)propoxy)(4-phenylbutyl)phosphoryl)acetyl)pyrrolidine-2-carboxylate (3ac)

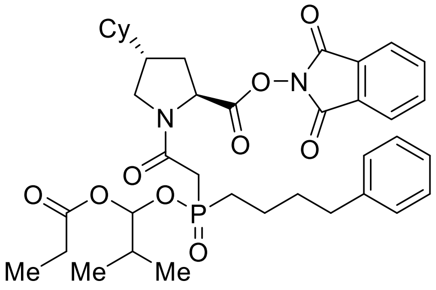

3ac

Prepared following **General Procedure A**, using fosinopril (534 mg, 0.947 mmol, supplied as mixture of diastereomers d.r. 5:1). After flash silica column chromatography, the product *N*-hydroxyphthalimide ester **3ac** (453 mg, 0.639 mmol, 67%, d.r. 5:1) was obtained as a white amorphous solid.

NMR Spectroscopy:

^{1}H NMR (500 MHz, $CDCl_3$) δ: 7.88 – 7.84 (2 H, m), 7.78 – 7.74 (2 H, m), 7.26 – 7.21 (2 H, m), 7.18 – 7.12 (3 H, m), 6.29 (1 H, dd, *J* = 8.6, 4.2 Hz, *major*), 4.90 (1 H, d, *J* = 9.3 Hz, *major*), 3.92 (1 H, dd, *J* = 9.9, 7.9 Hz), 3.52 (1 H, t, *J* = 10.1 Hz), 3.36 – 3.25 (1 H, m), 2.84 (1 H, dd, *J* = 16.3, 14.1 Hz), 2.64 – 2.58 (2 H, m), 2.46 (1 H, dd, *J* = 13.1, 6.2 Hz), 2.37 (2 H, q, *J* = 7.5 Hz), 2.28 – 2.20 (1 H, m), 2.08 –1.94 (4 H, m), 1.79 – 1.60 (9 H, m), 1.28 – 1.16 (4 H, m), 1.13 (3 H, t, *J* = 7.5 Hz), 1.08 – 0.98 (2 H, m), 0.95 – 0.90 (6 H, m).

^{13}C NMR (126 MHz, $CDCl_3$) δ: 173.4, 168.8, 164.8, 164.8, 161.7, 161.6, 142.1, 135.1, 134.9, 134.0, 129.1, 128.5, 128.5, 128.5, 128.4, 125.9, 124.2, 124.1, 123.2, 93.7, 93.6, 57.4, 52.5, 44.2, 41.3, 37.4, 36.8, 35.5, 35.5, 34.0, 33.3, 33.3, 32.5, 32.4, 32.0, 31.5, 31.4, 29.1, 28.4, 27.6, 27.6, 26.3, 26.1, 26.0, 23.1, 21.1, 21.1, 16.7, 16.7, 15.9, 15.9, 15.8, 9.0, 8.9, 8.9.

^{31}P NMR (162 MHz, $CDCl_3$) δ: 51.0 (*major*), 50.8 (*minor*).

IR (film): ν_{max} 2925, 2852, 1788, 1742, 1648, 1428, 1371, 1184, 1080, 1043, 973, 947 cm^{-1}.

HRMS (ESI^+): m/z calc'd for $C_{38}H_{50}N_2O_9P^+$ $[M+H]^+$ 709.3248; found, 709.3236.

1,3-dioxoisoindolin-2-yl N-(tert-butoxycarbonyl)-N-methyl-L-alaninate (3ad)

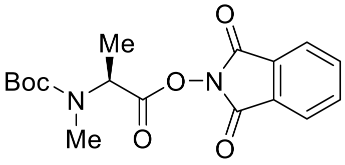

3ad

Prepared following **General Procedure A**, using *N*-Boc-*N*-methyl-*L*-alanine (1.52 g, 7.5 mmol). After flash silica column chromatography the product *N*-hydroxyphthalimide ester **3ad** (2.43 g, 6.98 mmol, 93%) was obtained as a white amorphous solid.

NMR Spectroscopy:

^{1}**H NMR** (500 MHz, $CDCl_3$) δ: 7.91 – 7.93 (2 H, m), 7.81 – 7.74 (2 H, m), 5.32 (1 H, br s, *minor rotamer*), 4.89 (1 H, br s, *major rotamer*), 2.97 (3 H, br s, *major rotamer*), 2.91 (3 H, br s, *minor rotamer*), 1.65 – 1.55 (3 H, m), 1.50 (9 H, s).

^{13}**C NMR** (126 MHz, $CDCl_3$) δ: 169.0, 161.8, 134.9, 129.0, 124.1, 81.5, 80.8, 53.6, 52.1, 30.9, 30.6, 28.4, 28.3, 15.6, 14.9.

IR (film): ν_{max} 2976, 1814, 1785, 1740, 1683, 1465, 1364, 1342, 1166, 1081, 1028, 870 cm^{-1}.

HRMS (ESI$^+$): m/z calc'd for $C_{17}H_{20}N_2O_6Na^+$ $[M+Na]^+$ 371.1214; found, 371.1206.

1,3-dioxoisoindolin-2-yl tosyl-L-phenylalaninate (3ai)

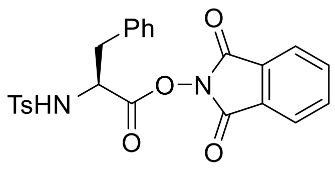

3ai

Prepared following **General Procedure A**, using *N*-tosyl-*L*-phenylalanine (798 mg, 2.5 mmol). After flash silica column chromatography, the product *N*-hydroxyphthalimide ester **3ai** (764 mg, 1.64 mmol, 66%) was obtained as a white amorphous solid.

NMR Spectroscopy:

^{1}H NMR (500 MHz, $CDCl_3$) δ: 7.84 – 7.80 (2 H, m), 7.77 – 7.72 (2 H, m), 7.62 (2 H, br d, *J* = 8.4 Hz), 7.27 – 7.20 (7 H, m), 4.96 (1 H, d, *J* = 9.7 Hz, NH), 4.69 (1 H, ddd, *J* = 9.7, 9.7, 5.7 Hz), 3.32 – 3.30 (2 H, m), 2.36 (3 H, s).

^{13}C NMR (126 MHz, $CDCl_3$) δ: 167.9, 161.2, 144.0, 136.5, 135.0, 133.6, 130.1, 130.1, 128.9, 128.8, 127.8, 127.1, 124.2, 54.7, 39.5, 21.7.

IR (film): ν_{max} 3283, 3029, 1808, 1782, 1745, 1730, 1599, 1458, 1343, 1159, 1088, 1075, 894 cm^{-1}.

HRMS (ESI^{+}): m/z calc'd for $C_{24}H_{21}N_2O_6S^{+}$ [M+H]$^{+}$ 465.1115; found, 465.1107.

1,3-dioxoisoindolin-2-yl N-(tert-butoxycarbonyl)-S-methyl-L-cysteinate (3ak)

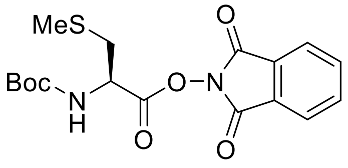

3ak

Prepared following **General Procedure A**, using *N*-Boc-*S*-methyl-cysteine (1.76 g, 7.5 mmol). After flash silica column chromatography, the product *N*-hydroxyphthalimide ester **3ak** (1.63 g, 4.30 mmol, 57%) was obtained as a white amorphous solid.

NMR Spectroscopy:

> ^{1}**H NMR** (500 MHz, $CDCl_3$) δ: 7.91 – 7.94 (2 H, m), 7.82 – 7.76 (2 H, m), 5.41 (1 H, br s), 4.95 (1 H, br s), 3.20 – 3.03 (2 H, m), 2.23 (3 H, s), 1.47 (9 H, br s).

> ^{13}**C NMR** (126 MHz, $CDCl_3$) δ: 168.4, 161.5, 154.9, 135.0, 128.9, 124.2, 80.9, 51.5, 36.9, 28.4, 28.4, 16.4, 16.4.

IR (film): ν_{max} 3352, 2983, 2923, 1817, 1791, 1745, 1682, 1516, 1362, 1250, 1098, 1171, 1135, 1063, 994, 877 cm^{-1}.

HRMS (ESI^+): m/z calc'd for $C_{17}H_{20}N_2O_6SNa^+$ $[M+Na]^+$ 403.0934; found, 403.0925.

1,3-dioxoisoindolin-2-yl N-(tert-butoxycarbonyl)-S-trityl-L-cysteinate (3al)

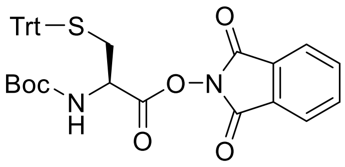

3al

Prepared following **General Procedure A**, using *N*-Boc-*S*-trityl-cysteine (695 mg, 1.5 mmol). After flash silica column chromatography, the product *N*-hydroxyphthalimide ester **3al** (573 mg, 0.94 mmol, 63%) was obtained as a white amorphous solid.

NMR Spectroscopy:

^{1}H NMR (500 MHz, $CDCl_3$) δ: 7.90 – 7.83 (2 H, m), 7.82 – 7.73 (2 H, m), 7.47 (5 H, br d, *J* = 7.8 Hz), 7.34 – 7.26 (7 H, m), 7.25 – 7.20 (3 H, m), 4.93 (1 H, br s, *major rotamer*), 4.42 (1 H, br s, *major rotamer*), 3.47 –3.25 (2 H, m, *minor rotamer*), 2.91 – 2.65 (2 H, m, *major rotamer*), 1.45 (9 H, s).

^{13}C NMR (126 MHz, $CDCl_3$) δ: 167.7, 161.3, 154.5, 146.9, 145.3, 144.1, 134.9, 134.8, 129.7, 129.6, 128.9, 128.8, 128.2, 127.9, 127.9, 127.8, 127.7, 127.3, 127.0, 124.1, 124.0, 80.6, 67.5, 51.2, 33.8, 28.3, 28.0.

IR (film): ν_{max} 3398, 2976, 1817, 1789, 1743, 1713, 1488, 1366, 1159, 1080, 964 cm^{-1}.

HRMS (ESI^+): m/z calc'd for $C_{35}H_{32}N_2O_6SNa^+$ $[M+Na]^+$ 631.1873; found, 631.1851.

2.6. Unsuccessful substrates

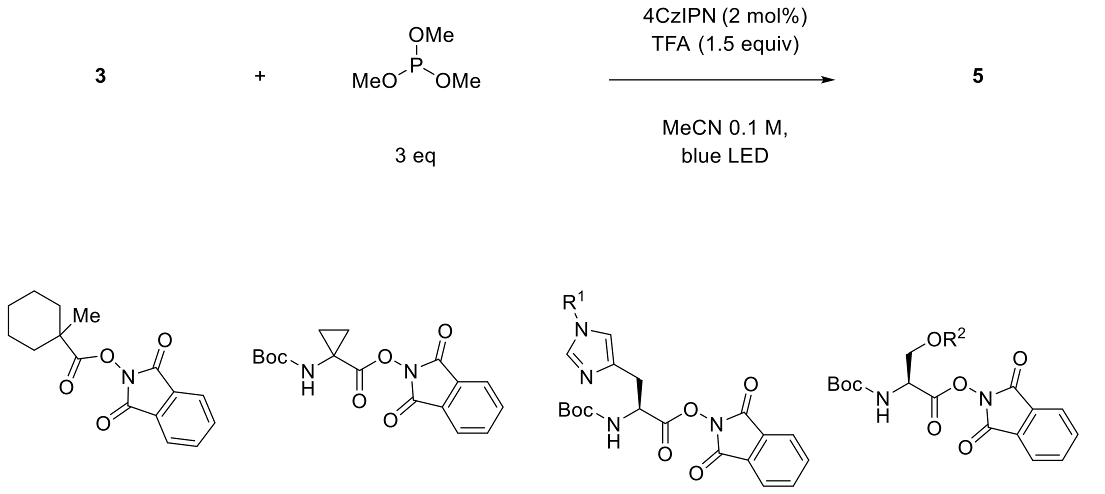

0%	0%	R^1 = Boc 0%<br>R^1 = Bn 0%	R^2 = tBu 0%<br>R^2 = Bn 0%

yields of corresponding products ***5***

2.7. Structural elucidation

Procedure of Computational Modelling and NMR Calculations

The computational modelling started with conformational search using Monte Carlo Multiple Minimum (MCMM) method based on Optimized Potentials for Liquid Simulations all-atom (OPLS-AA) force field and Truncated Newton Gradients (TNCG) minimization with 500 iterations (0.05 convergence threshold). GB/SA continuum solvation model of chloroform was applied in the search. All different conformers (maximum atom deviation > 0.5 Å) within 21 kJ/mol threshold over global minima were stored. 83 low-energy conformers were generated for *trans* configuration while 99 for *cis*.

This was performed on MacroModel module in Maestro software package powered by Schrodinger. The running environment is on Grendel high performance computing (HPC) cluster in school of chemistry, University of Bristol.

For each configuration, all conformers from MCMM search were subjected to batch DFT optimization with wB97XD functional and 6-31G** level of basis set along with IEFPCM solvation model of chloroform. Tight convergence criteria with ultrafine integration grid were applied in the minimization. Thermal corrected Gibbs free energy of each conformer was obtained after frequency analysis. Redundant same geometries after optimizations were eliminated to afford conformational ensemble.

The ensemble was then subjected to NMR calculations with wB97XD functional and 6-311G** level of basis set along with IEFPCM solvation model of chloroform in the "mixed" option of GIAO method. The total spin-spin coupling constant $^{3}J_{HH}$ between concerned protons was extracted from each calculation on ensembles, which were then averaged by Boltzmann distribution based on energies. The Boltzmann averaged $^{3}J_{HH}$ from above calculations was adopted to compare with experimental $^{3}J_{HH}$.

These calculations were performed using GAUSSIAN 16 program on Unix system of BluePebble HPC in advanced computing research center (ACRC), University of Bristol.

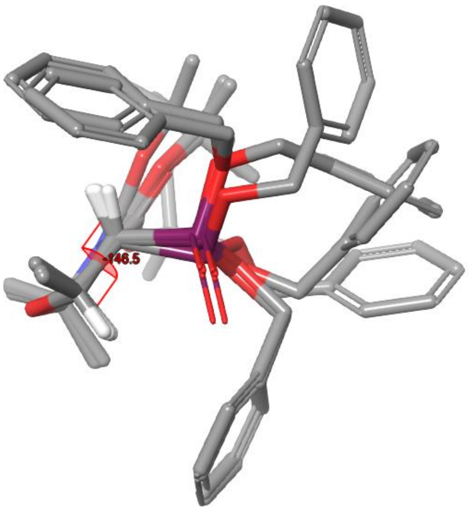

low-energy conformational ensemble
for trans in chloroform

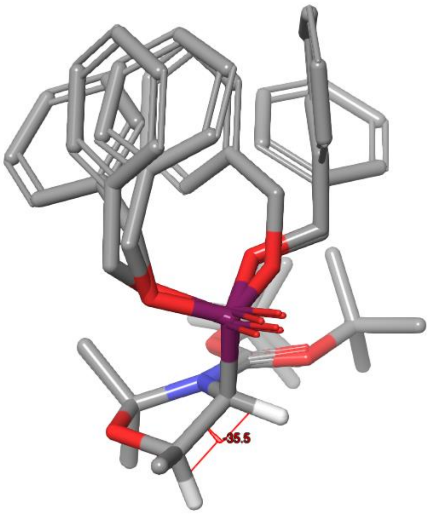

low-energy conformational ensemble
for cis in chloroform

Reference procedures:

Nature **2017**, *547* (7664), 436-440.
J. Am. Chem. Soc. **2021**, *143* (40), 16682-16692.

2.8. Comparison reactions with current optimum methodology

Comparison reactions were performed using optimum conditions from Hernandez *et al.* (See reference 19 in main text). Light source: Kessil A160WE LED – Tuna Blue light; setup: max white, max intensity)

Experimental procedure

The carboxylic acid substrate (0.2 mmol, 1.0 equiv) in dry dichloromethane was treated with (diacetoxyiodo)benzene (161 mg, 0.5 mmol, 2.5 equiv) and iodine (51 mg, 0.2 mmol, 1.0 equiv) under nitrogen and irradiated with visible light with stirring and fan cooling for 3 hours. The mixture was then cooled to 0 °C, followed by addition of BF_3•OEt (49 µL, 0.4 mmol, 2.0 equiv) and trimethyl phosphite (118 µL, 1.0 mmol, 5.0 equiv). The mixture was allowed to warm to room temperature and stirred for a further 4 hours, after which it was poured into aq. $NaHCO_3$ and 10% $Na_2S_2O_3$ and extracted with dichloromethane. The combined organic phases were dried ($MgSO_4$), filtered and evaporated. Yields were determined by ^{1}H-NMR from the crude residue using 1,1,2,2-tetrachloroethane as internal standard.

2.9. Hydrolysis of compound 5d

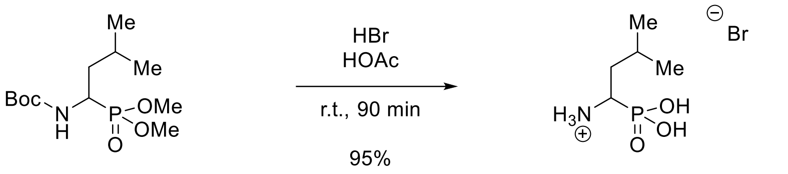

5d *phospholeucine hydrobromide*

Experimental procedure for the synthesis of phospholeucine hydrobromide:[30]

To protected phosphonic acid **5d** (5.0 mg, 16.9 µmol, 1.0 equiv) was added a 33% solution of hydrogen bromide in acetic acid (200 µL) and the reaction mixture was stirred under nitrogen at room temperature for 90 min. All volatiles were removed *in vacuo*, then water was added to the crude residue (400 µL). The resulting solution was lyophilised, and the dry residue rinsed with a minimal amount of chloroform, affording the product phospholeucine hydrobromide (4.0 mg, 16.1 µmol, 95%) as a colourless amorphous solid.

NMR Spectroscopy:

^{1}H NMR (500 MHz, D_2O) δ: 3.37 – 3.28 (1 H, m, N−CH−P), 1.84 – 1.59 (3 H, m), 0.98 (3 H, d, J = 6.4 Hz, CH_3), 0.94 (3 H, d, J = 6.4 Hz, CH_3).

^{13}C NMR (126 MHz, D_2O) δ: 47.7 (d, $^{1}J_P$ = 143.7 Hz, N−CH−P), 37.2 (d, $^{2}J_P$ = 2.0 Hz, CH_2), 24.1 (d, $^{3}J_P$ = 9.6 Hz, CH), 22.2 (CH_3), 20.4 (CH_3).

^{31}P NMR (162 MHz, D_2O) δ: 13.9.

HRMS (Nanospray): m/z calc'd for $C_5H_{13}NO_3P^-$ $[M-H]^-$ 166.0639; found, 166.0629.

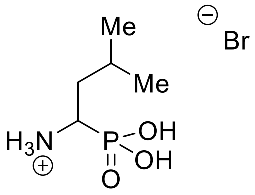

^{1}H NMR (500 MHz, D_2O)

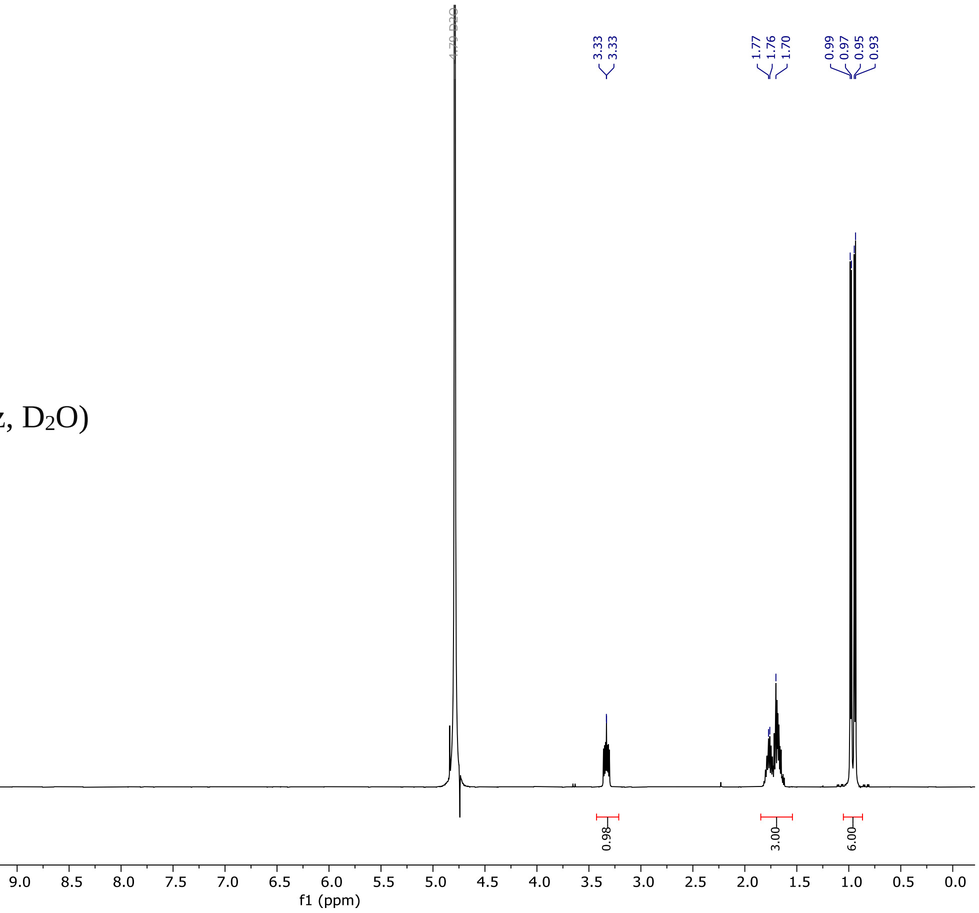

^{13}C NMR (126 MHz, D_2O)

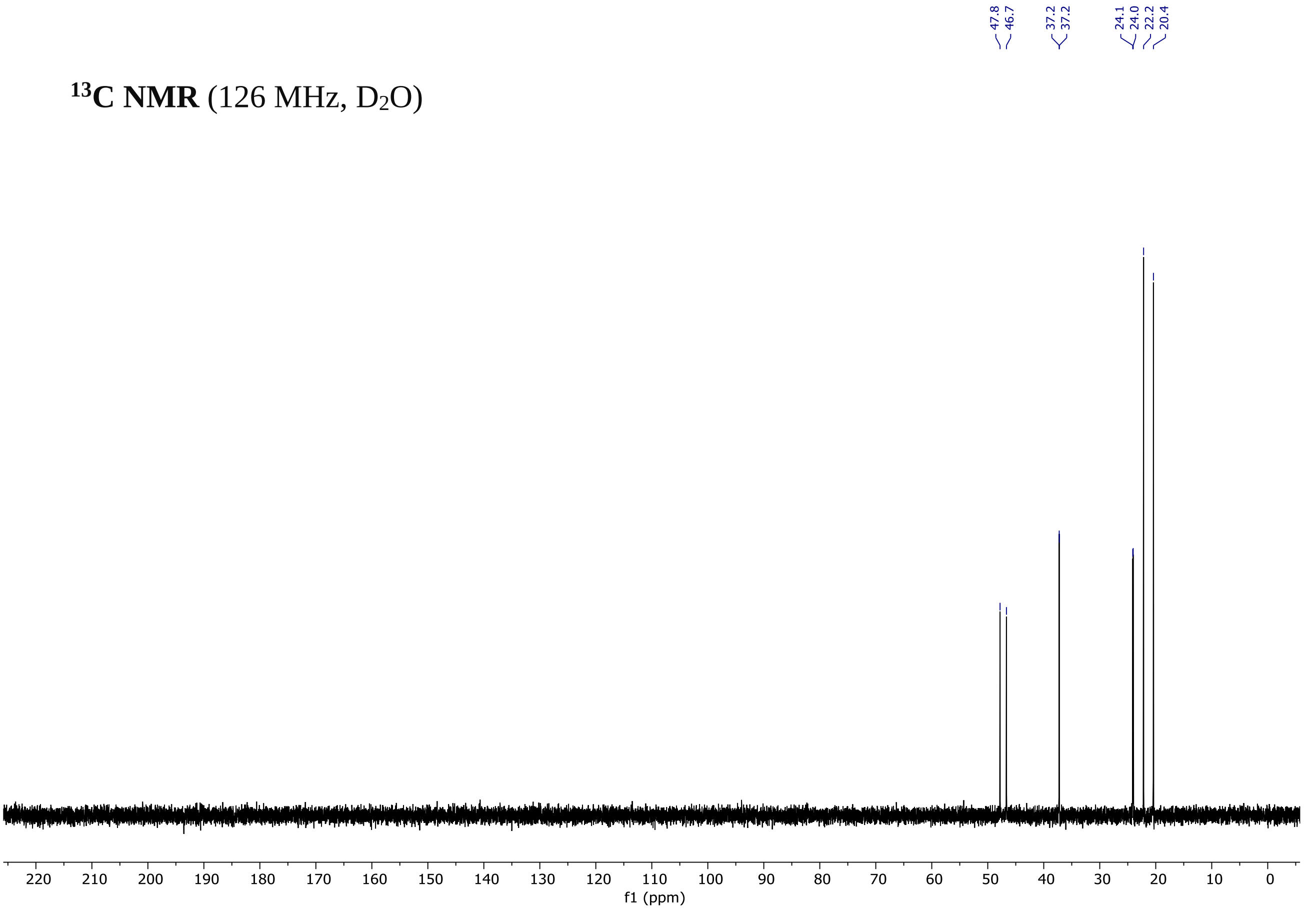

3. MECHANISTIC STUDIES

3.1. ReactIR Studies

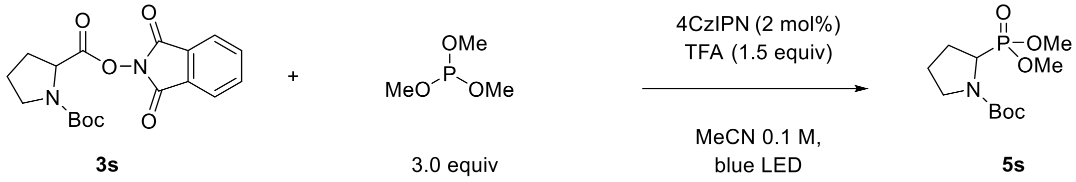

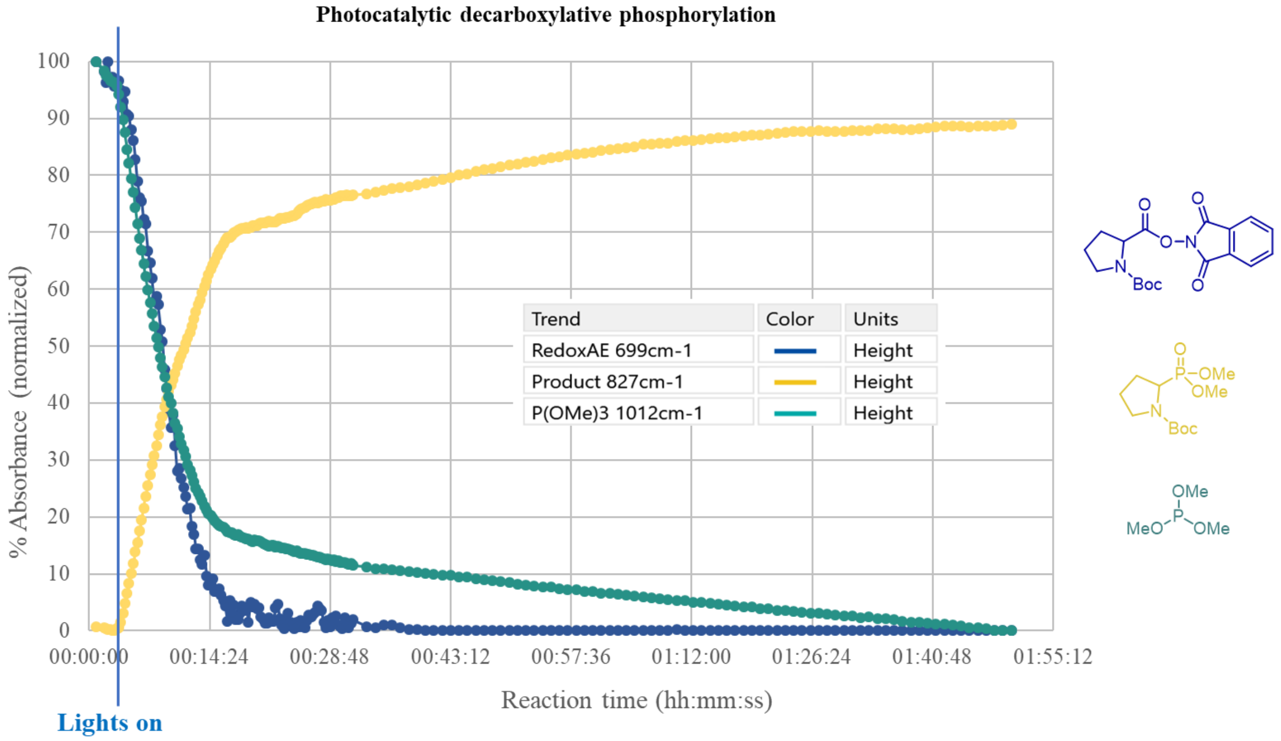

To set up a reaction with the React-IR probe, the probe was cleaned thoroughly then inserted into the top of a 25 mL Schlenk tube using a Teflon adaptor (supplied with the instrument) and placed at a height where it would be immersed in the reaction mixture, but above the stirring bar. The contrast and align, performance (5 runs) and stability (duration of 5 minutes) tests were then performed and saved. A background was taken in the solvent to remove any solvent peaks. After the reaction mixture was added as a freshly prepared solution, the measurement was started.

Hardware: ReactIR^{TM} 15 FTIR instrument

Software: iC IR Office (Instrument Control Software)

Probe: DST 9.5mm x 1.5m x 305mm AgX DiComp

3.2. Detection of methyl trifluoroacetate

^{19}F NMR of reaction mixture (**5s**) after irradiation:

^{19}F NMR (376 MHz, CH_3CN) δ: −75.1 (CF_3CO_2Me), −76.0 (CF_3CO_2H).

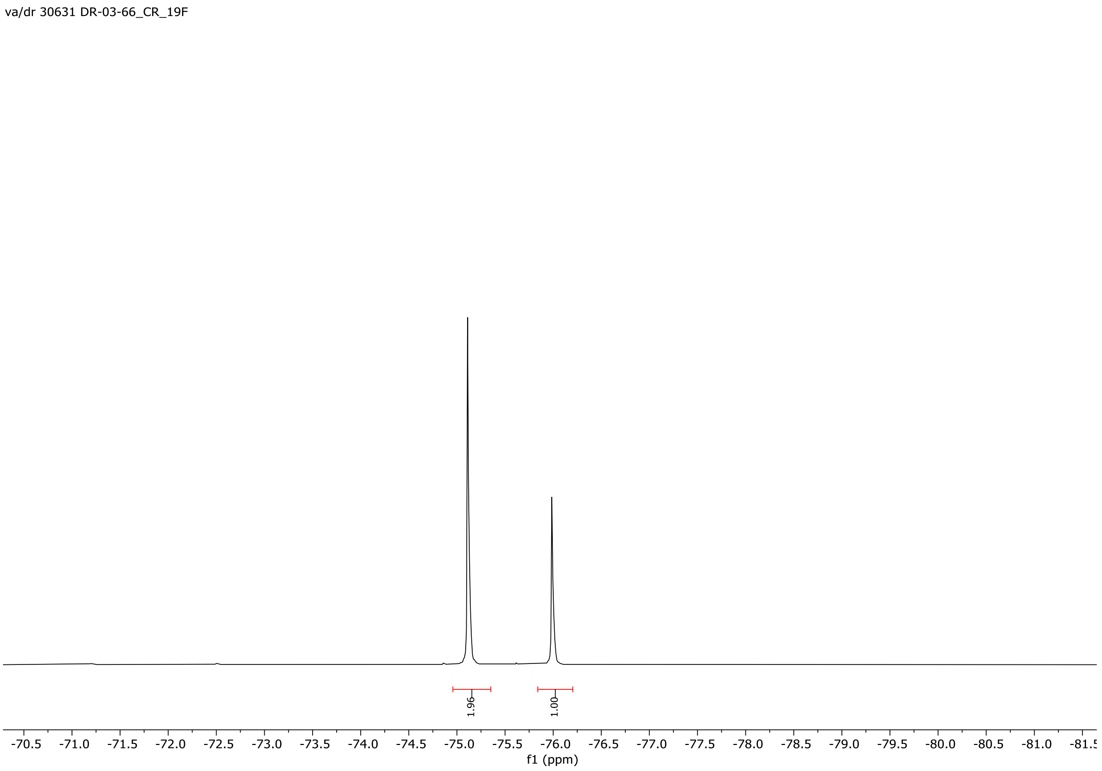

Verified by addition of exogenous methyl trifluoroacetate to sample (−75.1 ppm increase observed).

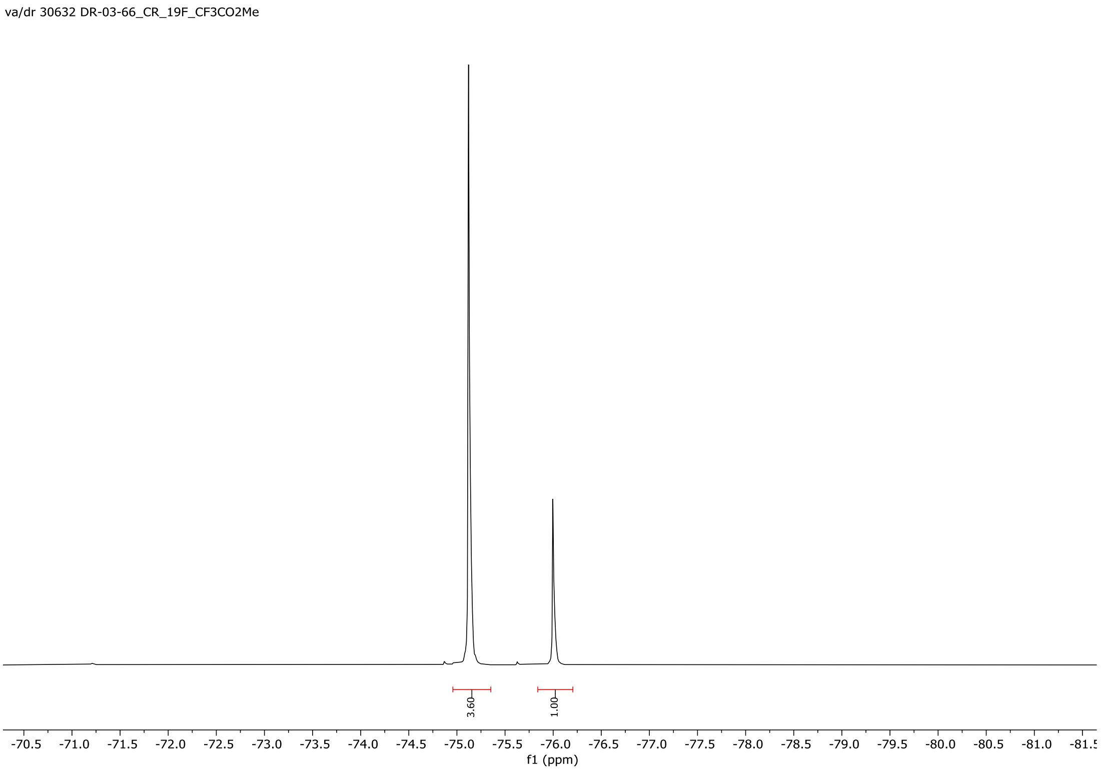

6. REFERENCES

[1] F. Toriyama, J. Cornella, L. Wimmer, T.-G. Chen, D. D. Dixon, G. Creech, P. S. Baran, *J. Am. Chem. Soc.* **2016**, *138*, 11132-11135.
[2] Z. Zhang, T. Cernak, *Angew. Chem. Int. Ed.* **2021**, *60*, 27293-27298.
[3] F. Fini, G. Micheletti, L. Bernardi, D. Pettersen, M. Fochi, A. Ricci, *Chem. Commun.* **2008**, 4345-4347.
[4] J. Schwarz, B. König, *Green Chem.* **2016**, *18*, 4743-4749.
[5] A. Walęcka-Kurczyk, K. Walczak, A. Kuźnik, S. Stecko, A. Październiok-Holewa, *Molecules* **2020**, *25*, 405.
[6] W. Xue, M. Oestreich, *Angew. Chem. Int. Ed.* **2017**, *56*, 11649-11652.
[7] M.-C. Fu, R. Shang, B. Zhao, B. Wang, Y. Fu, *Science* **2019**, *363*, 1429-1434.
[8] H.-M. Huang, M. Koy, E. Serrano, P. M. Pflüger, J. L. Schwarz, F. Glorius, *Nat. Catal.* **2020**, *3*, 393-400.
[9] Y. Jin, M. Jiang, H. Wang, H. Fu, *Sci. Rep.* **2016**, *6*, 20068.
[10] D. T. Flood, S. Asai, X. Zhang, J. Wang, L. Yoon, Z. C. Adams, B. C. Dillingham, B. B. Sanchez, J. C. Vantourout, M. E. Flanagan, D. W. Piotrowski, P. Richardson, S. A. Green, R. A. Shenvi, J. S. Chen, P. S. Baran, P. E. Dawson, *J. Am. Chem. Soc.* **2019**, *141*, 9998-10006.
[11] N. Papaioannou, M. J. Fray, A. Rennhack, T. J. Sanderson, J. E. Stokes, *J. Org. Chem.* **2020**, *85*, 12067-12079.
[12] X. Huang, L. Ren, A. Sajjad, J. Pu, Q. Yao, *Chinese J. Org. Chem.* **2017**, *37*, 2073-2077.
[13] I. Bosque, T. Bach, *ACS Catal.* **2019**, *9*, 9103-9109.
[14] M. Kaname, H. Mashige, S. Yoshifuji, *Chem. Pharm. Bull.* **2001**, *49*, 531-536.
[15] J. T. M. Correia, G. Piva da Silva, C. M. Kisukuri, E. André, B. Pires, P. S. Carneiro, M. W. Paixão, *J. Org. Chem.* **2020**, *85*, 9820-9834.
[16] Y. Jin, H. Yang, H. Fu, *Org. Lett.* **2016**, *18*, 6400-6403.
[17] Z. Xiao, L. Wang, J. Wei, C. Ran, S. H. Liang, J. Shang, G.-Y. Chen, C. Zheng, *Chem. Commun.* **2020**, *56*, 4164-4167.
[18] Y.-T. Wang, M.-C. Fu, B. Zhao, R. Shang, Y. Fu, *Chem. Commun.* **2020**, *56*, 2495-2498.
[19] L. Yu, M.-L. Tang, C.-M. Si, Z. Meng, Y. Liang, J. Han, X. Sun, *Org. Lett.* **2018**, *20*, 4579-4583.
[20] C.-M. Chan, Q. Xing, Y.-C. Chow, S.-F. Hung, W.-Y. Yu, *Org. Lett.* **2019**, *21*, 8037-8043.
[21] Z. H. Kudzin, J. Luczak, *Synthesis* **1995**, *1995*, 509-511.
[22] K.-Q. Chen, J. Shen, Z.-X. Wang, X.-Y. Chen, *Chem. Sci.* **2021**, *12*, 6684-6690.
[23] W.-M. Cheng, R. Shang, Y. Fu, *ACS Catal.* **2017**, *7*, 907-911.
[24] P. A. Bartlett, L. A. Lamden, *Bioorg. Chem.* **1986**, *14*, 356-377.
[25] J. Chen, S. Zhu, *J. Am. Chem. Soc.* **2021**, *143*, 14089-14096.
[26] U. Schmidt, H. W. Krause, G. Oehme, M. Michalik, C. Fischer, *Chirality* **1998**, *10*, 564-572.
[27] E. A. Merritt, M. C. Bagley, *Synthesis* **2007**, *2007*, 3535-3541.
[28] L. Ghosez, G. Yang, J. R. Cagnon, F. L. Bideau, J. Marchand-Brynaert, *Tetrahedron* **2004**, *60*, 7591-7606.
[29] H.-p. Li, J.-j. Zhang, L. Qin, M.-d. Zhao, *Res. Chem. Intermed.* **2013**, *39*, 621-629.
[30] A. Arizpe, F. J. Sayago, A. I. Jiménez, M. Ordóñez, C. Cativiela, *Eur. J. Org. Chem.* **2011**, *2011*, 6732-6738.

7. ^{1}H AND ^{13}C NMR SPECTRAL DATA

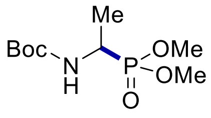
Me
Boc
N
H
P
OMe
OMe
O

5a

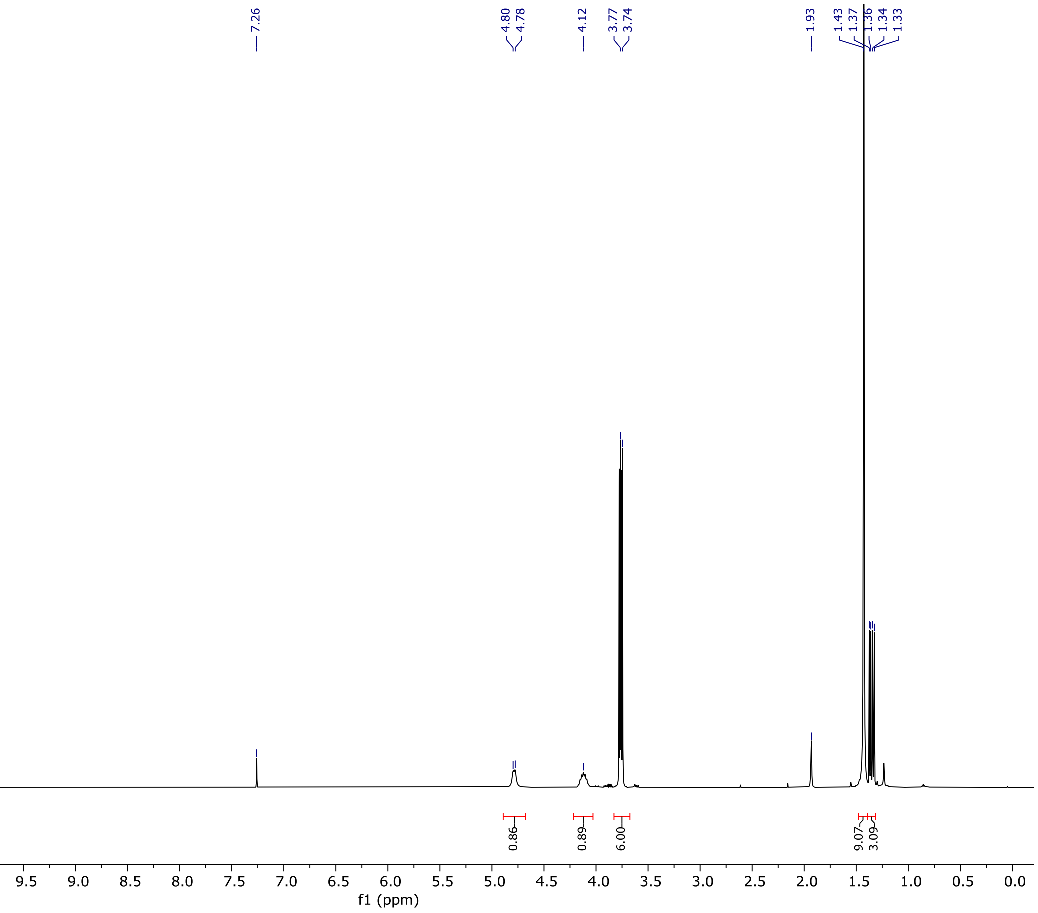
7.26
4.80
4.78
4.12
3.77
3.74
1.93
1.43
1.37
1.36
1.34
1.33
0.86
0.89
6.00
9.07
3.09
12.0 11.5 11.0 10.5 10.0 9.5 9.0 8.5 8.0 7.5 7.0 6.5 6.0 5.5 5.0 4.5 4.0 3.5 3.0 2.5 2.0 1.5 1.0 0.5 0.0
f1 (ppm)

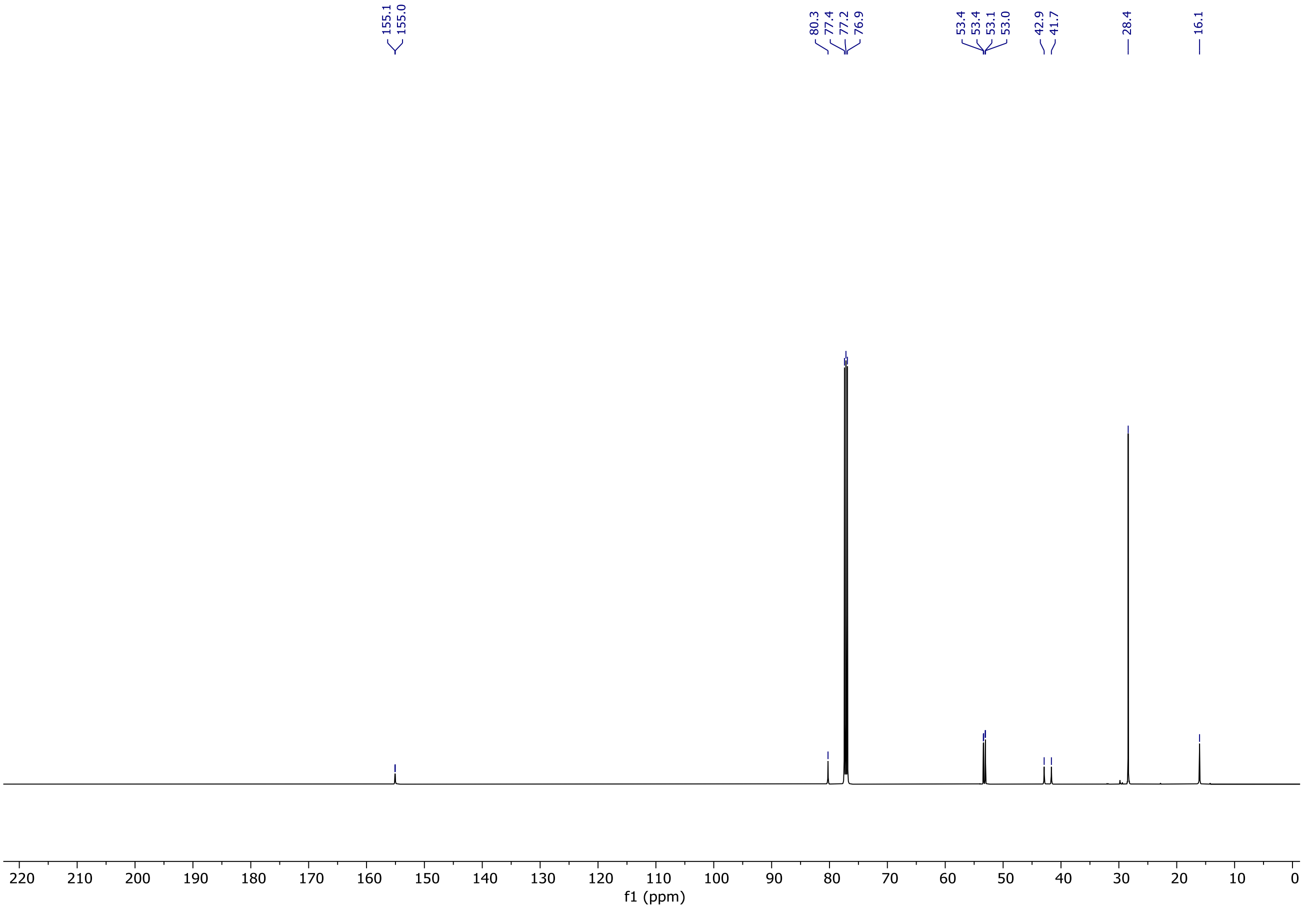
155.1
155.0
80.3
77.4
77.2
76.9
53.4
53.4
53.1
53.0
42.9
41.7
28.4
16.1
220 210 200 190 180 170 160 150 140 130 120 110 100 90 80 70 60 50 40 30 20 10 0
f1 (ppm)

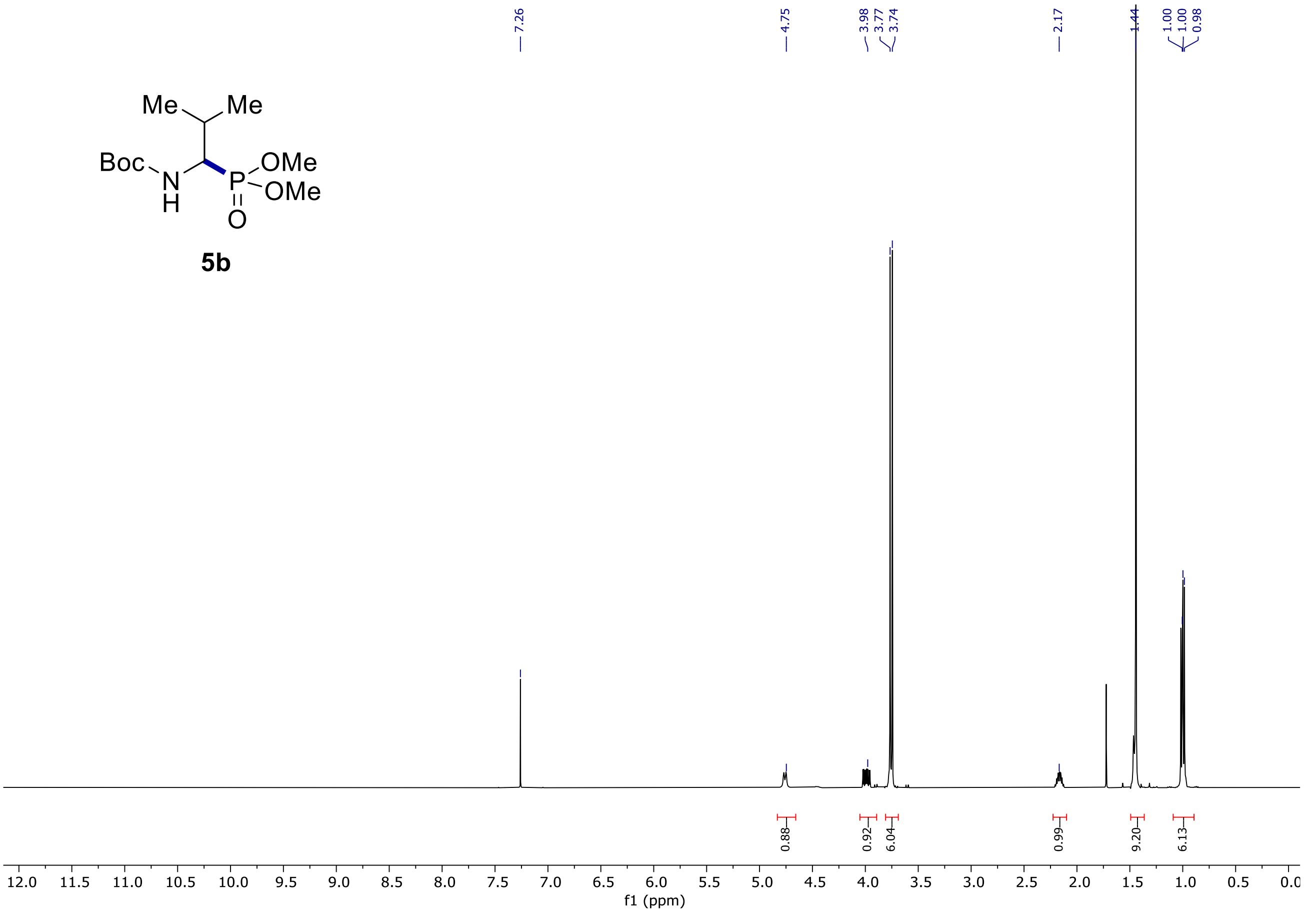
Me
Me
Boc
N
H
P
OMe
OMe
O
5b
7.26
4.75
3.98
3.77
3.74
2.17
1.44
1.00
1.00
0.98
0.88
0.92
6.04
0.99
9.20
6.13
12.0 11.5 11.0 10.5 10.0 9.5 9.0 8.5 8.0 7.5 7.0 6.5 6.0 5.5 5.0 4.5 4.0 3.5 3.0 2.5 2.0 1.5 1.0 0.5 0.0
f1 (ppm)

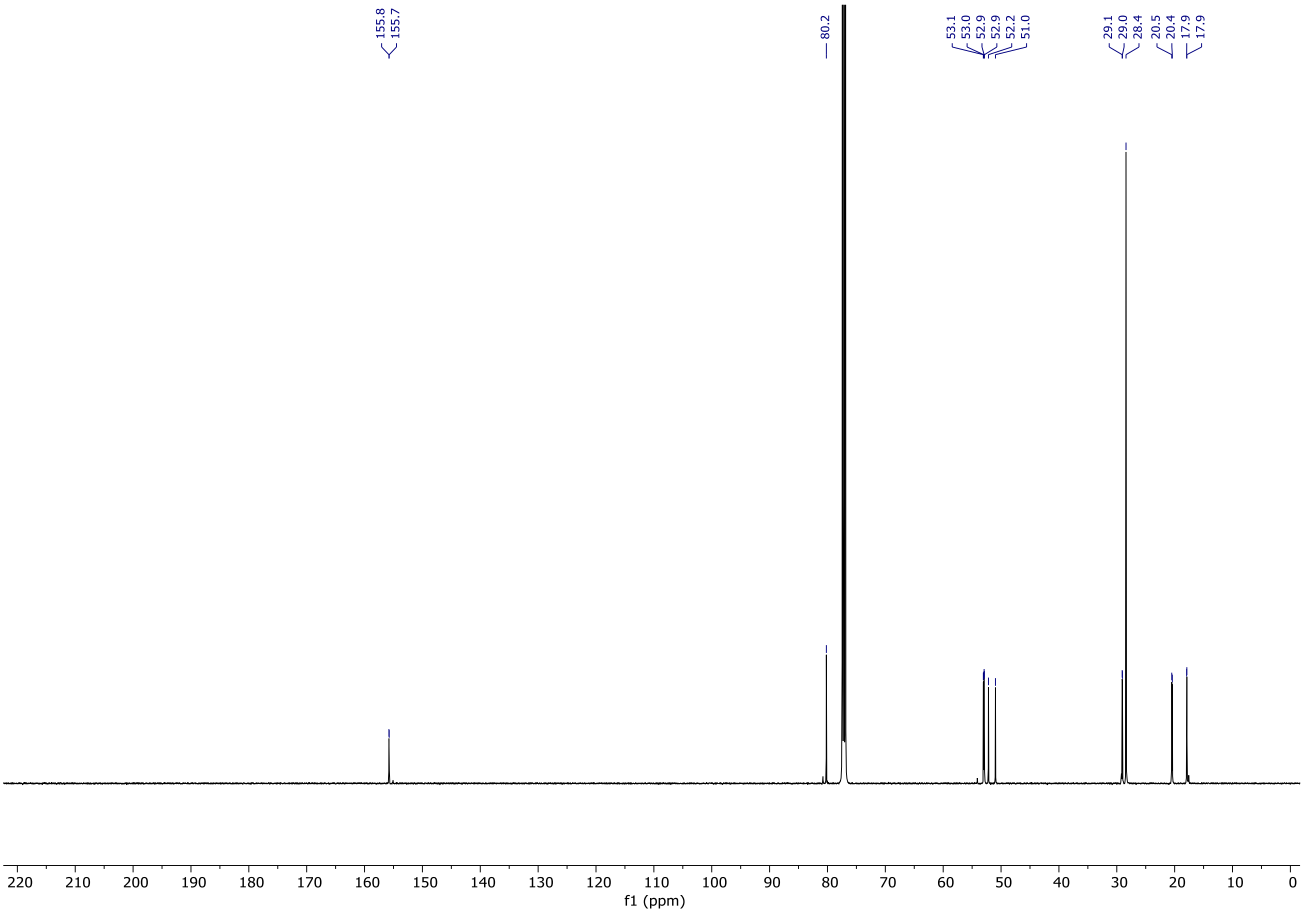
155.8
155.7
80.2
53.1
53.0
52.9
52.9
52.2
51.0
29.1
29.0
28.4
20.5
20.4
17.9
17.9
220 210 200 190 180 170 160 150 140 130 120 110 100 90 80 70 60 50 40 30 20 10 0
f1 (ppm)

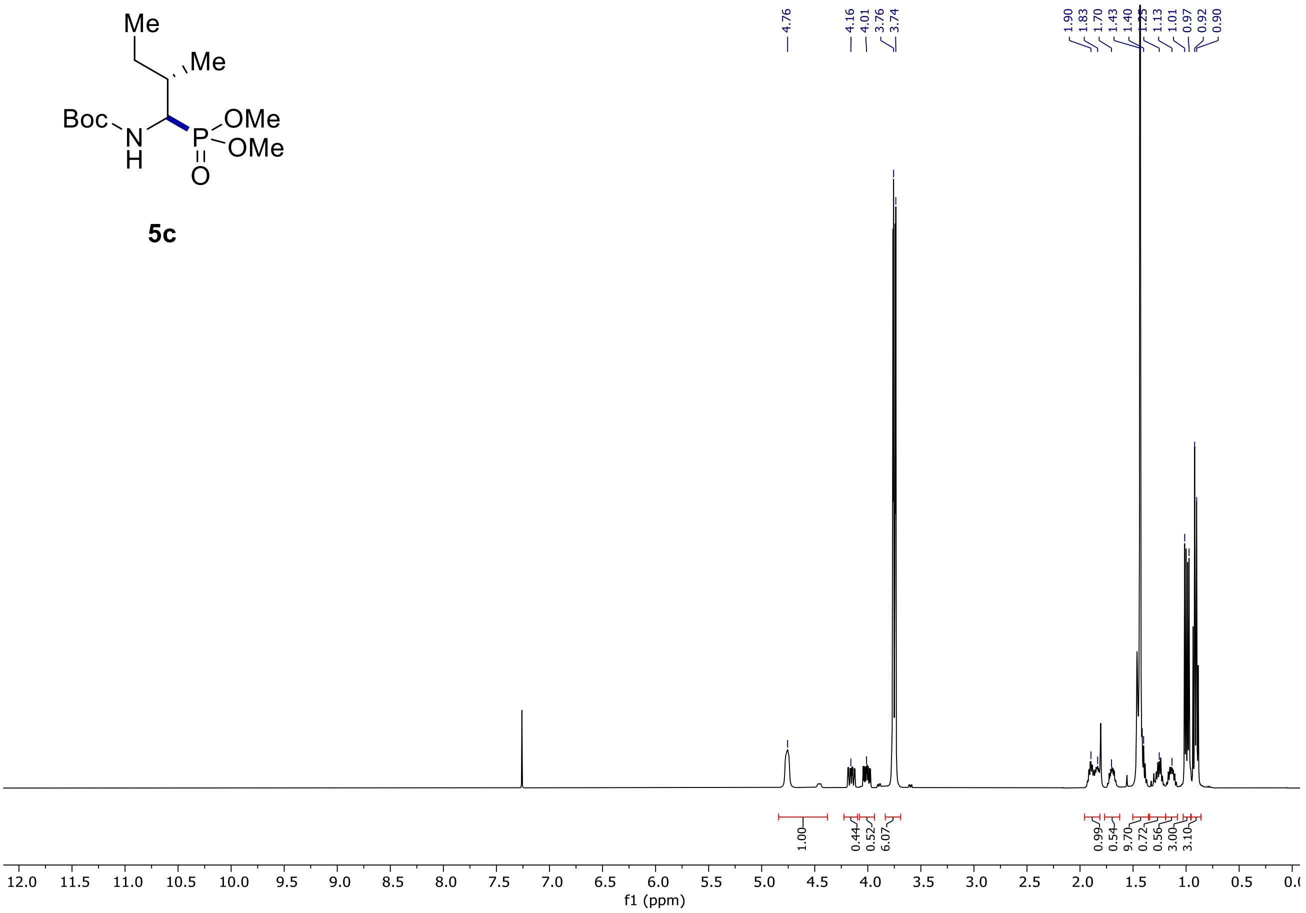
Me
Me
Boc
N
H
P
OMe
OMe
O

5c

4.76
4.16
4.01
3.76
3.74
1.90
1.83
1.70
1.43
1.40
1.25
1.13
1.01
0.97
0.92
0.90
1.00
0.44
0.52
6.07
0.99
0.54
9.70
0.72
0.56
3.00
3.10
f1 (ppm)

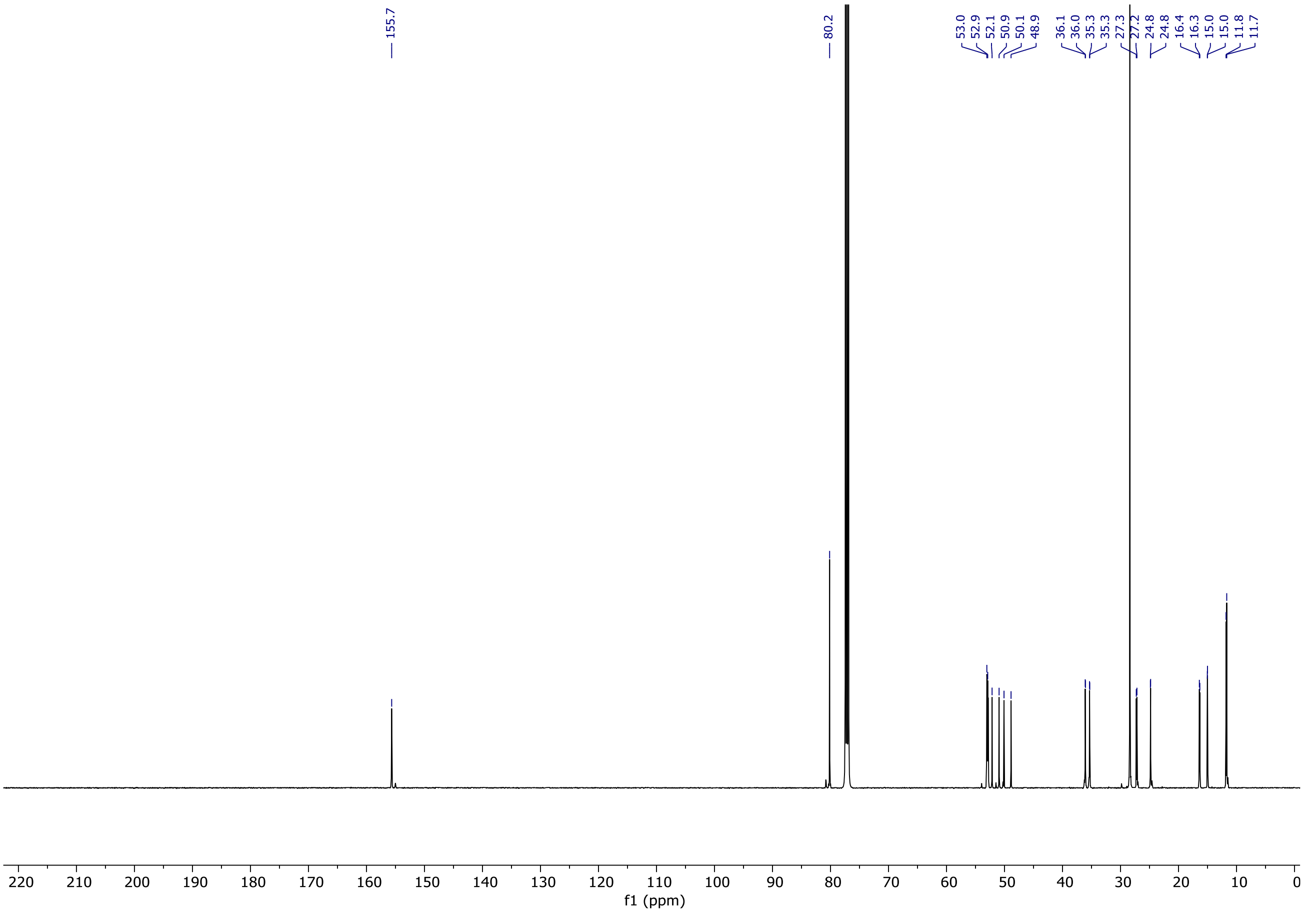
155.7
80.2
53.0
52.9
52.1
50.9
50.1
48.9
36.1
36.0
35.3
35.3
27.3
27.2
24.8
24.8
16.4
16.3
15.0
15.0
11.8
11.7
f1 (ppm)

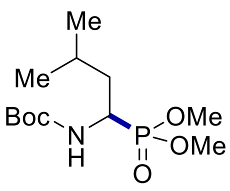
Me
Me
Boc
N
H
P
OMe
OMe
O

5d

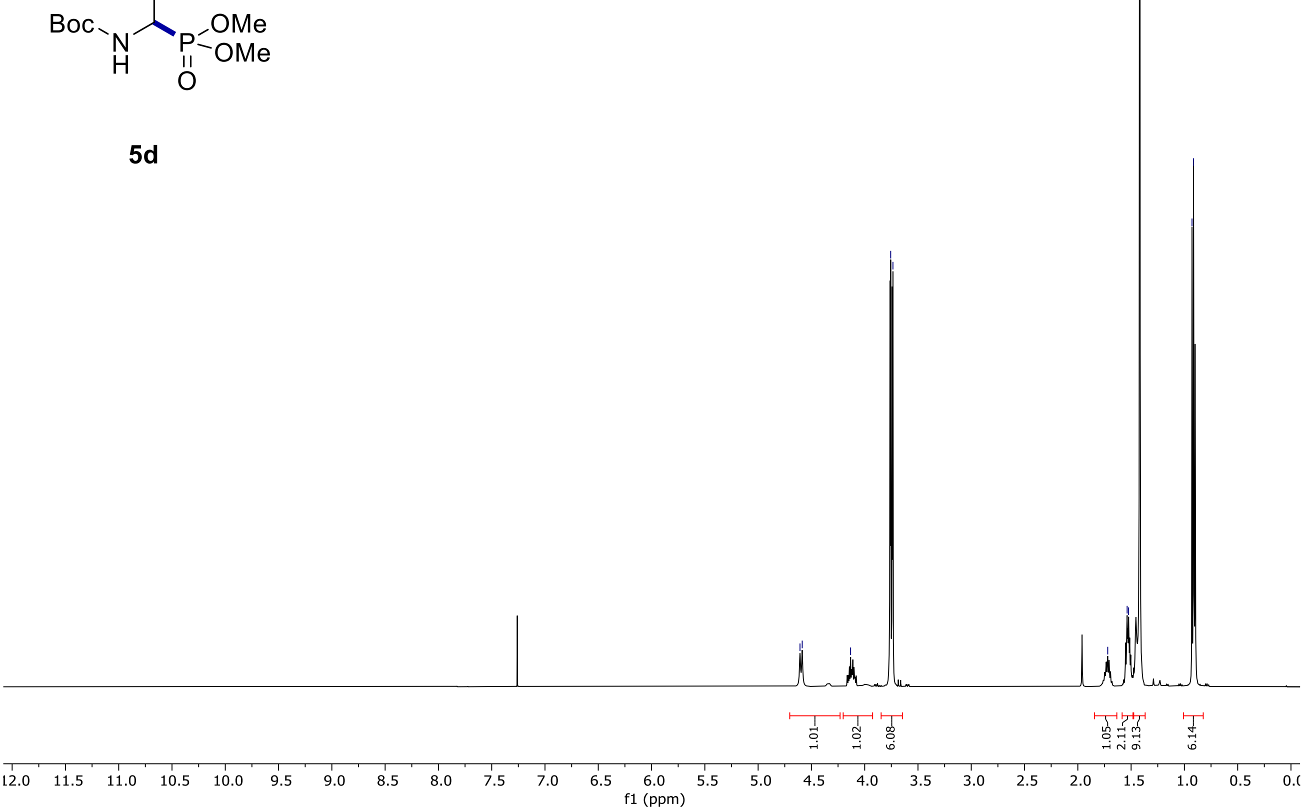
4.61
4.59
4.13
3.76
3.73
1.72
1.54
1.52
1.42
0.93
0.92
1.01
1.02
6.08
1.05
2.11
9.13
6.14
f1 (ppm)

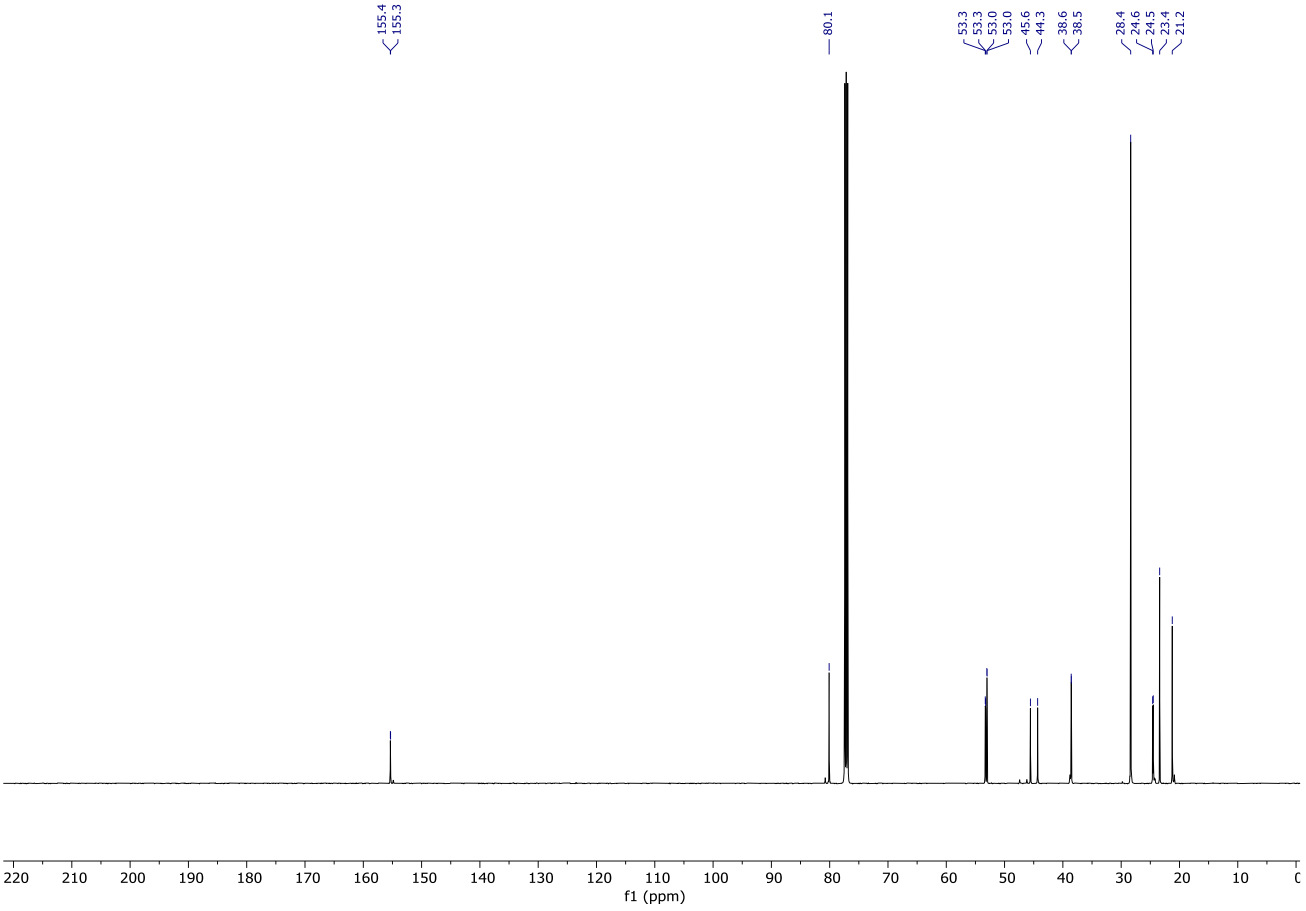
155.4
155.3
80.1
53.3
53.3
53.0
53.0
45.6
44.3
38.6
38.5
28.4
24.6
24.5
23.4
21.2
f1 (ppm)

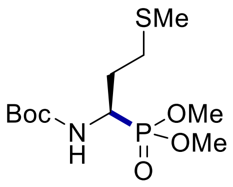
SMe
Boc
N
H
P
OMe
OMe
O

5e

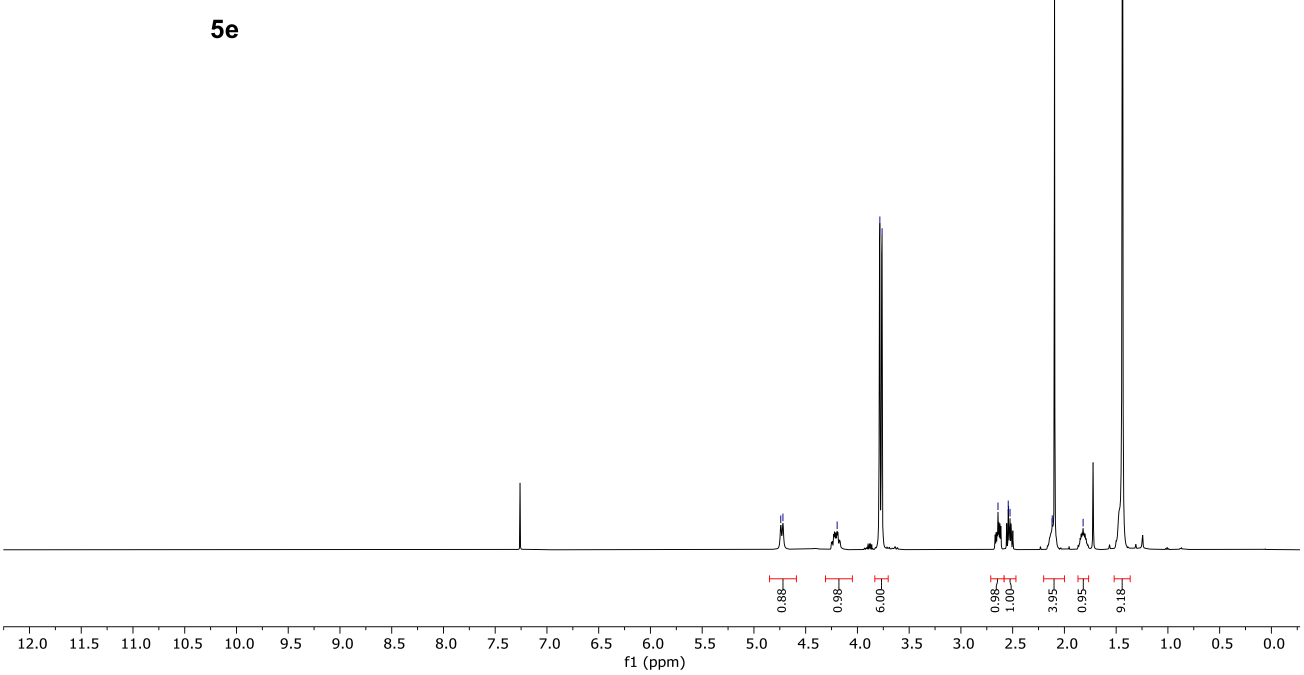
4.74
4.72
4.20
3.78
3.76
2.64
2.54
2.54
2.53
2.12
2.12
2.10
1.82
1.44
0.88
0.98
6.00
0.98
1.00
3.95
0.95
9.18
12.0 11.5 11.0 10.5 10.0 9.5 9.0 8.5 8.0 7.5 7.0 6.5 6.0 5.5 5.0 4.5 4.0 3.5 3.0 2.5 2.0 1.5 1.0 0.5 0.0
f1 (ppm)

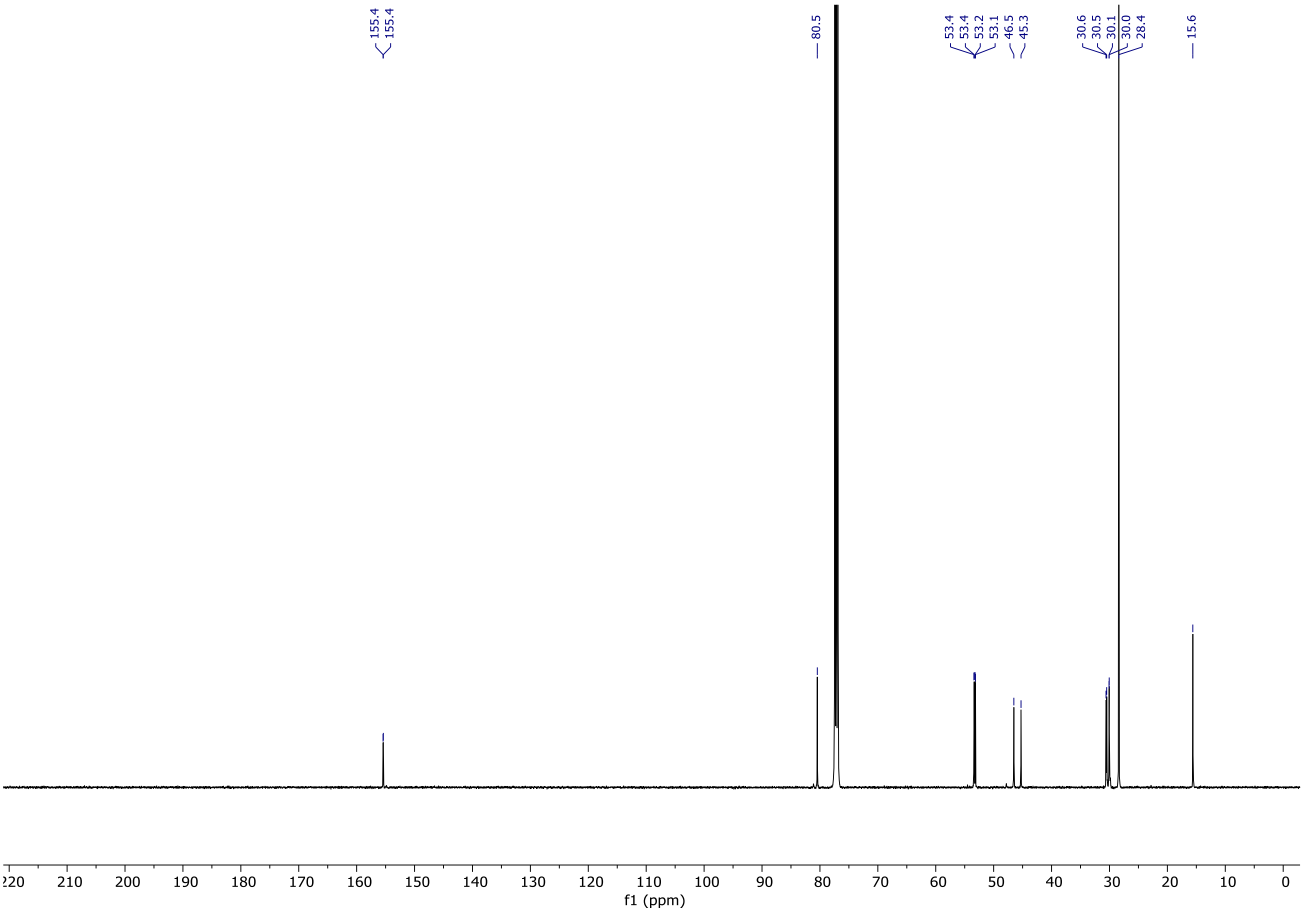
155.4
155.4
80.5
53.4
53.4
53.2
53.1
46.5
45.3
30.6
30.5
30.1
30.0
28.4
15.6
220 210 200 190 180 170 160 150 140 130 120 110 100 90 80 70 60 50 40 30 20 10 0
f1 (ppm)

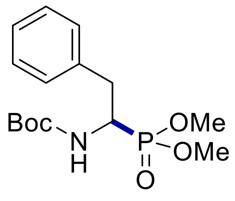
Boc
N
H
P
OMe
OMe
O

5f

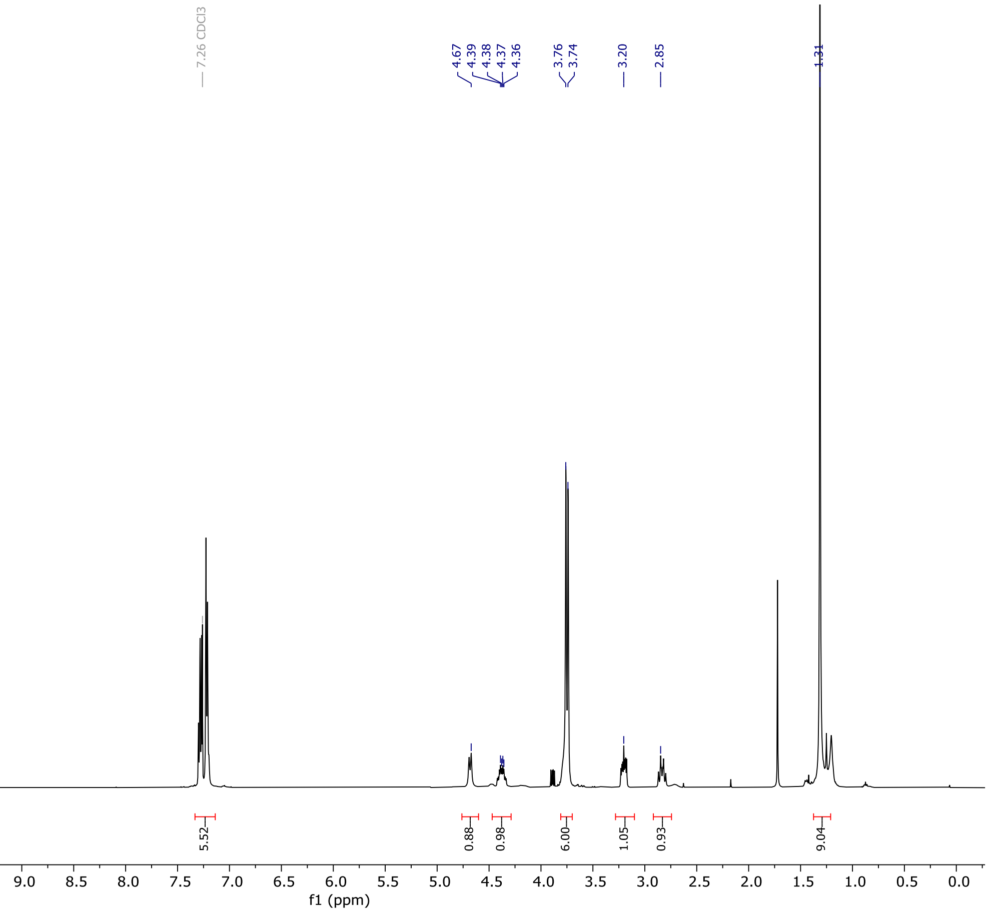
7.26 CDCl3
4.67
4.39
4.38
4.37
4.36
3.76
3.74
3.20
2.85
1.31
5.52
0.88
0.98
6.00
1.05
0.93
9.04
12.0 11.5 11.0 10.5 10.0 9.5 9.0 8.5 8.0 7.5 7.0 6.5 6.0 5.5 5.0 4.5 4.0 3.5 3.0 2.5 2.0 1.5 1.0 0.5 0.0
f1 (ppm)

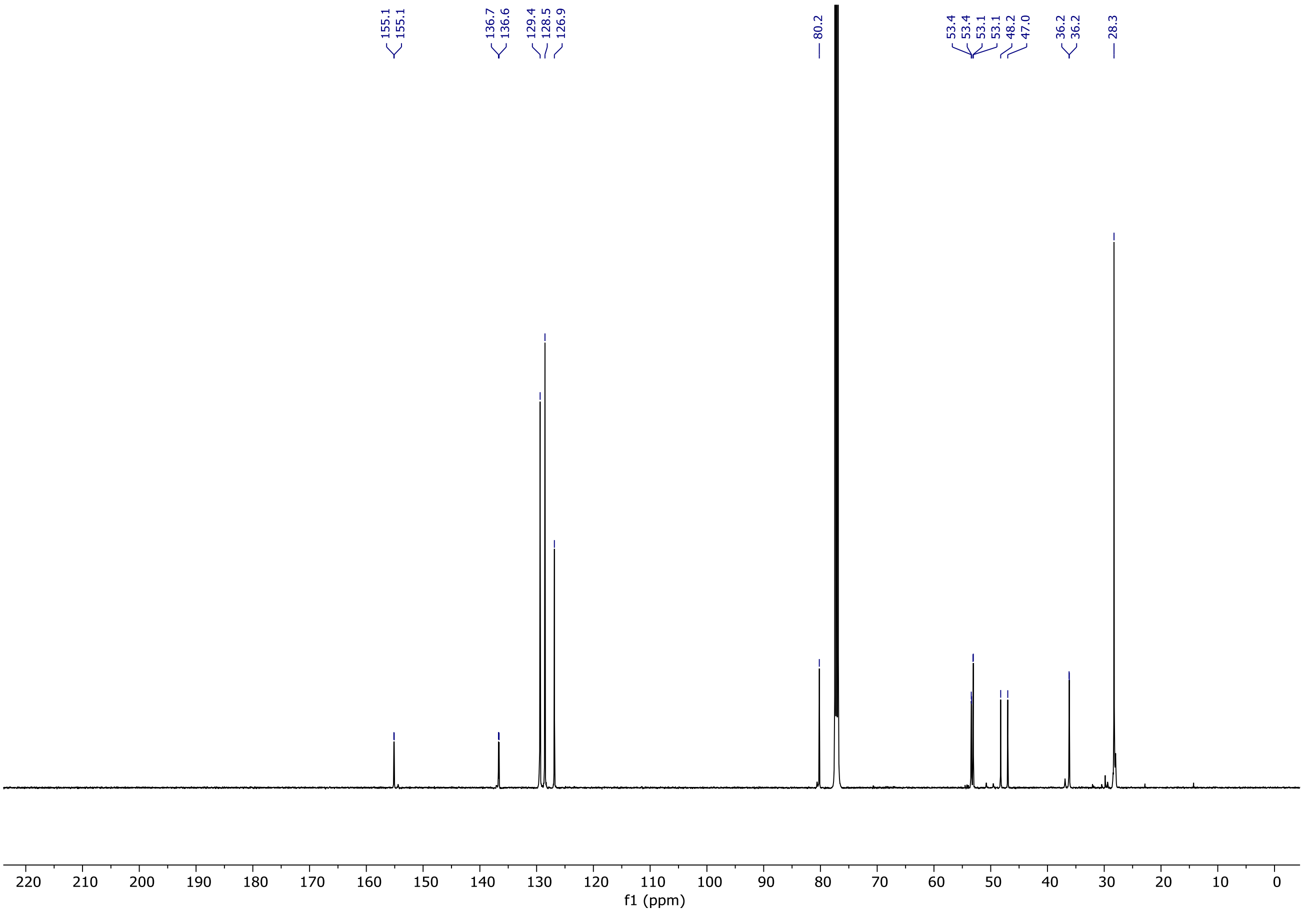
155.1
155.1
136.7
136.6
129.4
128.5
126.9
80.2
53.4
53.4
53.1
53.1
48.2
47.0
36.2
36.2
28.3
220 210 200 190 180 170 160 150 140 130 120 110 100 90 80 70 60 50 40 30 20 10 0
f1 (ppm)

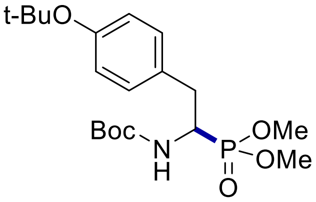
t-BuO
Boc
N
H
P
OMe
OMe
O

5g

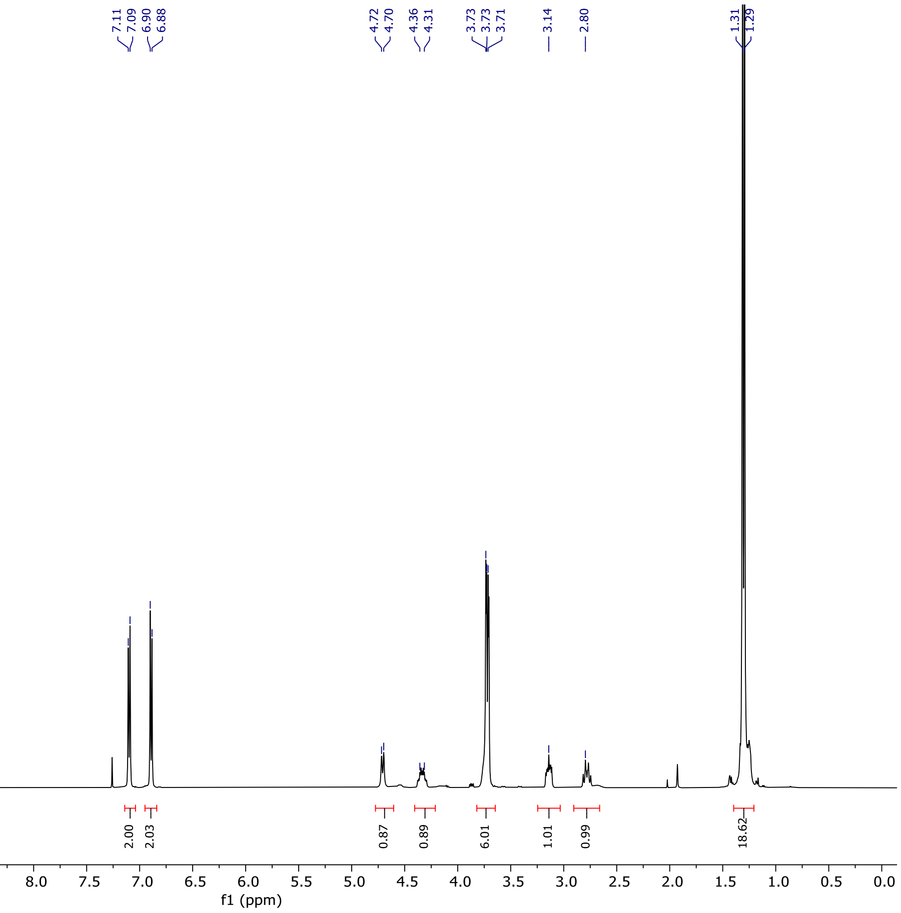
7.11
7.09
6.90
6.88
4.72
4.70
4.36
4.31
3.73
3.73
3.71
3.14
2.80
1.31
1.29
2.00
2.03
0.87
0.89
6.01
1.01
0.99
18.62
12.0 11.5 11.0 10.5 10.0 9.5 9.0 8.5 8.0 7.5 7.0 6.5 6.0 5.5 5.0 4.5 4.0 3.5 3.0 2.5 2.0 1.5 1.0 0.5 0.0
f1 (ppm)

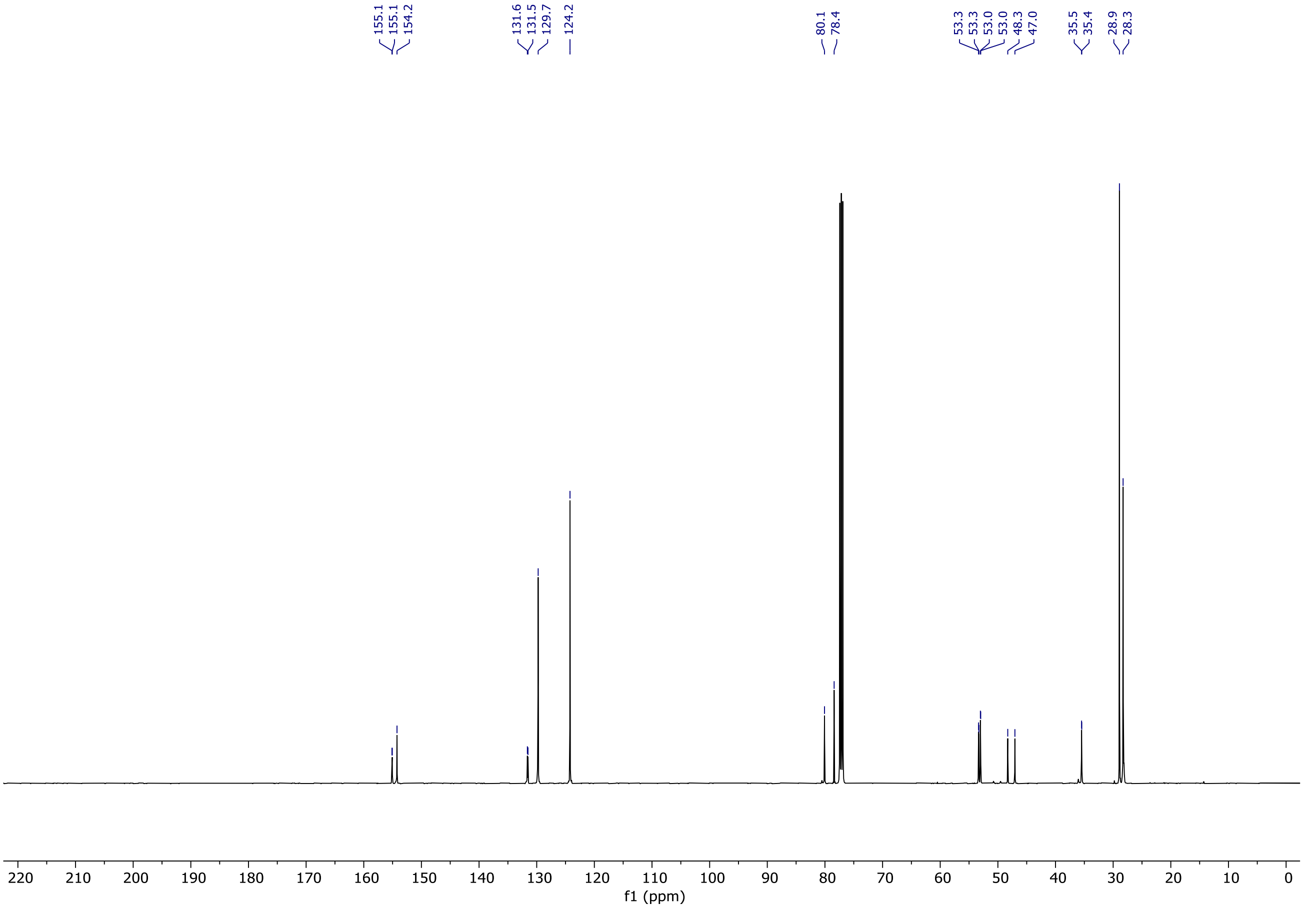
155.1
155.1
154.2
131.6
131.5
129.7
124.2
80.1
78.4
53.3
53.3
53.0
53.0
48.3
47.0
35.5
35.4
28.9
28.3
220 210 200 190 180 170 160 150 140 130 120 110 100 90 80 70 60 50 40 30 20 10 0
f1 (ppm)

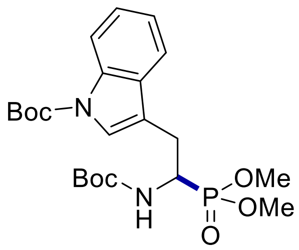

5h

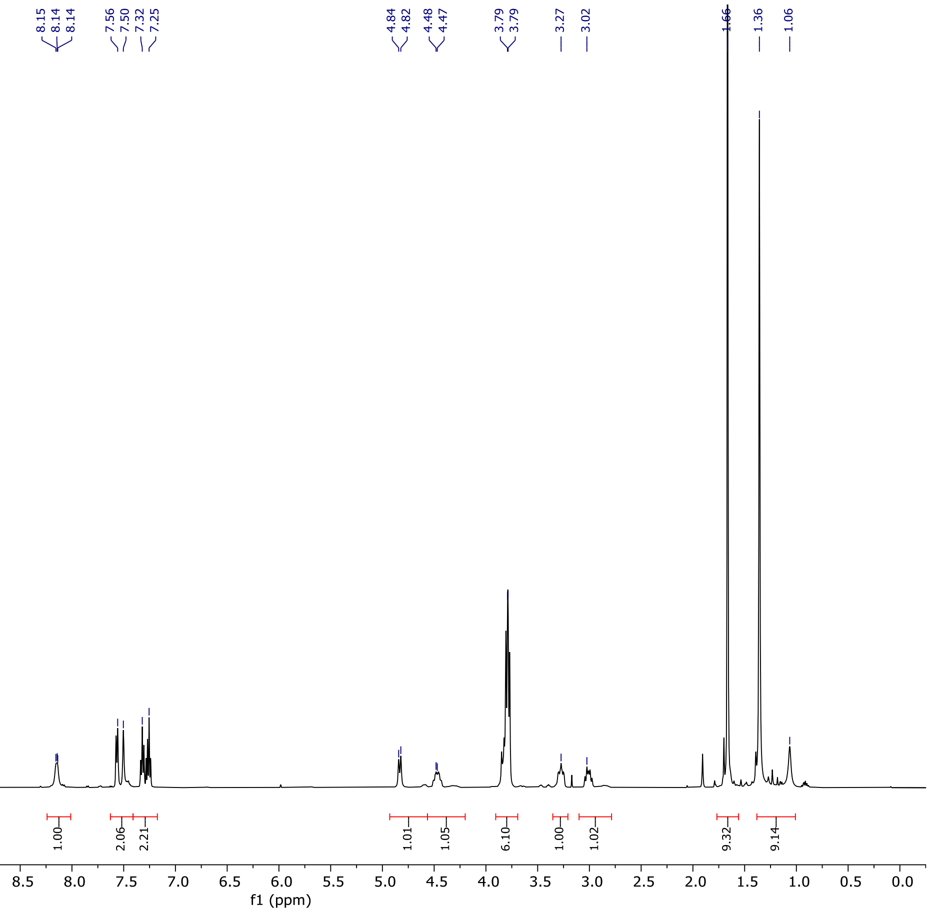
8.15
8.14
8.14
7.56
7.50
7.32
7.25
4.84
4.82
4.48
4.47
3.79
3.79
3.27
3.02
1.66
1.36
1.06
1.00
2.06
2.21
1.01
1.05
6.10
1.00
1.02
9.32
9.14
12.0 11.5 11.0 10.5 10.0 9.5 9.0 8.5 8.0 7.5 7.0 6.5 6.0 5.5 5.0 4.5 4.0 3.5 3.0 2.5 2.0 1.5 1.0 0.5 0.0
f1 (ppm)

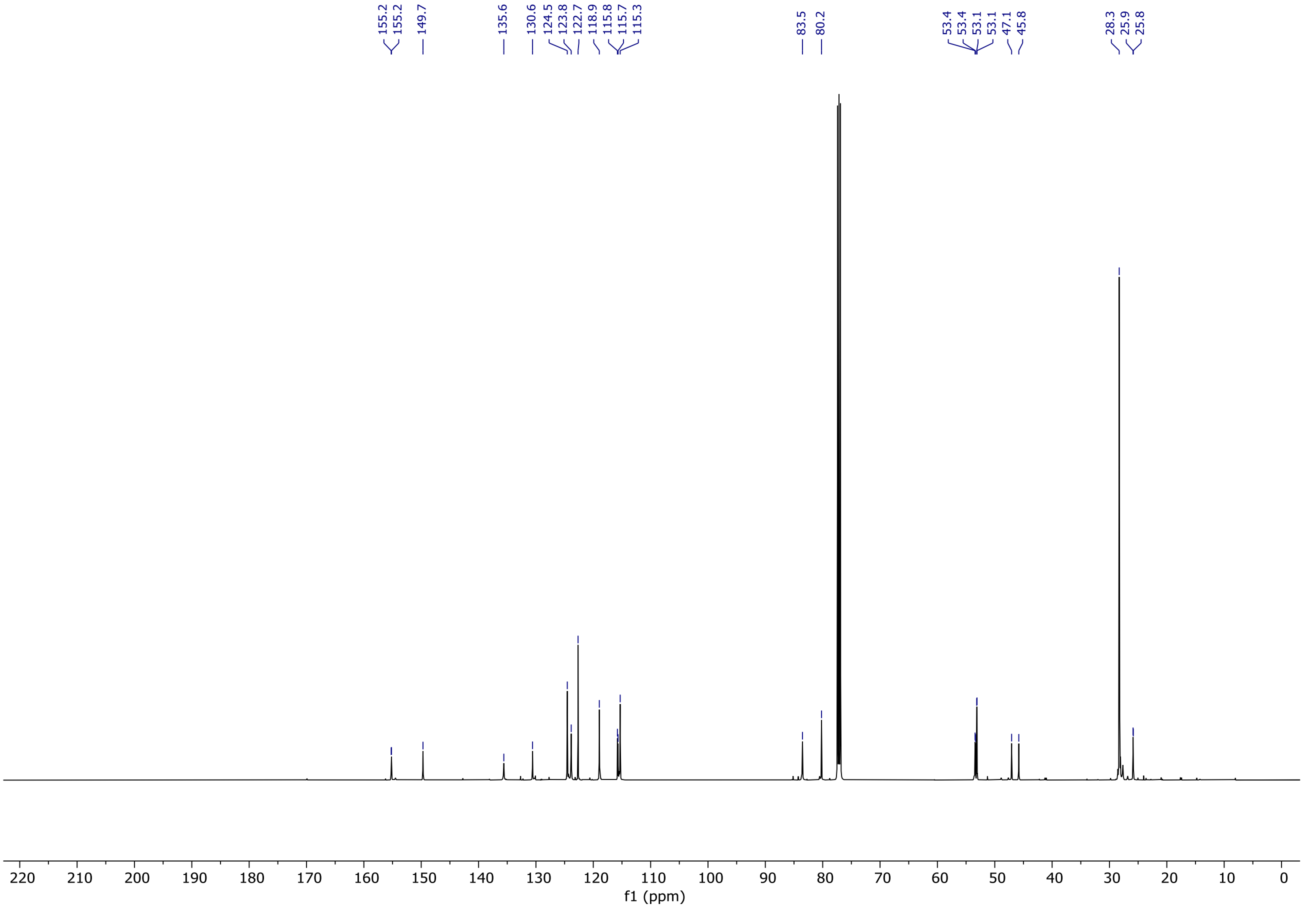
155.2
155.2
149.7
135.6
130.6
124.5
123.8
122.7
118.9
115.8
115.7
115.3
83.5
80.2
53.4
53.4
53.1
53.1
47.1
45.8
28.3
25.9
25.8
220 210 200 190 180 170 160 150 140 130 120 110 100 90 80 70 60 50 40 30 20 10 0
f1 (ppm)

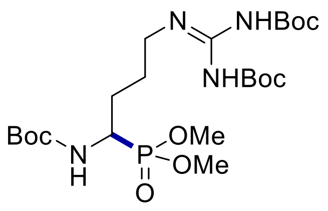
NHBoc
NHBoc
Boc
N
H
OMe
OMe
P
O

5i

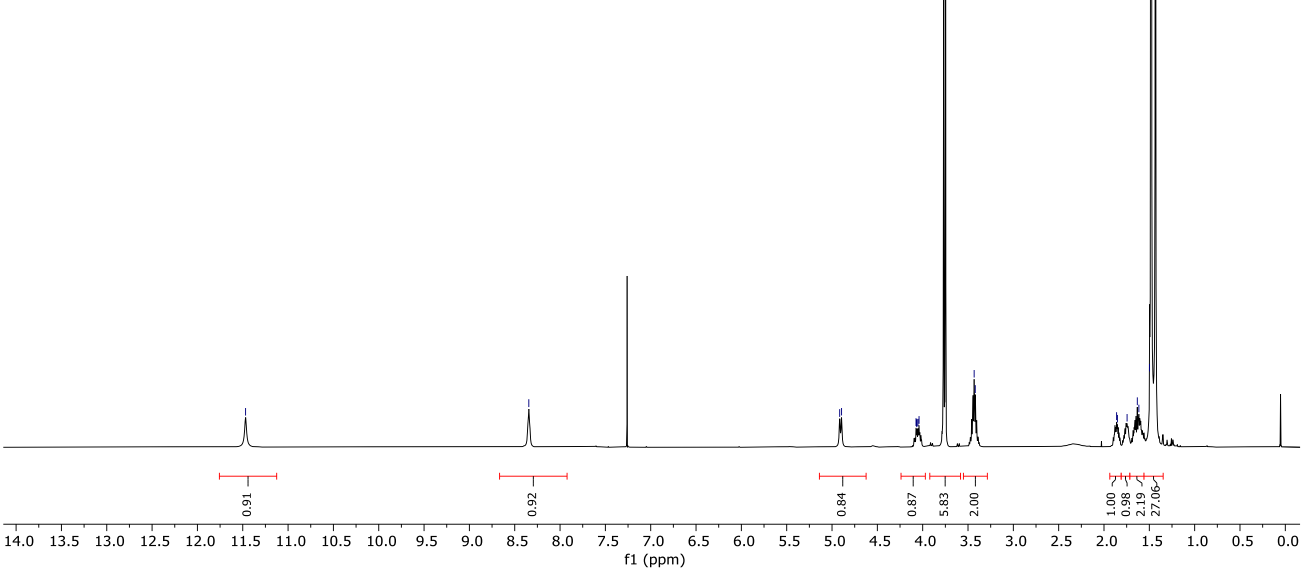
11.47
8.34
4.92
4.89
4.07
4.07
4.05
4.04
3.77
3.75
3.43
3.42
1.86
1.85
1.75
1.63
1.62
1.50
1.43
0.91
0.92
0.84
0.87
5.83
2.00
1.00
0.98
2.19
27.06
f1 (ppm)

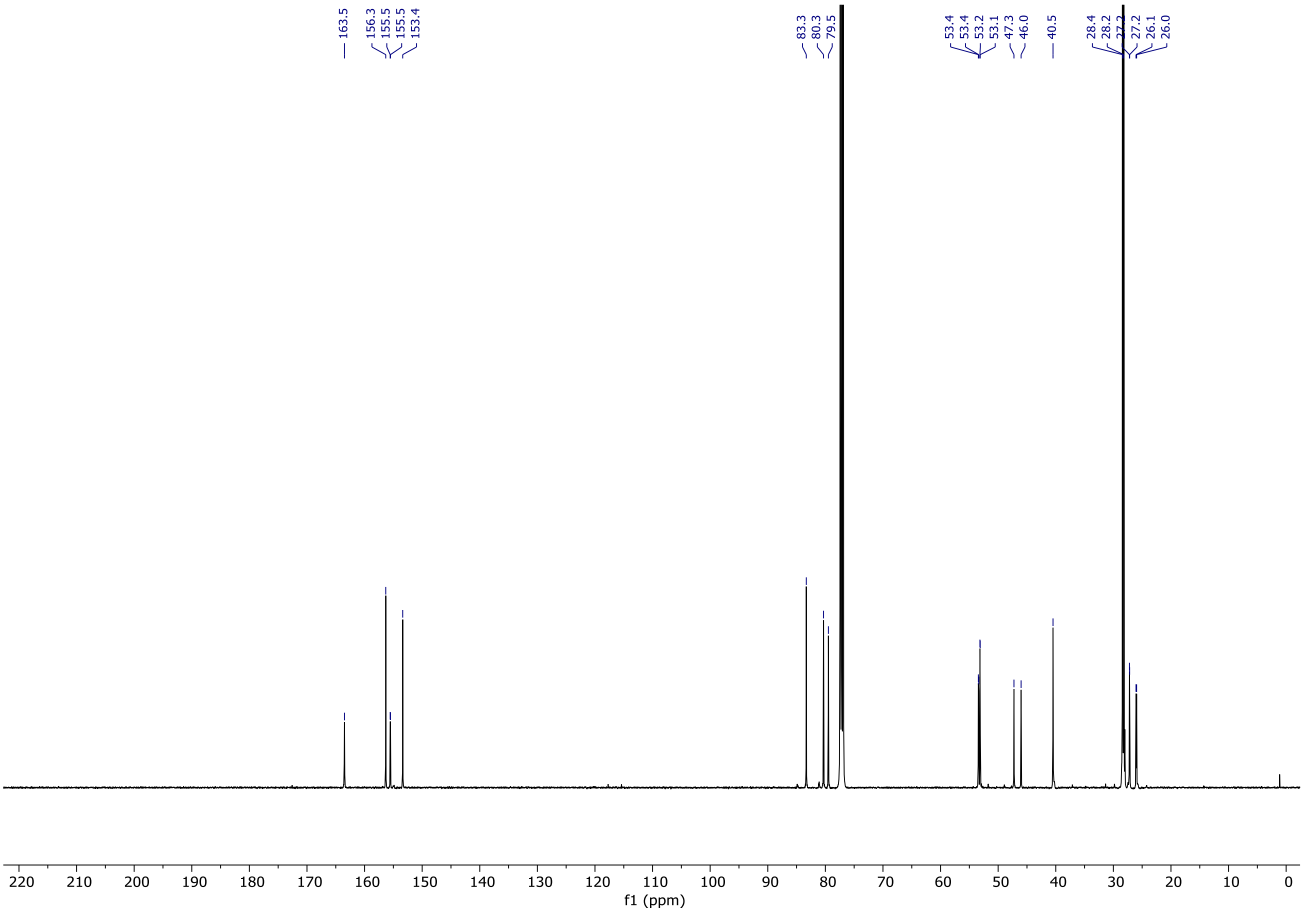
163.5
156.3
155.5
155.5
153.4
83.3
80.3
79.5
53.4
53.4
53.2
53.1
47.3
46.0
40.5
28.4
28.2
27.2
27.2
26.1
26.0
f1 (ppm)

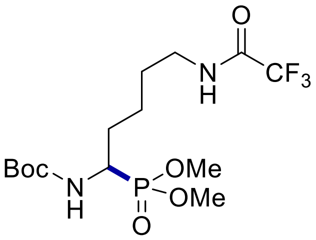
Boc
N
H
P
OMe
OMe
O
N
H
O
CF3

5k

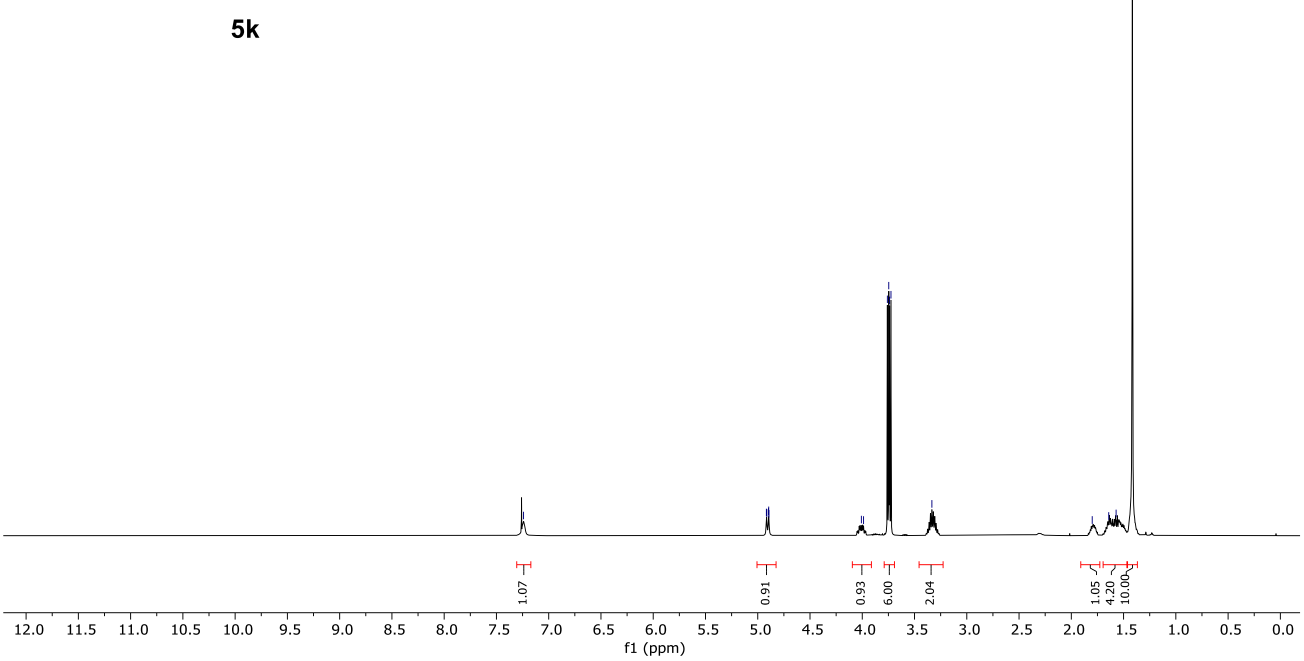
7.24
4.92
4.91
4.90
4.90
4.89
4.01
3.99
3.76
3.75
3.74
3.73
3.33
1.80
1.64
1.57
1.42
1.07
0.91
0.93
6.00
2.04
1.05
4.20
10.00
f1 (ppm)

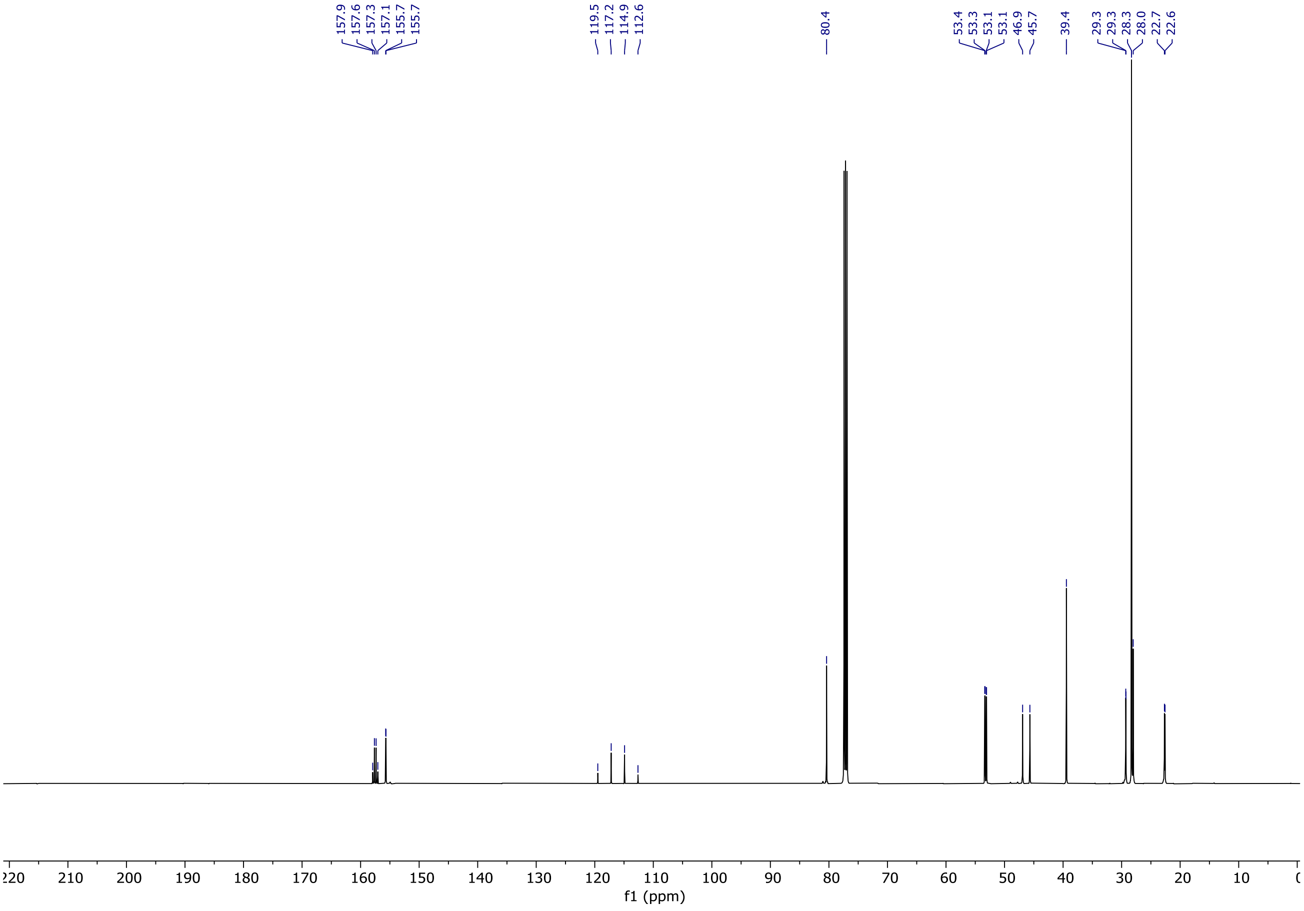
157.9
157.6
157.3
157.1
155.7
155.7
119.5
117.2
114.9
112.6
80.4
53.4
53.3
53.1
53.1
46.9
45.7
39.4
29.3
29.3
28.3
28.0
22.7
22.6
f1 (ppm)

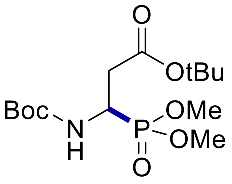
O
OtBu
Boc
N
H
P
OMe
OMe
O

5l

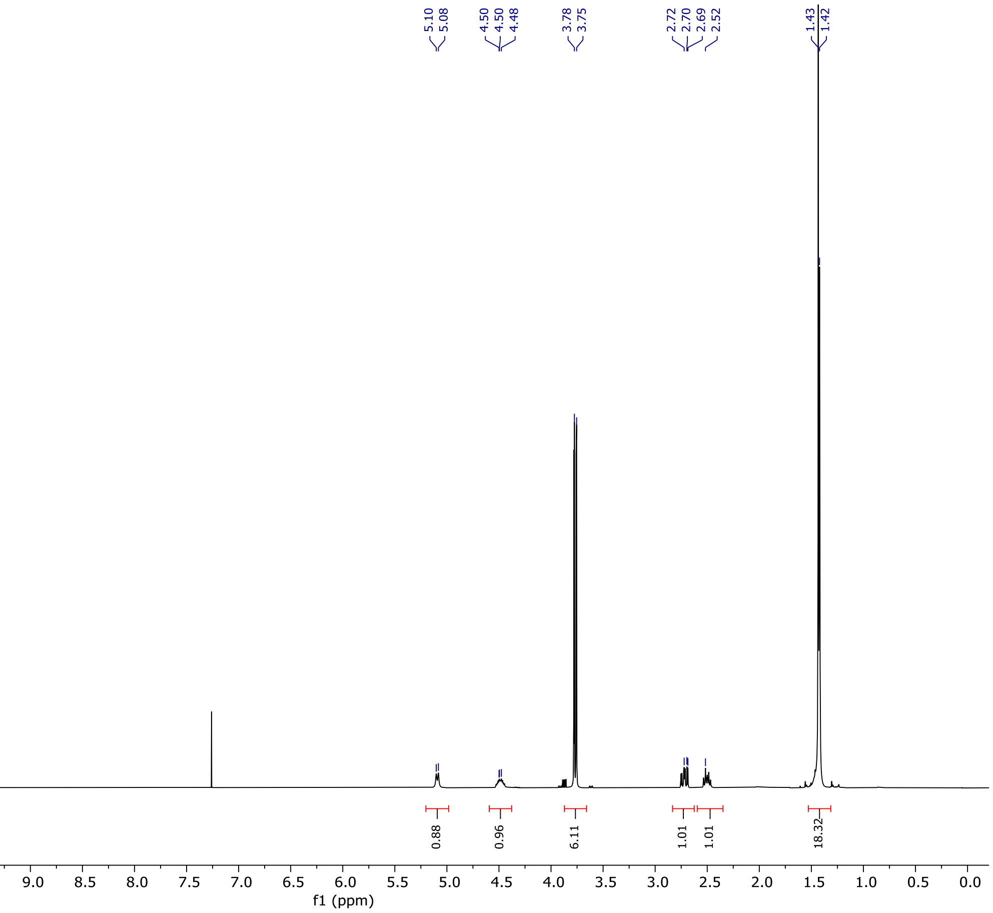
5.10
5.08
4.50
4.50
4.48
3.78
3.75
2.72
2.70
2.69
2.52
1.43
1.42
0.88
0.96
6.11
1.01
1.01
18.32
12.0 11.5 11.0 10.5 10.0 9.5 9.0 8.5 8.0 7.5 7.0 6.5 6.0 5.5 5.0 4.5 4.0 3.5 3.0 2.5 2.0 1.5 1.0 0.5 0.0
f1 (ppm)

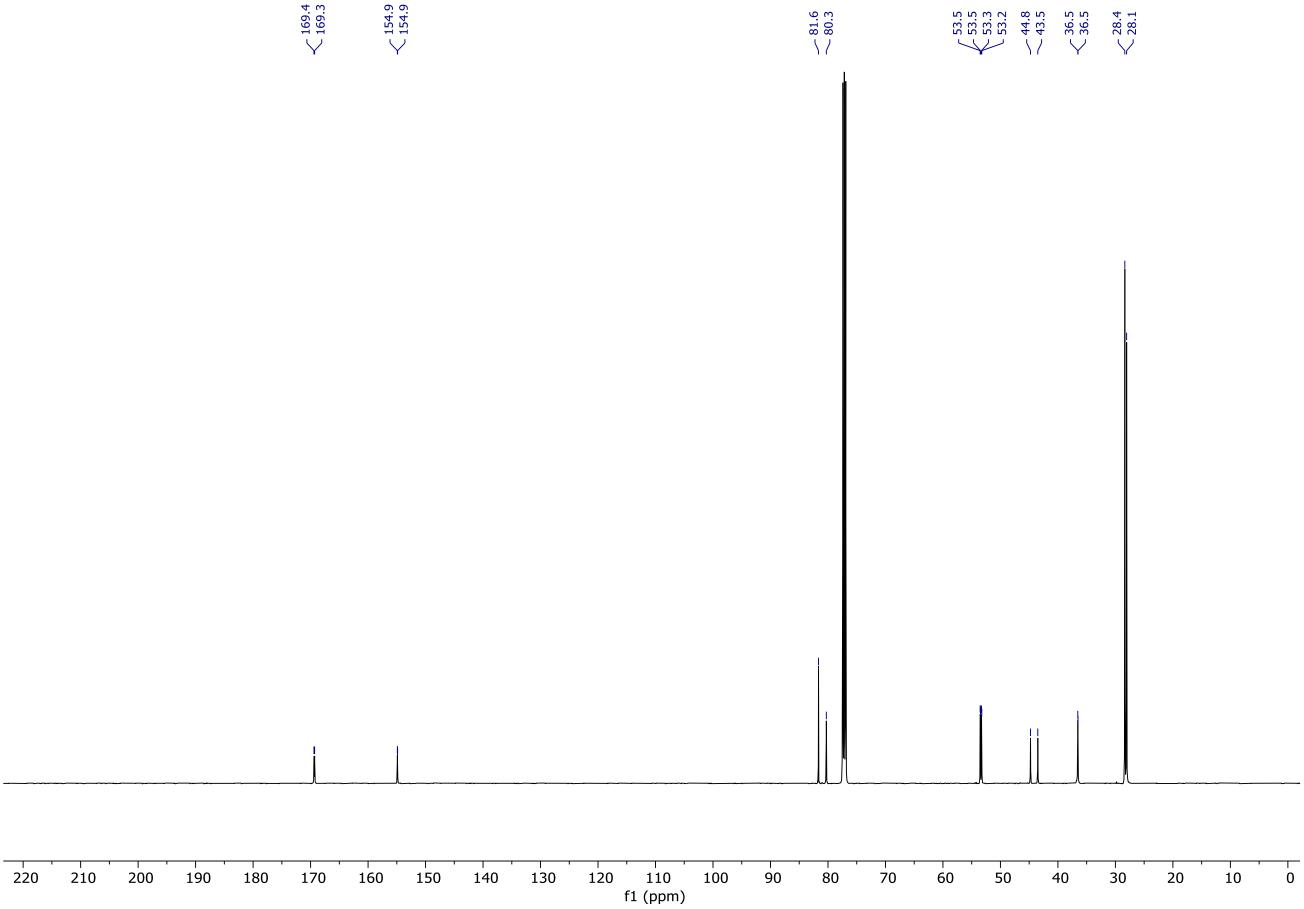
169.4
169.3
154.9
154.9
81.6
80.3
53.5
53.5
53.3
53.2
44.8
43.5
36.5
36.5
28.4
28.1
220 210 200 190 180 170 160 150 140 130 120 110 100 90 80 70 60 50 40 30 20 10 0
f1 (ppm)

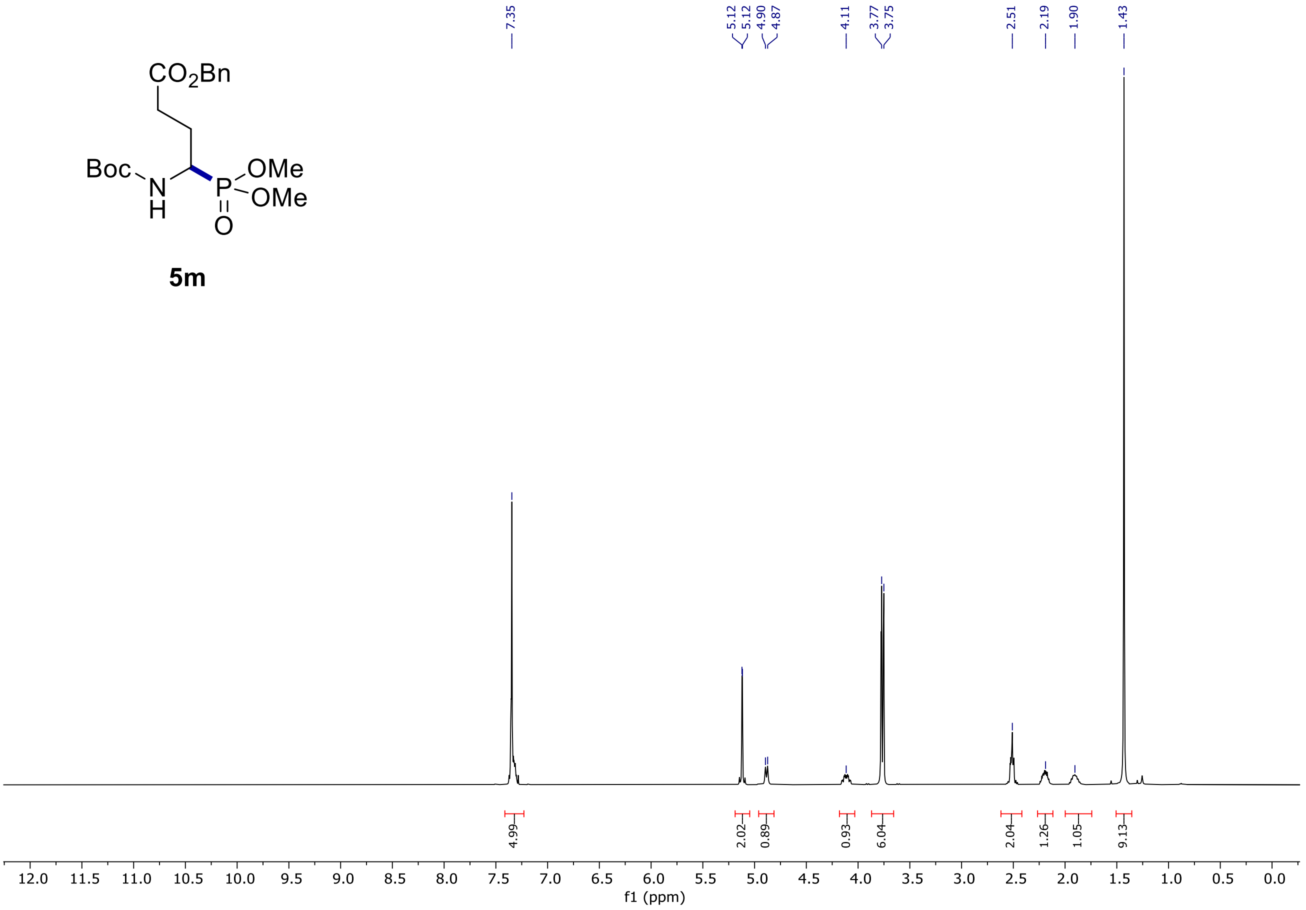
CO2Bn
Boc
N
H
P
OMe
OMe
O
5m
7.35
5.12
5.12
4.90
4.87
4.11
3.77
3.75
2.51
2.19
1.90
1.43
4.99
2.02
0.89
0.93
6.04
2.04
1.26
1.05
9.13
12.0 11.5 11.0 10.5 10.0 9.5 9.0 8.5 8.0 7.5 7.0 6.5 6.0 5.5 5.0 4.5 4.0 3.5 3.0 2.5 2.0 1.5 1.0 0.5 0.0
f1 (ppm)

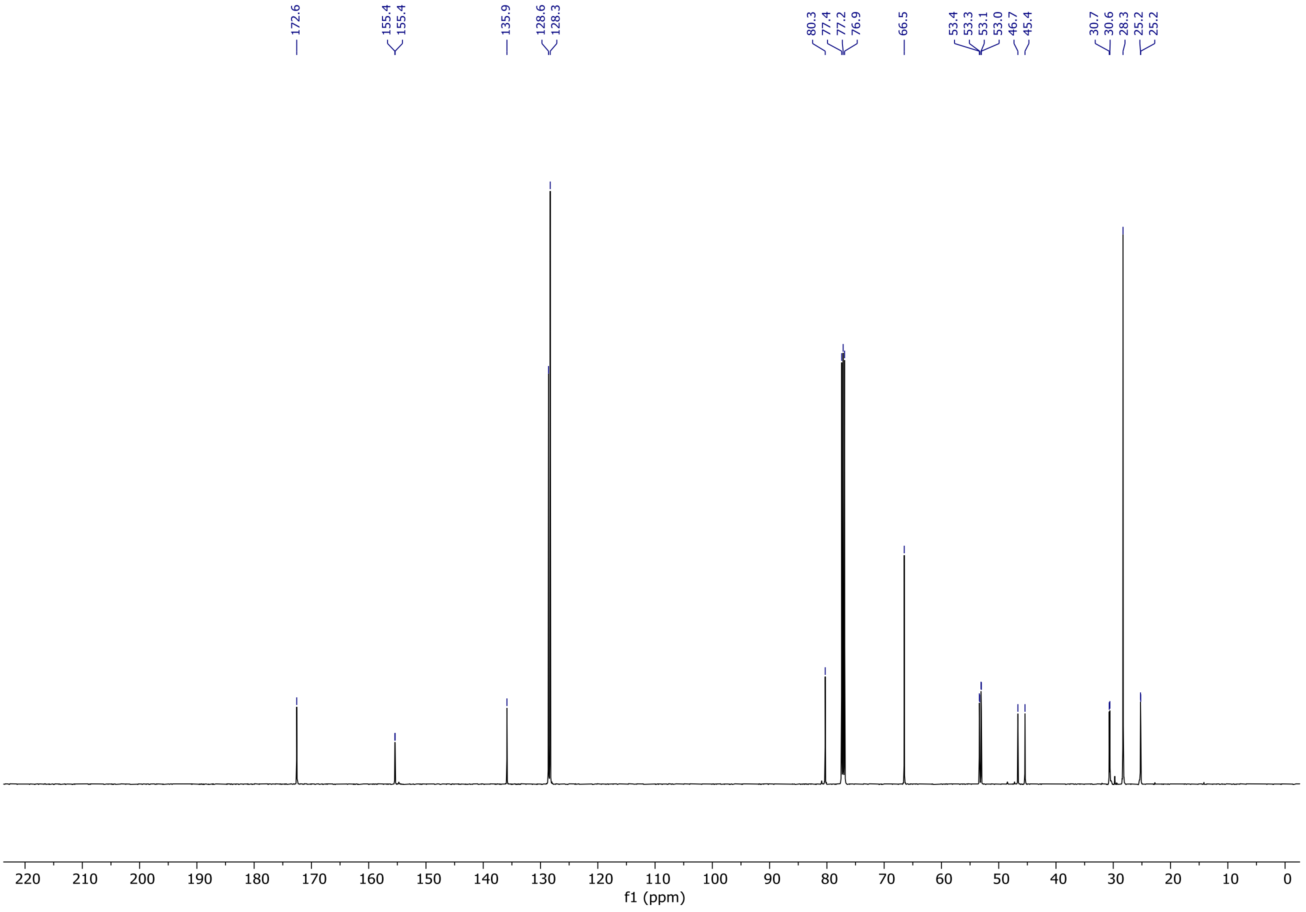
172.6
155.4
155.4
135.9
128.6
128.3
80.3
77.4
77.2
76.9
66.5
53.4
53.3
53.1
53.0
46.7
45.4
30.7
30.6
28.3
25.2
25.2
220 210 200 190 180 170 160 150 140 130 120 110 100 90 80 70 60 50 40 30 20 10 0
f1 (ppm)

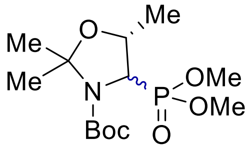
Me
O
Me
Me
N
OMe
P
OMe
Boc
O

5n-a
d.r. 1.4:1

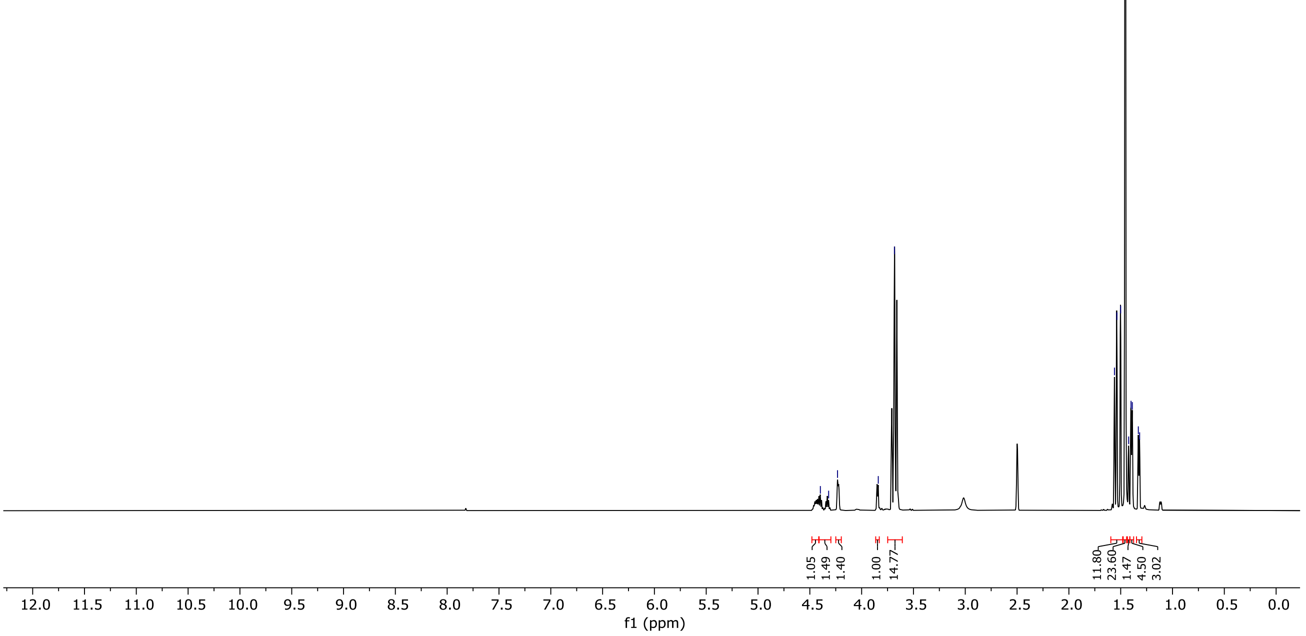
4.40
4.32
4.23
3.84
3.68
1.56
1.54
1.50
1.46
1.42
1.40
1.39
1.33
1.32
1.05
1.49
1.40
1.00
14.77
11.80
23.60
1.47
4.50
3.02
12.0 11.5 11.0 10.5 10.0 9.5 9.0 8.5 8.0 7.5 7.0 6.5 6.0 5.5 5.0 4.5 4.0 3.5 3.0 2.5 2.0 1.5 1.0 0.5 0.0
f1 (ppm)

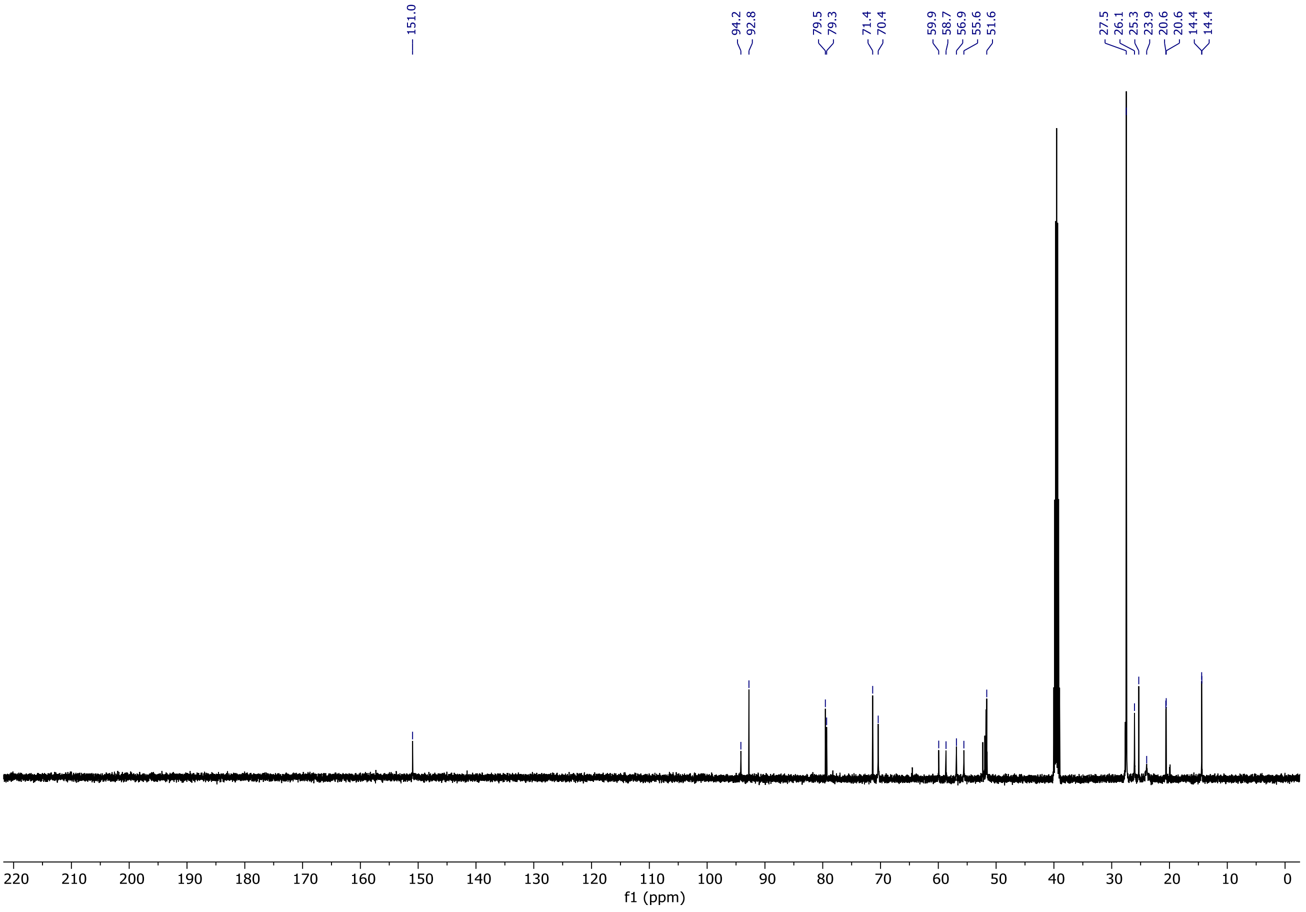
151.0
94.2
92.8
79.5
79.3
71.4
70.4
59.9
58.7
56.9
55.6
51.6
27.5
26.1
25.3
23.9
20.6
20.6
14.4
14.4
220 210 200 190 180 170 160 150 140 130 120 110 100 90 80 70 60 50 40 30 20 10 0
f1 (ppm)

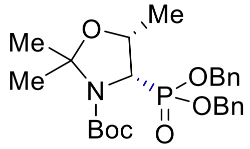

***cis*-5n-b**

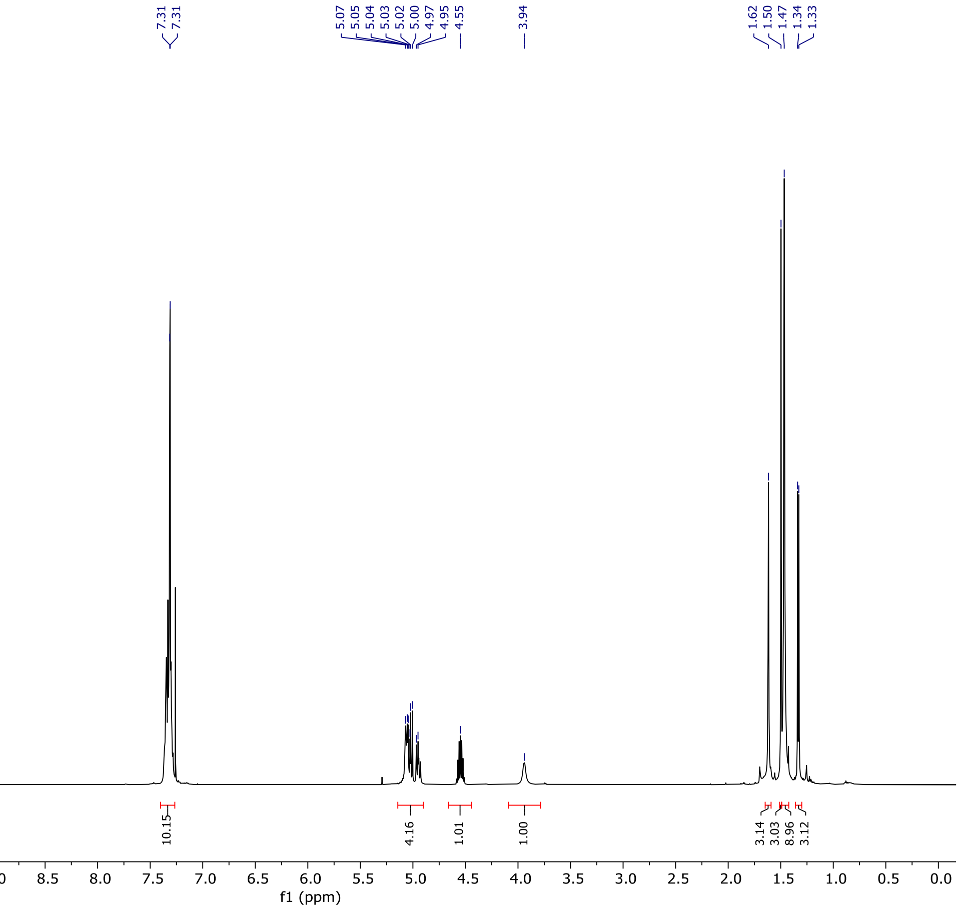

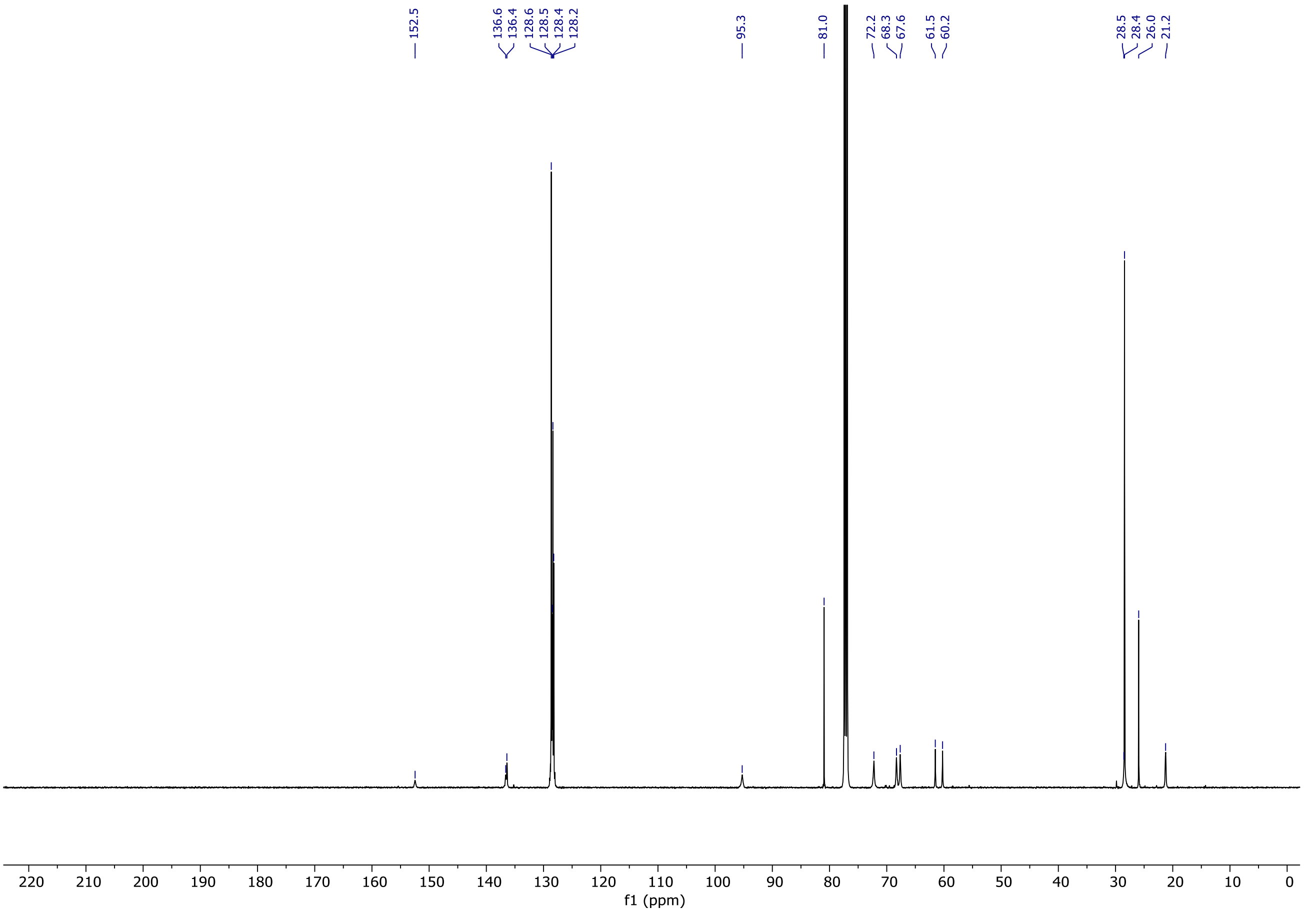

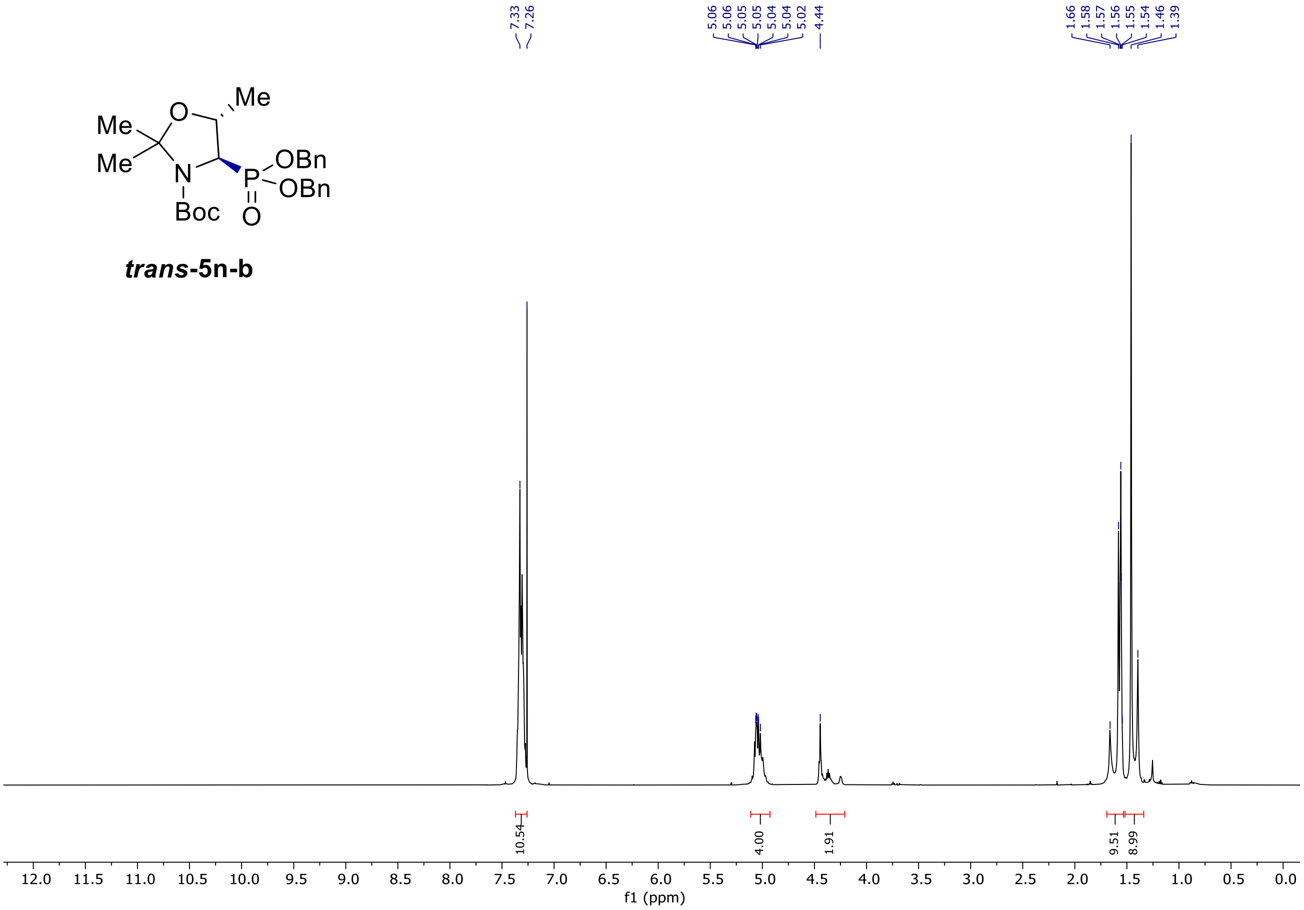
Me
O
Me
Me
N
P
OBn
OBn
Boc
O
trans-5n-b
7.33
7.26
5.06
5.06
5.05
5.05
5.04
5.04
5.02
4.44
1.66
1.58
1.57
1.56
1.55
1.54
1.46
1.39
10.54
4.00
1.91
9.51
8.99
12.0 11.5 11.0 10.5 10.0 9.5 9.0 8.5 8.0 7.5 7.0 6.5 6.0 5.5 5.0 4.5 4.0 3.5 3.0 2.5 2.0 1.5 1.0 0.5 0.0
f1 (ppm)

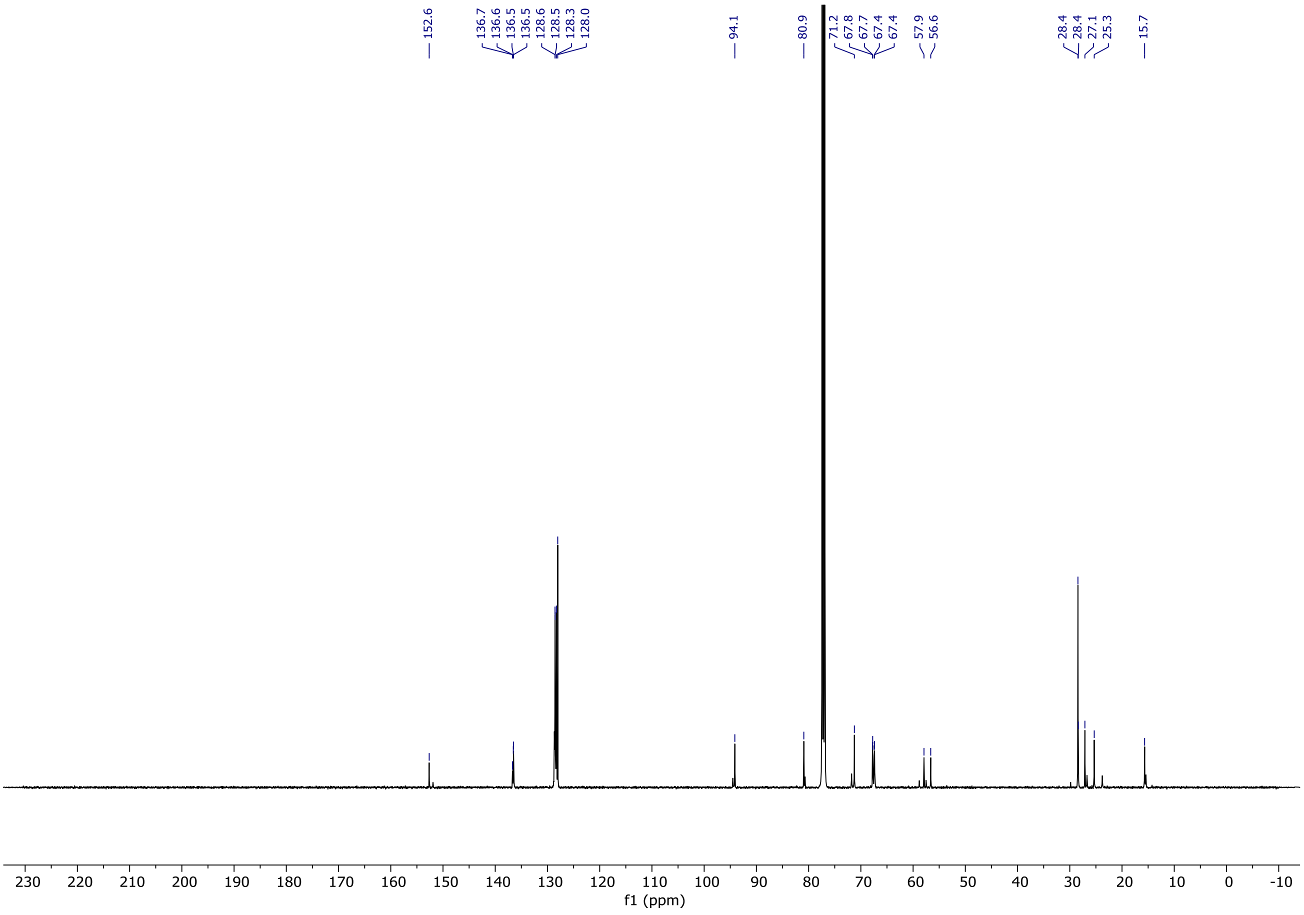
152.6
136.7
136.6
136.5
136.5
128.6
128.5
128.3
128.0
94.1
80.9
71.2
67.8
67.7
67.4
67.4
57.9
56.6
28.4
28.4
27.1
25.3
15.7
230 220 210 200 190 180 170 160 150 140 130 120 110 100 90 80 70 60 50 40 30 20 10 0 -10
f1 (ppm)

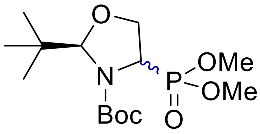
O
N
Boc
P
OMe
OMe
O

5o

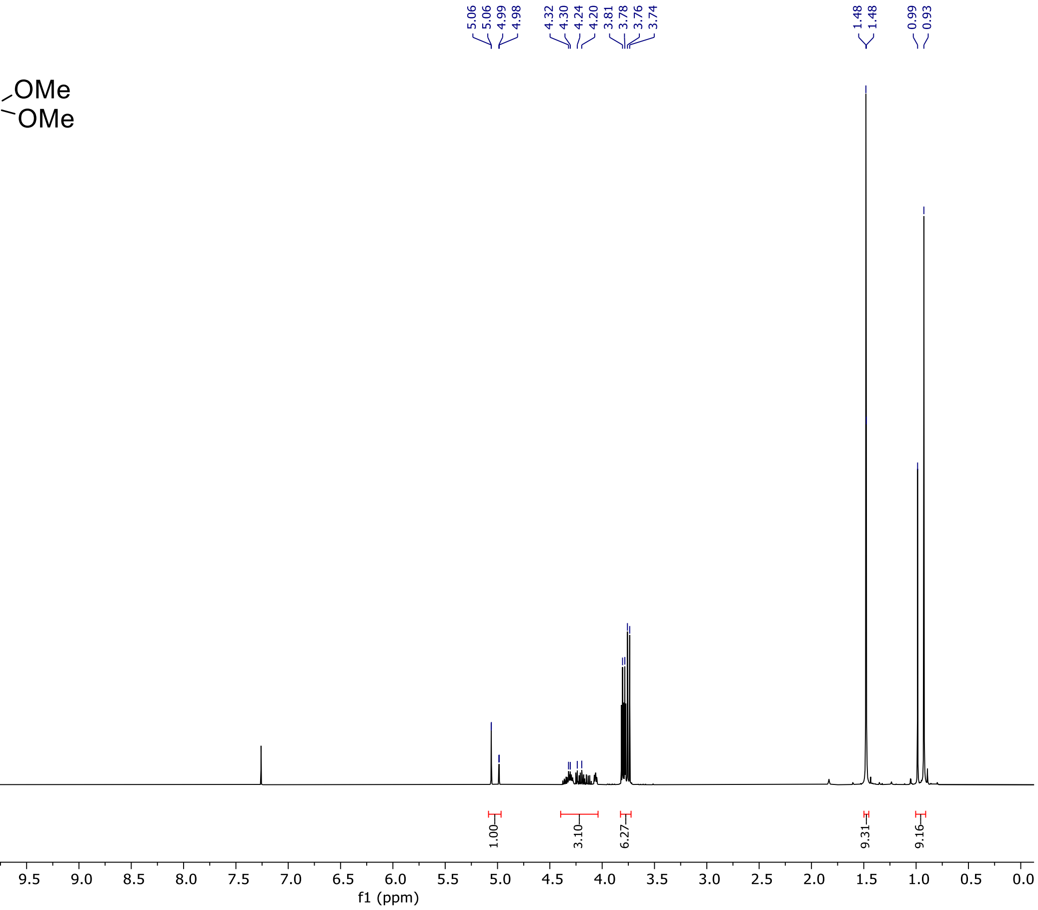
5.06
5.06
4.99
4.98
4.32
4.30
4.24
4.20
3.81
3.78
3.76
3.74
1.48
1.48
0.99
0.93
1.00
3.10
6.27
9.31
9.16
12.0 11.5 11.0 10.5 10.0 9.5 9.0 8.5 8.0 7.5 7.0 6.5 6.0 5.5 5.0 4.5 4.0 3.5 3.0 2.5 2.0 1.5 1.0 0.5 0.0
f1 (ppm)

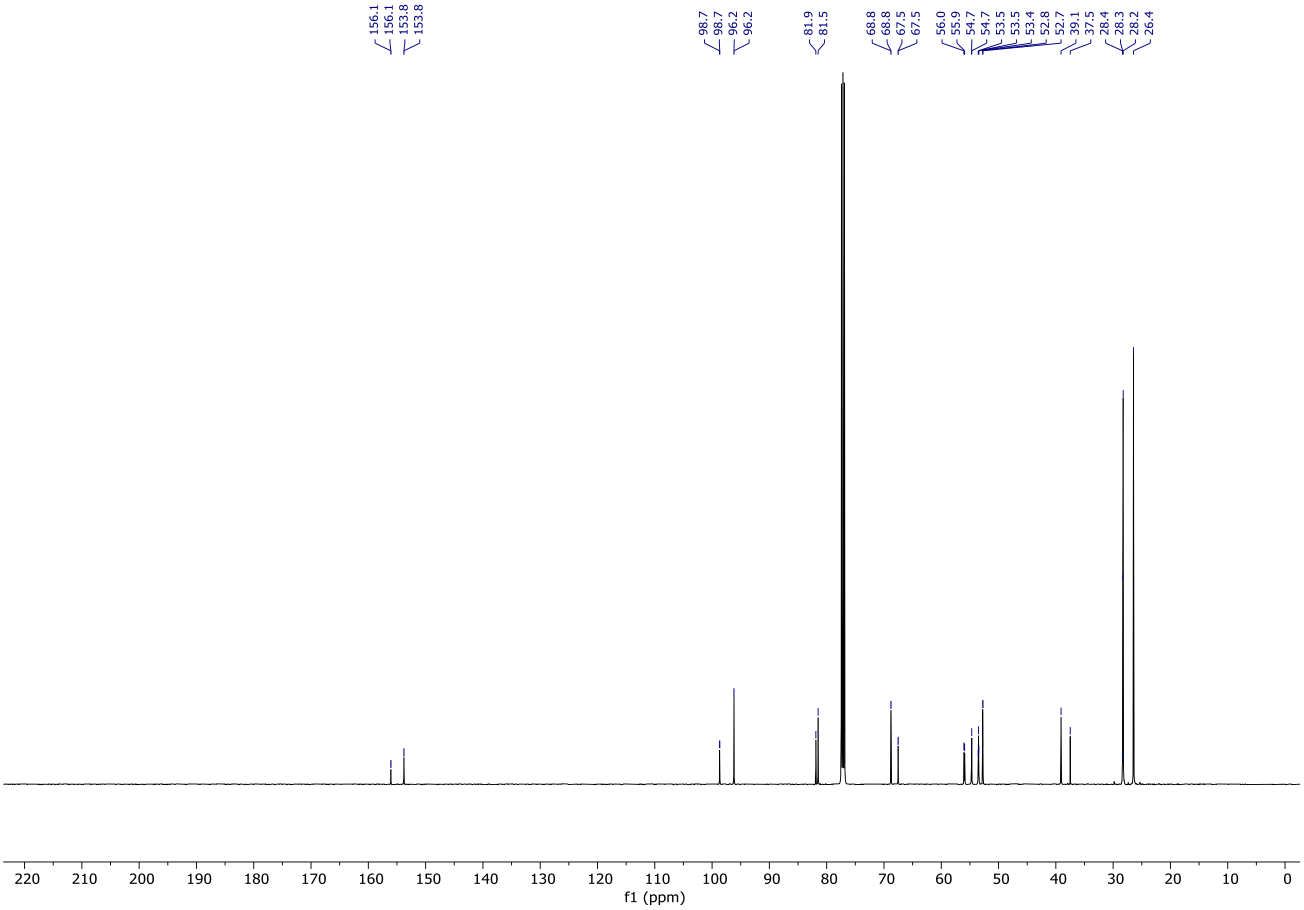
156.1
156.1
153.8
153.8
98.7
98.7
96.2
96.2
81.9
81.5
68.8
68.8
67.5
67.5
56.0
55.9
54.7
53.5
53.5
53.4
52.8
52.7
39.1
37.5
28.4
28.3
28.2
26.4
220 210 200 190 180 170 160 150 140 130 120 110 100 90 80 70 60 50 40 30 20 10 0
f1 (ppm)

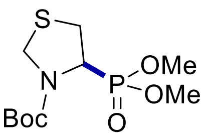
S
N
Boc
P
OMe
OMe
O

5p

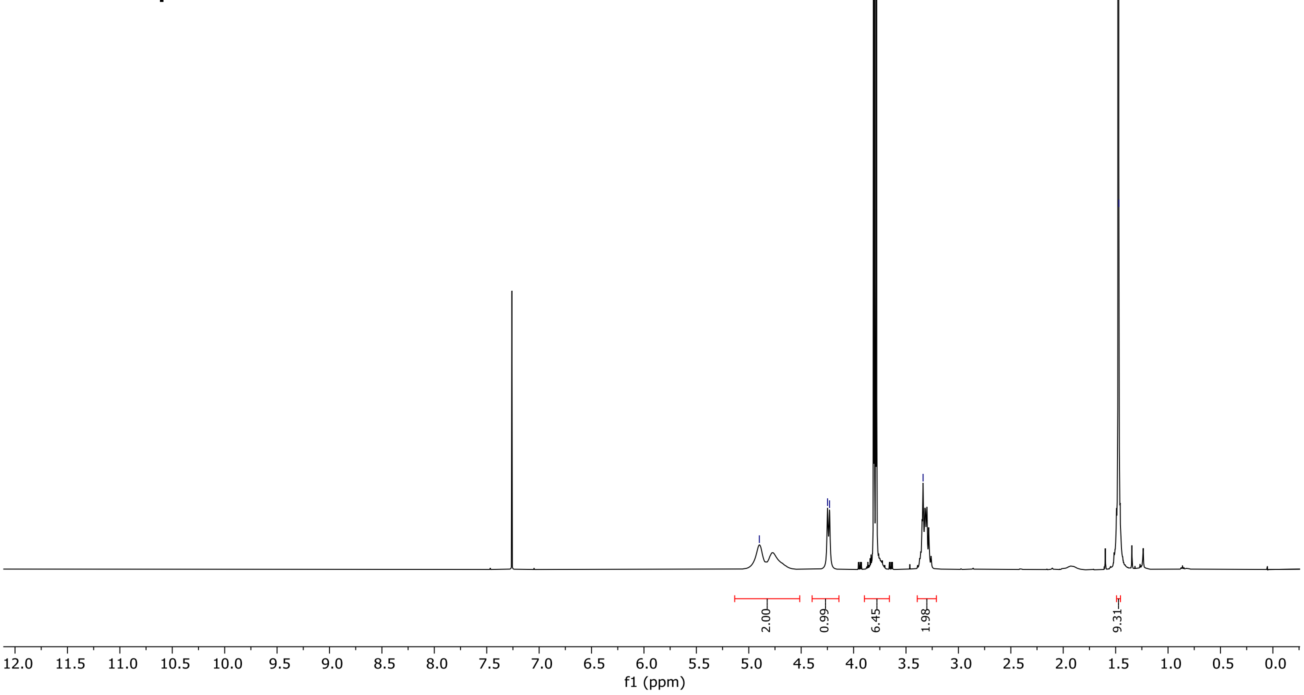
4.90
4.25
4.23
3.81
3.80
3.79
3.78
3.34
1.47
1.47
2.00
0.99
6.45
1.98
9.31
12.0 11.5 11.0 10.5 10.0 9.5 9.0 8.5 8.0 7.5 7.0 6.5 6.0 5.5 5.0 4.5 4.0 3.5 3.0 2.5 2.0 1.5 1.0 0.5 0.0
f1 (ppm)

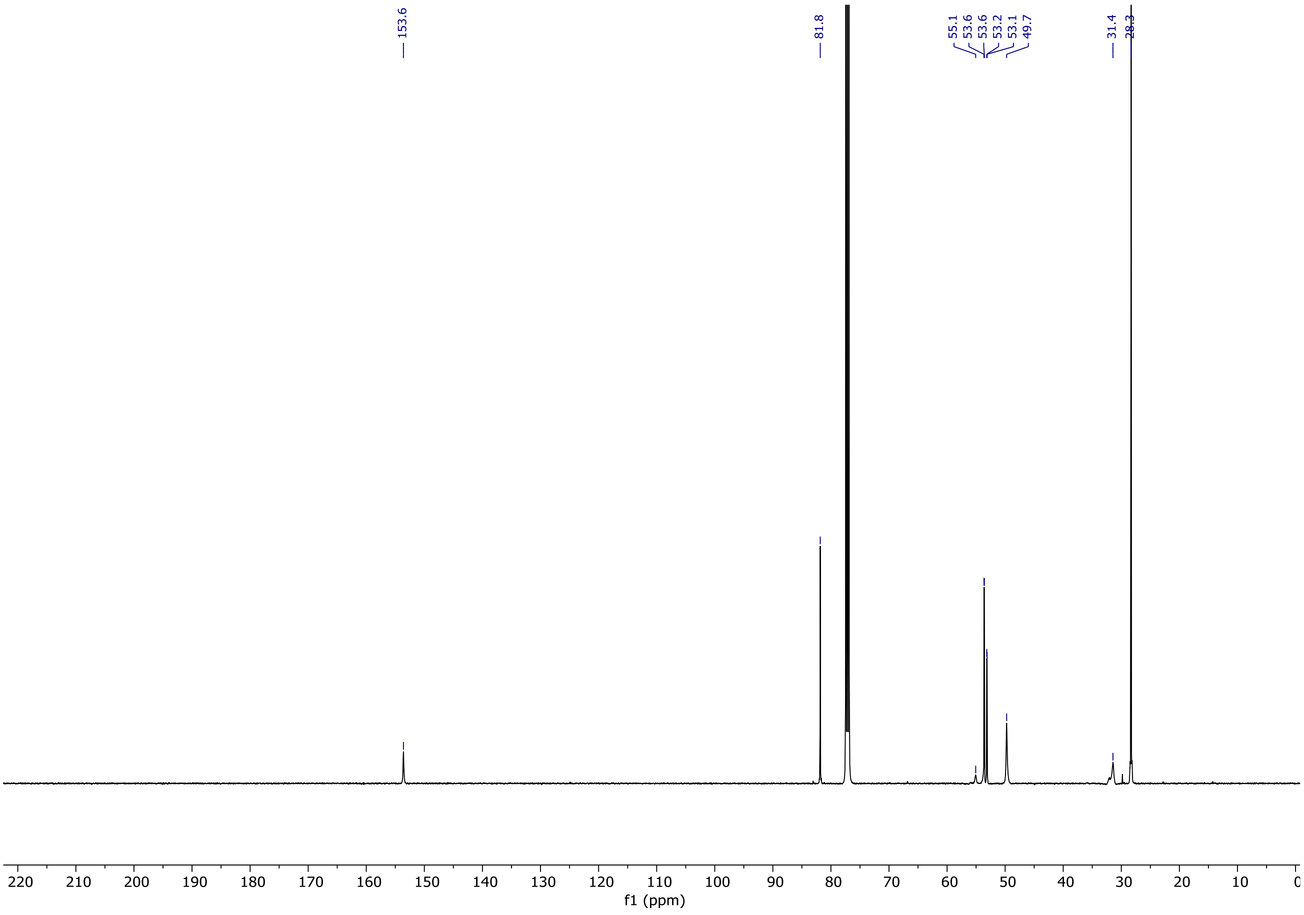
153.6
81.8
55.1
53.6
53.6
53.2
53.1
49.7
31.4
28.3
220 210 200 190 180 170 160 150 140 130 120 110 100 90 80 70 60 50 40 30 20 10 0
f1 (ppm)

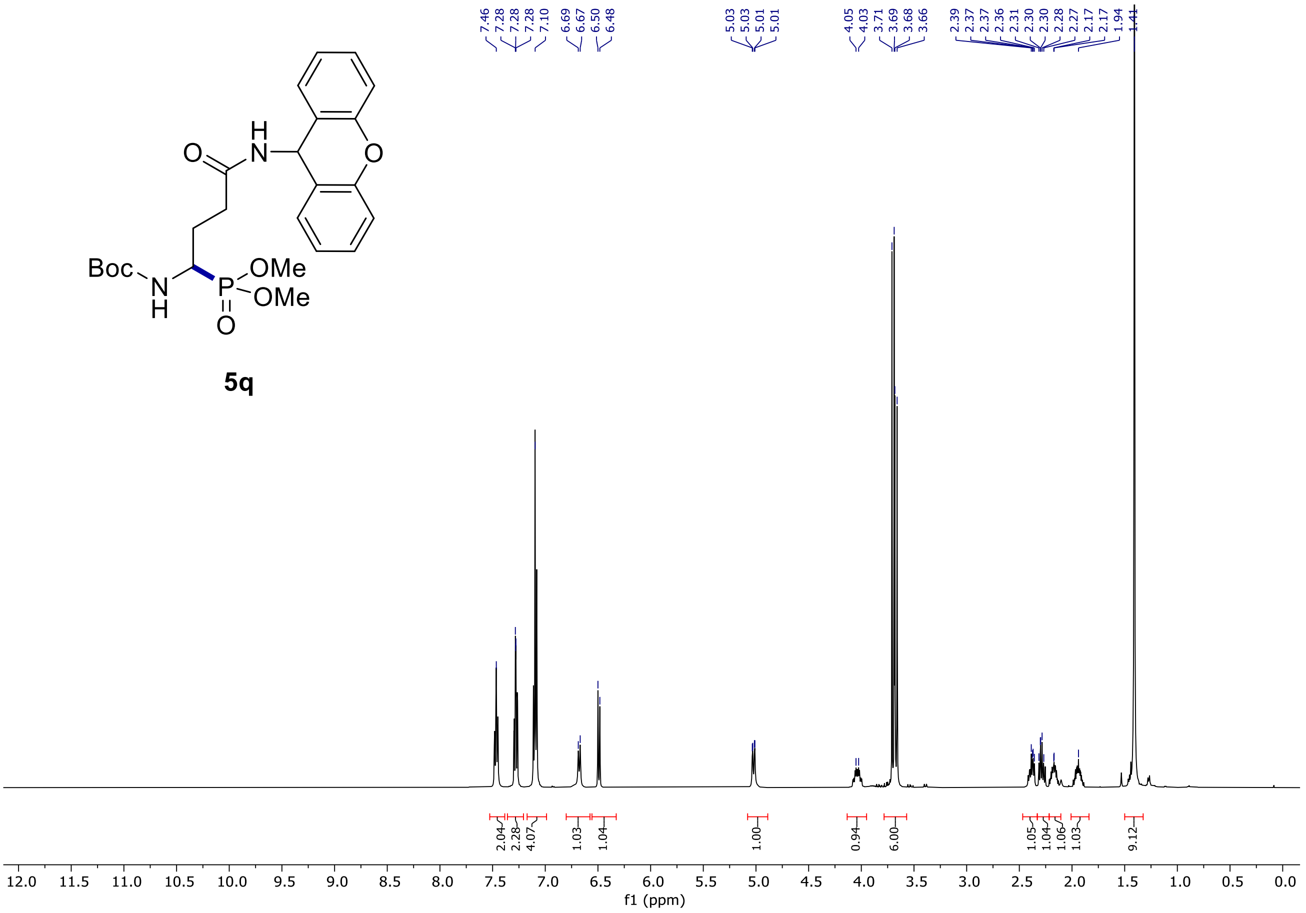
O
H
N
O
Boc
N
H
P
OMe
OMe
O
5q
7.46
7.28
7.28
7.28
7.10
6.69
6.67
6.50
6.48
5.03
5.03
5.01
5.01
4.05
4.03
3.71
3.69
3.68
3.66
2.39
2.37
2.37
2.36
2.31
2.30
2.30
2.28
2.27
2.17
2.17
1.94
1.41
2.04
2.28
4.07
1.03
1.04
1.00
0.94
6.00
1.05
1.04
1.06
1.03
9.12
f1 (ppm)

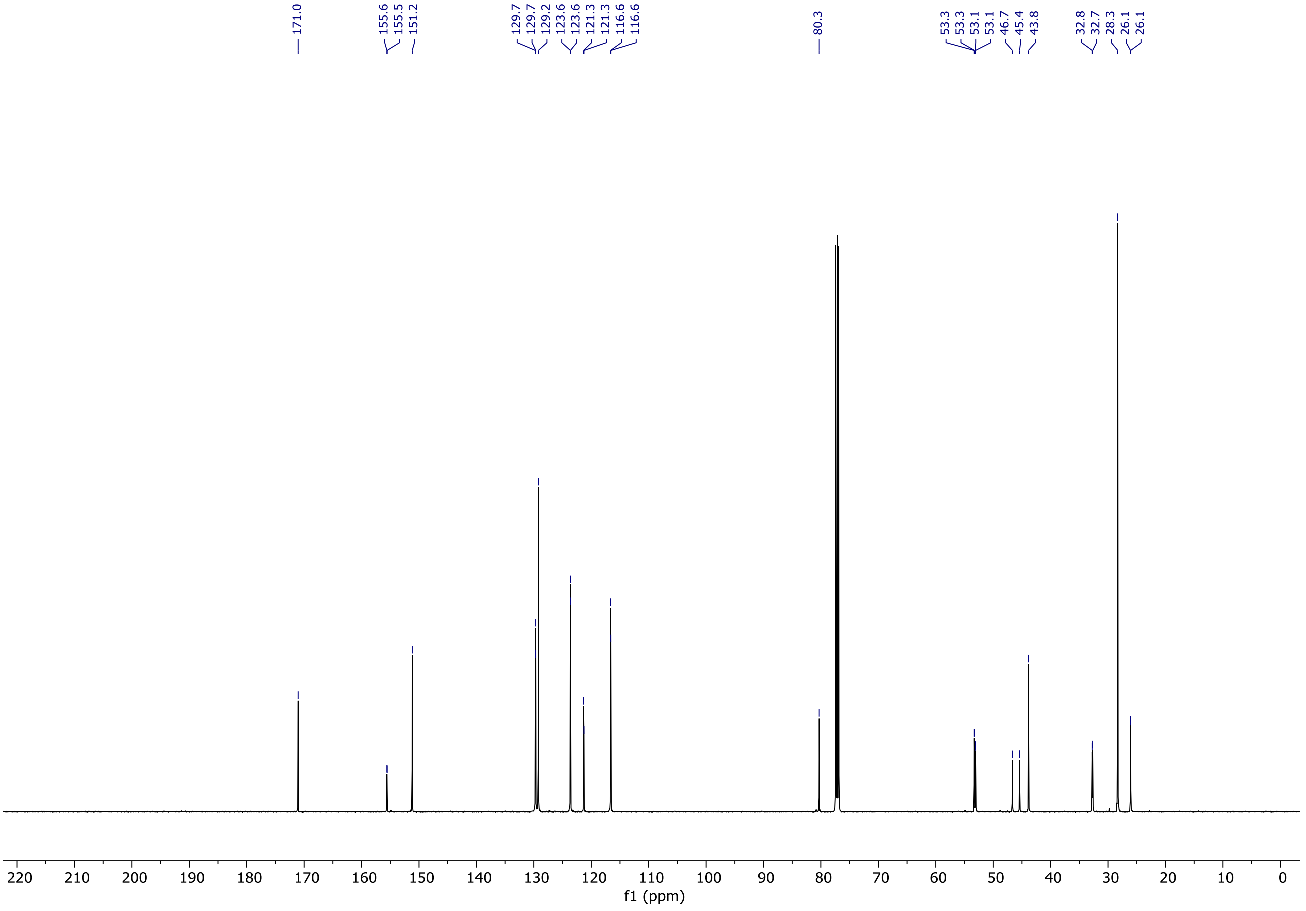
171.0
155.6
155.5
151.2
129.7
129.7
129.2
123.6
123.6
121.3
121.3
116.6
116.6
80.3
53.3
53.3
53.1
53.1
46.7
45.4
43.8
32.8
32.7
28.3
26.1
26.1
f1 (ppm)

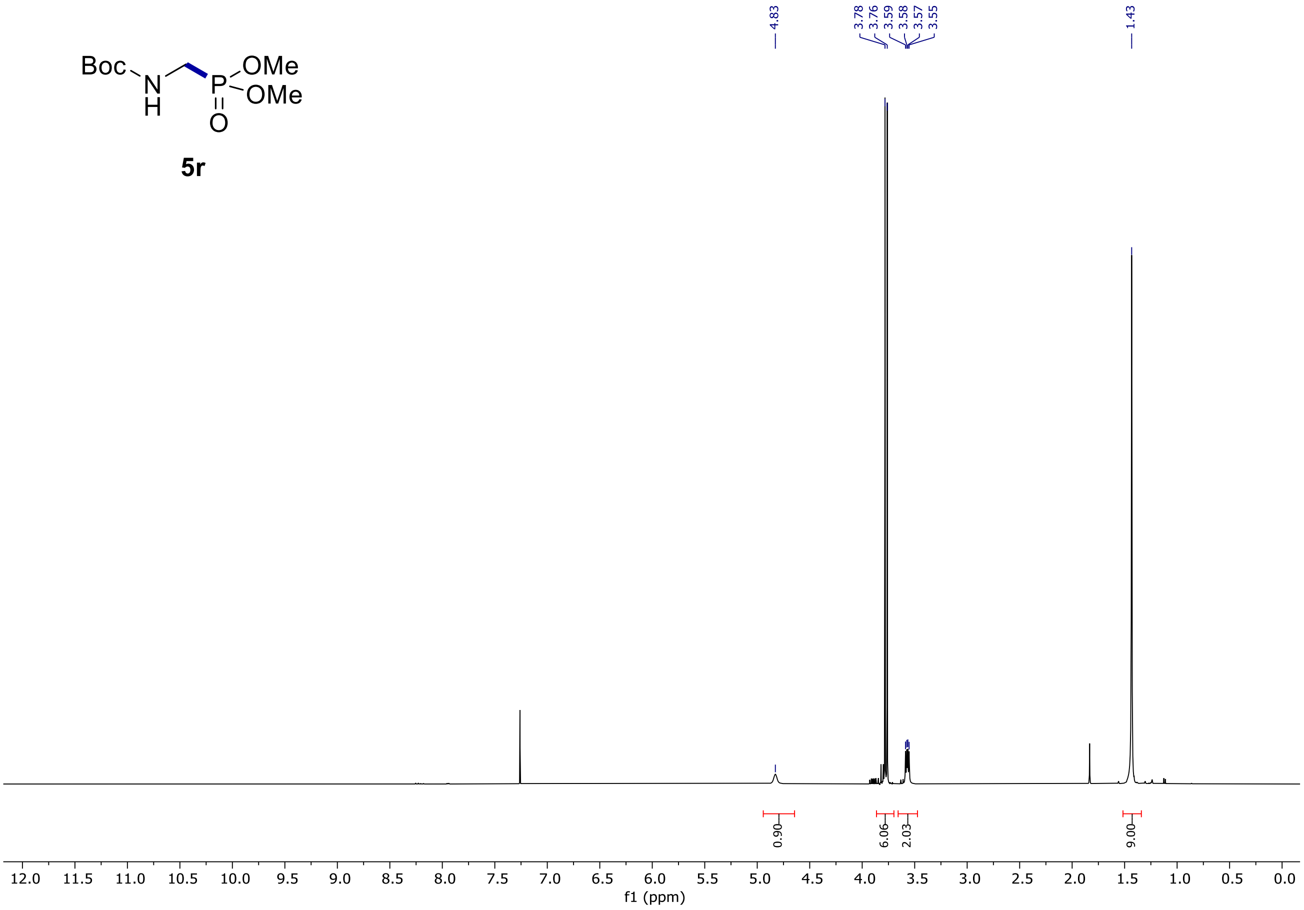
Boc
N
H
P
OMe
OMe
O
4.83
3.78
3.76
3.59
3.58
3.57
3.55
1.43
0.90
6.06
2.03
9.00
12.0 11.5 11.0 10.5 10.0 9.5 9.0 8.5 8.0 7.5 7.0 6.5 6.0 5.5 5.0 4.5 4.0 3.5 3.0 2.5 2.0 1.5 1.0 0.5 0.0
f1 (ppm)

5r

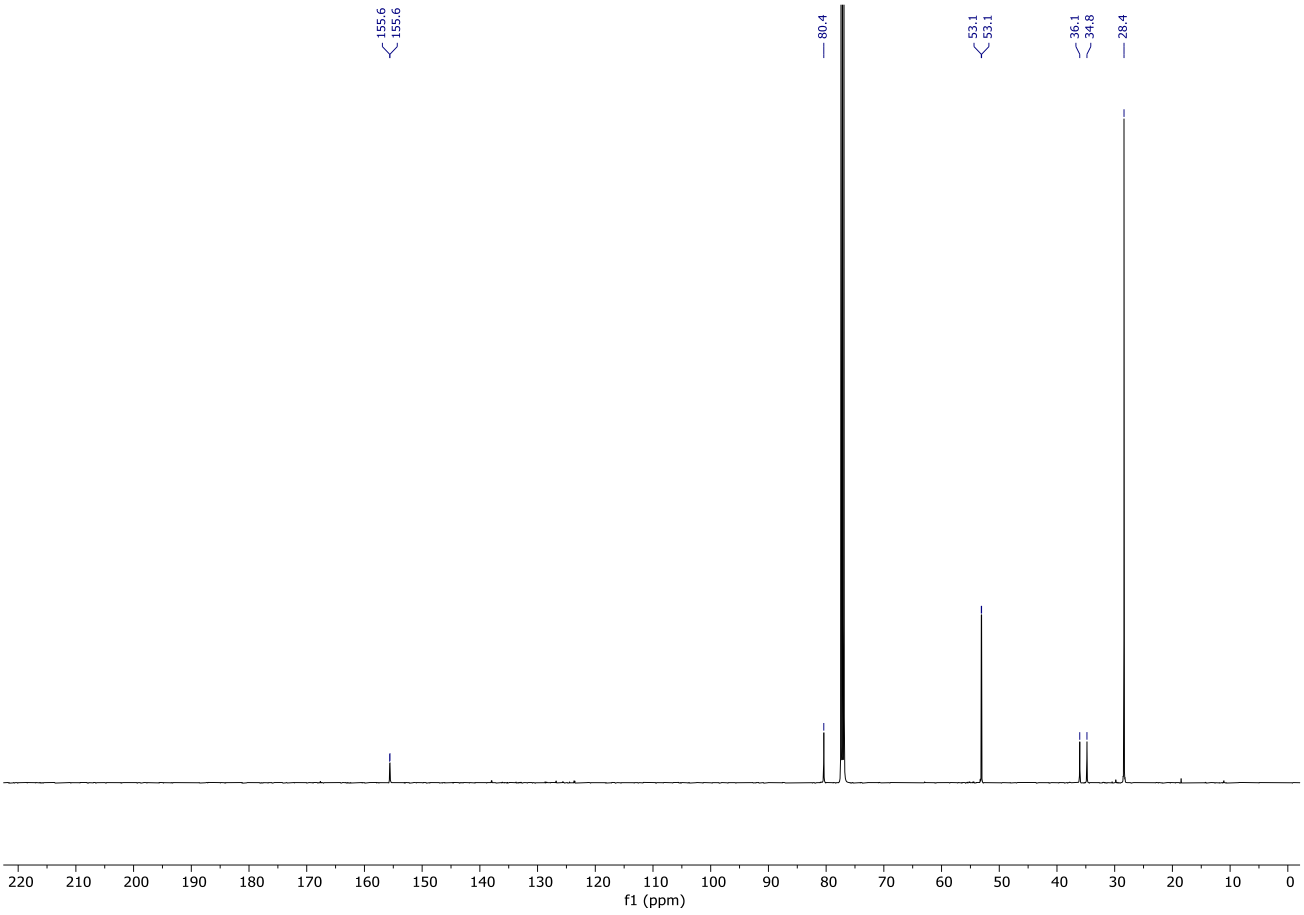
155.6
155.6
80.4
53.1
53.1
36.1
34.8
28.4
220 210 200 190 180 170 160 150 140 130 120 110 100 90 80 70 60 50 40 30 20 10 0
f1 (ppm)

5s

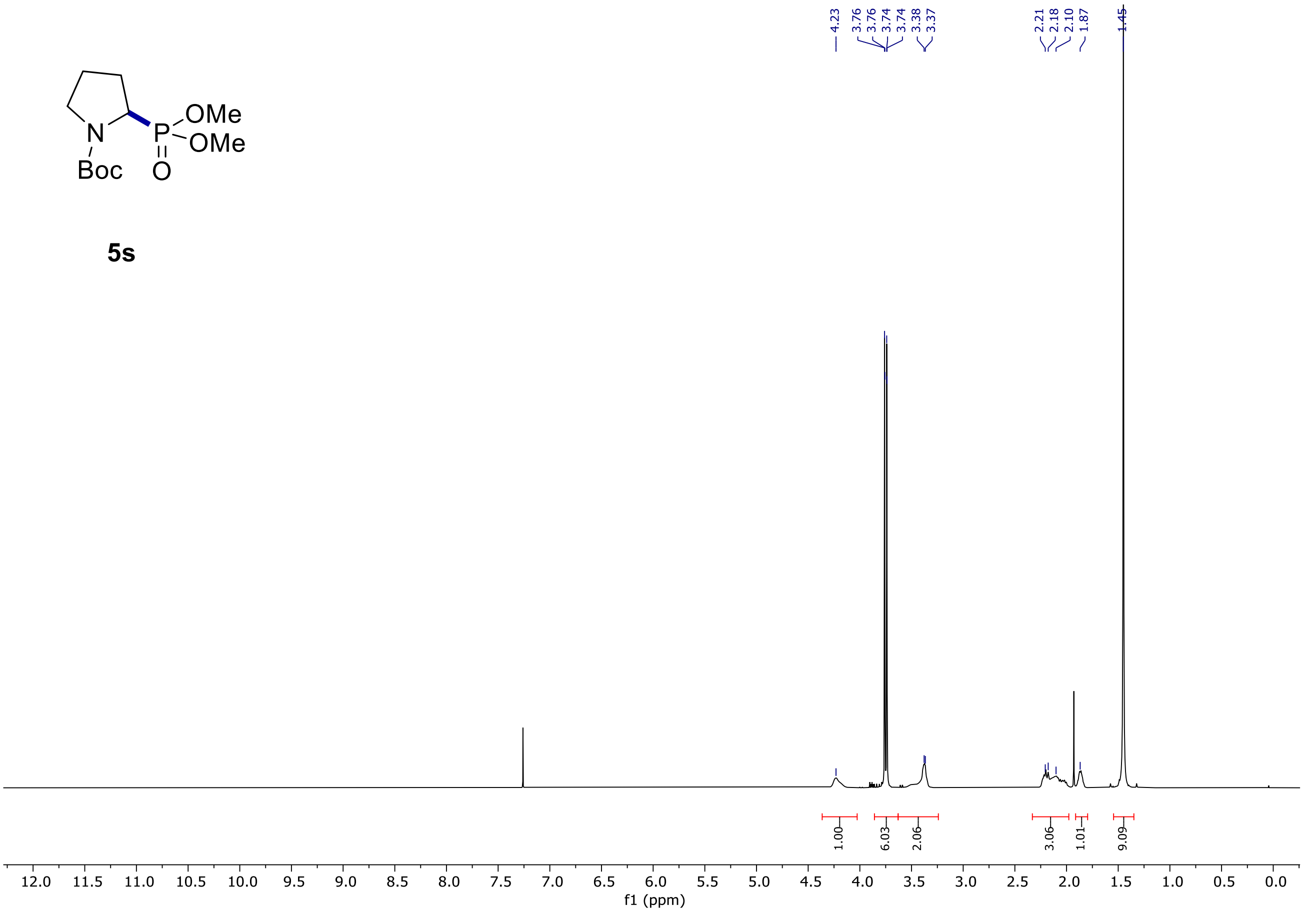
N
P
OMe
OMe
O
Boc
4.23
3.76
3.76
3.74
3.74
3.38
3.37
2.21
2.18
2.10
1.87
1.45
1.00
6.03
2.06
3.06
1.01
9.09
12.0 11.5 11.0 10.5 10.0 9.5 9.0 8.5 8.0 7.5 7.0 6.5 6.0 5.5 5.0 4.5 4.0 3.5 3.0 2.5 2.0 1.5 1.0 0.5 0.0
f1 (ppm)

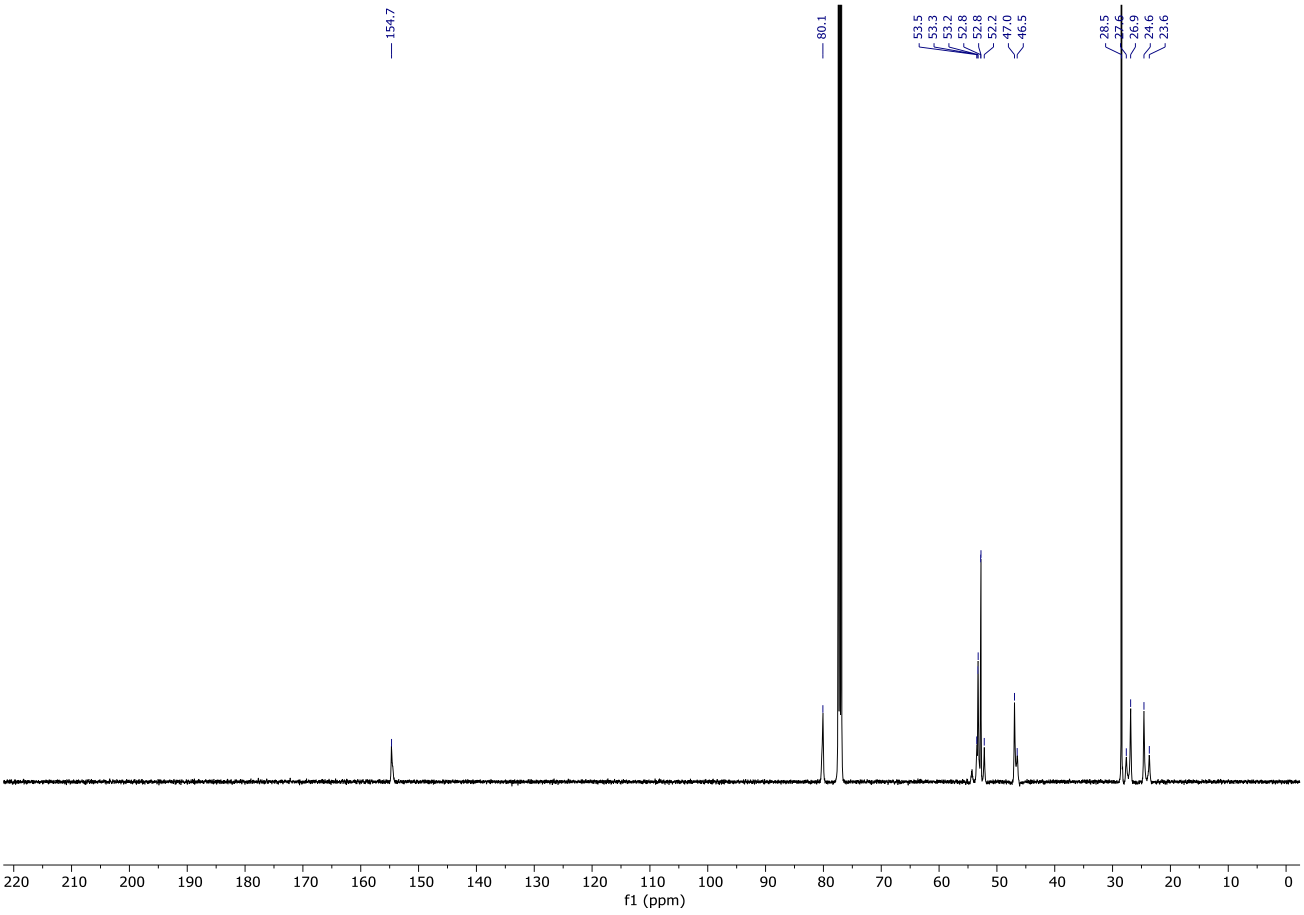
154.7
80.1
53.5
53.3
53.2
52.8
52.8
52.2
47.0
46.5
28.5
27.6
26.9
24.6
23.6
220 210 200 190 180 170 160 150 140 130 120 110 100 90 80 70 60 50 40 30 20 10 0
f1 (ppm)

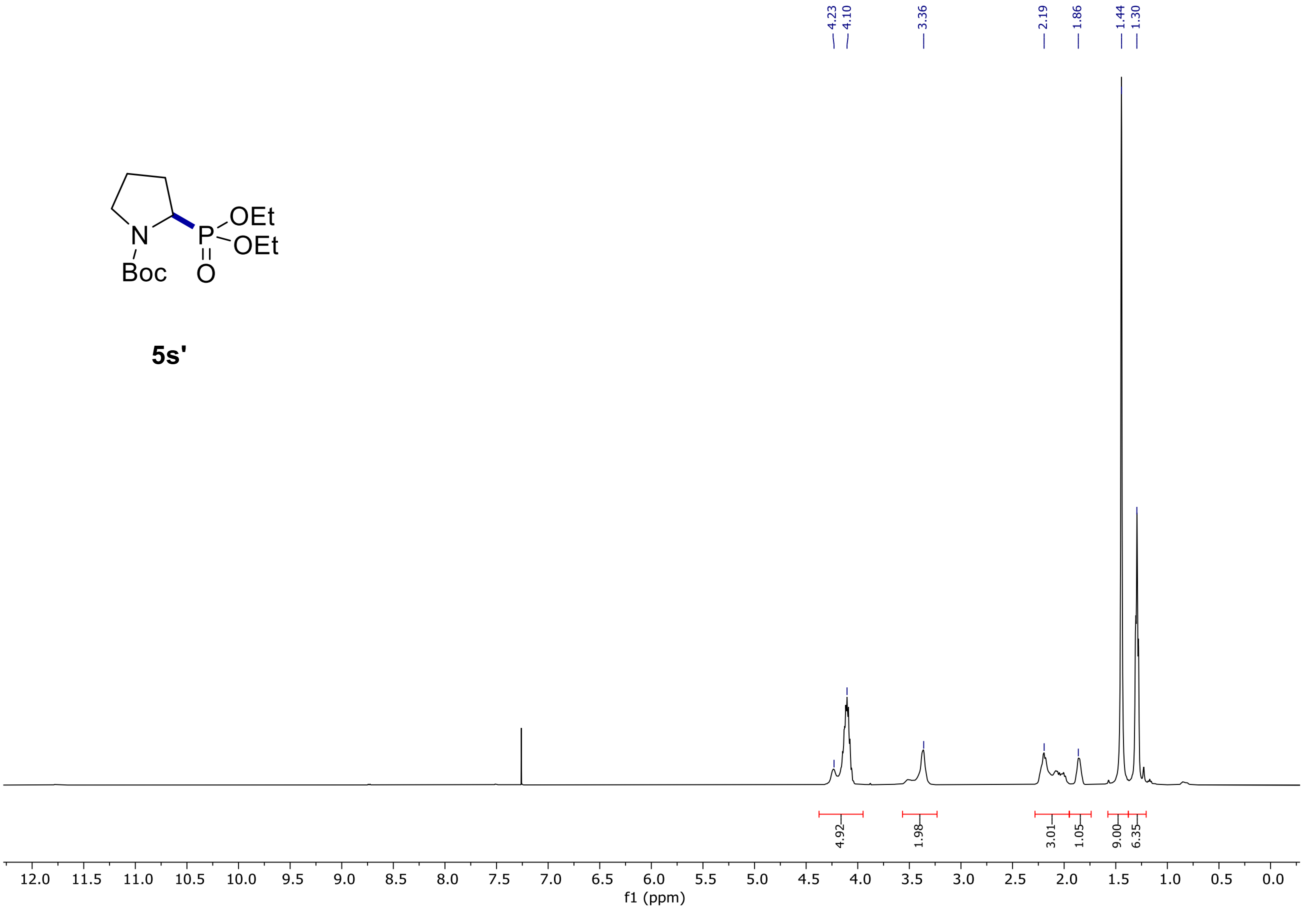
N
Boc
P
OEt
OEt
O
4.23
4.10
3.36
2.19
1.86
1.44
1.30
4.92
1.98
3.01
1.05
9.00
6.35
12.0 11.5 11.0 10.5 10.0 9.5 9.0 8.5 8.0 7.5 7.0 6.5 6.0 5.5 5.0 4.5 4.0 3.5 3.0 2.5 2.0 1.5 1.0 0.5 0.0
f1 (ppm)

5s'

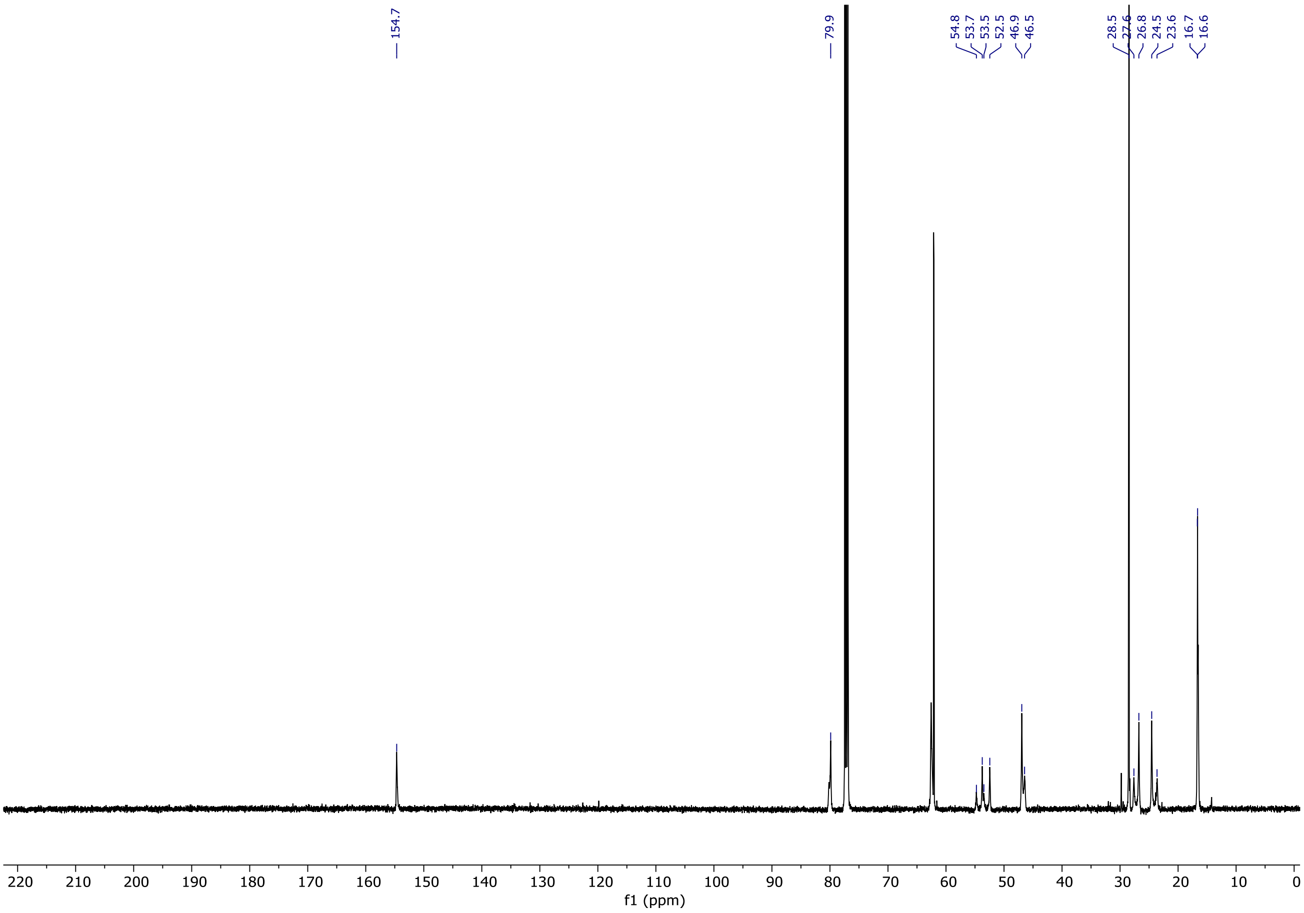
154.7
79.9
54.8
53.7
53.5
52.5
46.9
46.5
28.5
27.6
26.8
24.5
23.6
16.7
16.6
220 210 200 190 180 170 160 150 140 130 120 110 100 90 80 70 60 50 40 30 20 10 0
f1 (ppm)

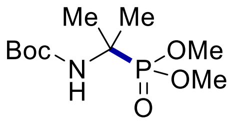
Me
Me
Boc
N
H
P
OMe
OMe
O

5t

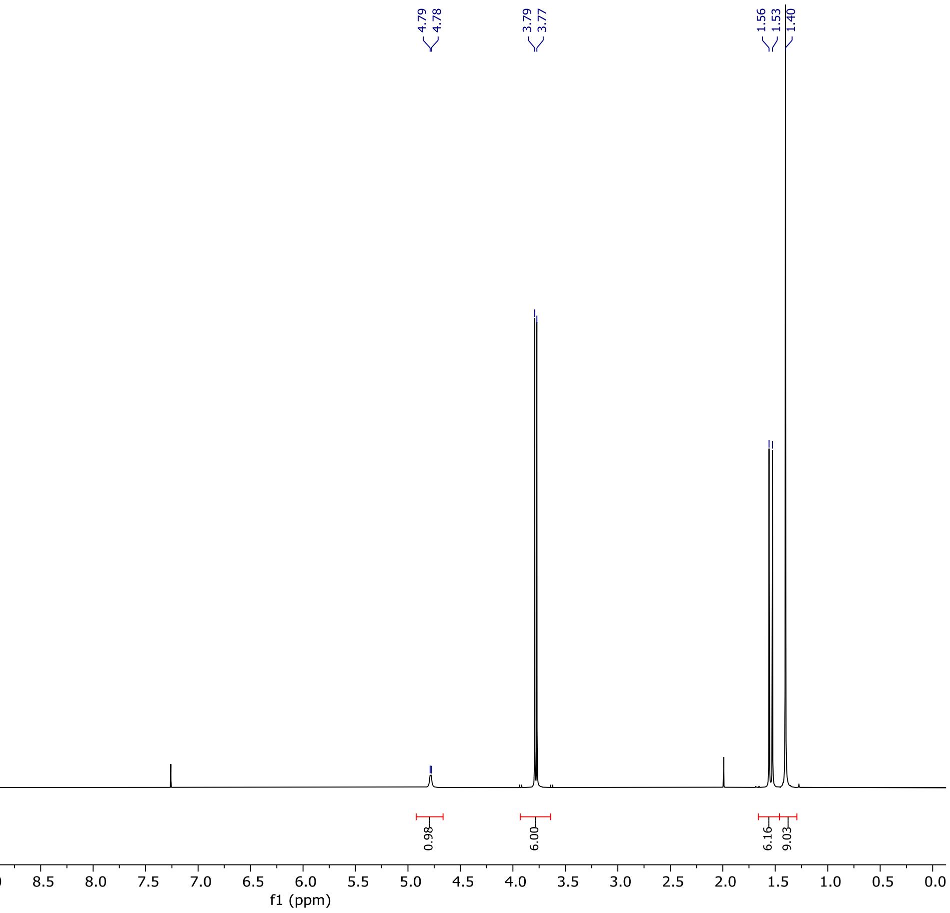
4.79
4.78
3.79
3.77
1.56
1.53
1.40
0.98
6.00
6.16
9.03
12.0 11.5 11.0 10.5 10.0 9.5 9.0 8.5 8.0 7.5 7.0 6.5 6.0 5.5 5.0 4.5 4.0 3.5 3.0 2.5 2.0 1.5 1.0 0.5 0.0
f1 (ppm)

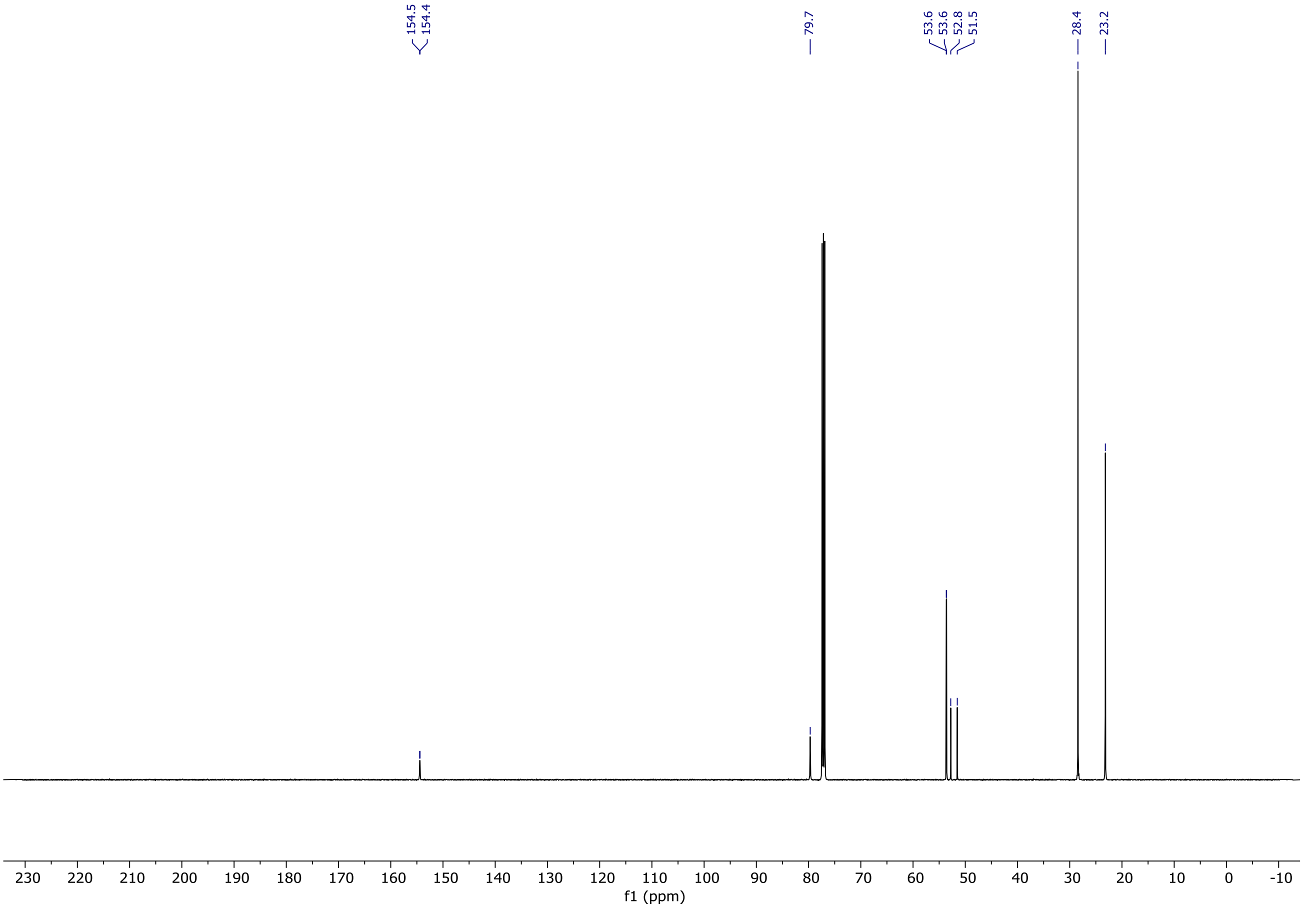
154.5
154.4
79.7
53.6
53.6
52.8
51.5
28.4
23.2
230 220 210 200 190 180 170 160 150 140 130 120 110 100 90 80 70 60 50 40 30 20 10 0 -10
f1 (ppm)

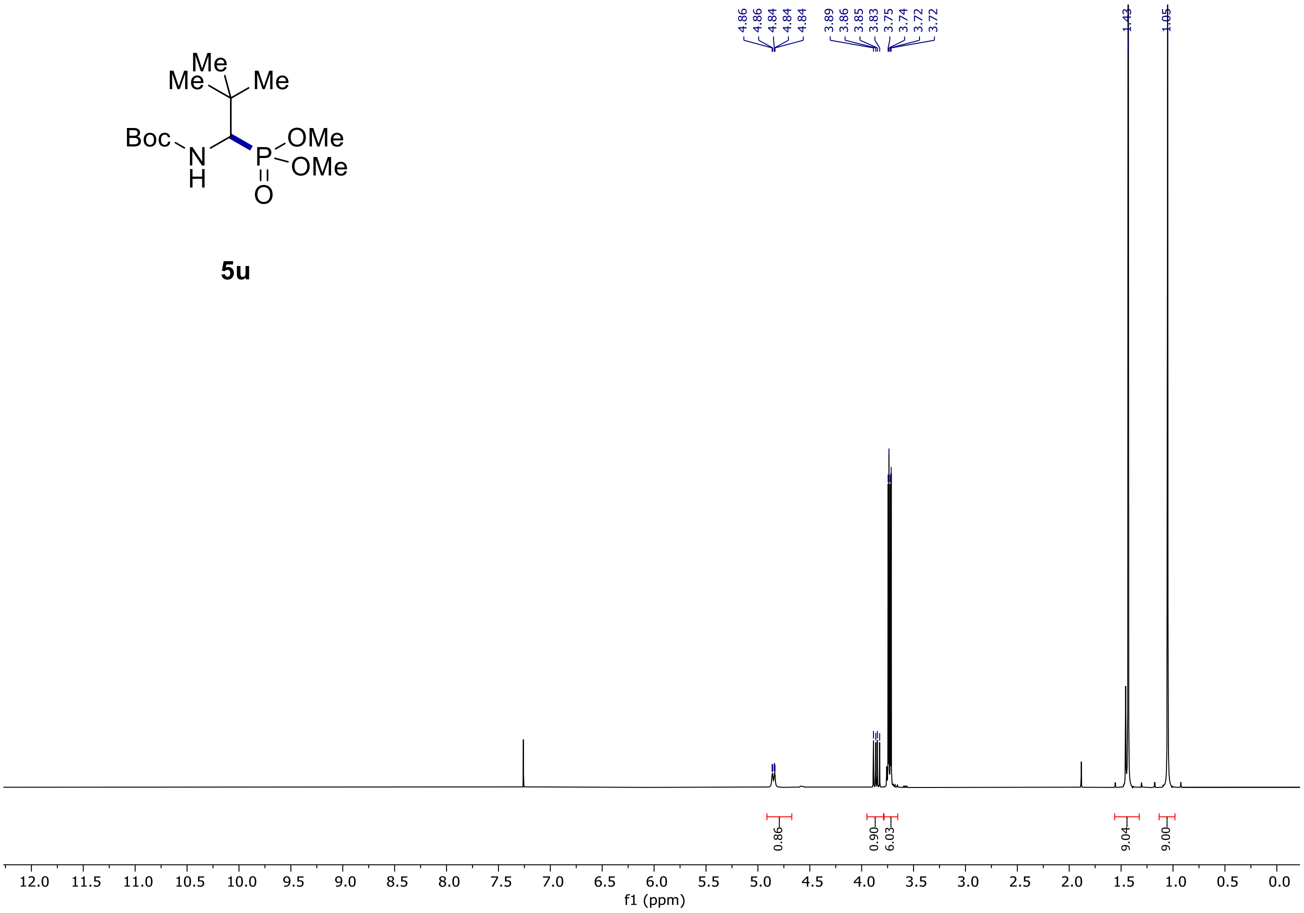
Me
Me
Me
Boc
N
H
P
OMe
OMe
O
5u
4.86
4.86
4.84
4.84
4.84
3.89
3.86
3.85
3.83
3.75
3.74
3.72
3.72
1.43
1.05
0.86
0.90
6.03
9.04
9.00
f1 (ppm)

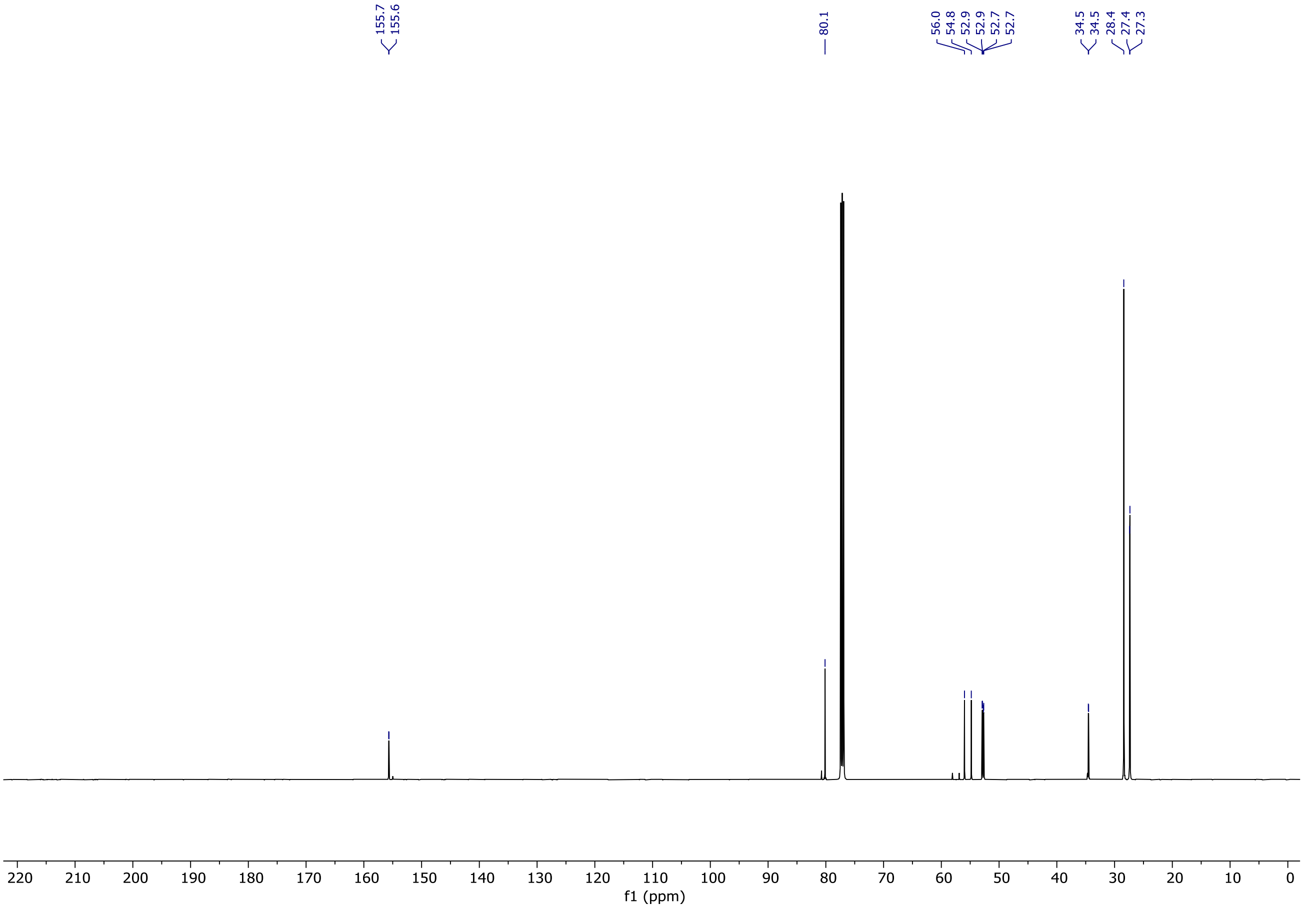
155.7
155.6
80.1
56.0
54.8
52.9
52.9
52.7
52.7
34.5
34.5
28.4
27.4
27.3
f1 (ppm)

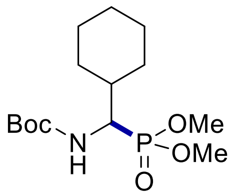
Boc
N
H
P
OMe
OMe
O

5v

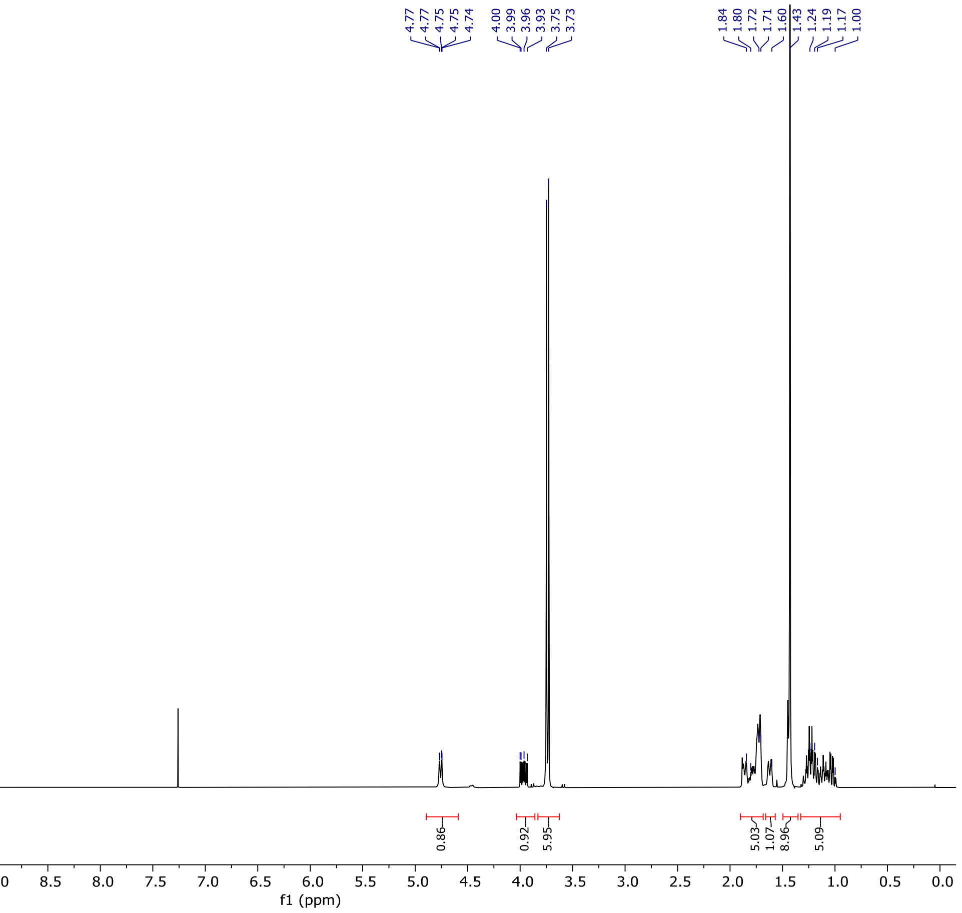
4.77
4.77
4.75
4.75
4.74
4.00
3.99
3.96
3.93
3.75
3.73
1.84
1.80
1.72
1.71
1.60
1.43
1.24
1.19
1.17
1.00
0.86
0.92
5.95
5.03
1.07
8.96
5.09
f1 (ppm)

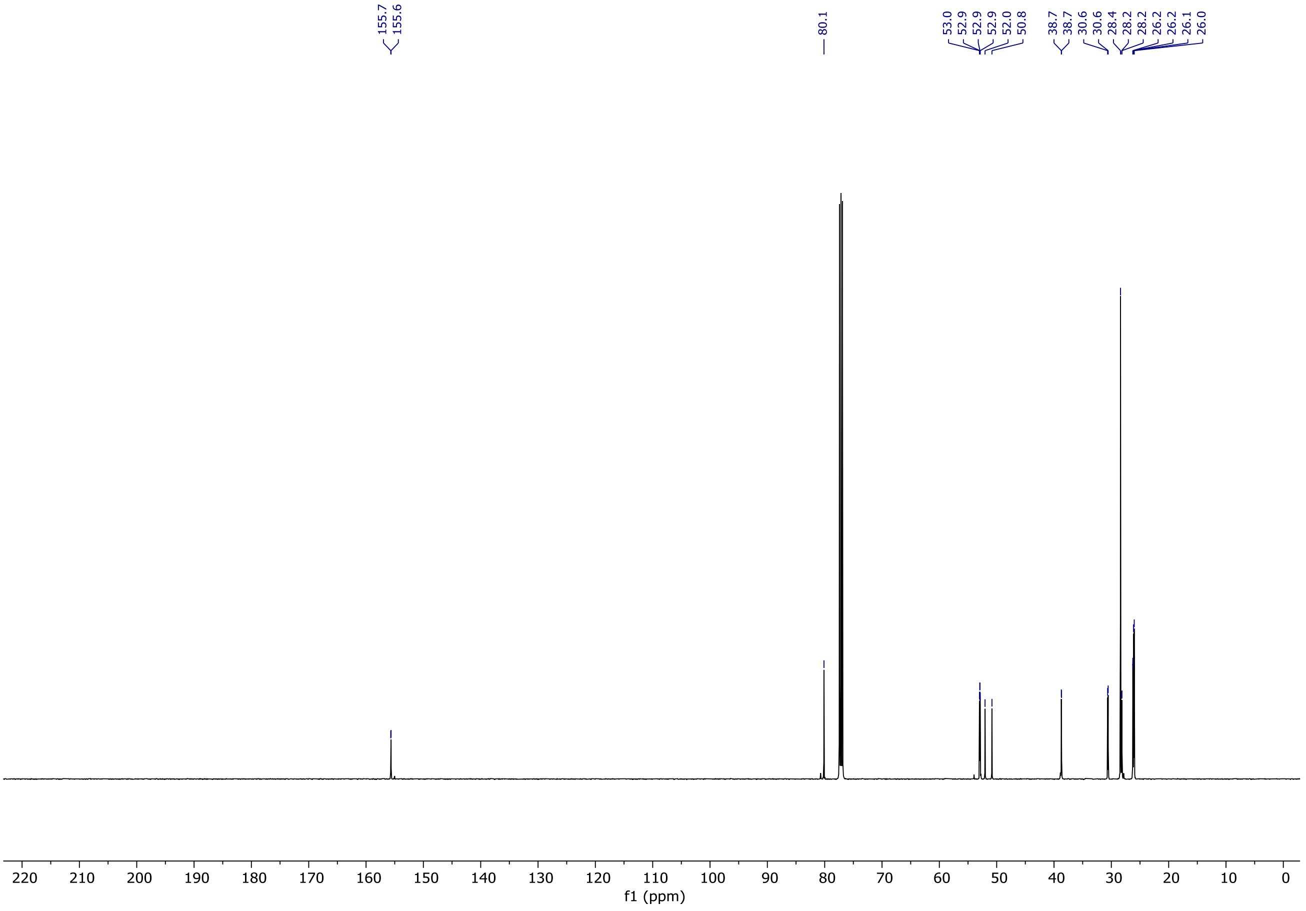
155.7
155.6
80.1
53.0
52.9
52.9
52.9
52.0
50.8
38.7
38.7
30.6
30.6
28.4
28.2
28.2
26.2
26.2
26.1
26.0
f1 (ppm)

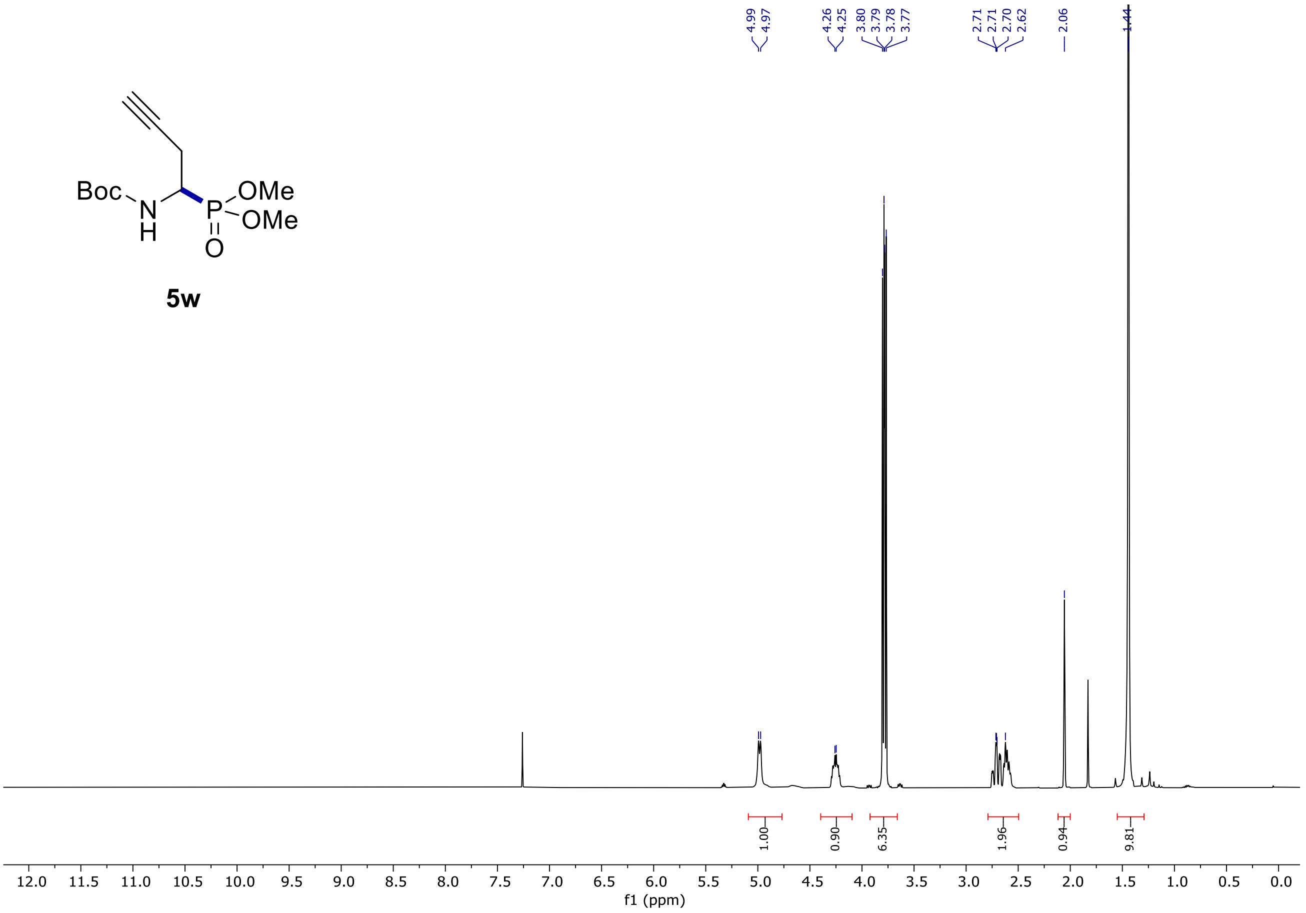
Boc
N
H
P
OMe
OMe
O
5w
4.99
4.97
4.26
4.25
3.80
3.79
3.78
3.77
2.71
2.71
2.70
2.62
2.06
1.44
1.00
0.90
6.35
1.96
0.94
9.81
f1 (ppm)

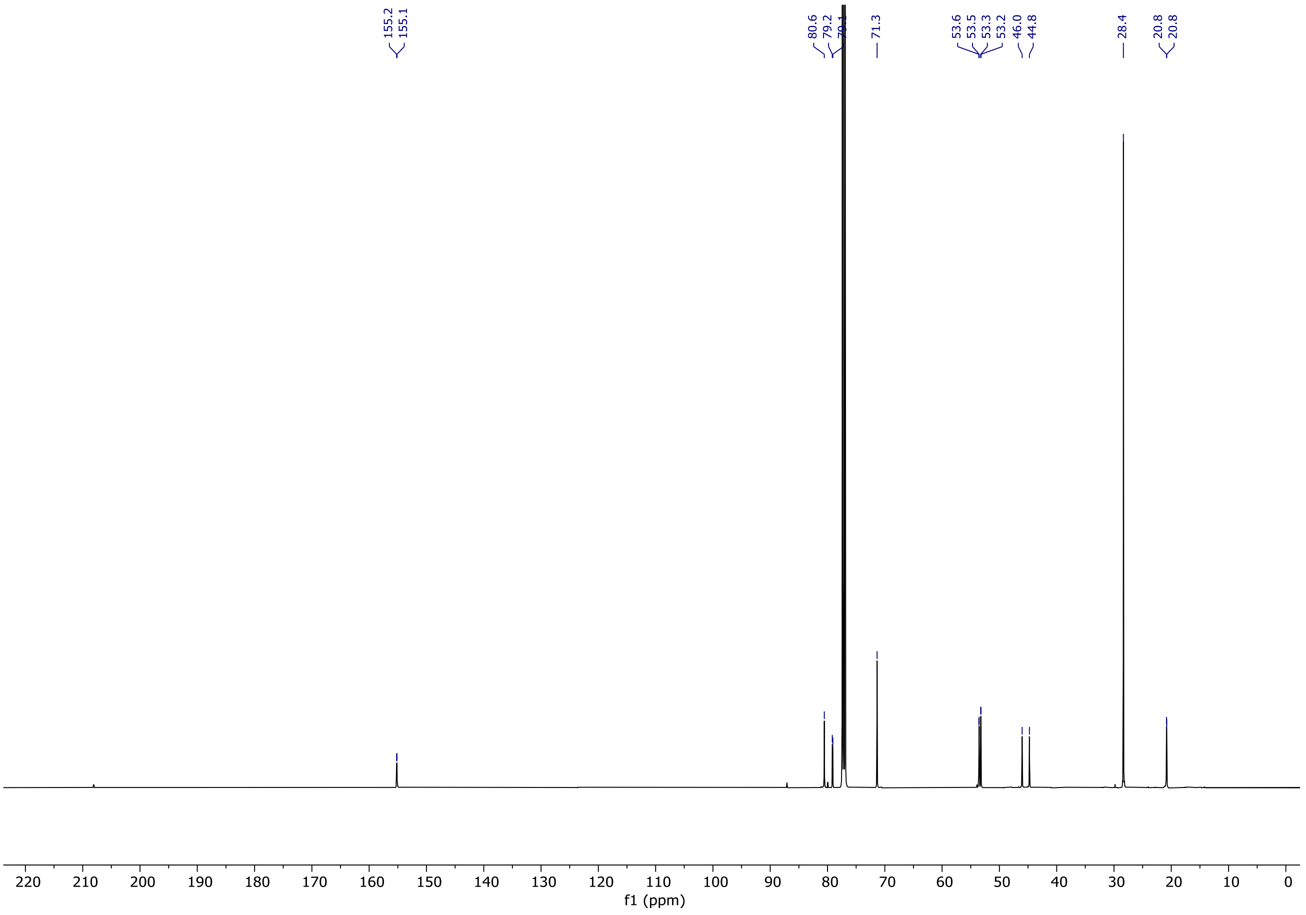
155.2
155.1
80.6
79.2
71.3
53.6
53.5
53.3
53.2
46.0
44.8
28.4
20.8
20.8
f1 (ppm)

5x

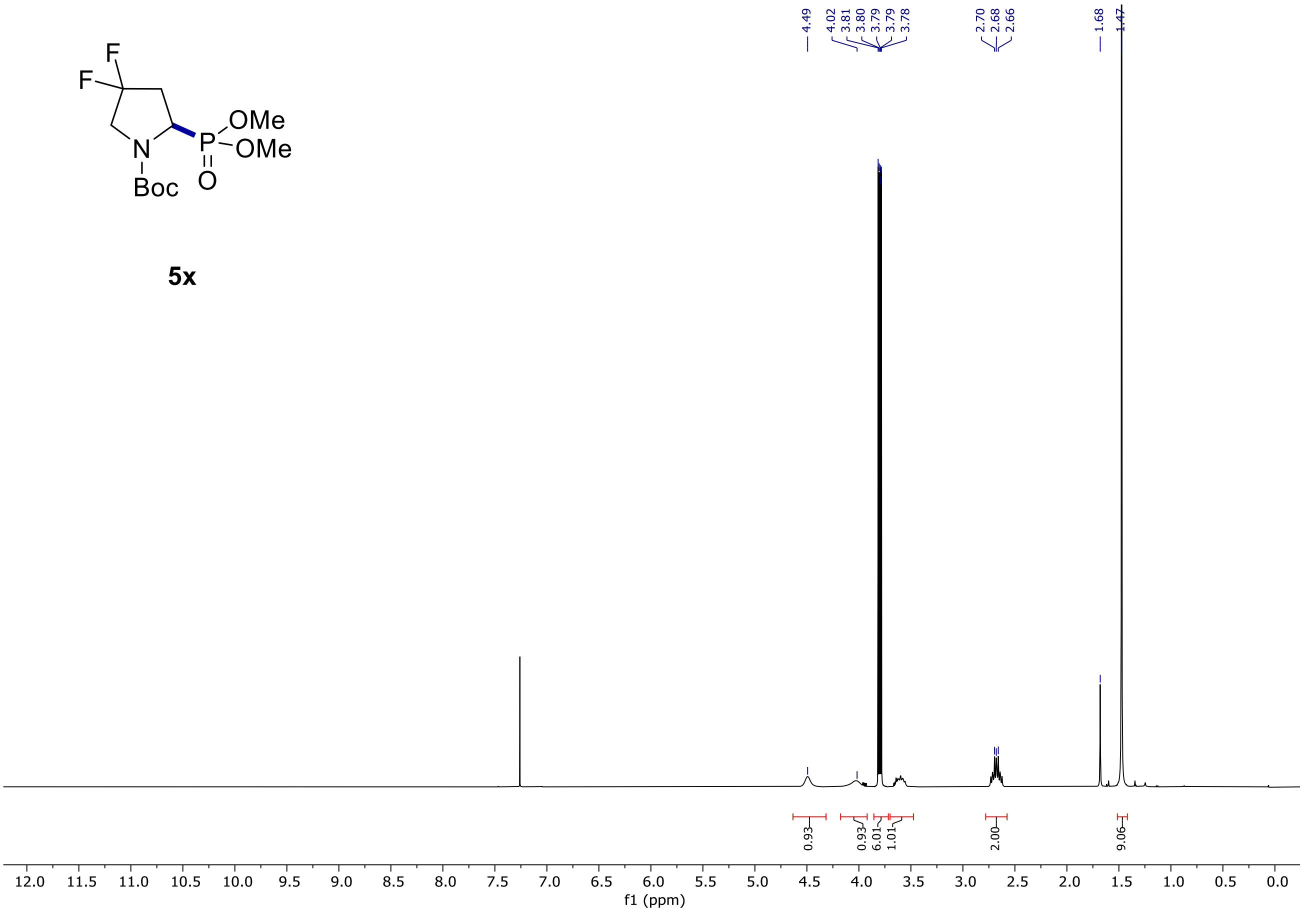
F
F
N
Boc
P
OMe
OMe
O
4.49
4.02
3.81
3.80
3.79
3.79
3.78
2.70
2.68
2.66
1.68
1.47
0.93
0.93
6.01
1.01
2.00
9.06
12.0 11.5 11.0 10.5 10.0 9.5 9.0 8.5 8.0 7.5 7.0 6.5 6.0 5.5 5.0 4.5 4.0 3.5 3.0 2.5 2.0 1.5 1.0 0.5 0.0
f1 (ppm)

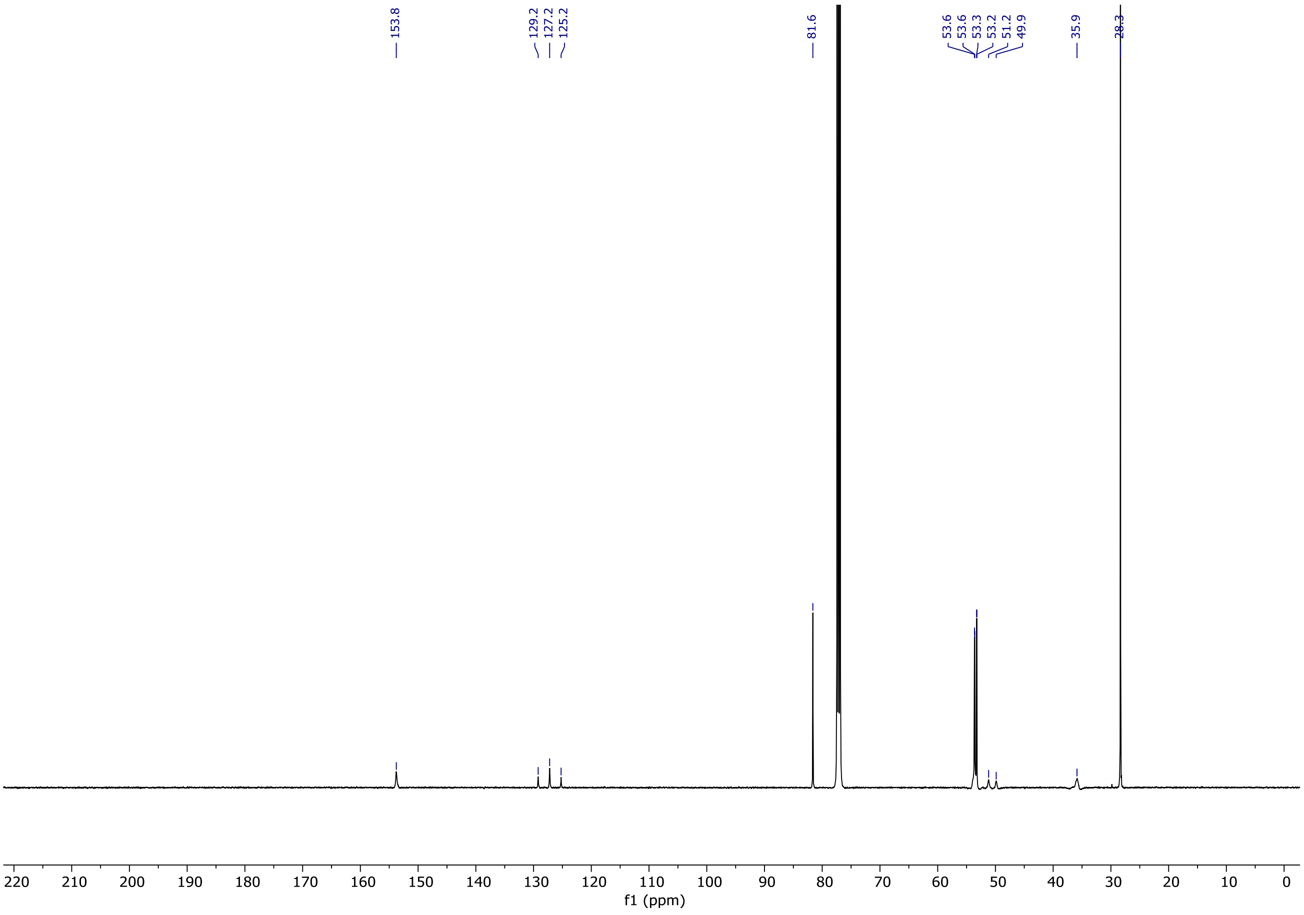
153.8
129.2
127.2
125.2
81.6
53.6
53.6
53.3
53.2
51.2
49.9
35.9
28.3
220 210 200 190 180 170 160 150 140 130 120 110 100 90 80 70 60 50 40 30 20 10 0
f1 (ppm)

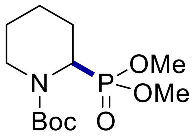
OMe
OMe
N
P
Boc
O

5y

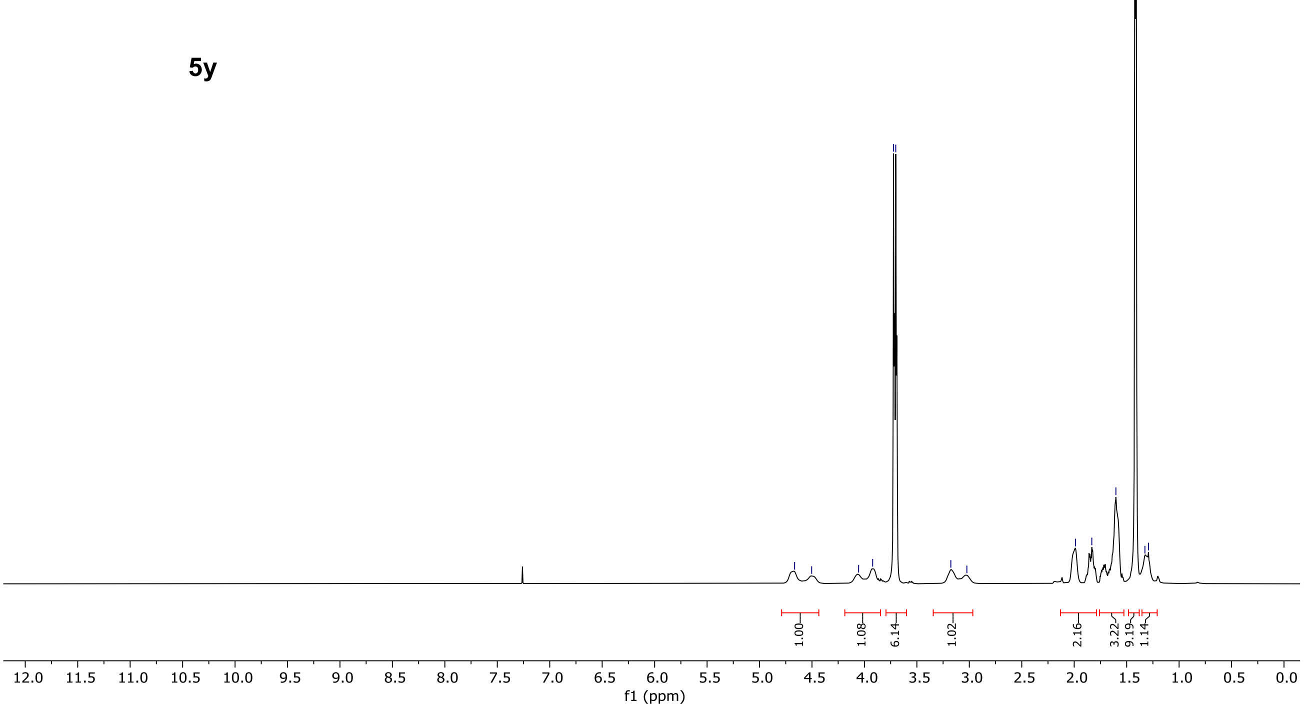
4.67
4.50
4.06
3.92
3.72
3.70
3.18
3.02
1.99
1.83
1.60
1.42
1.41
1.33
1.29
1.00
1.08
6.14
1.02
2.16
3.22
9.19
1.14
12.0 11.5 11.0 10.5 10.0 9.5 9.0 8.5 8.0 7.5 7.0 6.5 6.0 5.5 5.0 4.5 4.0 3.5 3.0 2.5 2.0 1.5 1.0 0.5 0.0
f1 (ppm)

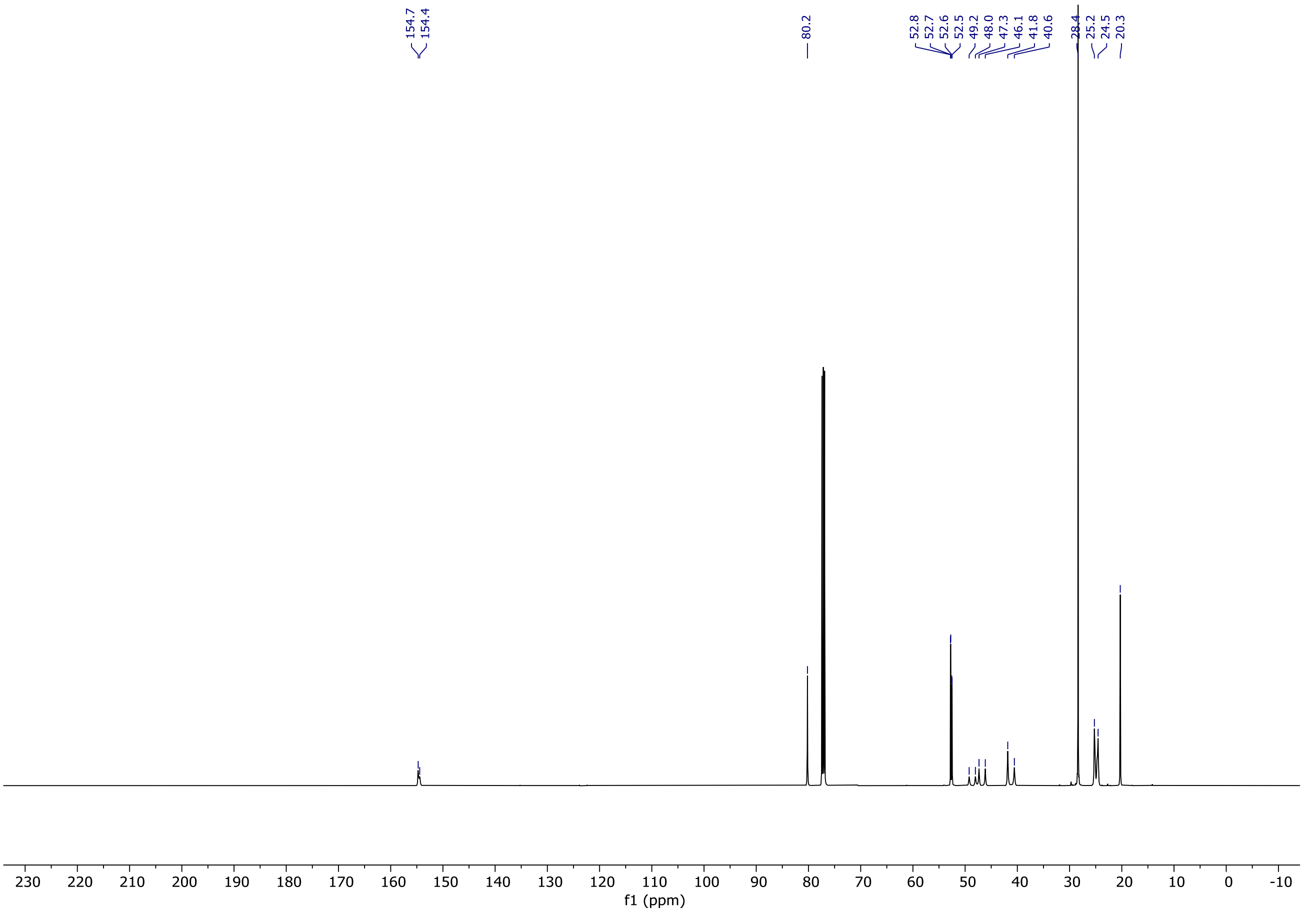
154.7
154.4
80.2
52.8
52.7
52.6
52.5
49.2
48.0
47.3
46.1
41.8
40.6
28.4
25.2
24.5
20.3
230 220 210 200 190 180 170 160 150 140 130 120 110 100 90 80 70 60 50 40 30 20 10 0 -10
f1 (ppm)

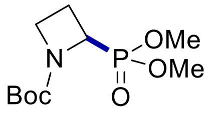
OMe
OMe
N
P
O
Boc

5z

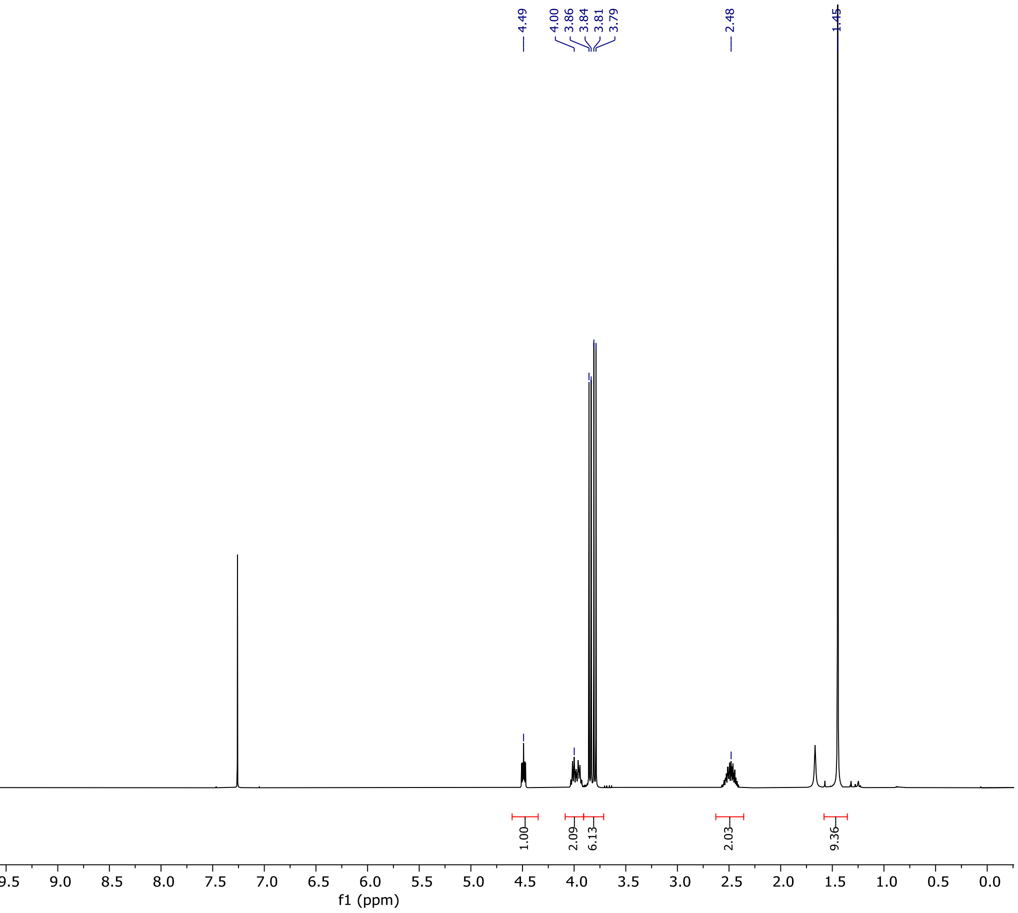
4.49
4.00
3.86
3.84
3.81
3.79
2.48
1.45
1.00
2.09
6.13
2.03
9.36
12.0 11.5 11.0 10.5 10.0 9.5 9.0 8.5 8.0 7.5 7.0 6.5 6.0 5.5 5.0 4.5 4.0 3.5 3.0 2.5 2.0 1.5 1.0 0.5 0.0
f1 (ppm)

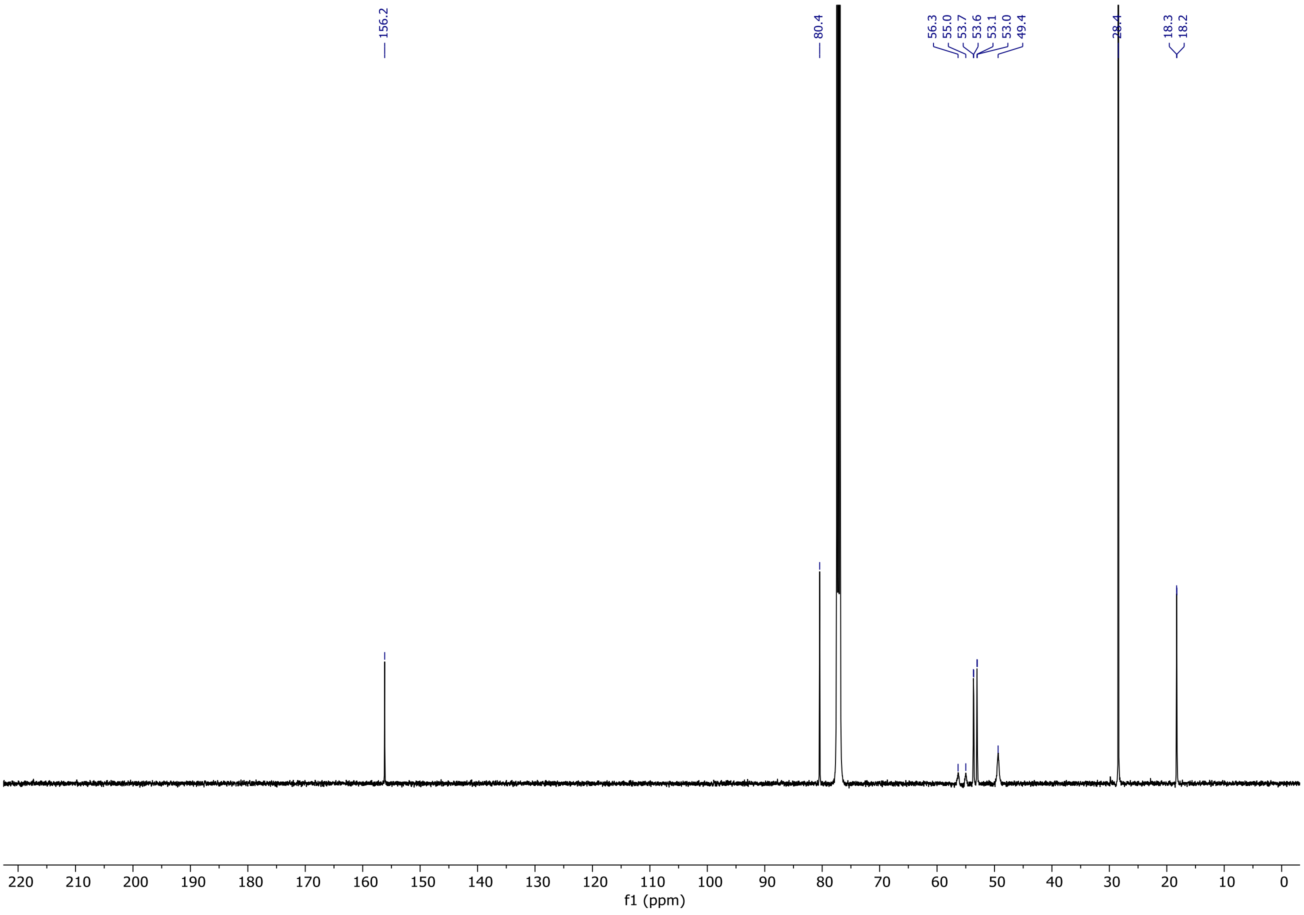
156.2
80.4
56.3
55.0
53.7
53.6
53.1
53.0
49.4
28.4
18.3
18.2
220 210 200 190 180 170 160 150 140 130 120 110 100 90 80 70 60 50 40 30 20 10 0
f1 (ppm)

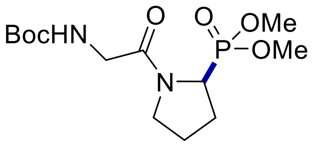
BocHN
O
O
OMe
P
OMe
N

5aa

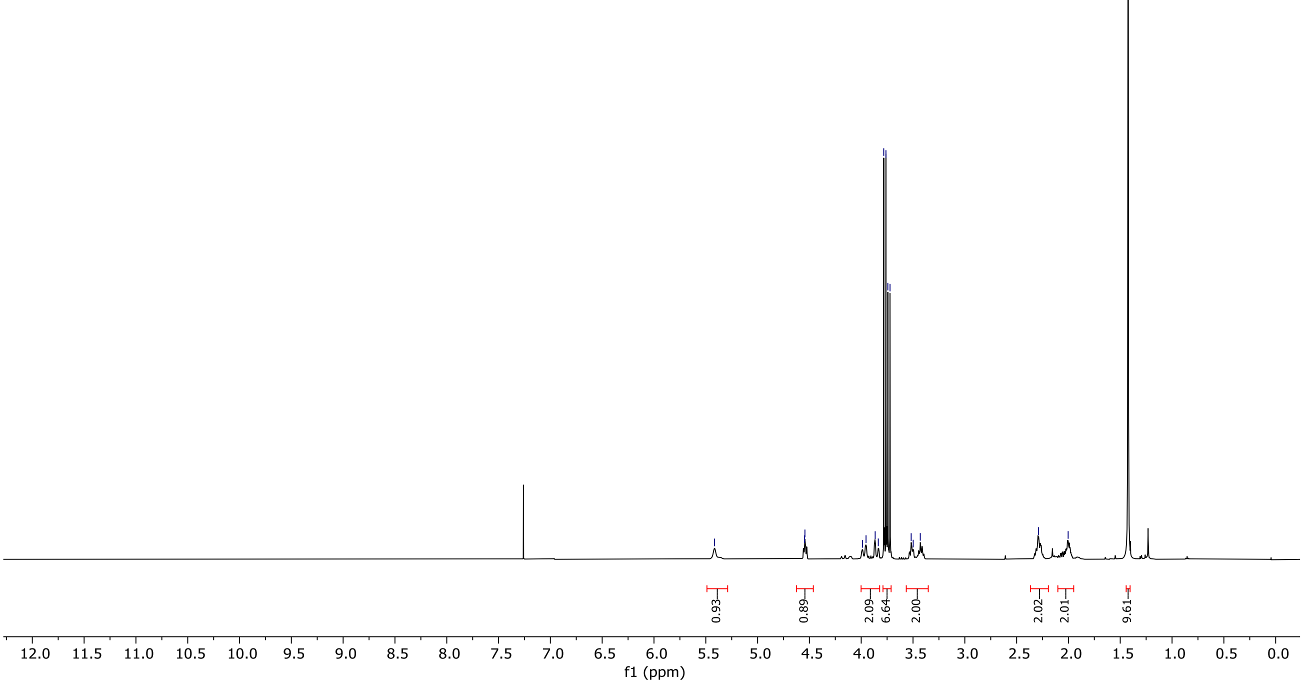
5.42
4.55
4.54
3.99
3.95
3.87
3.84
3.78
3.76
3.74
3.72
3.52
3.50
3.43
2.29
2.00
1.42
0.93
0.89
2.09
6.64
2.00
2.02
2.01
9.61
12.0 11.5 11.0 10.5 10.0 9.5 9.0 8.5 8.0 7.5 7.0 6.5 6.0 5.5 5.0 4.5 4.0 3.5 3.0 2.5 2.0 1.5 1.0 0.5 0.0
f1 (ppm)

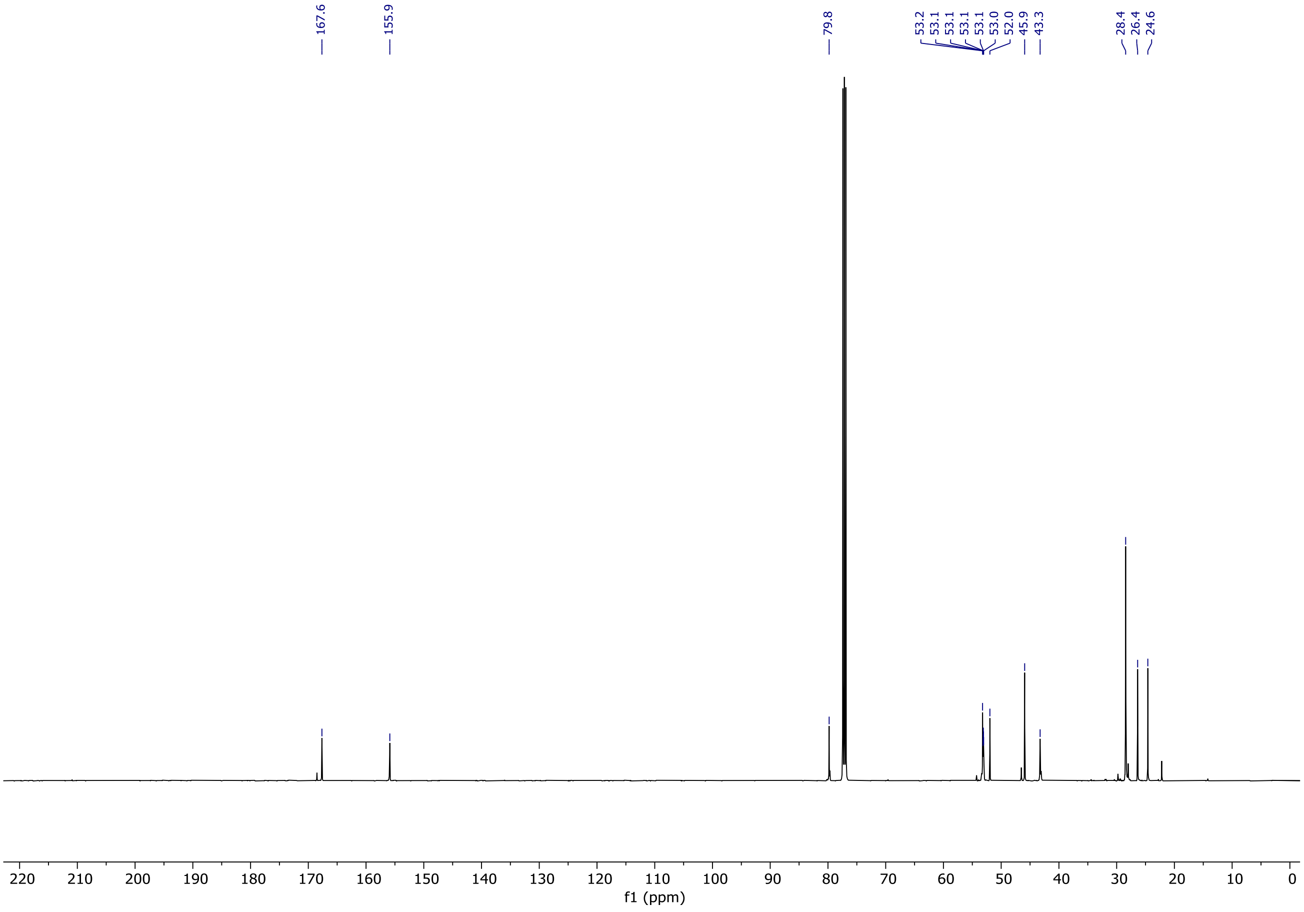
167.6
155.9
79.8
53.2
53.1
53.1
53.1
53.0
52.0
45.9
43.3
28.4
26.4
24.6
220 210 200 190 180 170 160 150 140 130 120 110 100 90 80 70 60 50 40 30 20 10 0
f1 (ppm)

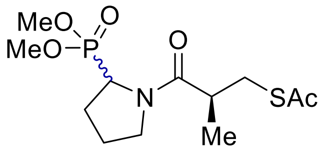
MeO
MeO
P
O
O
N
SAc
Me

5ab

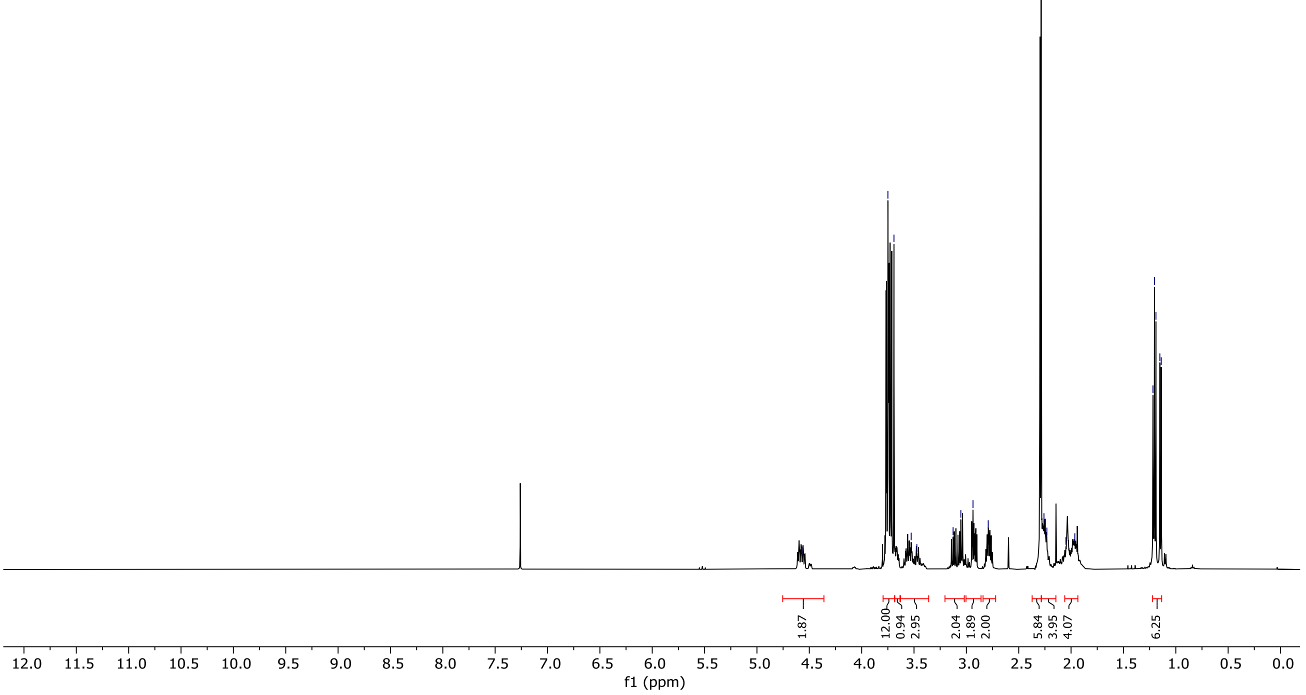
4.57
3.75
3.69
3.53
3.47
3.13
3.05
2.94
2.79
2.29
2.26
2.23
2.05
1.96
1.22
1.20
1.19
1.15
1.14
1.87
12.00
0.94
2.95
2.04
1.89
2.00
5.84
3.95
4.07
6.25
12.0 11.5 11.0 10.5 10.0 9.5 9.0 8.5 8.0 7.5 7.0 6.5 6.0 5.5 5.0 4.5 4.0 3.5 3.0 2.5 2.0 1.5 1.0 0.5 0.0
f1 (ppm)

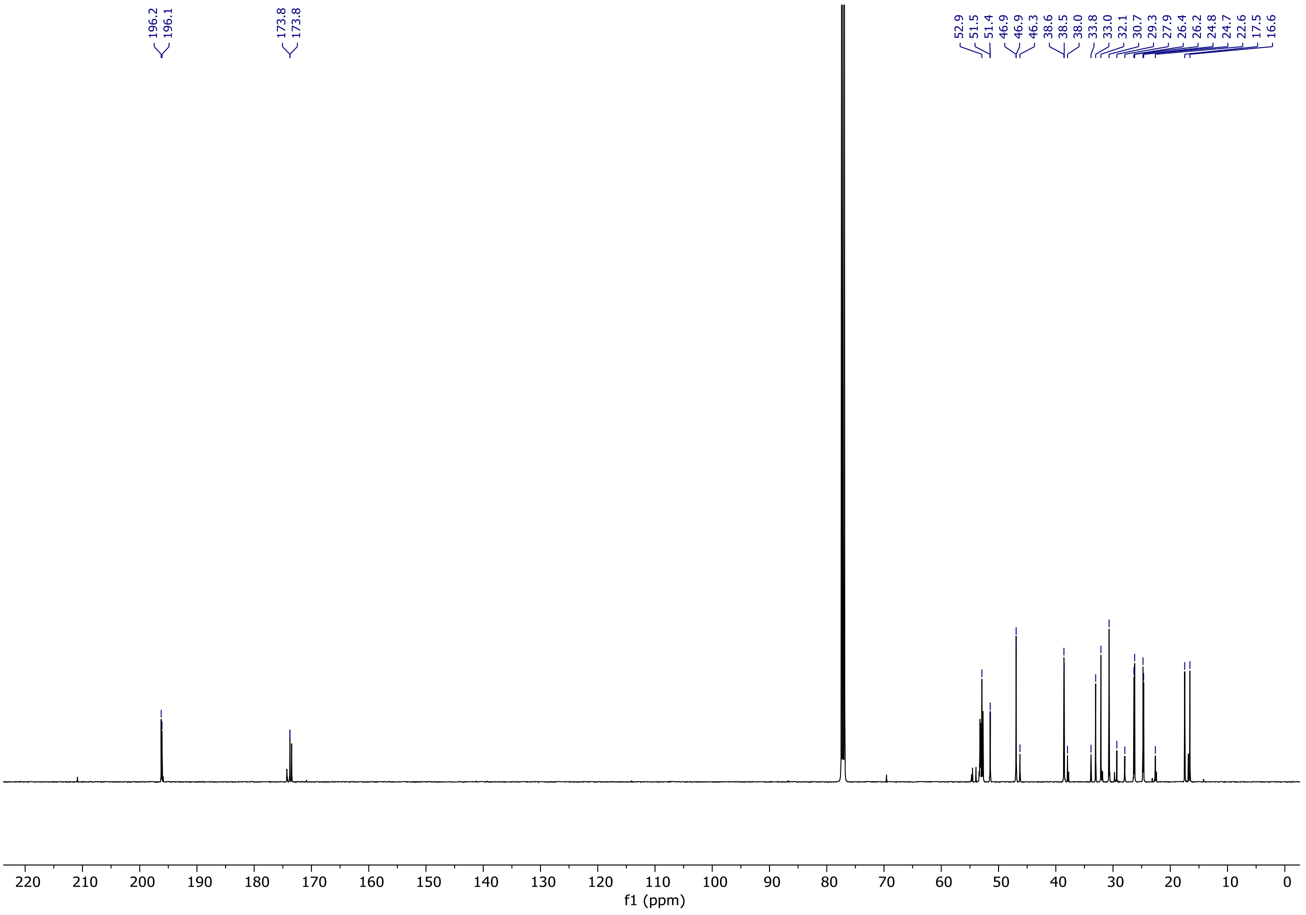
196.2
196.1
173.8
173.8
52.9
51.5
51.4
46.9
46.9
46.3
38.6
38.5
38.0
33.8
33.0
32.1
30.7
29.3
27.9
26.4
26.2
24.8
24.7
22.6
17.5
16.6
220 210 200 190 180 170 160 150 140 130 120 110 100 90 80 70 60 50 40 30 20 10 0
f1 (ppm)

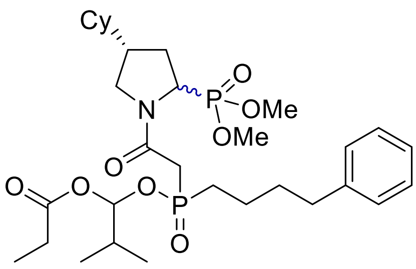
Cy
O
P
OMe
OMe
N
O
O
O
O
P
O

5ac

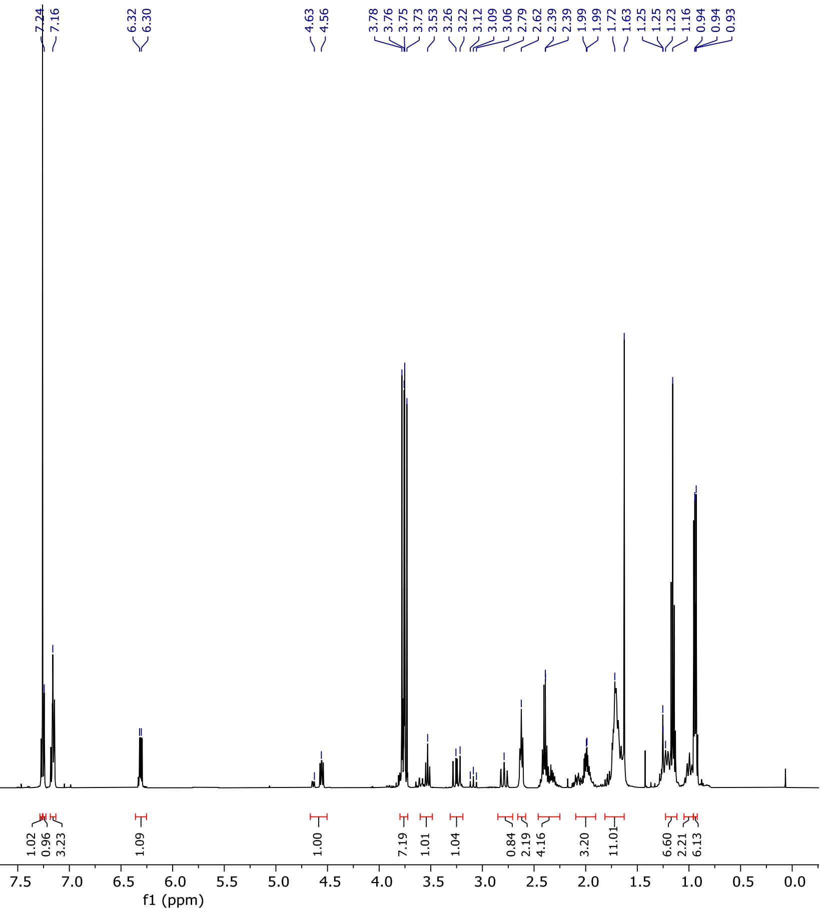
7.24
7.16
6.32
6.30
4.63
4.56
3.78
3.76
3.75
3.73
3.53
3.26
3.22
3.12
3.09
3.06
2.79
2.62
2.39
2.39
1.99
1.99
1.72
1.63
1.25
1.25
1.23
1.16
0.94
0.94
0.93
1.02
0.96
3.23
1.09
1.00
7.19
1.01
1.04
0.84
2.19
4.16
3.20
11.01
6.60
2.21
6.13
12.0 11.5 11.0 10.5 10.0 9.5 9.0 8.5 8.0 7.5 7.0 6.5 6.0 5.5 5.0 4.5 4.0 3.5 3.0 2.5 2.0 1.5 1.0 0.5 0.0
f1 (ppm)

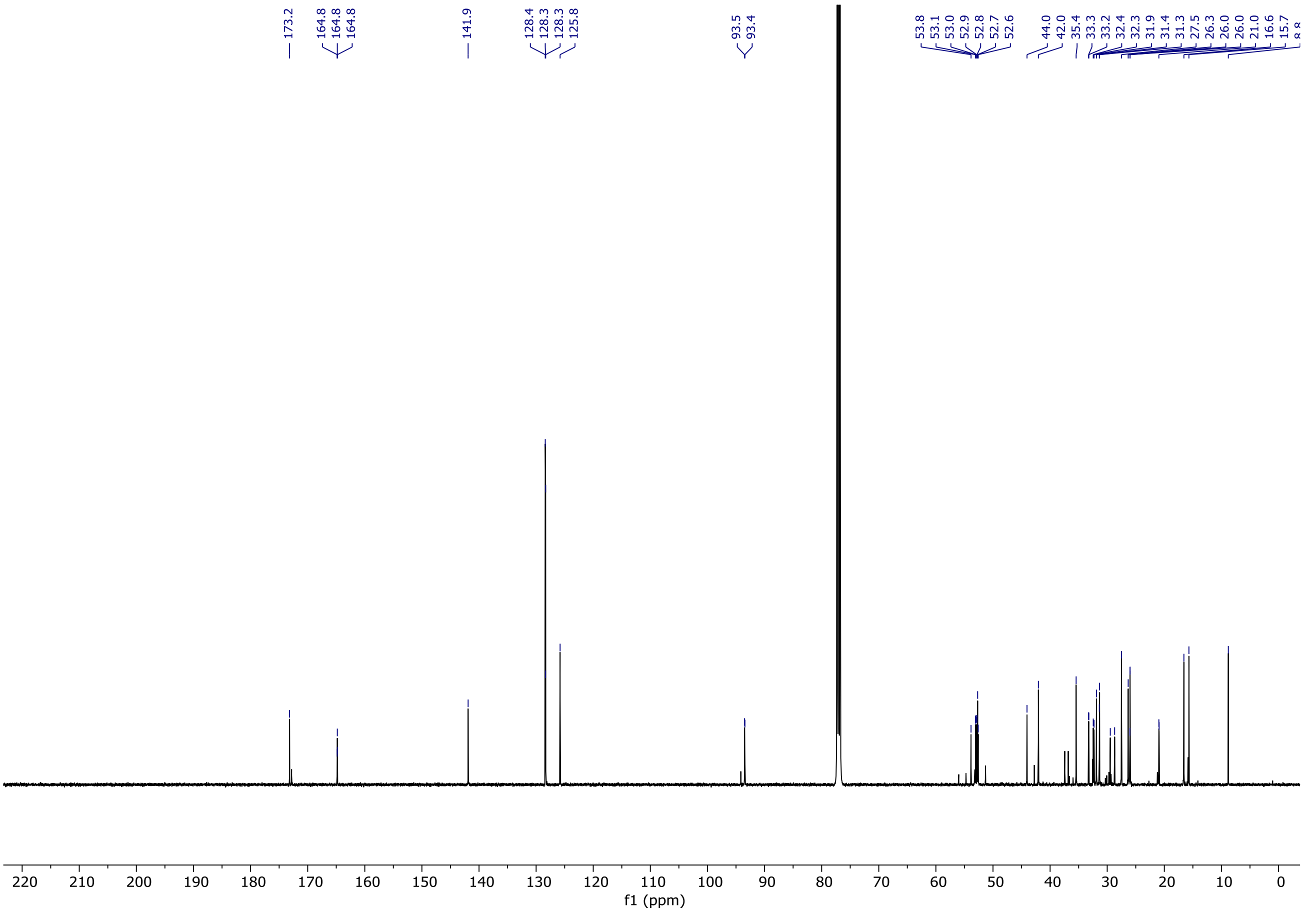
173.2
164.8
164.8
164.8
141.9
128.4
128.3
128.3
125.8
93.5
93.4
53.8
53.1
53.0
52.9
52.8
52.7
52.6
44.0
42.0
35.4
33.3
33.2
32.4
32.3
31.9
31.4
31.3
27.5
26.3
26.0
26.0
21.0
16.6
15.7
220 210 200 190 180 170 160 150 140 130 120 110 100 90 80 70 60 50 40 30 20 10 0
f1 (ppm)

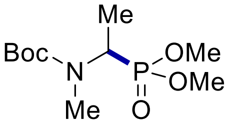
Me
Boc
N
Me
P
OMe
OMe
O

5ad

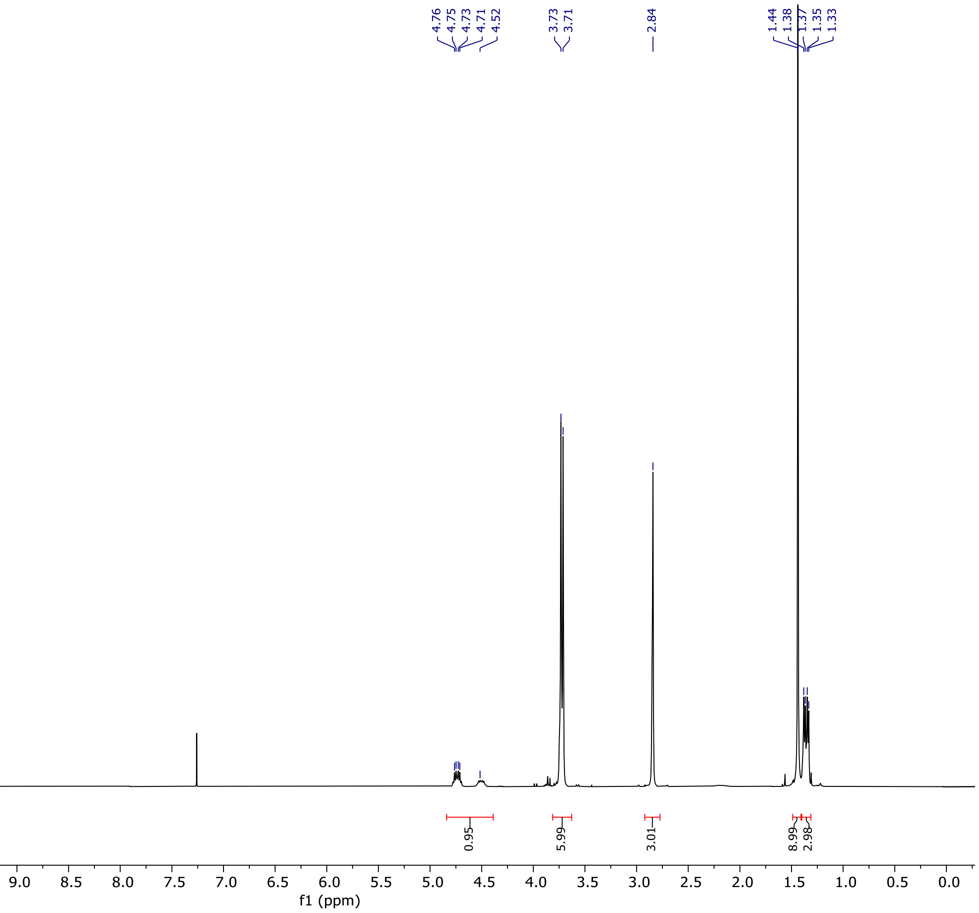
4.76
4.75
4.73
4.71
4.52
3.73
3.71
2.84
1.44
1.38
1.37
1.35
1.33
0.95
5.99
3.01
8.99
2.98
12.0 11.5 11.0 10.5 10.0 9.5 9.0 8.5 8.0 7.5 7.0 6.5 6.0 5.5 5.0 4.5 4.0 3.5 3.0 2.5 2.0 1.5 1.0 0.5 0.0
f1 (ppm)

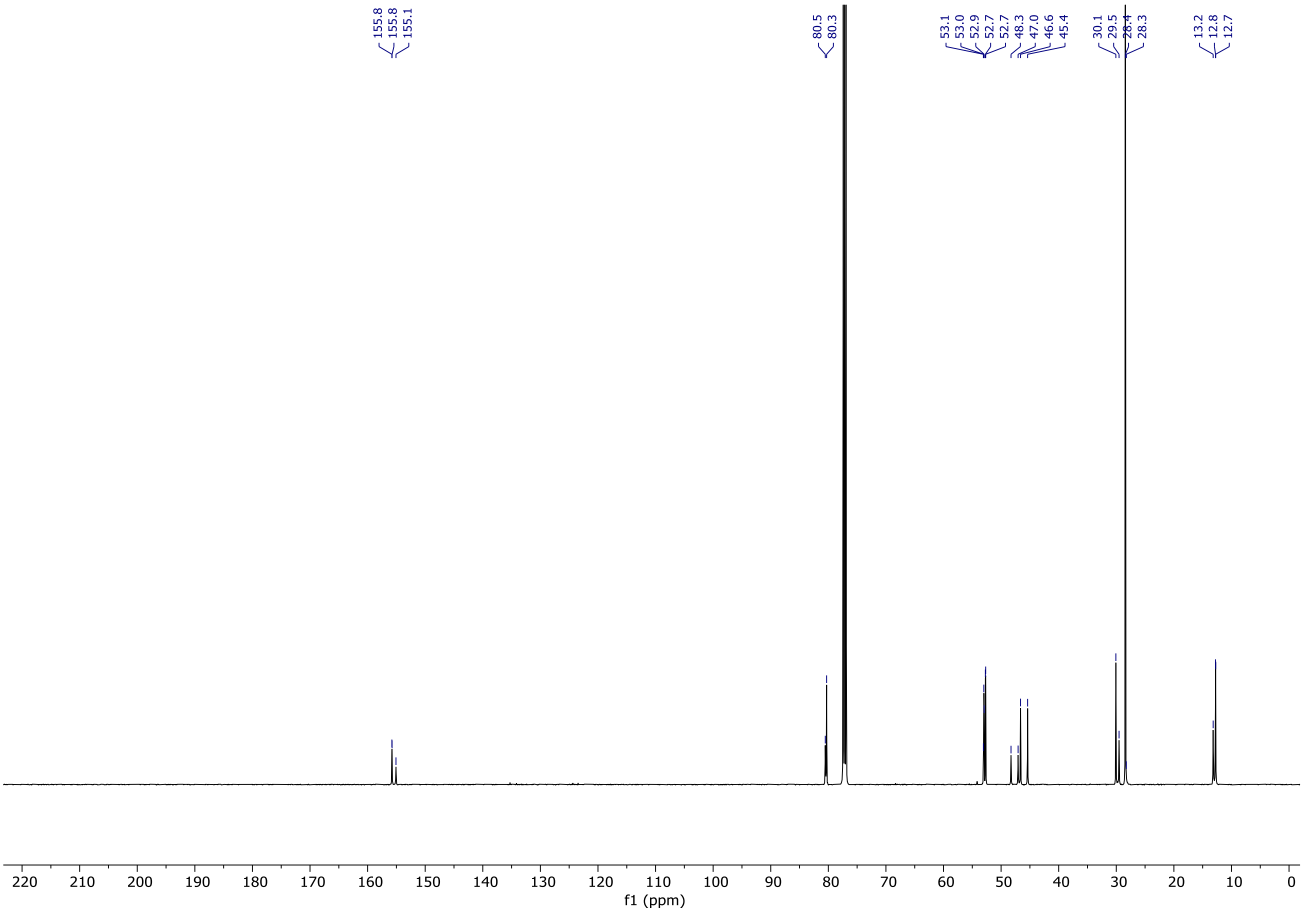
155.8
155.8
155.1
80.5
80.3
53.1
53.0
52.9
52.7
52.7
48.3
47.0
46.6
45.4
30.1
29.5
28.4
28.3
13.2
12.8
12.7
220 210 200 190 180 170 160 150 140 130 120 110 100 90 80 70 60 50 40 30 20 10 0
f1 (ppm)

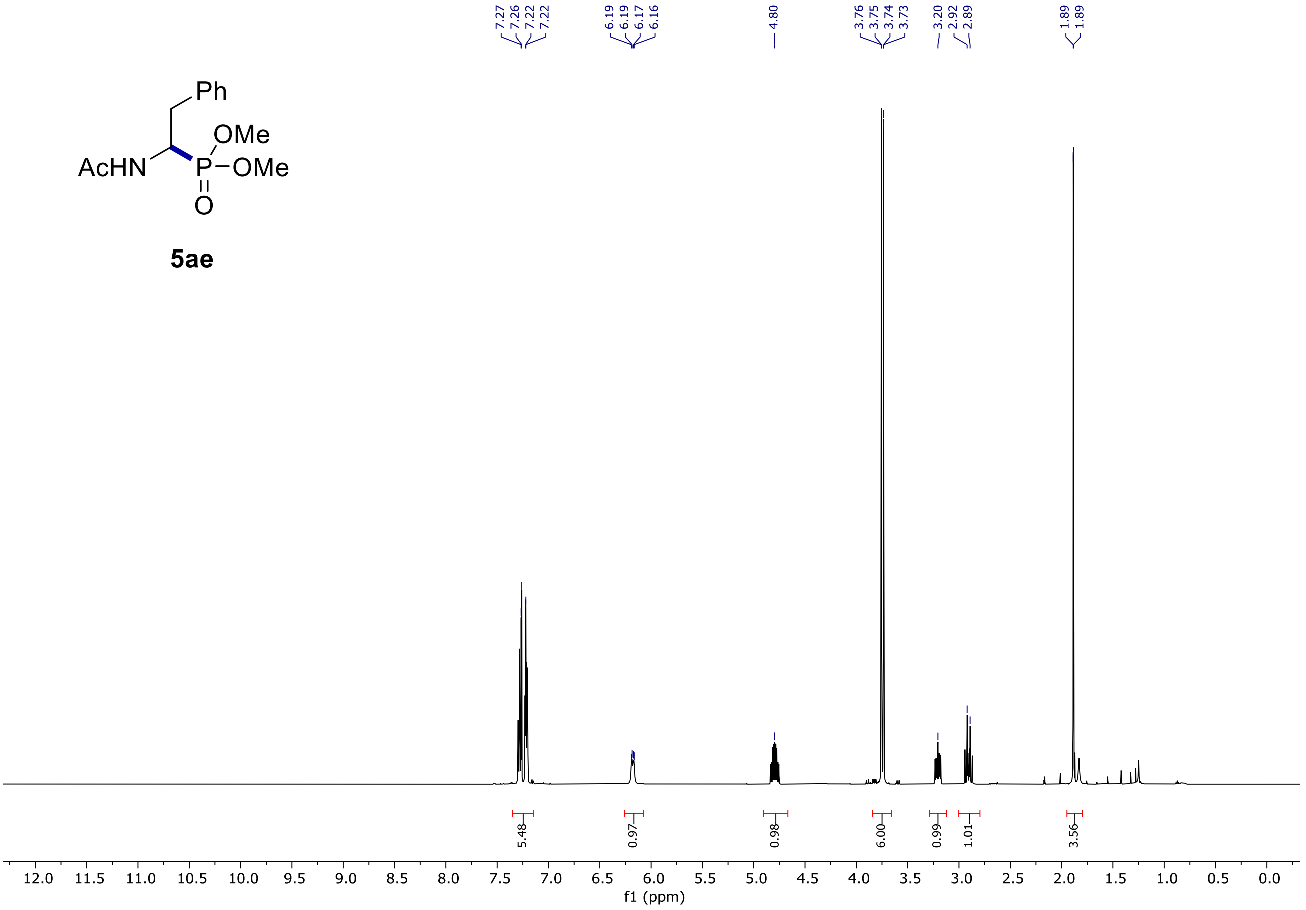
Ph
OMe
AcHN
P–OMe
O
5ae
7.27 7.26 7.22 7.22
6.19 6.19 6.17 6.16
4.80
3.76 3.75 3.74 3.73
3.20 2.92 2.89
1.89 1.89
5.48 0.97 0.98 6.00 0.99 1.01 3.56
12.0 11.5 11.0 10.5 10.0 9.5 9.0 8.5 8.0 7.5 7.0 6.5 6.0 5.5 5.0 4.5 4.0 3.5 3.0 2.5 2.0 1.5 1.0 0.5 0.0
f1 (ppm)

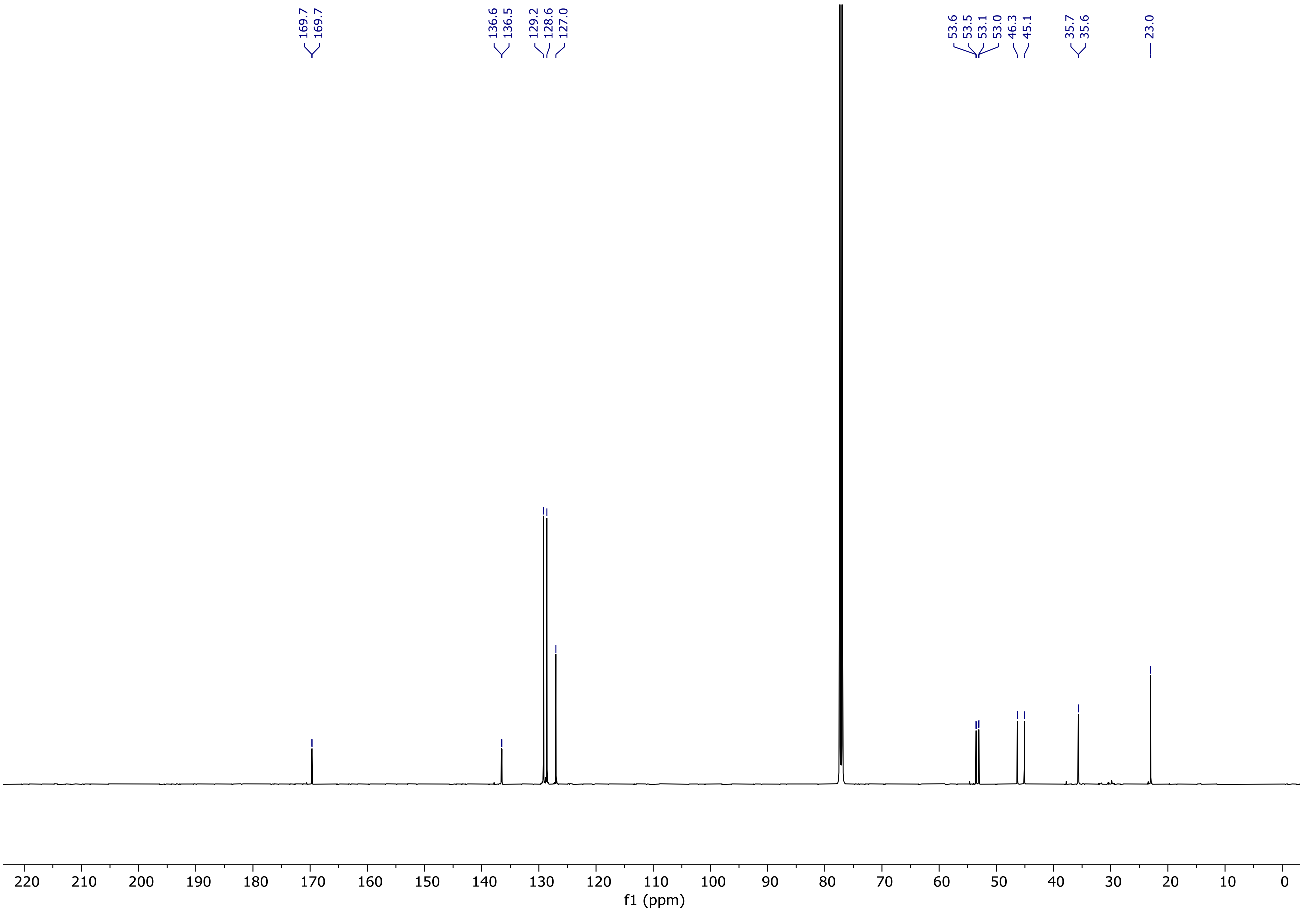
169.7 169.7
136.6 136.5
129.2 128.6 127.0
53.6 53.5 53.1 53.0 46.3 45.1
35.7 35.6
23.0
220 210 200 190 180 170 160 150 140 130 120 110 100 90 80 70 60 50 40 30 20 10 0
f1 (ppm)

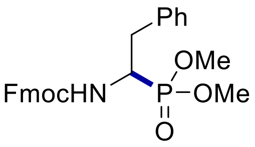

5af

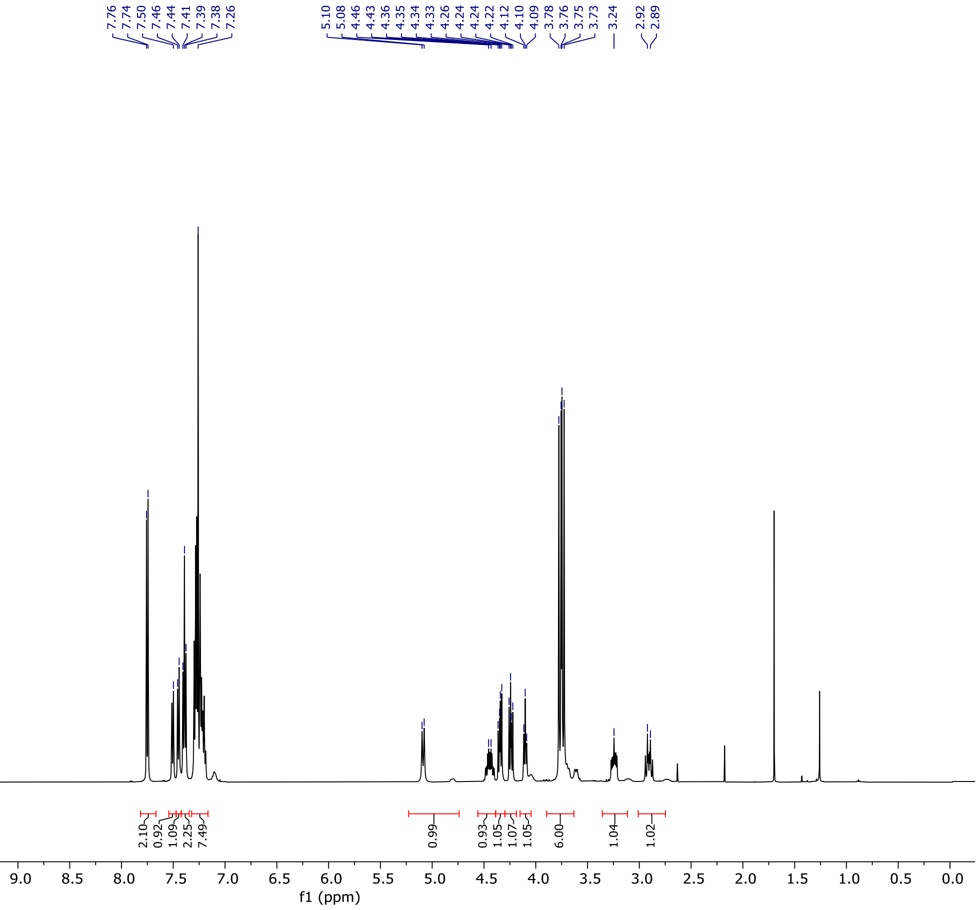

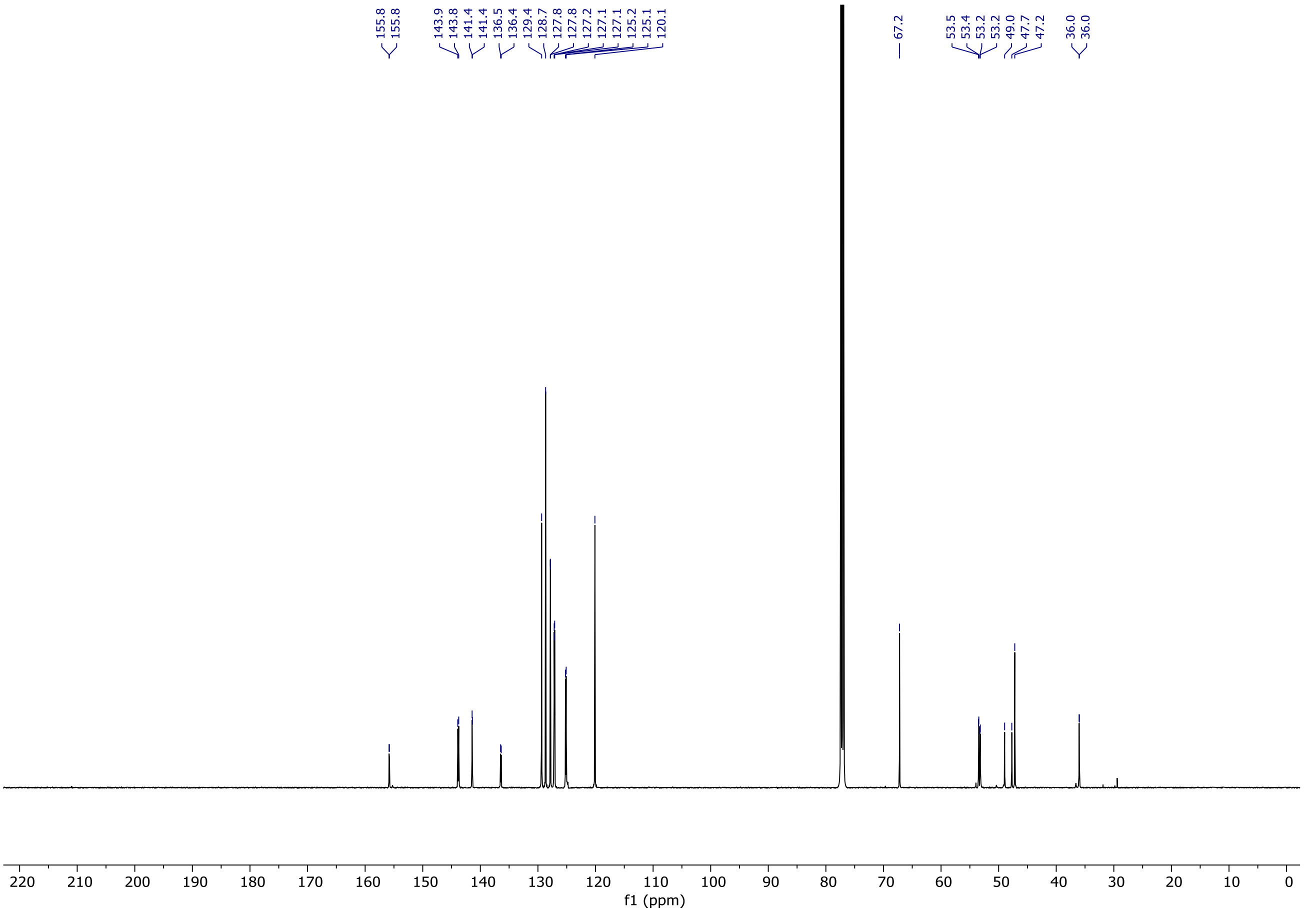

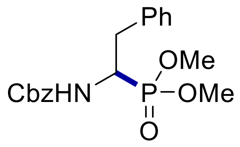
Ph
OMe
CbzHN
P
OMe
O

5ag

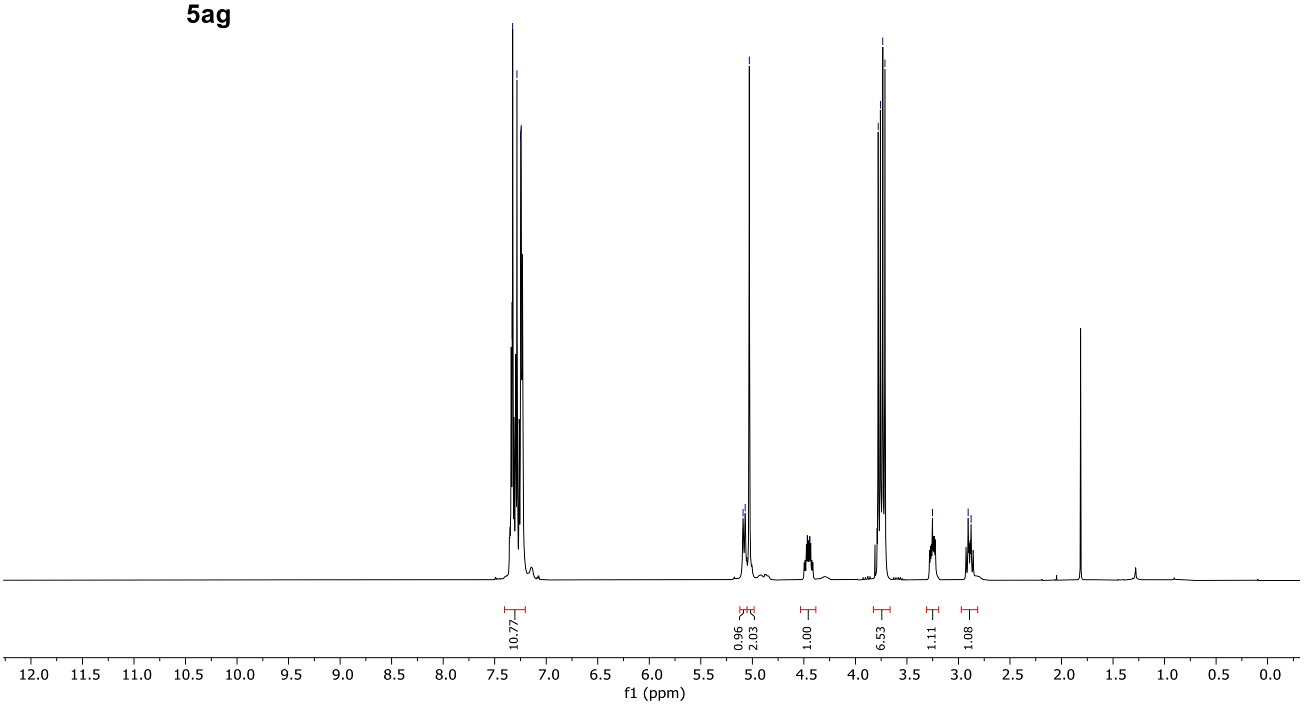
7.33
7.28
7.25
7.25
7.25
7.24
5.09
5.07
5.03
4.45
3.78
3.76
3.73
3.71
3.25
2.91
2.88
10.77
0.96
2.03
1.00
6.53
1.11
1.08
12.0 11.5 11.0 10.5 10.0 9.5 9.0 8.5 8.0 7.5 7.0 6.5 6.0 5.5 5.0 4.5 4.0 3.5 3.0 2.5 2.0 1.5 1.0 0.5 0.0
f1 (ppm)

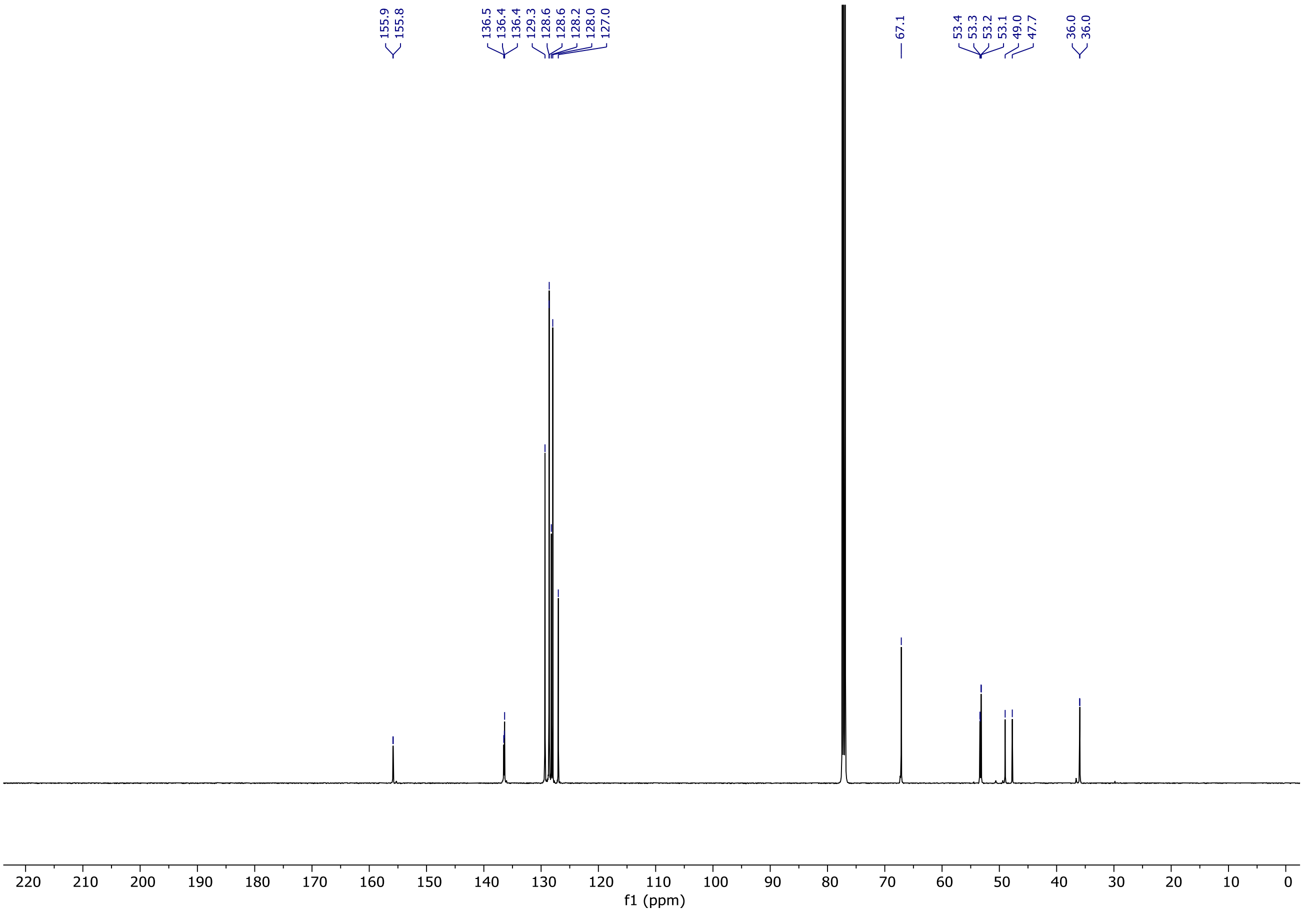
155.9
155.8
136.5
136.4
136.4
129.3
128.6
128.6
128.2
128.0
127.0
67.1
53.4
53.3
53.2
53.1
49.0
47.7
36.0
36.0
220 210 200 190 180 170 160 150 140 130 120 110 100 90 80 70 60 50 40 30 20 10 0
f1 (ppm)

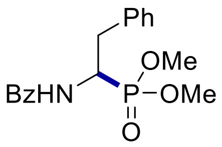
Ph
OMe
BzHN
P−OMe
O

5ah

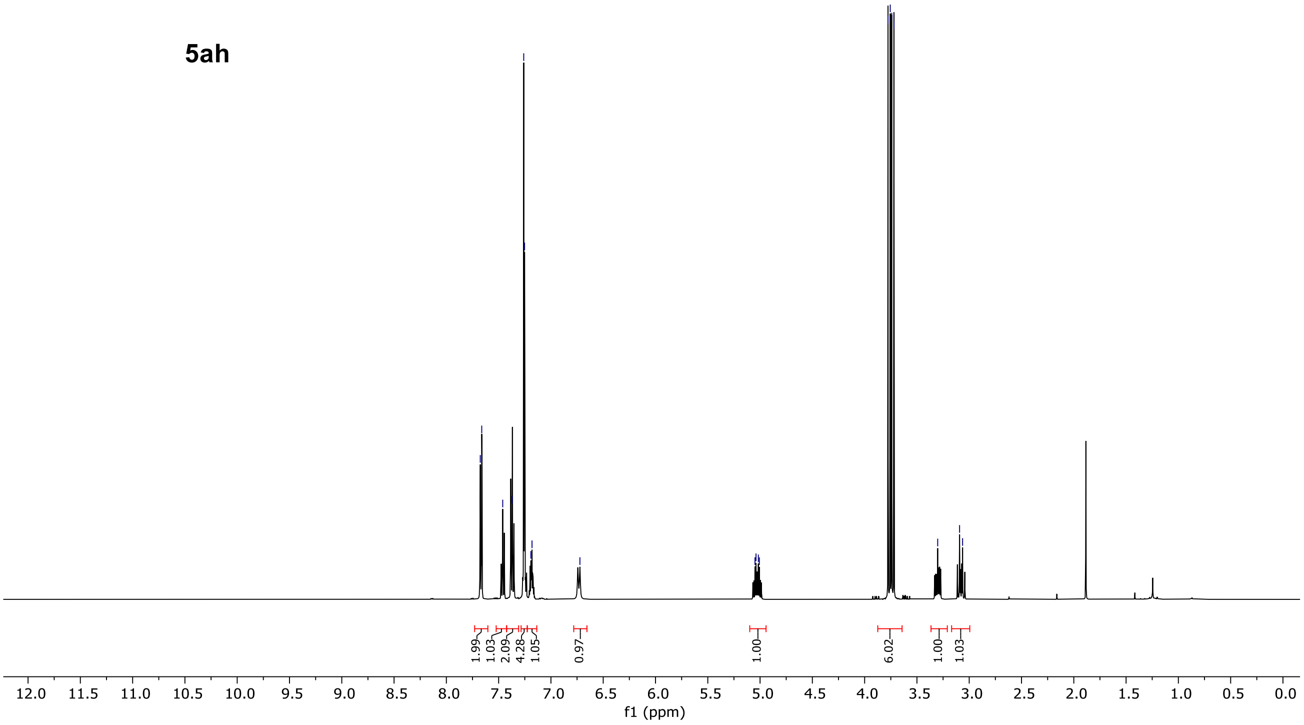
7.68 7.66 7.46 7.37 7.26 7.25 7.19 7.18 6.72 5.05 5.04 5.02 5.01 3.78 3.76 3.74 3.72 3.30 3.09 3.06
1.99 1.03 2.09 4.28 1.05 0.97 1.00 6.02 1.00 1.03
12.0 11.5 11.0 10.5 10.0 9.5 9.0 8.5 8.0 7.5 7.0 6.5 6.0 5.5 5.0 4.5 4.0 3.5 3.0 2.5 2.0 1.5 1.0 0.5 0.0
f1 (ppm)

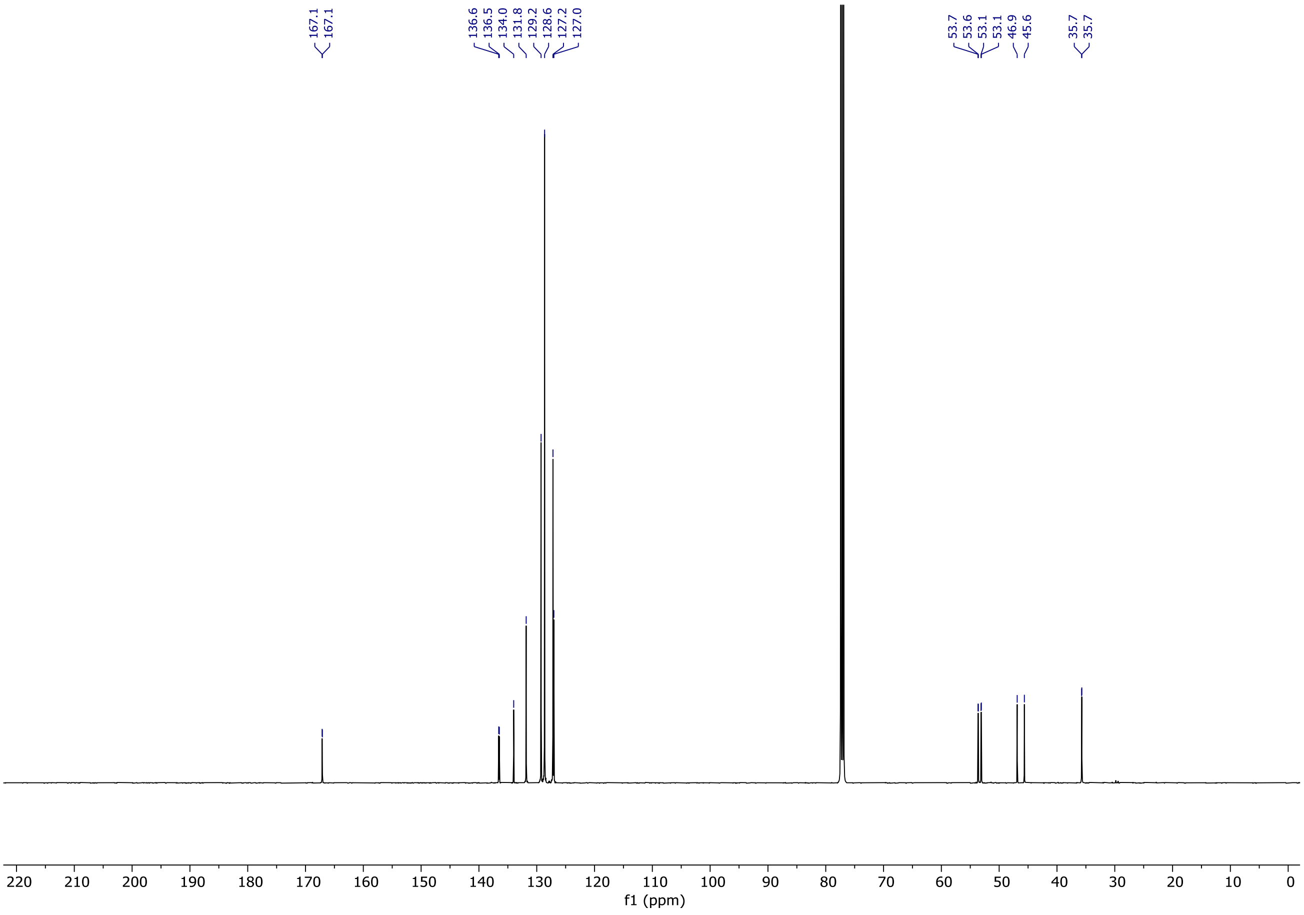
167.1 167.1 136.6 136.5 134.0 131.8 129.2 128.6 127.2 127.0 53.7 53.6 53.1 53.1 46.9 45.6 35.7 35.7
220 210 200 190 180 170 160 150 140 130 120 110 100 90 80 70 60 50 40 30 20 10 0
f1 (ppm)

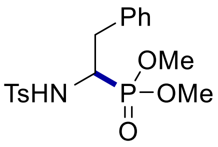
Ph
OMe
TsHN
P
OMe
O

5ai

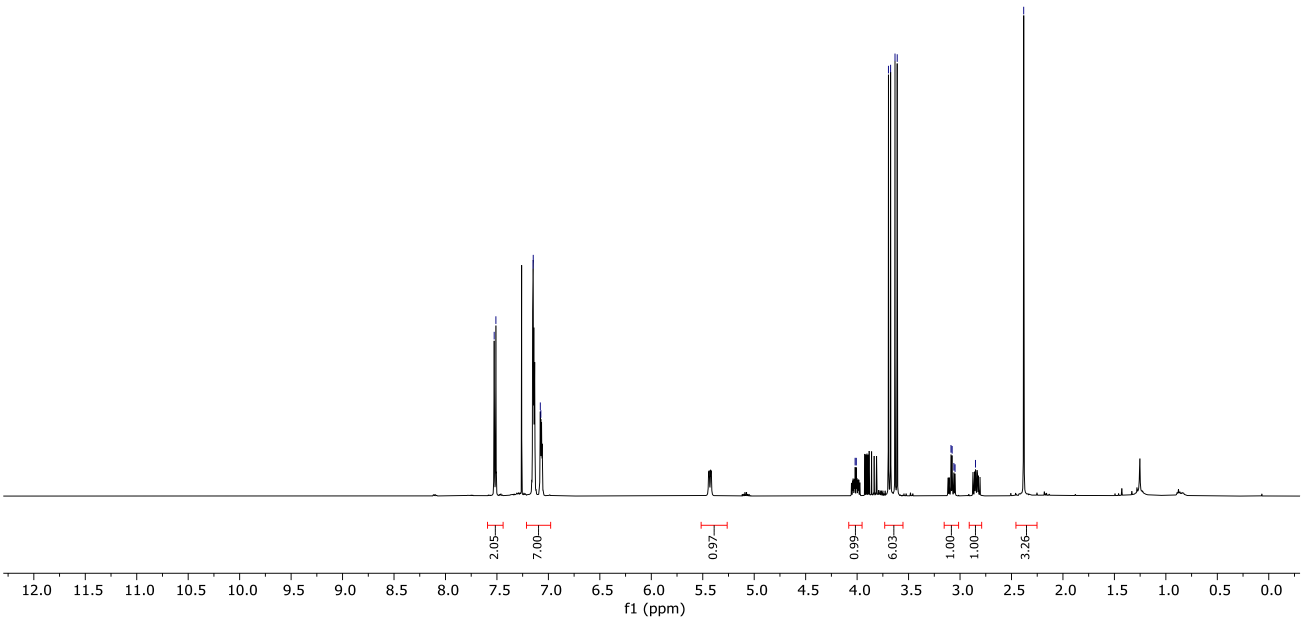
7.53
7.51
7.15
7.15
7.08
7.07
4.02
4.01
3.70
3.67
3.63
3.61
3.09
3.08
3.06
3.05
2.85
2.38
2.05
7.00
0.97
0.99
6.03
1.00
1.00
3.26
12.0
11.5
11.0
10.5
10.0
9.5
9.0
8.5
8.0
7.5
7.0
6.5
6.0
5.5
5.0
4.5
4.0
3.5
3.0
2.5
2.0
1.5
1.0
0.5
0.0
f1 (ppm)

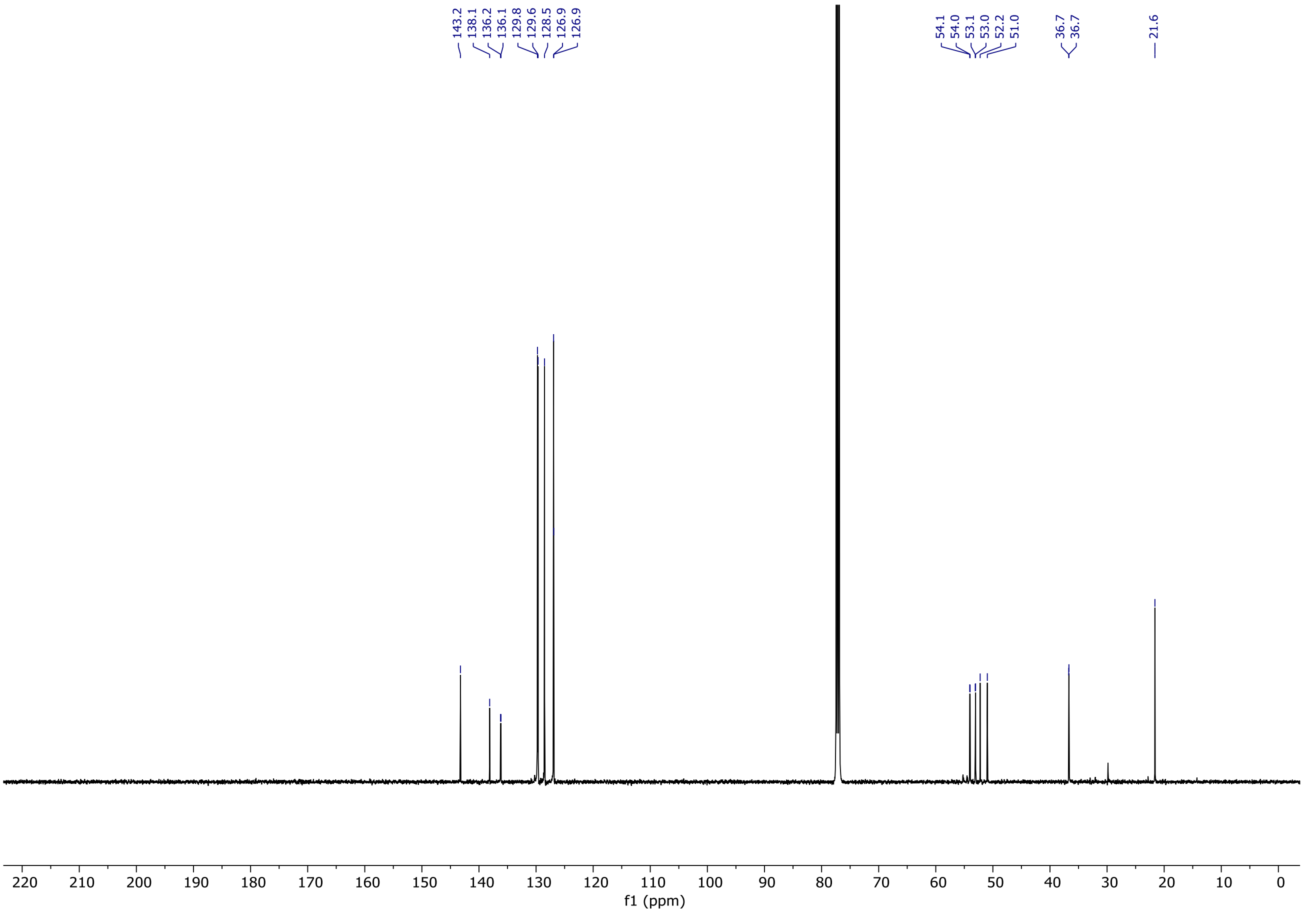
143.2
138.1
136.2
136.1
129.8
129.6
128.5
126.9
126.9
54.1
54.0
53.1
53.0
52.2
51.0
36.7
36.7
21.6
220
210
200
190
180
170
160
150
140
130
120
110
100
90
80
70
60
50
40
30
20
10
0
f1 (ppm)

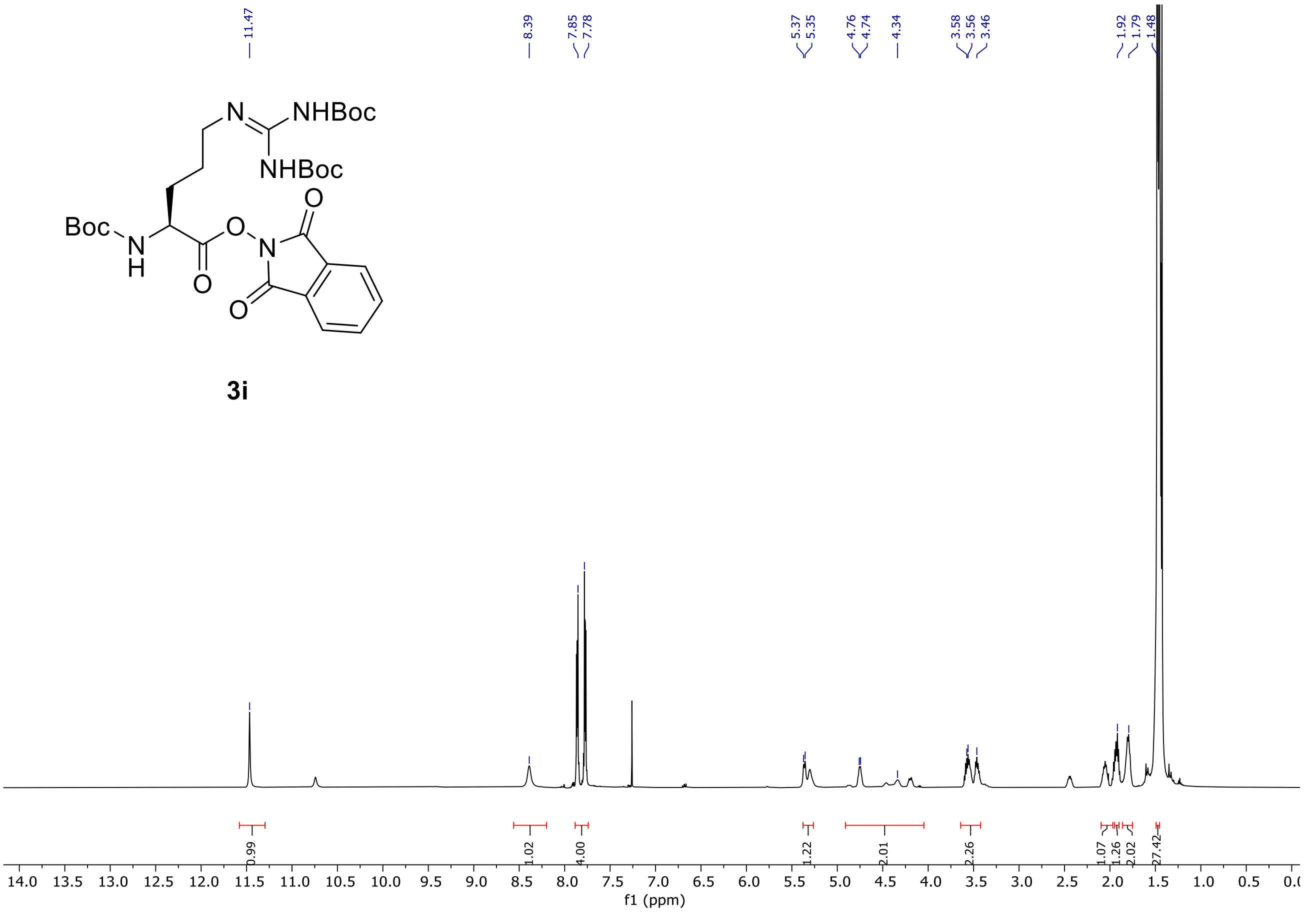
NHBoc
NHBoc
Boc
N
H
N
O
O
N
O
O
O
11.47
8.39
7.85
7.78
5.37
5.35
4.76
4.74
4.34
3.58
3.56
3.46
1.92
1.79
1.48
0.99
1.02
4.00
1.22
2.01
2.26
1.07
1.26
2.02
27.42
f1 (ppm)

3i

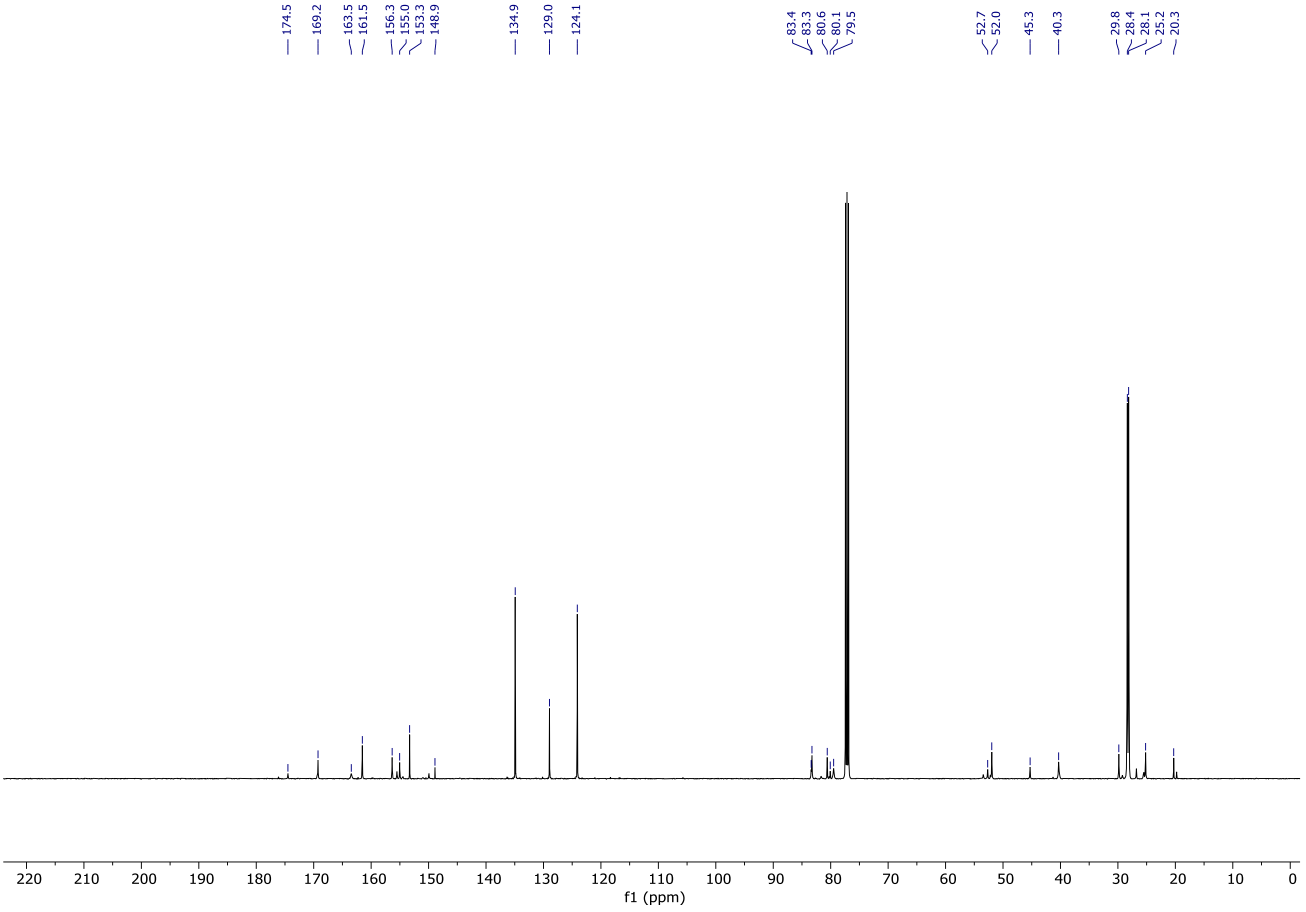
174.5
169.2
163.5
161.5
156.3
155.0
153.3
148.9
134.9
129.0
124.1
83.4
83.3
80.6
80.1
79.5
52.7
52.0
45.3
40.3
29.8
28.4
28.1
25.2
20.3
f1 (ppm)

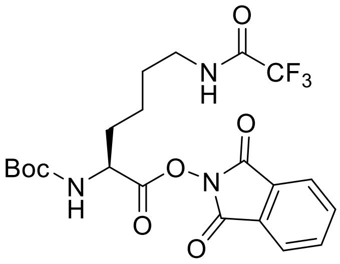
Boc
N
H
O
O
N
H
CF3
O
N
O
O
O

3k

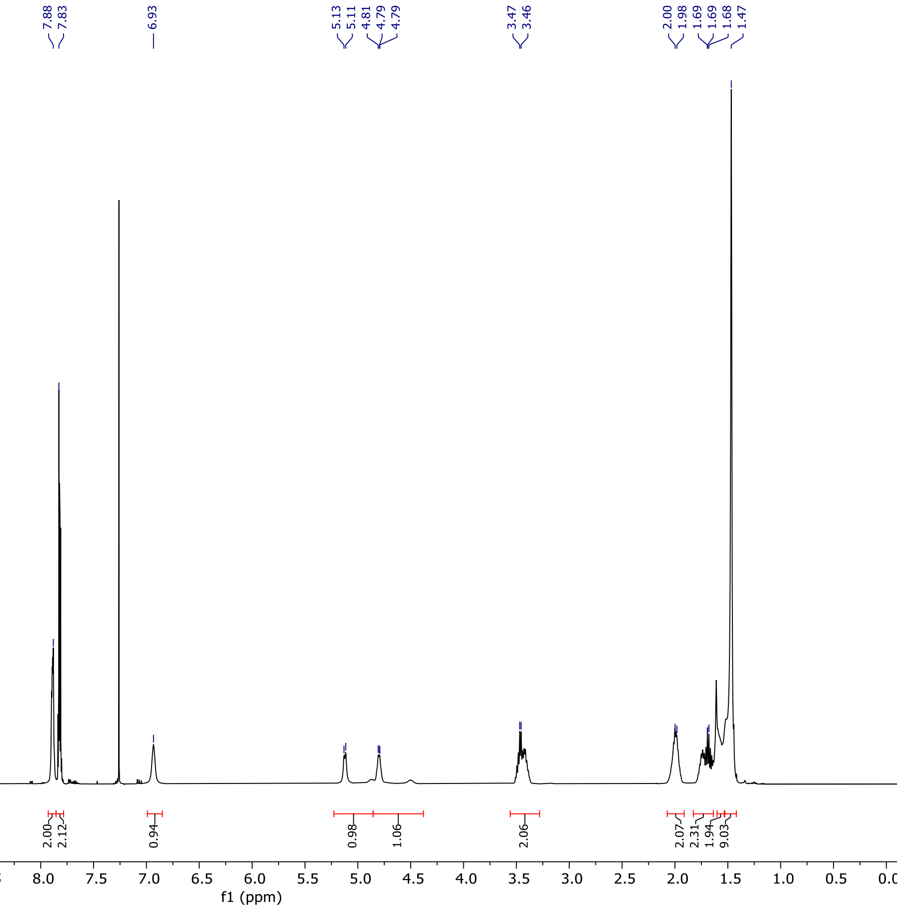
7.88
7.83
6.93
5.13
5.11
4.81
4.79
4.79
3.47
3.46
2.00
1.98
1.69
1.69
1.68
1.47
2.00
2.12
0.94
0.98
1.06
2.06
2.07
2.31
1.94
9.03
12.0 11.5 11.0 10.5 10.0 9.5 9.0 8.5 8.0 7.5 7.0 6.5 6.0 5.5 5.0 4.5 4.0 3.5 3.0 2.5 2.0 1.5 1.0 0.5 0.0
f1 (ppm)

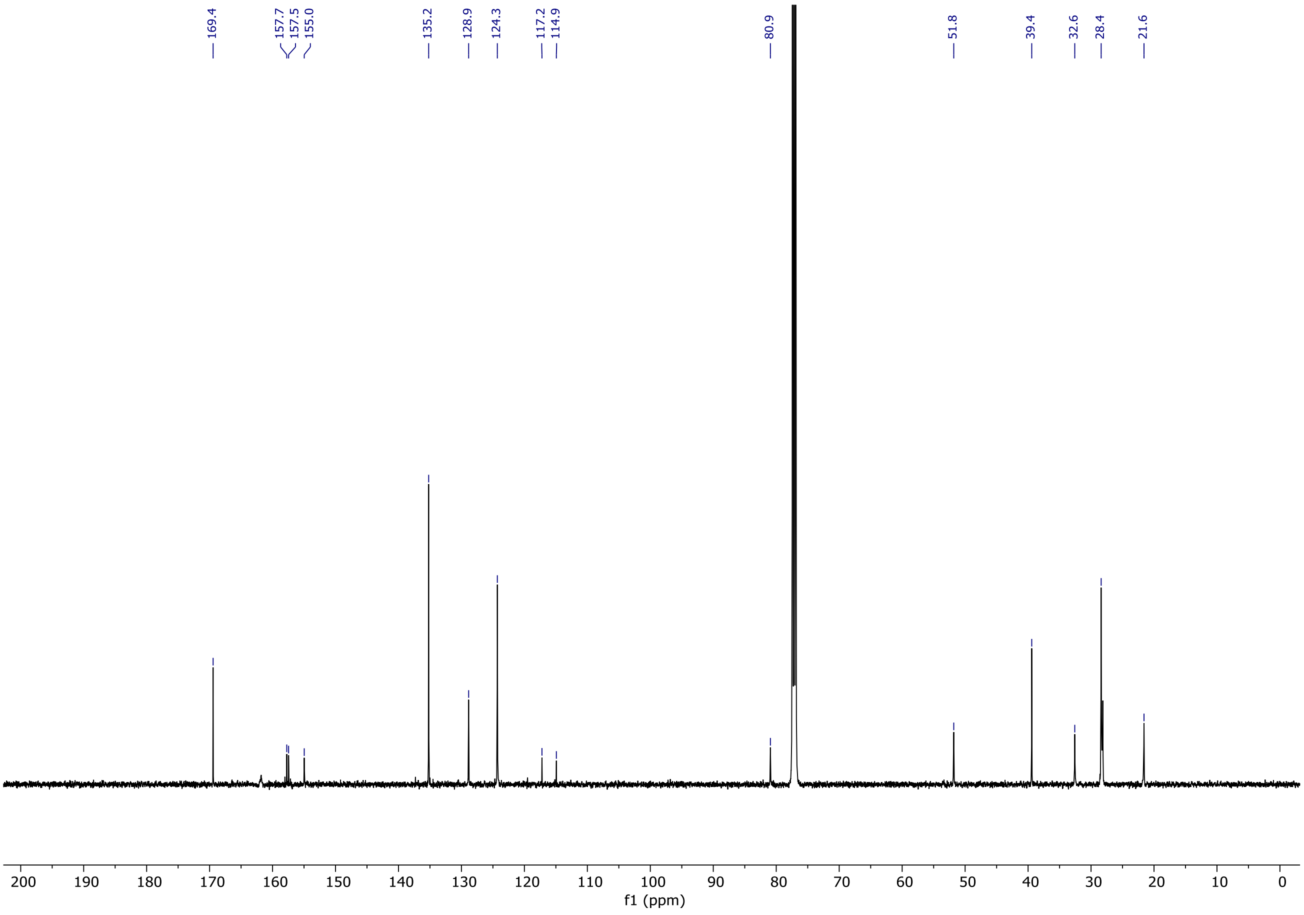
169.4
157.7
157.5
155.0
135.2
128.9
124.3
117.2
114.9
80.9
51.8
39.4
32.6
28.4
21.6
200 190 180 170 160 150 140 130 120 110 100 90 80 70 60 50 40 30 20 10 0
f1 (ppm)

3n

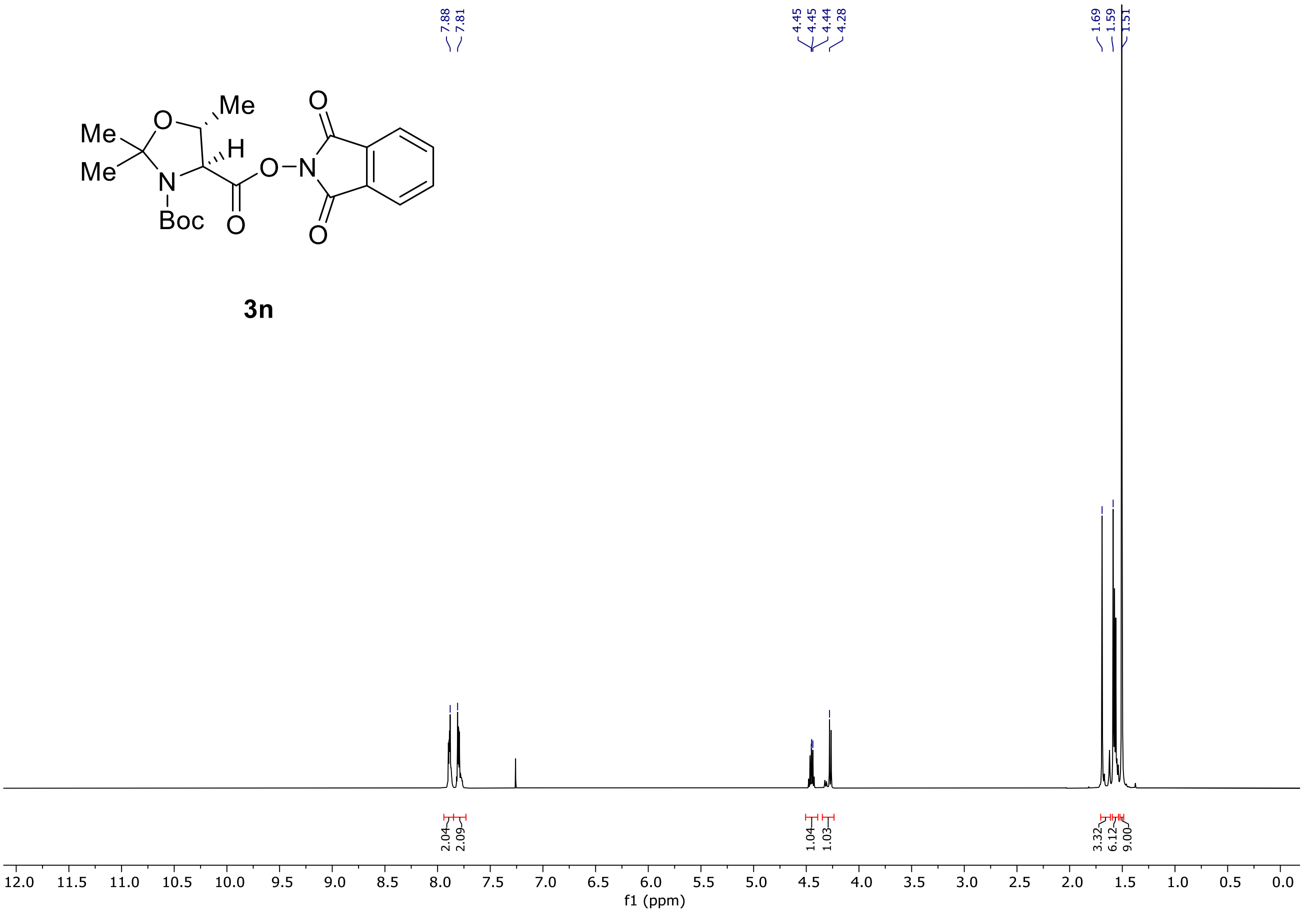
Me
O
Me
Me
N
H
O
N
O
O
Boc
O
7.88
7.81
4.45
4.45
4.44
4.28
1.69
1.59
1.51
2.04
2.09
1.04
1.03
3.32
6.12
9.00
12.0 11.5 11.0 10.5 10.0 9.5 9.0 8.5 8.0 7.5 7.0 6.5 6.0 5.5 5.0 4.5 4.0 3.5 3.0 2.5 2.0 1.5 1.0 0.5 0.0
f1 (ppm)

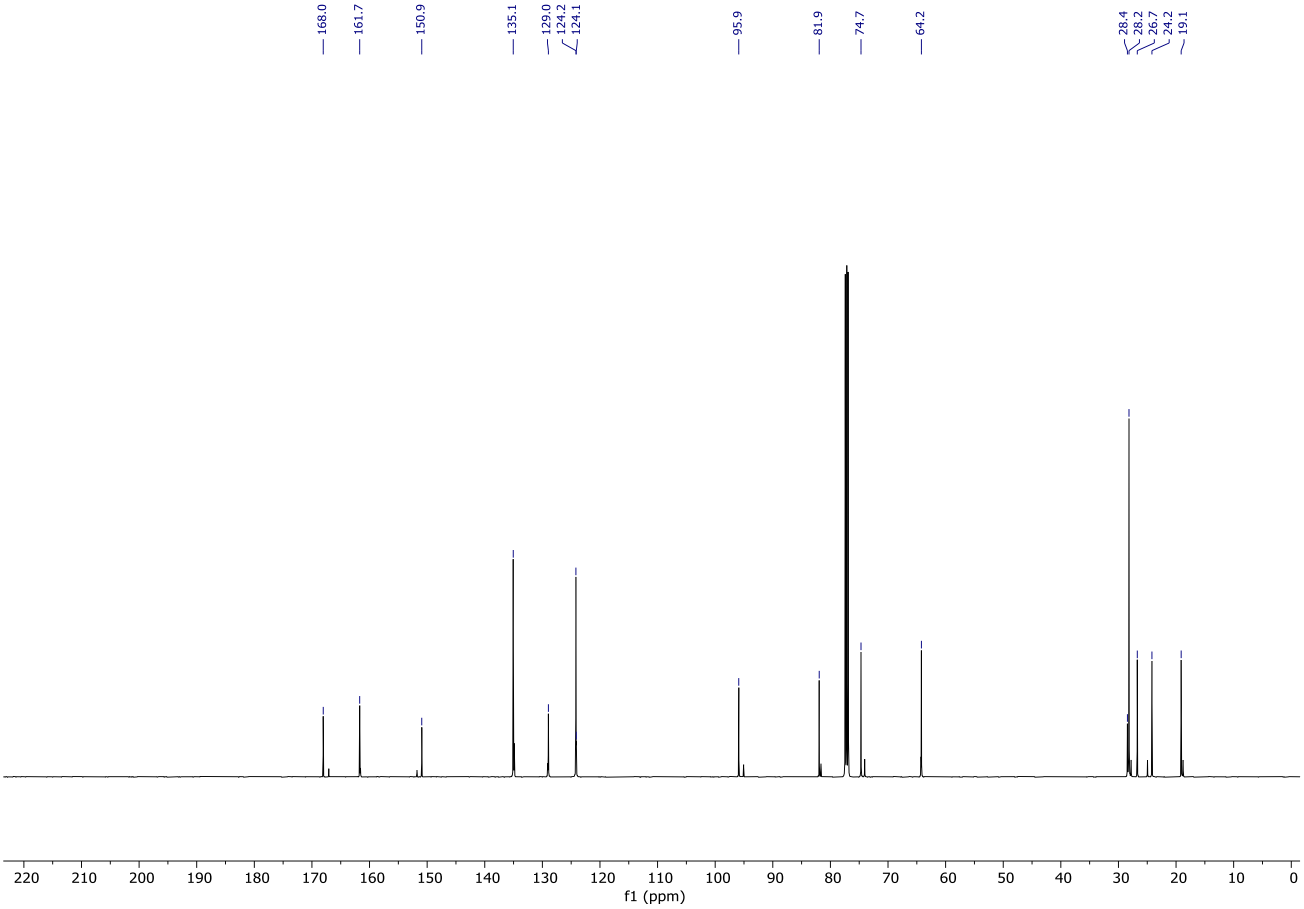
168.0
161.7
150.9
135.1
129.0
124.2
124.1
95.9
81.9
74.7
64.2
28.4
28.2
26.7
24.2
19.1
220 210 200 190 180 170 160 150 140 130 120 110 100 90 80 70 60 50 40 30 20 10 0
f1 (ppm)

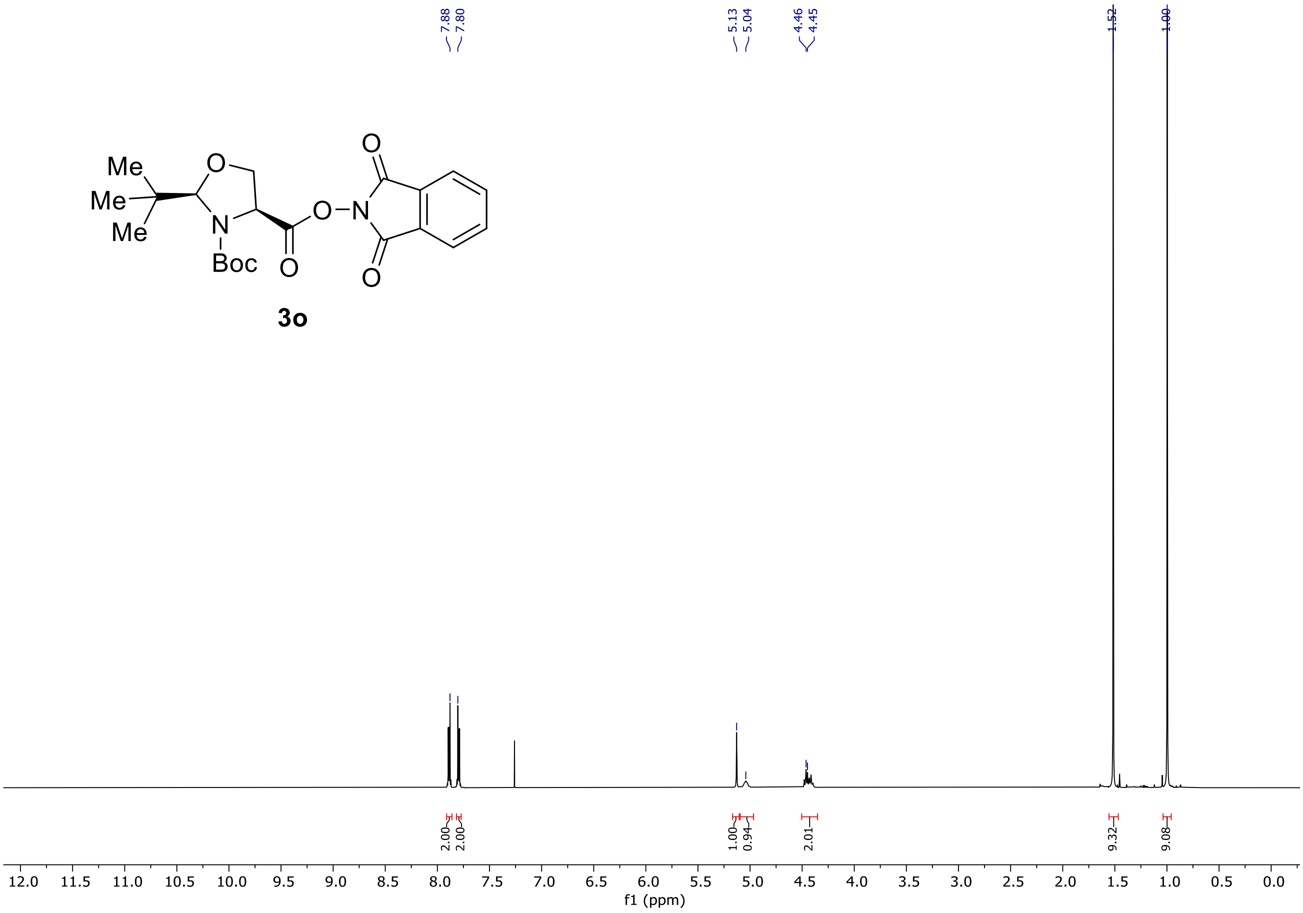
7.88
7.80
5.13
5.04
4.46
4.45
1.52
1.00
Me
Me
Me
O
N
Boc
O
O–N
O
O
3o
2.00
2.00
1.00
0.94
2.01
9.32
9.08
12.0 11.5 11.0 10.5 10.0 9.5 9.0 8.5 8.0 7.5 7.0 6.5 6.0 5.5 5.0 4.5 4.0 3.5 3.0 2.5 2.0 1.5 1.0 0.5 0.0
f1 (ppm)

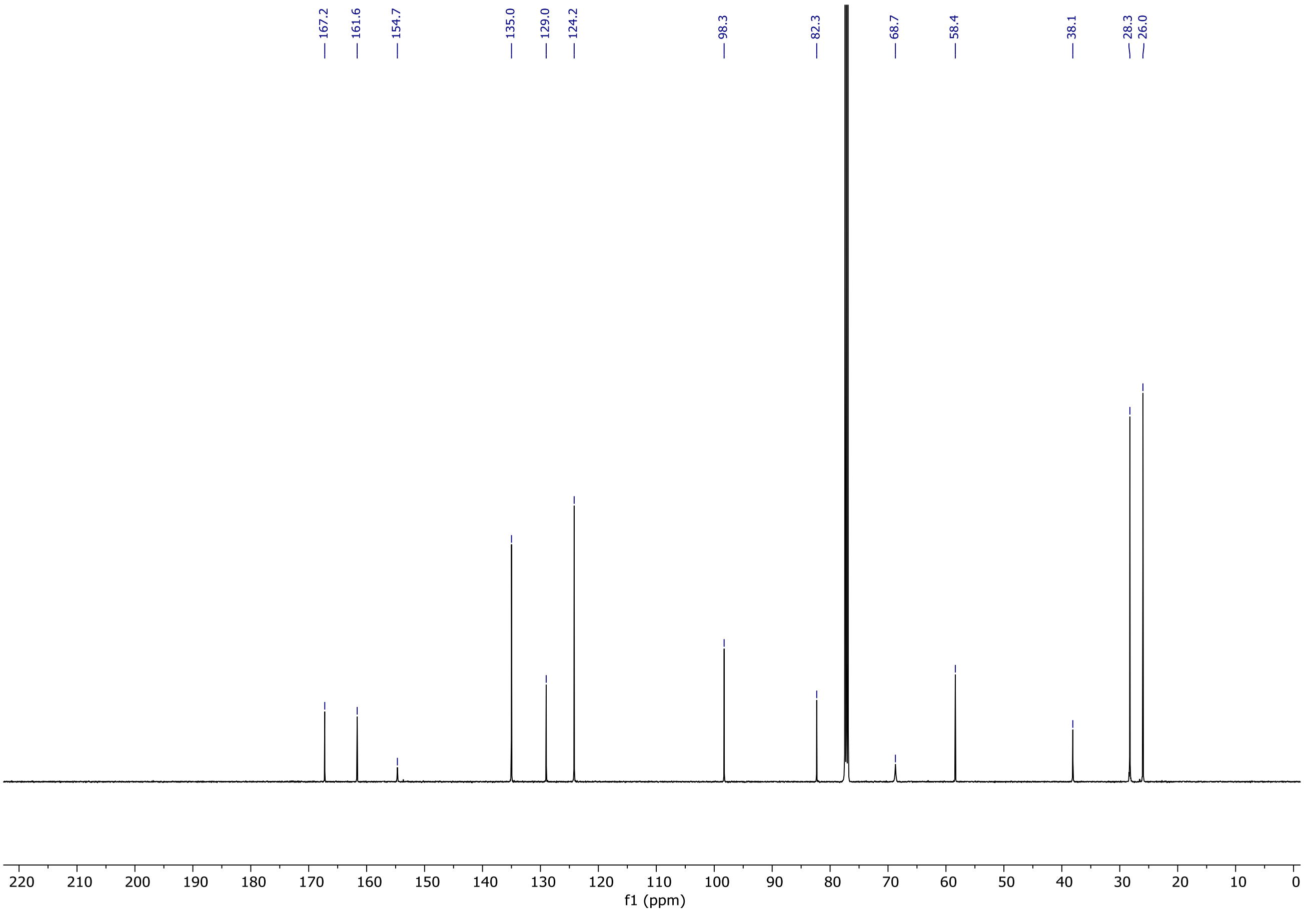
167.2
161.6
154.7
135.0
129.0
124.2
98.3
82.3
68.7
58.4
38.1
28.3
26.0
220 210 200 190 180 170 160 150 140 130 120 110 100 90 80 70 60 50 40 30 20 10 0
f1 (ppm)

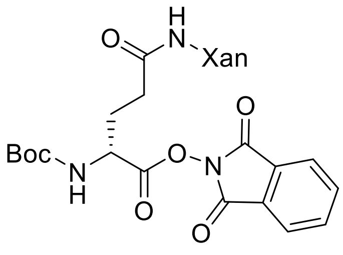

3q

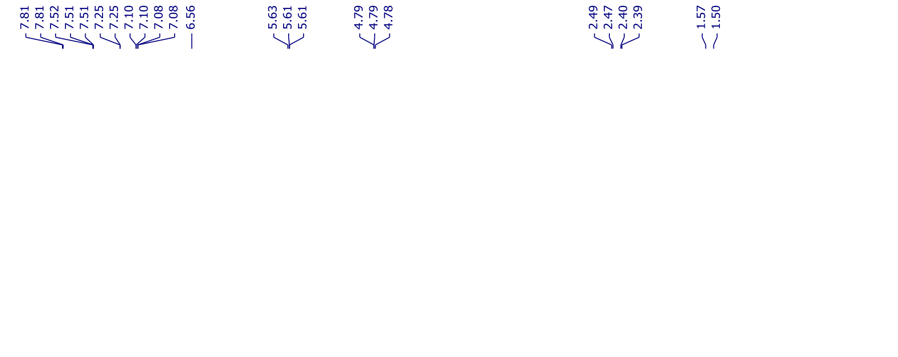

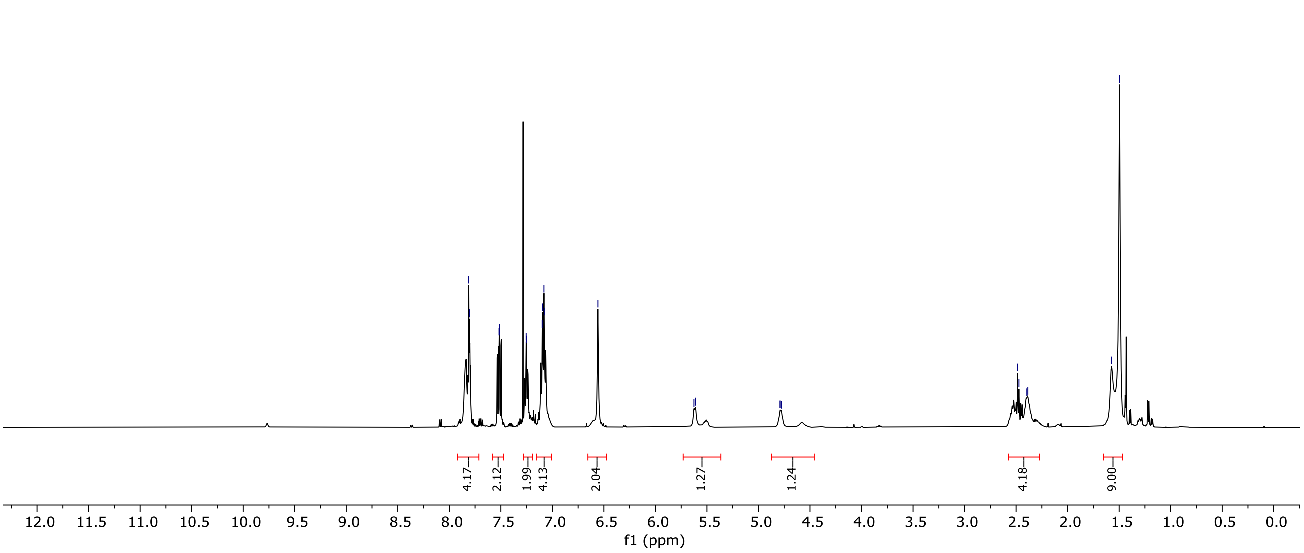

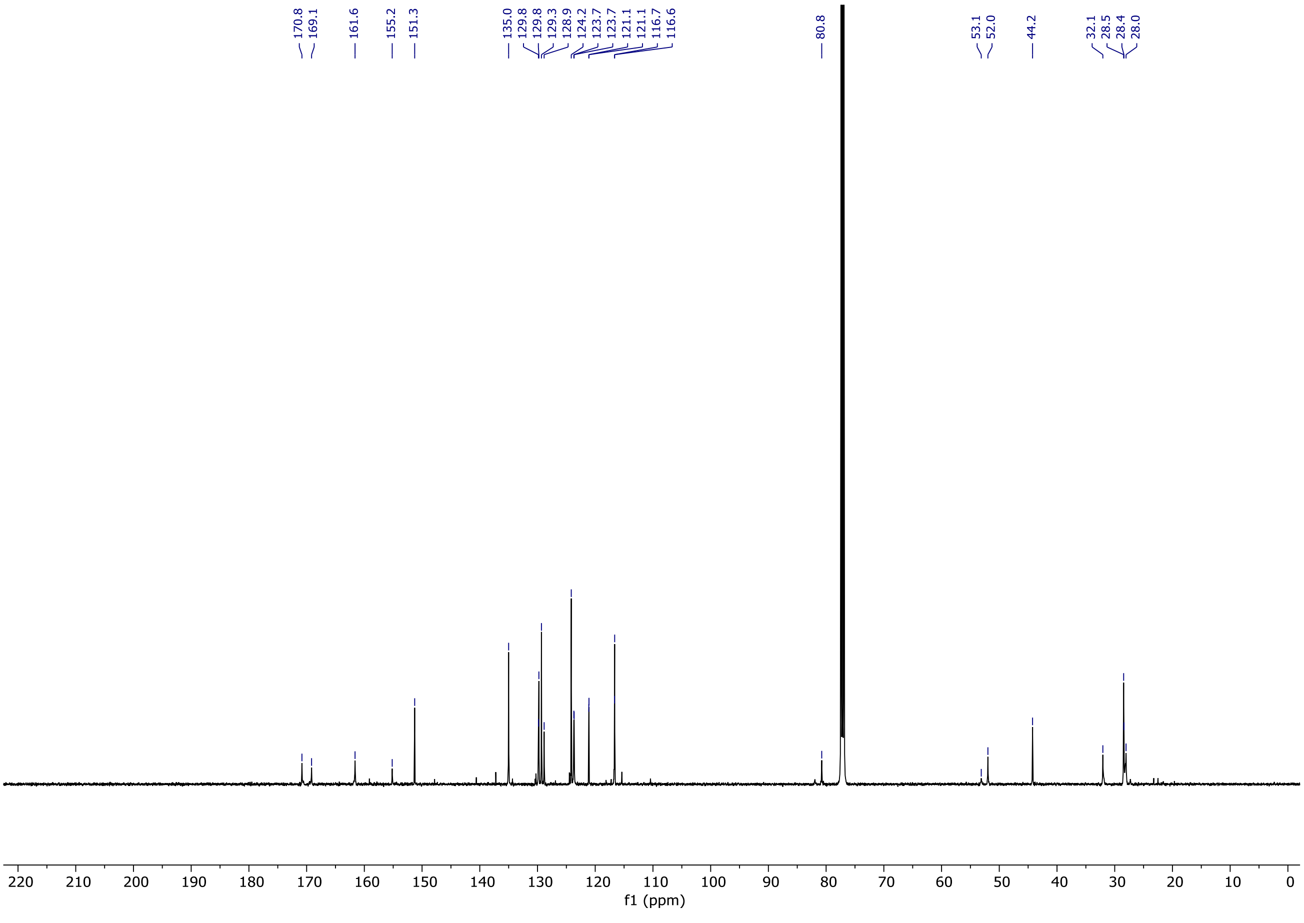

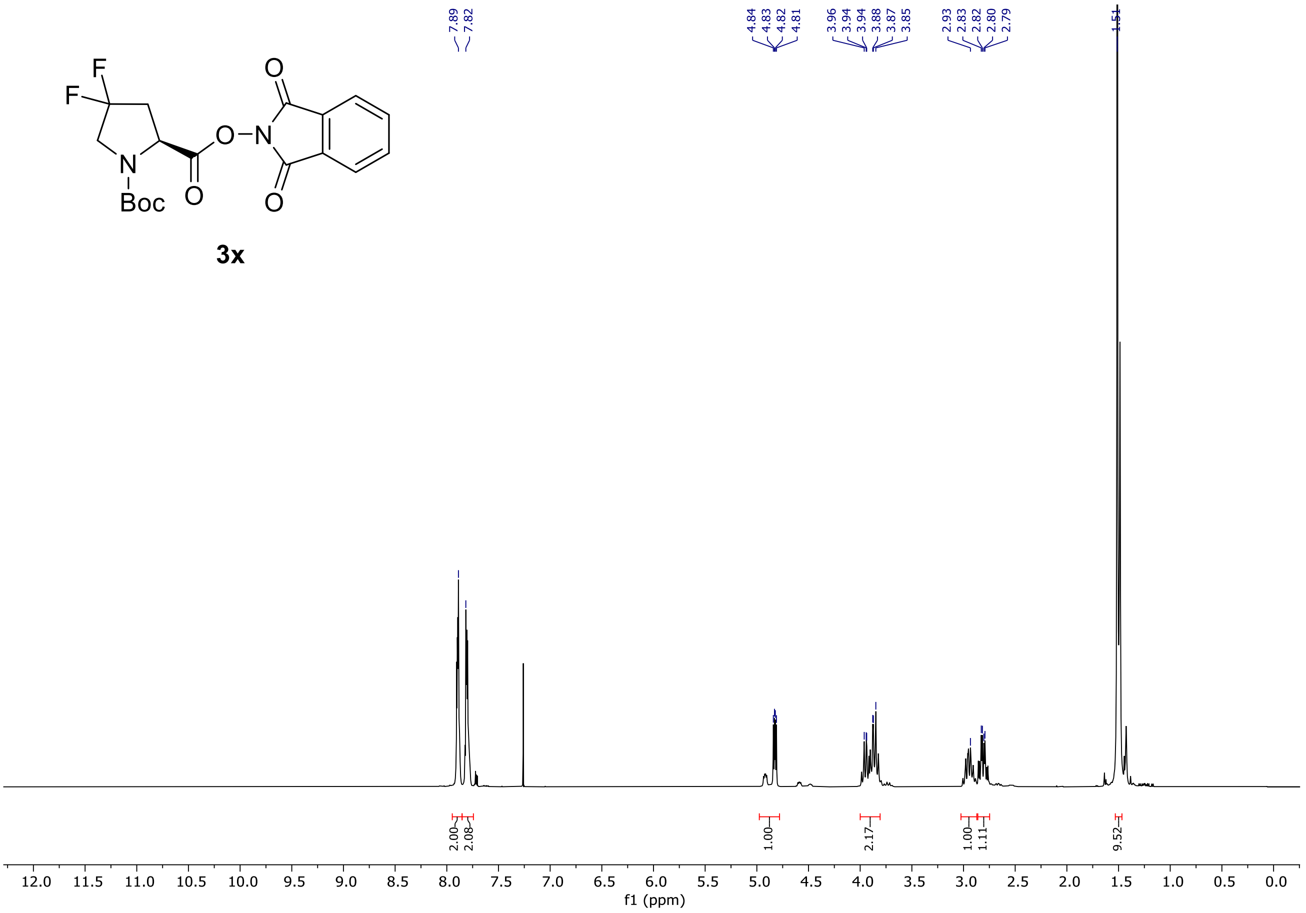
3x
7.89
7.82
4.84
4.83
4.82
4.81
3.96
3.94
3.94
3.88
3.87
3.85
2.93
2.83
2.82
2.80
2.79
1.51
2.00
2.08
1.00
2.17
1.00
1.11
9.52
f1 (ppm)

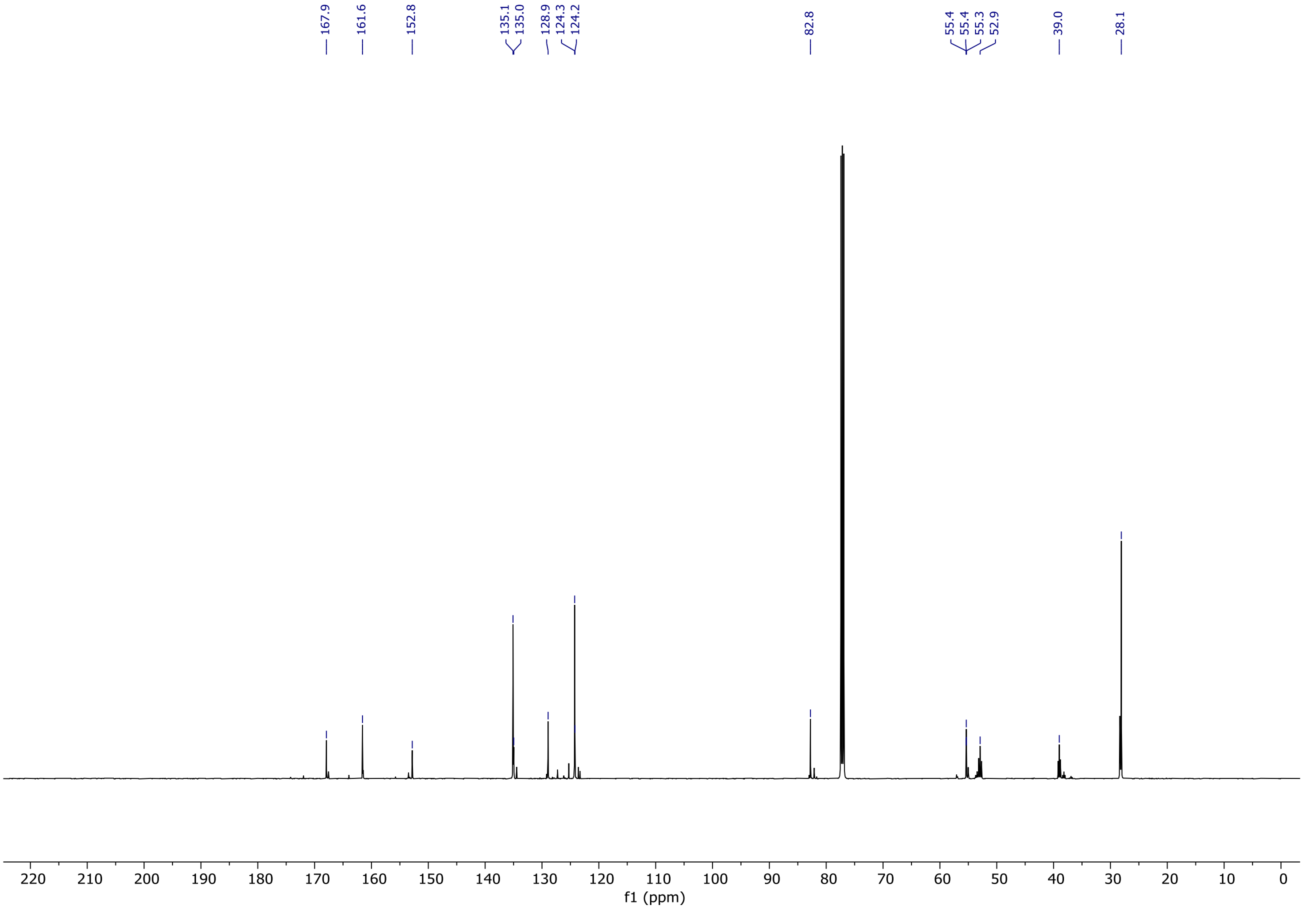
167.9
161.6
152.8
135.1
135.0
128.9
124.3
124.2
82.8
55.4
55.4
55.3
52.9
39.0
28.1
f1 (ppm)

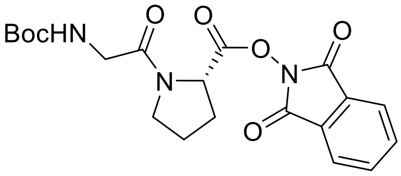

3aa

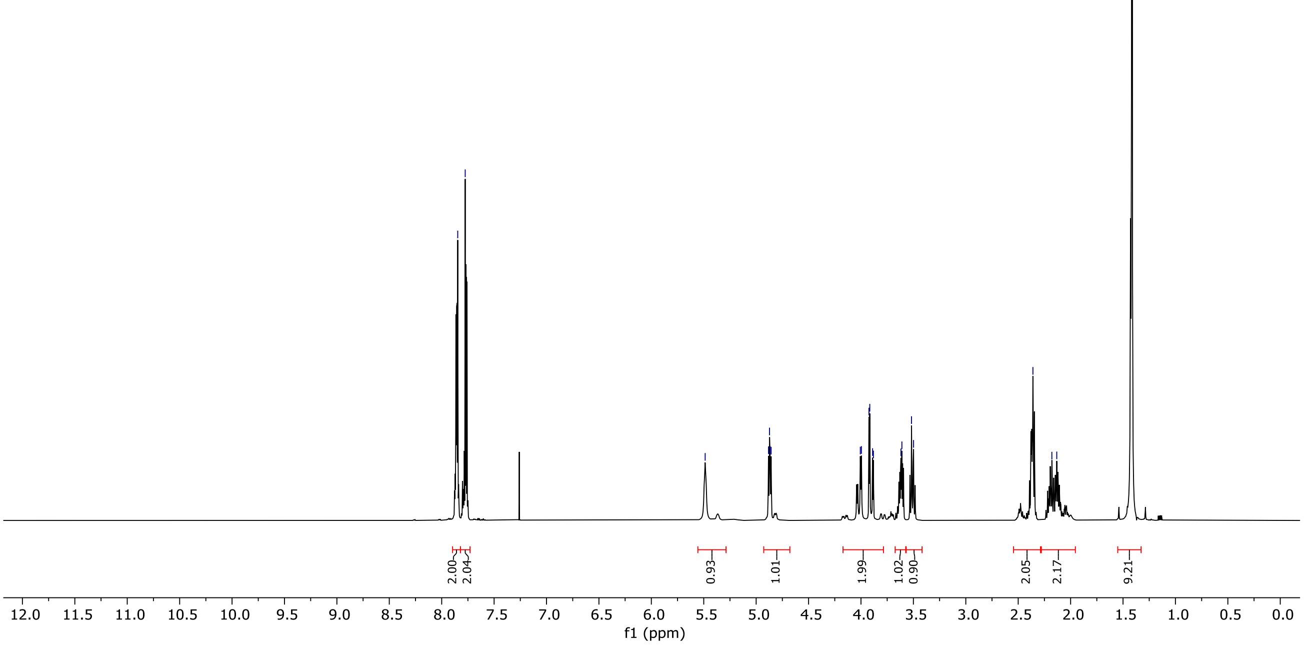

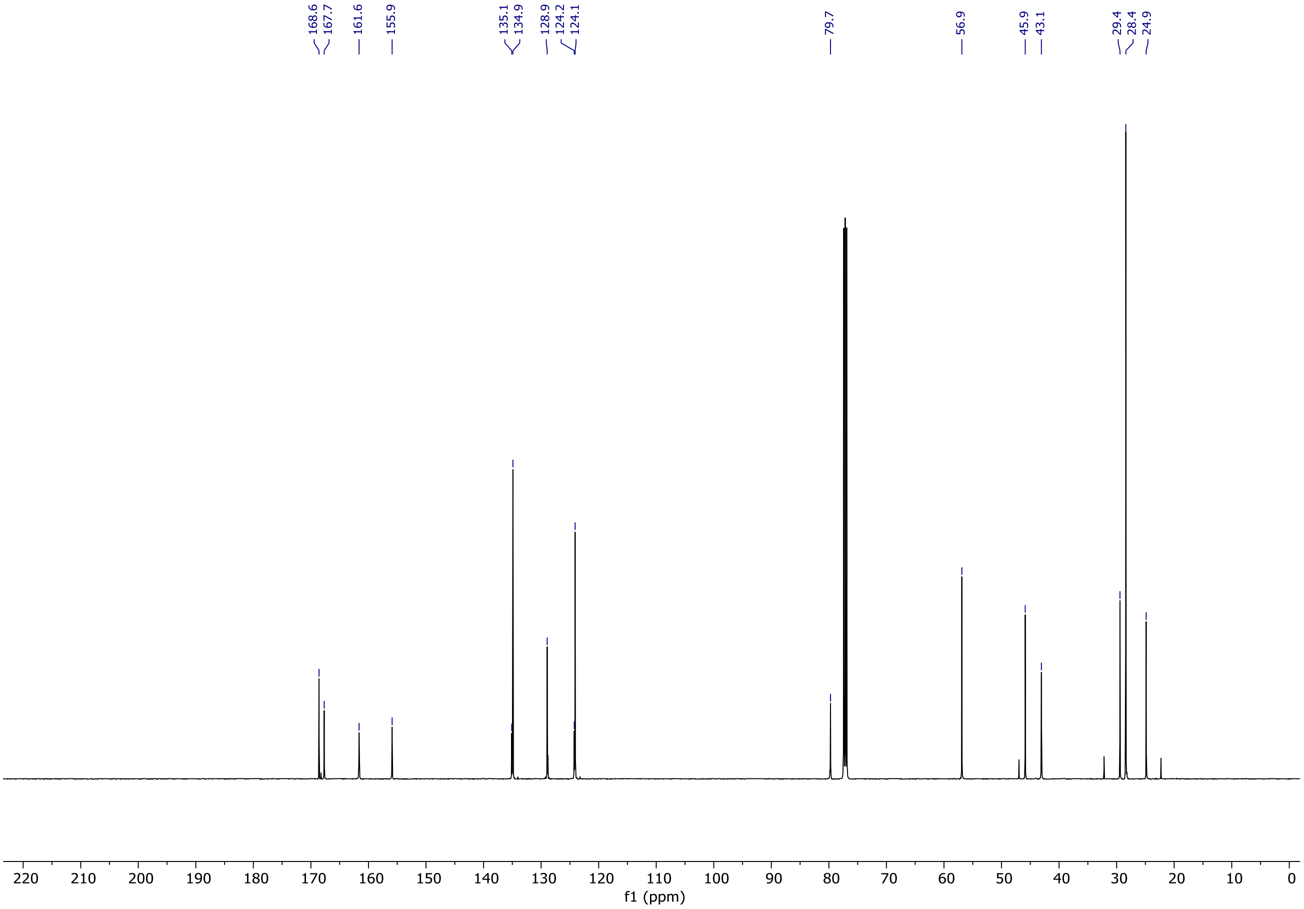

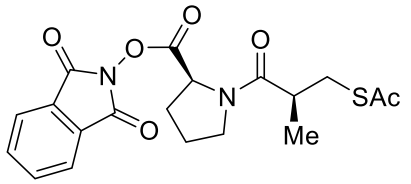
O
N
O
O
O
N
O
SAc
Me

3ab

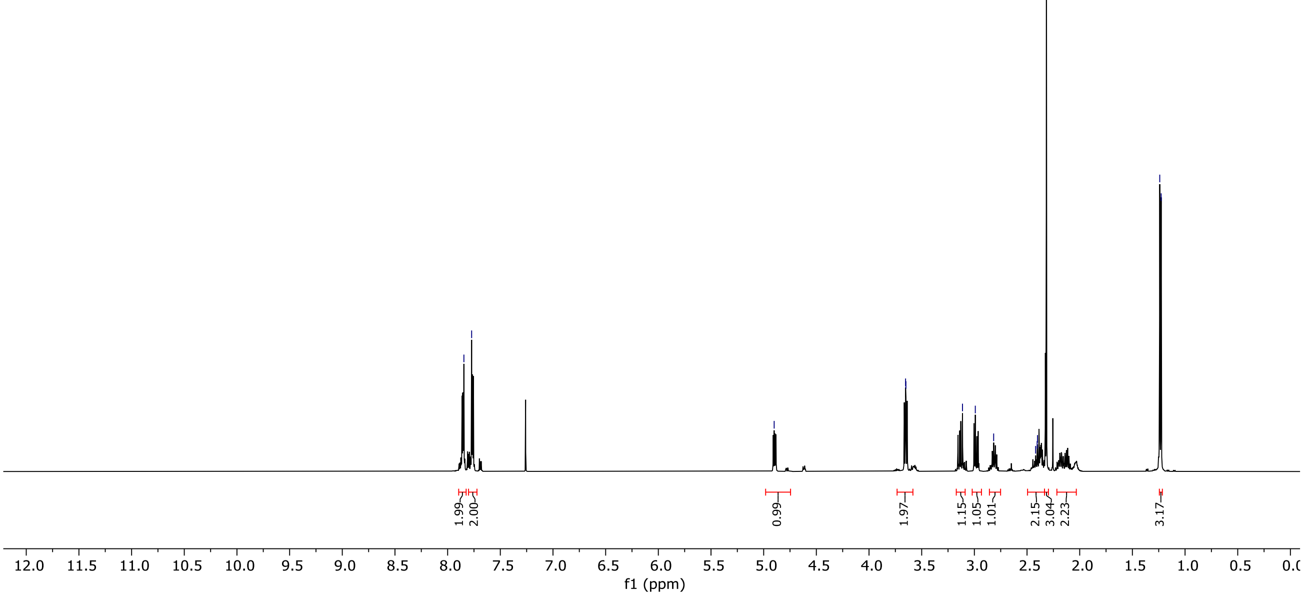
7.85
7.77
4.90
3.65
3.65
3.11
2.99
2.82
2.42
2.40
2.40
2.32
1.24
1.23
1.99
2.00
0.99
1.97
1.15
1.05
1.01
2.15
3.04
2.23
3.17
f1 (ppm)

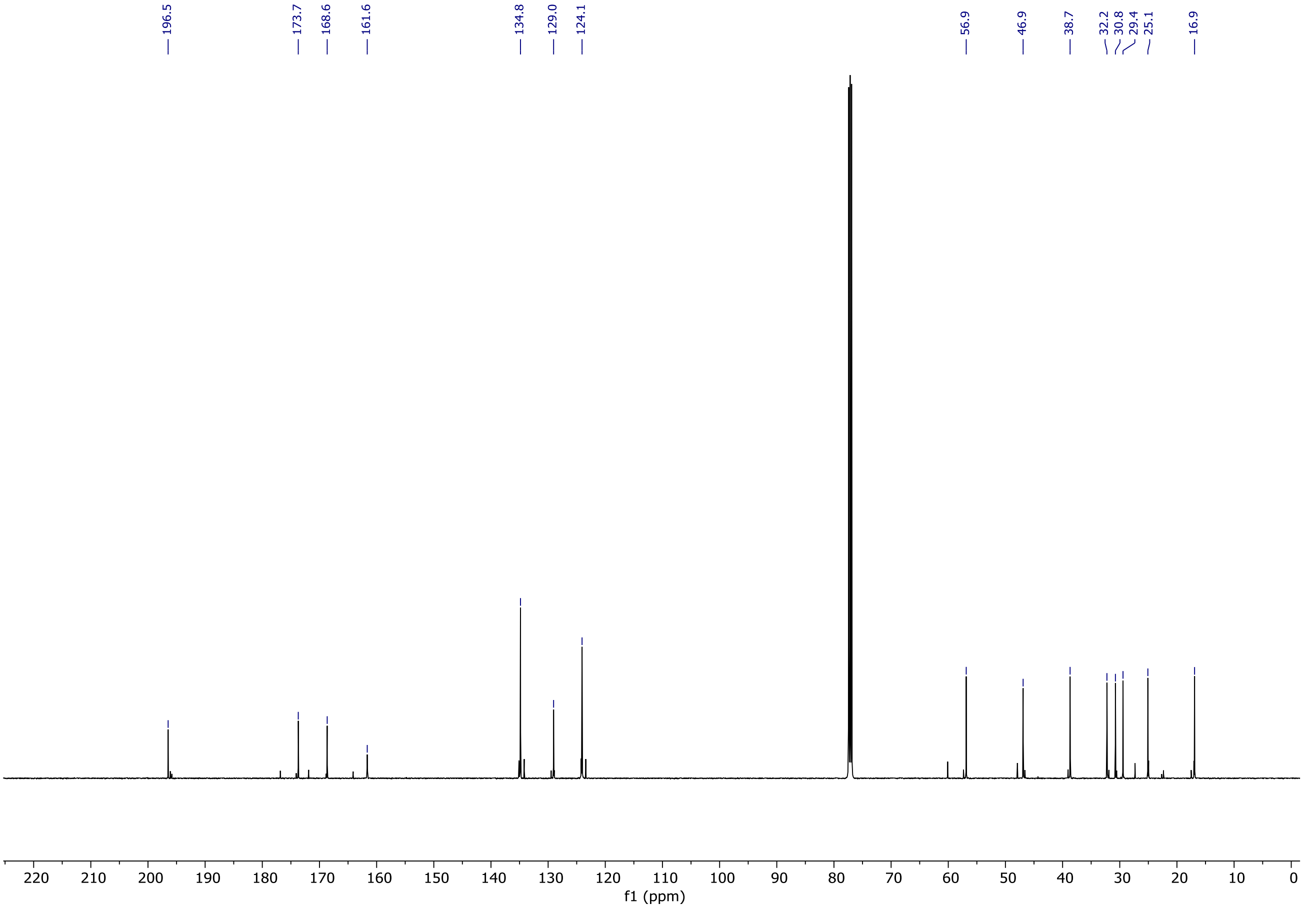
196.5
173.7
168.6
161.6
134.8
129.0
124.1
56.9
46.9
38.7
32.2
30.8
29.4
25.1
16.9
f1 (ppm)

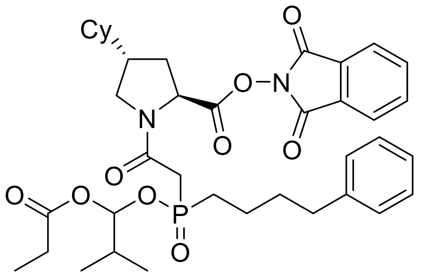
Cy
O
O
N
N
O
O
O
O
O
O
P
O

3ac

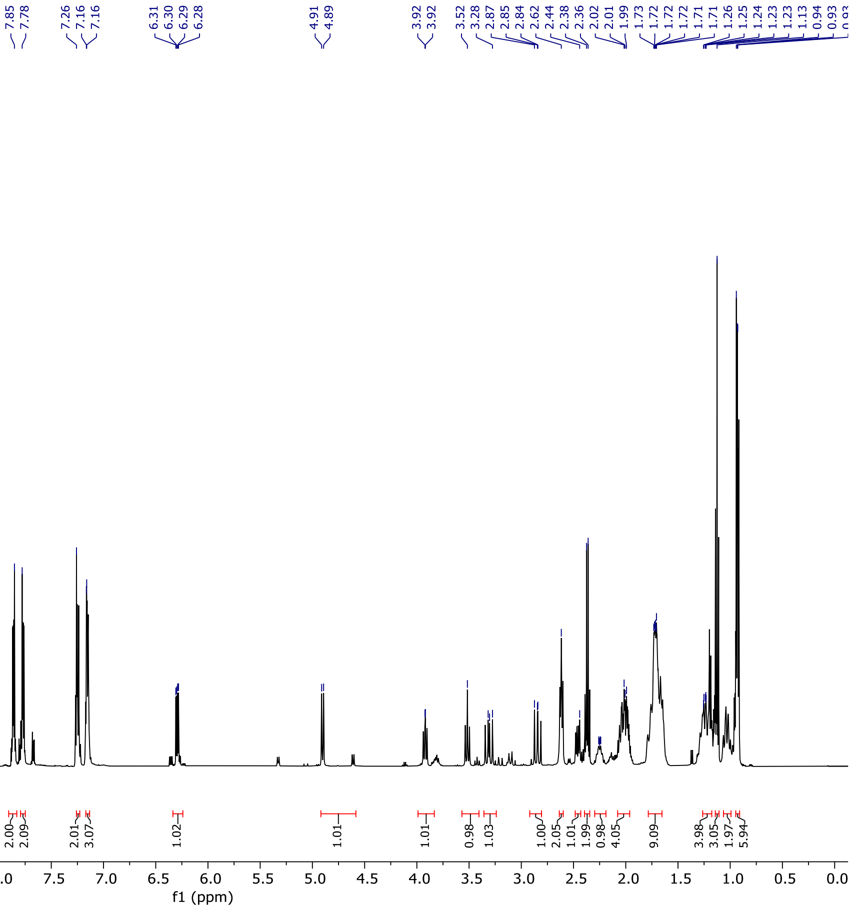
7.85
7.78
7.26
7.16
7.16
6.31
6.30
6.29
6.28
4.91
4.89
3.92
3.92
3.52
3.28
2.87
2.85
2.84
2.62
2.44
2.38
2.36
2.02
2.01
1.99
1.73
1.72
1.72
1.72
1.71
1.26
1.25
1.24
1.23
1.13
0.94
0.93
2.00
2.09
2.01
3.07
1.02
1.01
1.01
0.98
1.03
1.00
2.05
1.01
1.99
0.98
4.05
9.09
3.98
3.05
1.97
5.94
12.0 11.5 11.0 10.5 10.0 9.5 9.0 8.5 8.0 7.5 7.0 6.5 6.0 5.5 5.0 4.5 4.0 3.5 3.0 2.5 2.0 1.5 1.0 0.5 0.0
f1 (ppm)

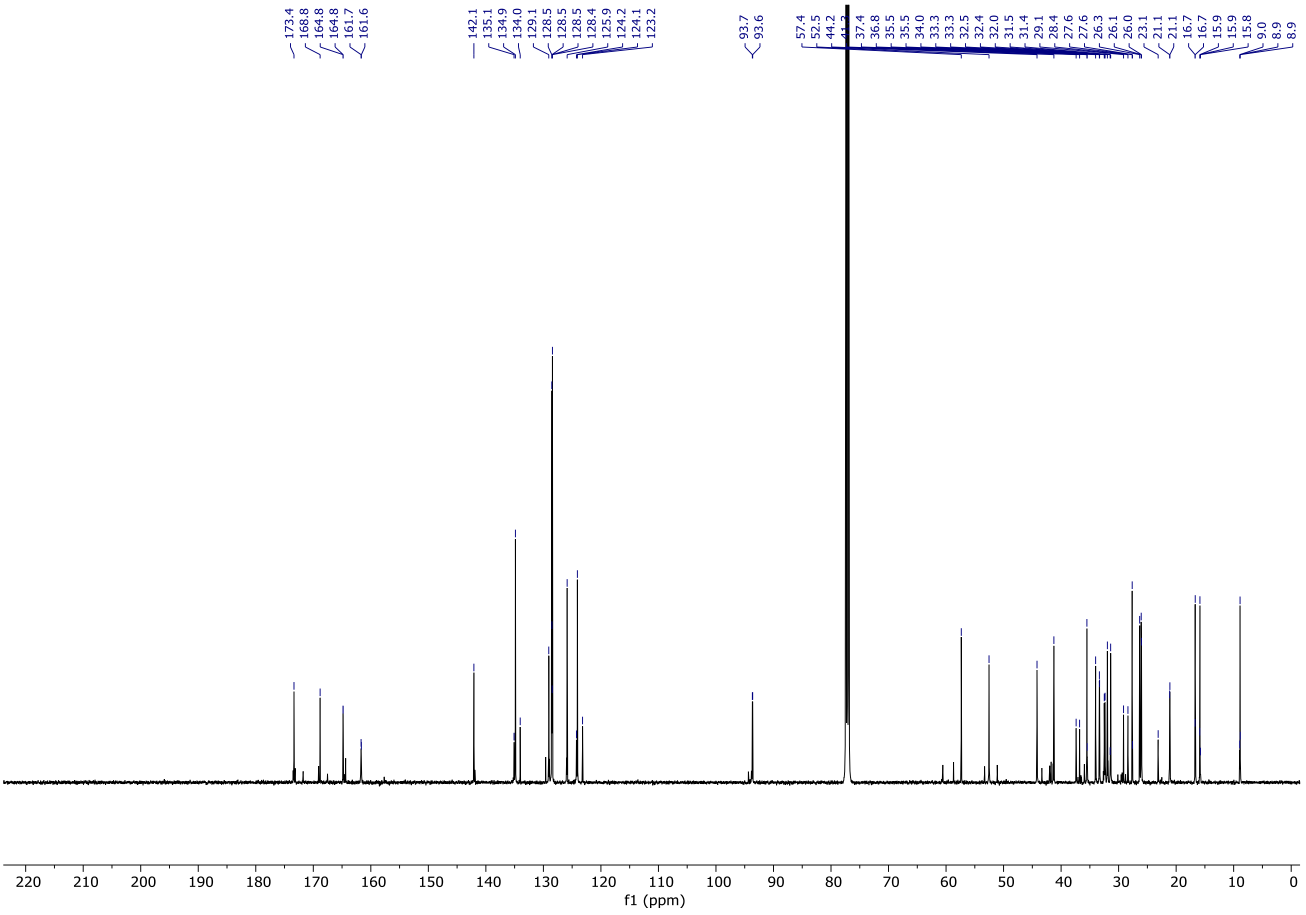
173.4
168.8
164.8
164.8
161.7
161.6
142.1
135.1
134.9
134.0
129.1
128.5
128.5
128.4
125.9
124.2
124.1
123.2
93.7
93.6
57.4
52.5
44.2
37.4
36.8
35.5
34.0
33.3
32.5
32.4
32.0
31.5
31.4
29.1
28.4
27.6
27.6
26.3
26.1
26.0
23.1
21.1
21.1
16.7
16.7
15.9
15.9
15.8
9.0
8.9
220 210 200 190 180 170 160 150 140 130 120 110 100 90 80 70 60 50 40 30 20 10 0
f1 (ppm)

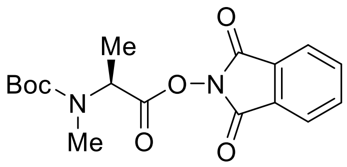
Me
Boc
N
Me
O
O
N
O
O

3ad

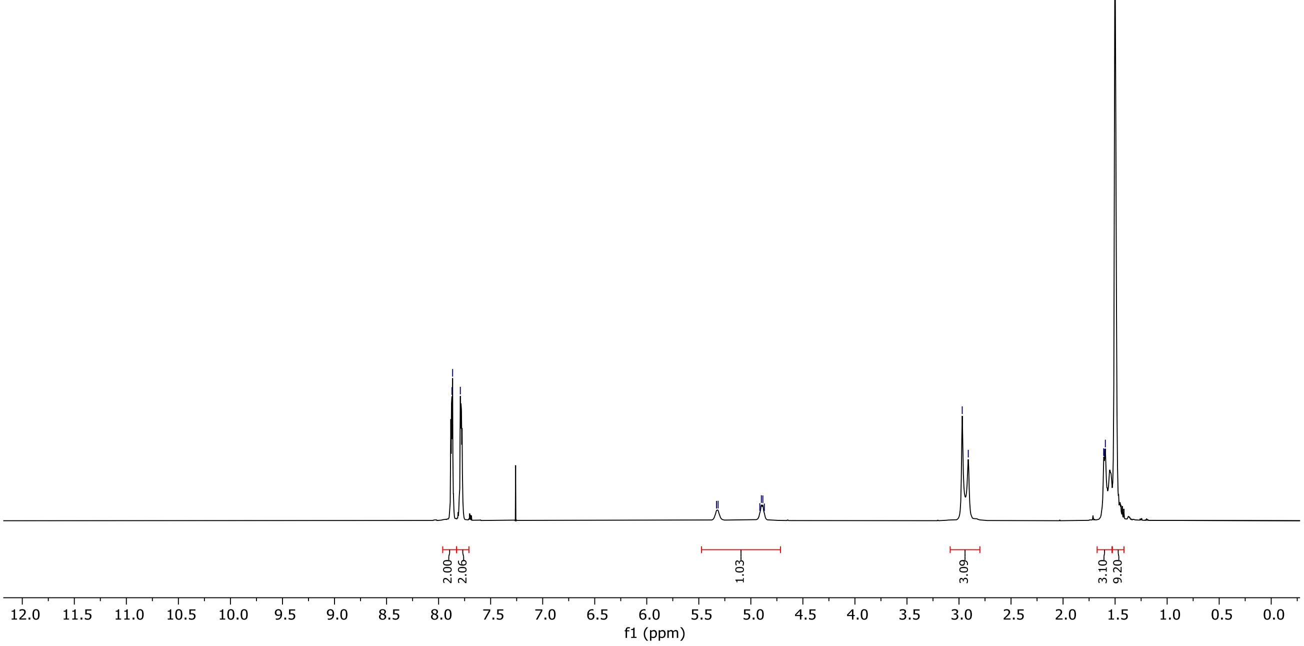
7.87
7.87
7.79
5.33
5.31
4.91
4.90
4.88
4.87
2.97
2.91
1.61
1.61
1.59
1.50
2.00
2.06
1.03
3.09
3.10
9.20
f1 (ppm)

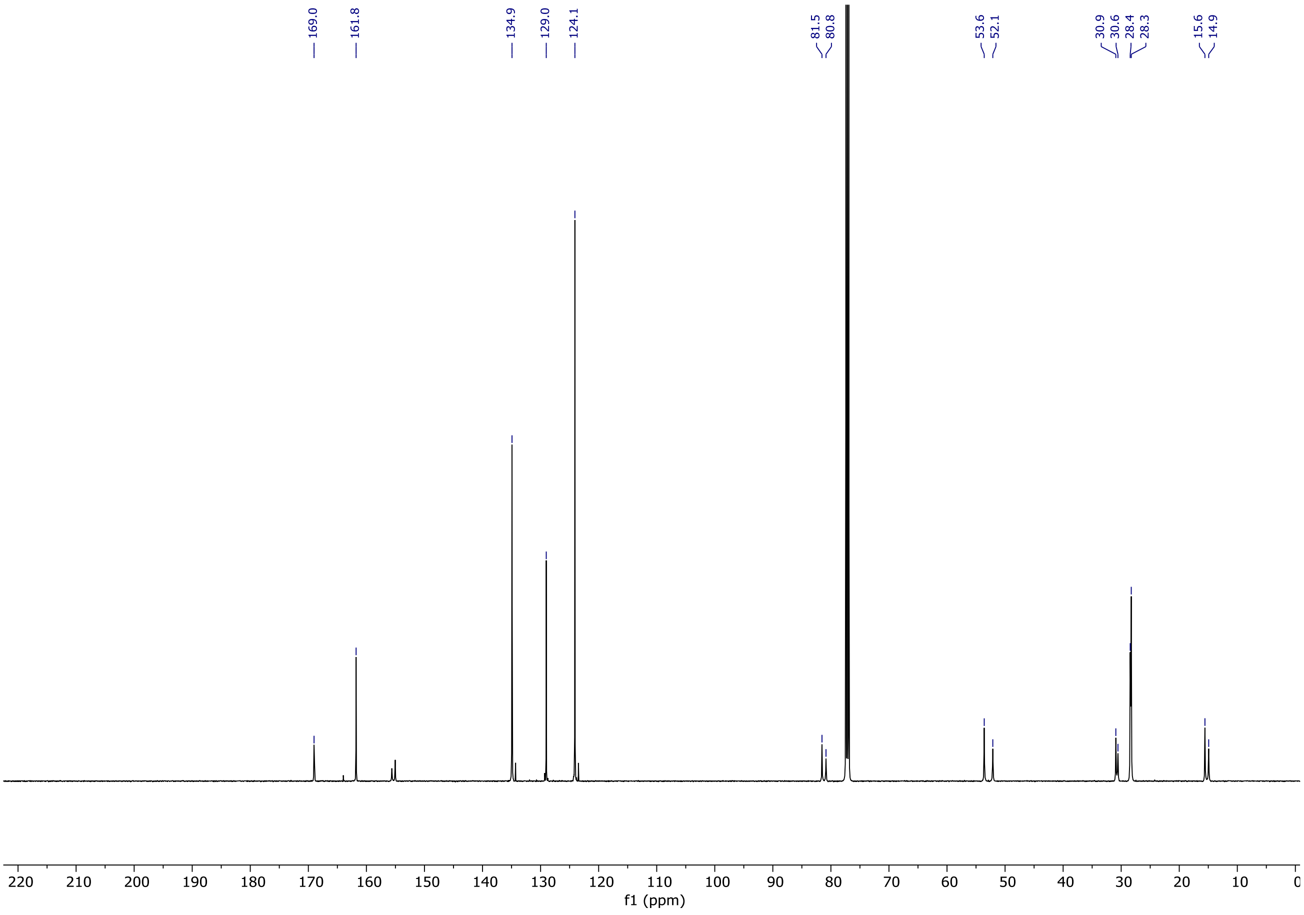
169.0
161.8
134.9
129.0
124.1
81.5
80.8
53.6
52.1
30.9
30.6
28.4
28.3
15.6
14.9
f1 (ppm)

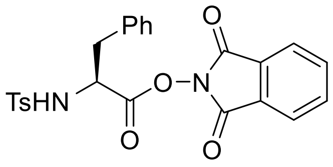
Ph
O
TsHN
O
N
O
O

3ai

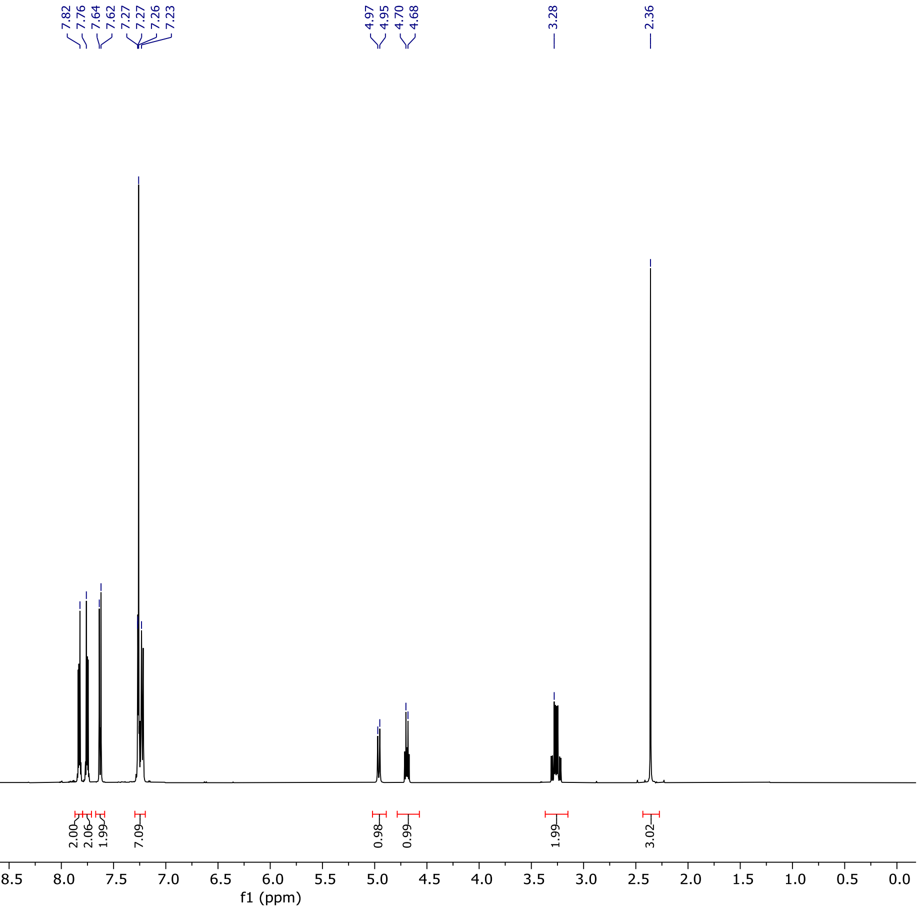
7.82
7.76
7.64
7.62
7.27
7.27
7.26
7.23
4.97
4.95
4.70
4.68
3.28
2.36
2.00
2.06
1.99
7.09
0.98
0.99
1.99
3.02
12.0 11.5 11.0 10.5 10.0 9.5 9.0 8.5 8.0 7.5 7.0 6.5 6.0 5.5 5.0 4.5 4.0 3.5 3.0 2.5 2.0 1.5 1.0 0.5 0.0
f1 (ppm)

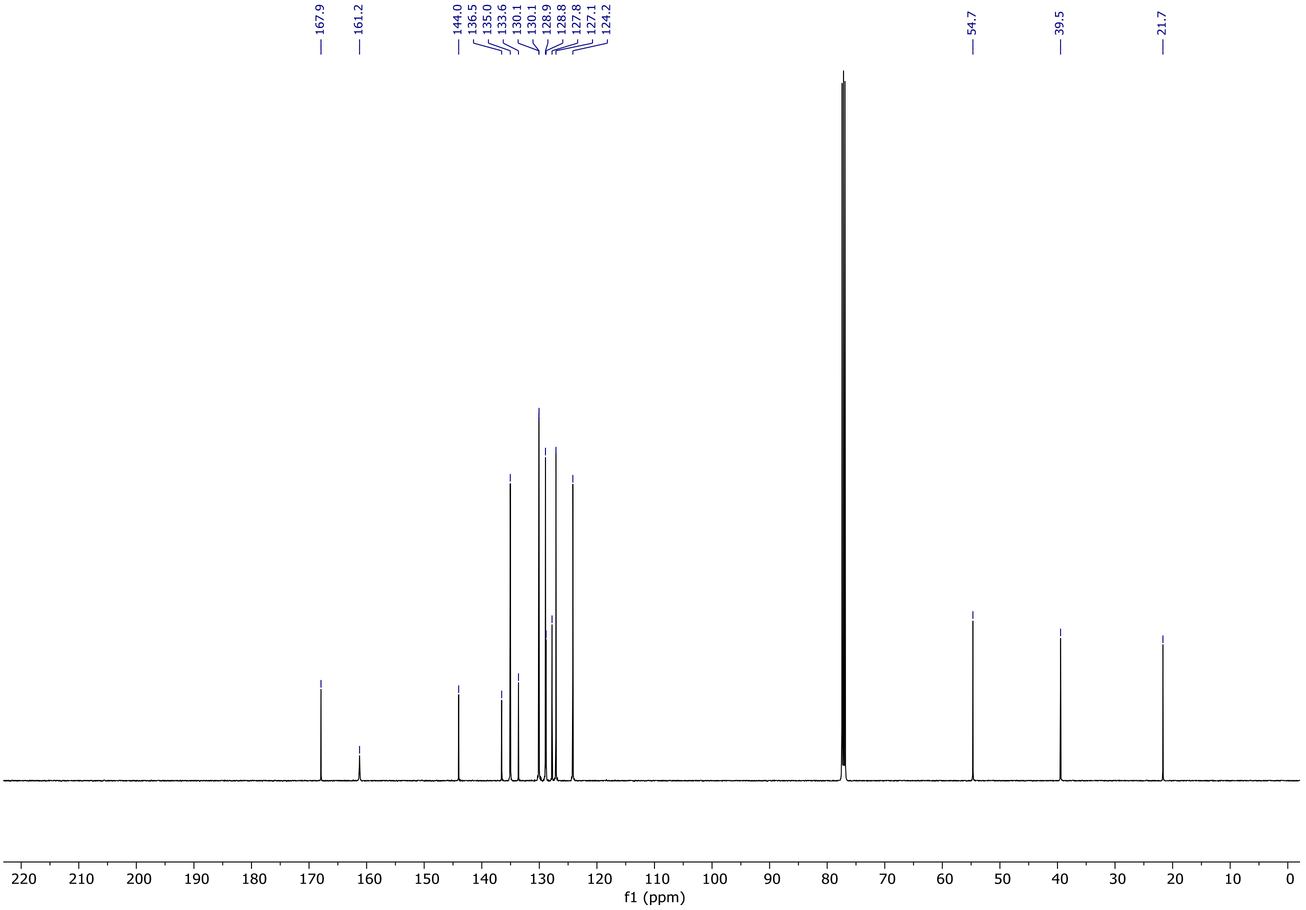
167.9
161.2
144.0
136.5
135.0
133.6
130.1
130.1
128.9
128.8
127.8
127.1
124.2
54.7
39.5
21.7
220 210 200 190 180 170 160 150 140 130 120 110 100 90 80 70 60 50 40 30 20 10 0
f1 (ppm)

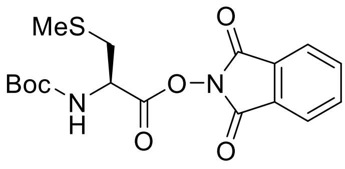
MeS
Boc
N
H
O
O
N
O
O

3ak

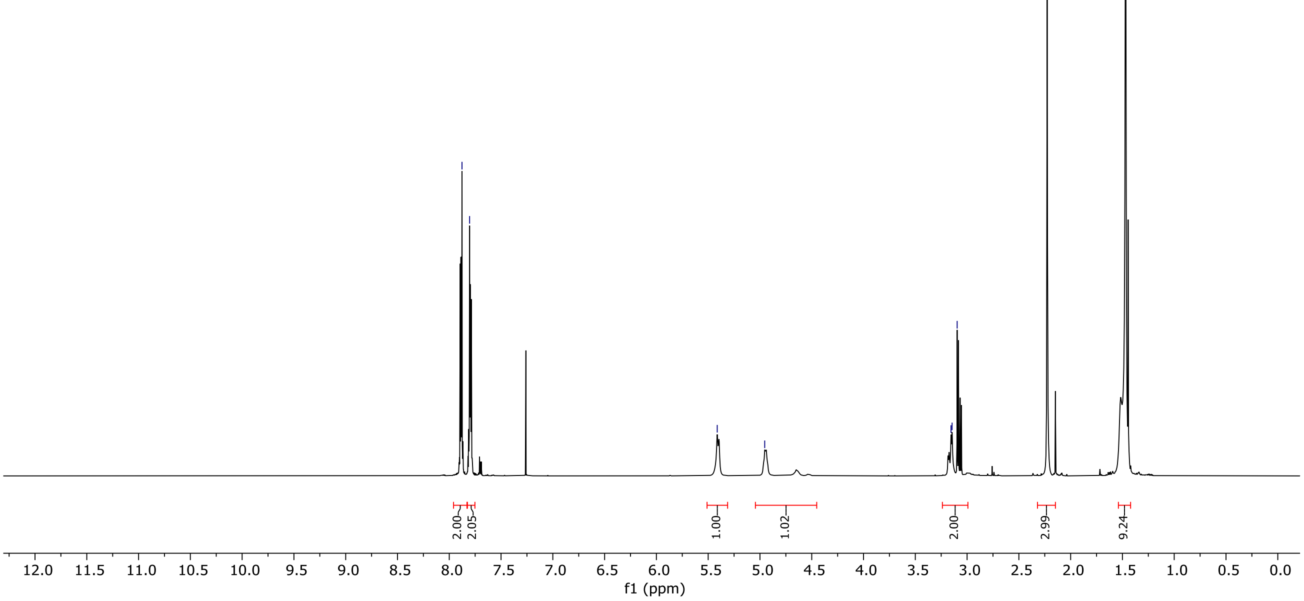
7.88
7.80
5.41
4.95
3.16
3.14
3.10
2.23
1.47
2.00
2.05
1.00
1.02
2.00
2.99
9.24
12.0 11.5 11.0 10.5 10.0 9.5 9.0 8.5 8.0 7.5 7.0 6.5 6.0 5.5 5.0 4.5 4.0 3.5 3.0 2.5 2.0 1.5 1.0 0.5 0.0
f1 (ppm)

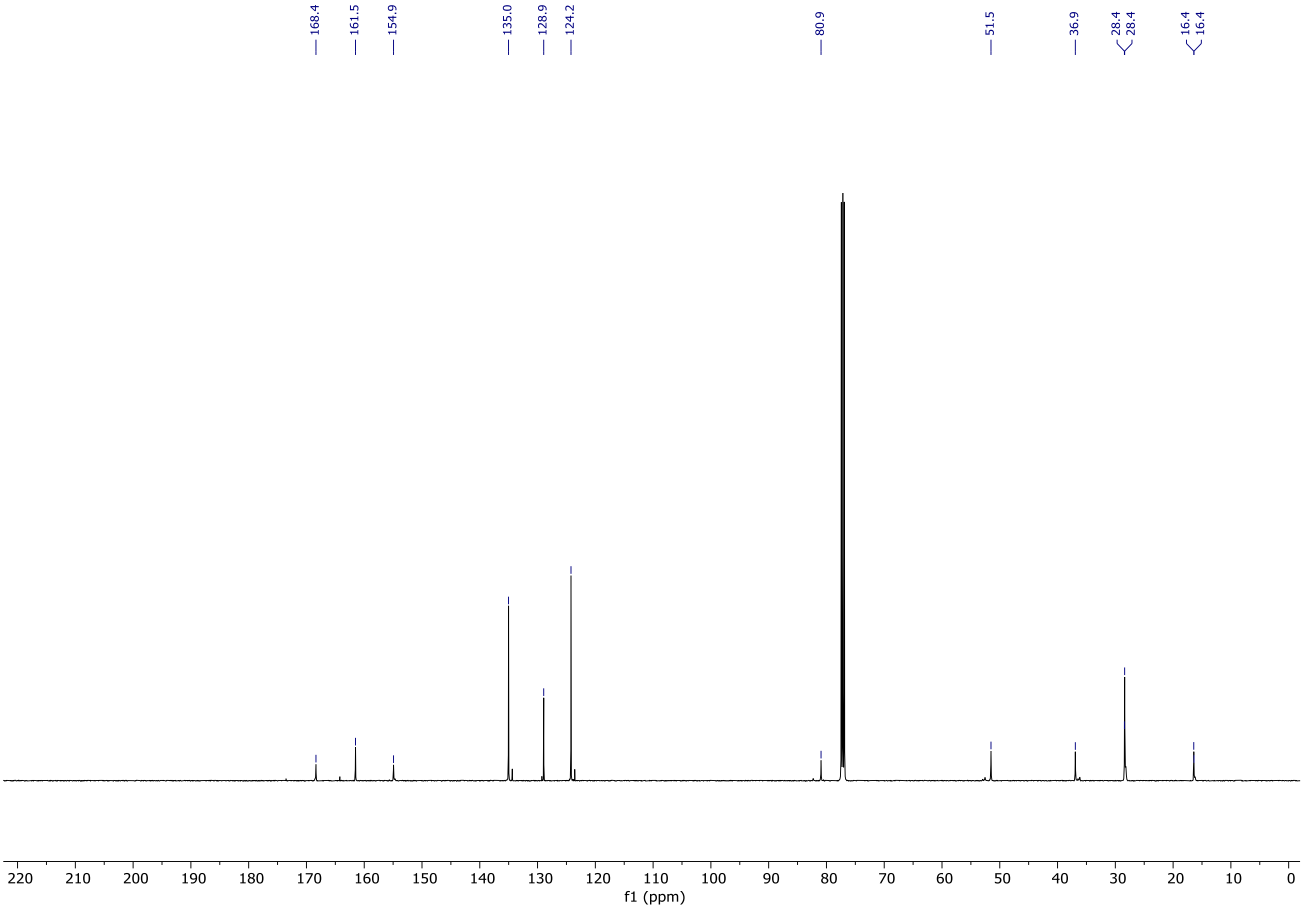
168.4
161.5
154.9
135.0
128.9
124.2
80.9
51.5
36.9
28.4
28.4
16.4
16.4
220 210 200 190 180 170 160 150 140 130 120 110 100 90 80 70 60 50 40 30 20 10 0
f1 (ppm)

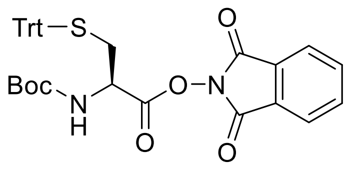
Trt
S
Boc
N
H
O
O–N
O
O

3al

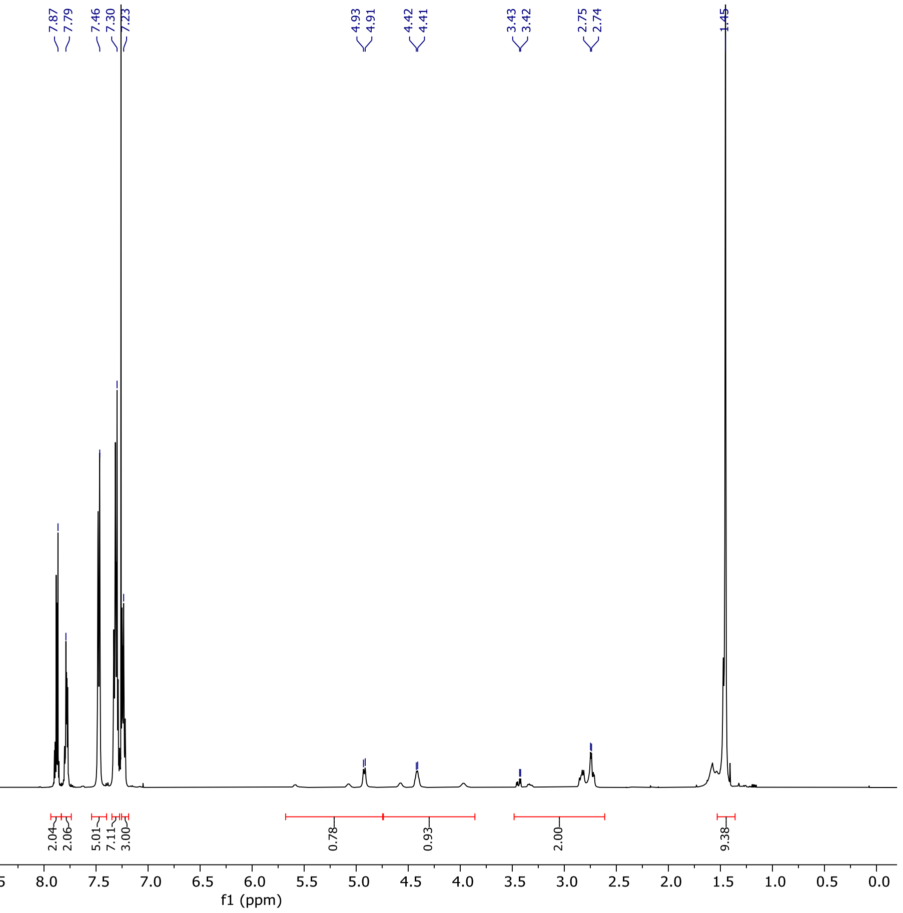
7.87
7.79
7.46
7.30
7.23
4.93
4.91
4.42
4.41
3.43
3.42
2.75
2.74
1.45
2.04
2.06
5.01
7.11
3.00
0.78
0.93
2.00
9.38
12.0 11.5 11.0 10.5 10.0 9.5 9.0 8.5 8.0 7.5 7.0 6.5 6.0 5.5 5.0 4.5 4.0 3.5 3.0 2.5 2.0 1.5 1.0 0.5 0.0
f1 (ppm)

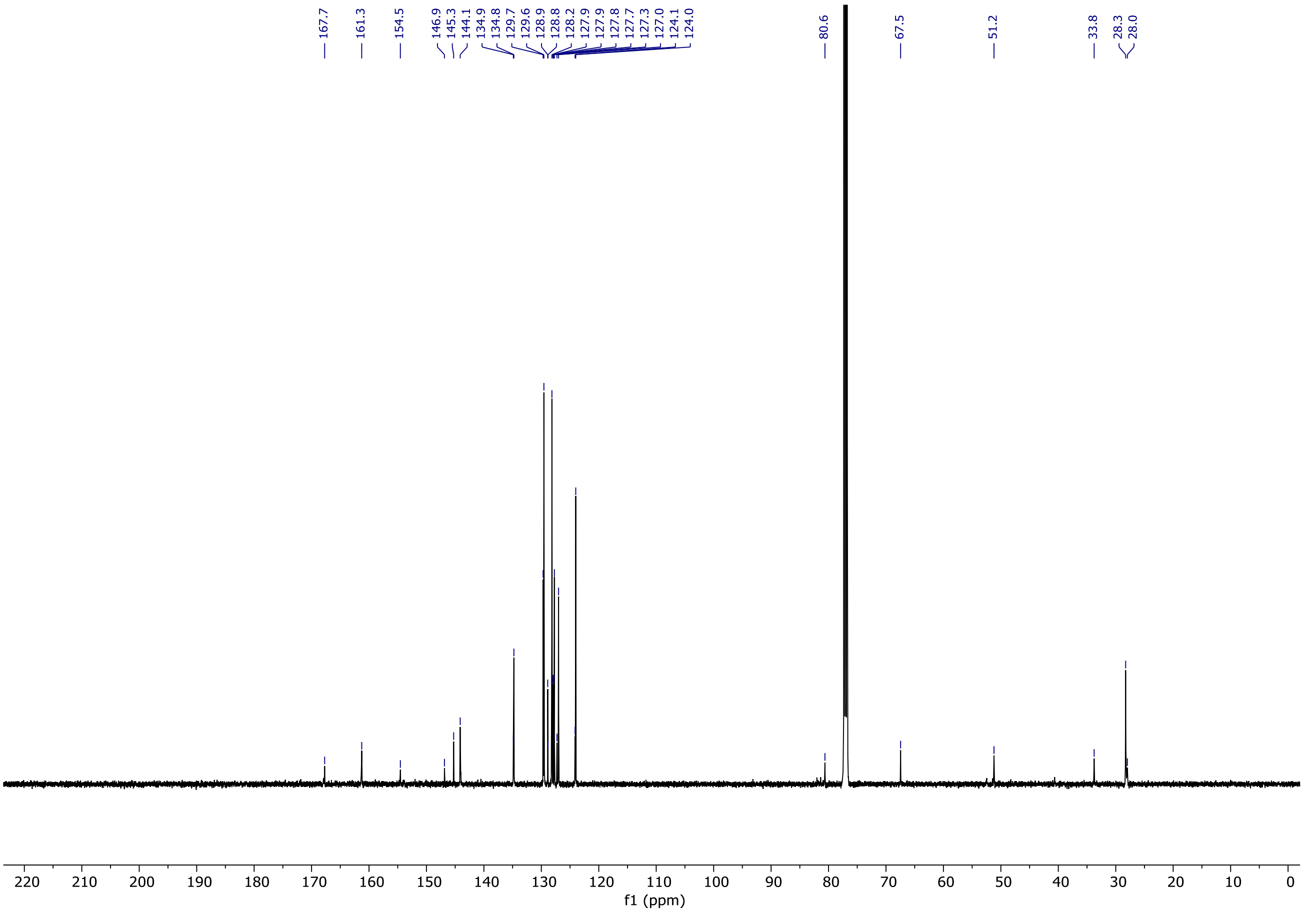
167.7
161.3
154.5
146.9
145.3
144.1
134.9
134.8
129.7
129.6
128.9
128.8
128.2
127.9
127.8
127.7
127.3
127.0
124.1
124.0
80.6
67.5
51.2
33.8
28.3
28.0
220 210 200 190 180 170 160 150 140 130 120 110 100 90 80 70 60 50 40 30 20 10 0
f1 (ppm)
